# Post-transcriptional modulation of the SigF regulon in *Mycobacterium smegmatis* by the PhoH2 toxin-antitoxin

**DOI:** 10.1371/journal.pone.0236551

**Published:** 2020-07-29

**Authors:** Emma S. V. Andrews, Elizabeth Rzoska-Smith, Vickery L. Arcus

**Affiliations:** School of Science, Division of Health, Engineering, Computing and Science, University of Waikato, Hamilton, New Zealand; University of British Columbia, CANADA

## Abstract

PhoH2 proteins are highly conserved across bacteria and archaea yet their biological function is poorly characterised. We examined the growth profiles of *Mycobacterium smegmatis* strains *mc*^*2*^*155* and *mc*^*2*^*155 ΔphoH2* and observed the same growth profile and growth rate in a variety of conditions. In light of the comparable growth, we used RNAseq to provide a snapshot of the differences between the transcriptomes of *M*. *smegmatis mc*^*2*^*155* and *M*. *smegmatis mc*^*2*^*155 ΔphoH2* during normal growth. At 48 hours, elevated expression of the *sigF* regulon was observed in *ΔphoH2* relative to wild type. In biochemical assays, PhoH2 showed activity toward *sigF* mRNA insinuating a role of PhoH2 in modulating the pool of *sigF* mRNA in the cell during normal growth, adding further complexity to the repertoire of reported mechanisms of post-translational regulation. Multiple copies of the preferred target site of PhoH2 were identified in loops of the *sigF* mRNA structure, leading us to propose a mechanism for the activity of PhoH2 that is initiated after assembly on specific single-stranded loops of RNA. We hypothesise that PhoH2 is a toxin-antitoxin that contributes to the regulation of SigF at a post-transcriptional level through targeted activity on *sigF* mRNA. This work presents the first evidence for post-transcriptional regulation of SigF along with the biological function of PhoH2 from *M*. *smegmatis*. This has implications for the highly conserved PhoH2 toxin-antitoxin module across the mycobacteria including the important human pathogen *M*. *tuberculosis*.

## Introduction

Sigma factors initiate gene expression through their interaction with RNAP [1]. Their function directs the binding of RNAP to specific promoter sites to initiate transcription of specific subsets of genes [1]. In mycobacteria, sigma factors SigA and SigB are responsible for the expression of essential genes [2]. Alternate sigma factors function to coordinate gene regulation in response to different environmental stresses and changing physiological conditions [3]. One such alternate sigma factor, SigF, is involved in the cell’s adaptation to stationary phase, heat, oxidative stress and antimicrobials [4–6]. SigF has been shown to regulate cell wall composition through the modulation of lipid biosynthesis [7] suggesting a prominent role in mycobacterial cell wall structure and function, persistence and pathogenesis [8]. Evidence in the literature points towards complex, post-translational regulation of SigF, through the activity of anti-sigma factors and their antagonists [6, 7, 9] with no reports regarding regulation at the post-transcriptional level.

**Fig 1:**
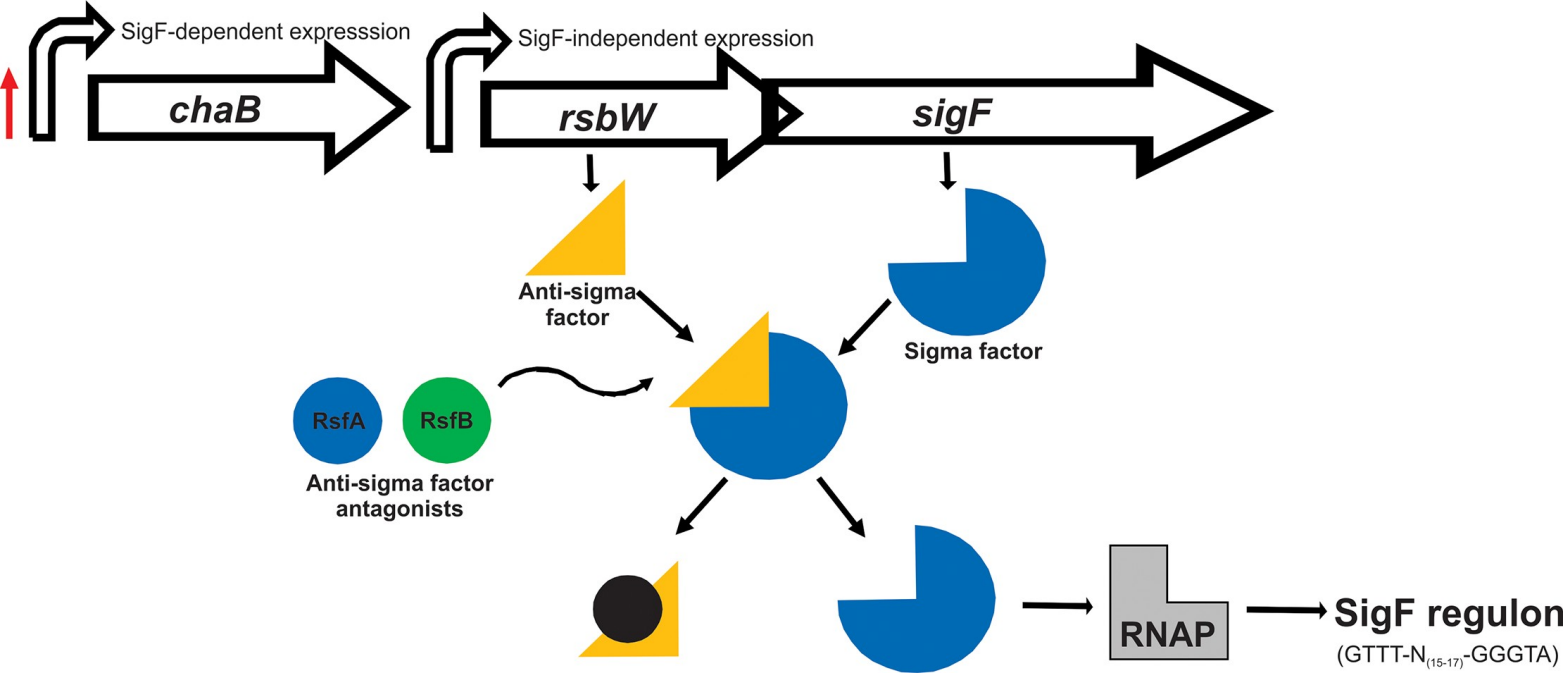
Schematic of the genomic arrangement of *sigF* from *M*. *smegmatis* and its regulation.

The genomic arrangement of *sigF* is conserved among mycobacteria [4] (Fig 1). In *Mycobacterium smegmatis* the gene encoding *sigF* is co-transcribed with anti-sigma factor RsbW (MSMEG_1803) and a protein of unknown function, ChaB (MSMEG_1802), from two promoter sites; one upstream of MSMEG_1802, and one upstream of *rsbW* [10]. Expression from the promoter positioned upstream of *chaB* is dependent on SigF and shows a 2-fold increase in transcription upon entry into stationary phase [10]. Expression from the second promoter position, upstream of *rsbW*, is independent of SigF and is constitutive throughout growth and exposure to stress [10]. The regulon of genes controlled by SigF share a consensus promoter sequence (GTTT-N_(15–17)_-GGGTA) [6].

The gene encoding *sigF* is co-expressed with *rsbW* and *chaB* from two promoter sites (indicated by large curved arrows). During normal growth the activity of SigF is sequestered by is co-expressed, cognate anti-sigma factor RsbW. Upon entry into stationary phase, the expression of the *sigF* transcript increases 2x fold from the promoter site located upstream of *chaB* (indicated by the red arrow). In response to stationary phase and/or environmental cues, anti-sigma factor antagonists interact with RsbW in a negative fashion, lifting the repression of SigF, permitting its interaction with RNAP and expression of its regulon of genes that share the consensus promoter binding sequence GTTT-N_(15–17)_-GGGTA.

The activity of SigF in *Mycobacterium tuberculosis* is post-translationally regulated by anti-sigma factor UsfX [9] along with two anti-sigma factor antagonists, RsfA and RsfB that further regulate UsfX in a negative fashion [9]. In *M*. *smegmatis*, a second copy of a *rsbW* gene, (MSMEG_1787), is encoded in the genome and may pose as a second anti-sigma factor of SigF [6]. There are also a number of predicted anti-sigma factor antagonists [6] (similar to *M*. *tuberculosis* [11]). Two, RsfA (MSMEG_1786) and RsfB (MSMEG_6127) have been shown to interact with RsbW [7] and if these function similarly to their counterparts in *M*. *tuberculosis*, are regulated by two different environmental cues, redox potential and phosphorylation respectively [9].

PhoH2 is a modular enzyme with a PIN-domain RNAse fused with a PhoH-domain RNA helicase [12, 13]. In mycobacteria, PhoH2 is co-transcribed with a short, upstream gene, *phoAT*, whose protein product interacts with PhoH2 [12]. This small protein is comparable to VapB of VapBC (PIN-domain) toxin-antitoxin systems, where VapB functions as a transient inhibitor of VapC (PIN-domain) [14]. Experimental investigations show that PhoH2 from *M*. *tuberculosis* and *M*. *smegmatis* has sequence-specific RNAse and ATP-dependent helicase activities, with preference for double-stranded RNA that contains a 5’-3’ overhang, and where the terminal combination of RNA bases is 5’- A C [A/U] [A/U] [G/C] [12]. This suggests that PhoH2, similar to other PIN-domain containing proteins [14], is a toxin-antitoxin with additional RNA helicase activity that acts on specific RNA substrates and is likely to play a role in the adaptive response to changing environmental conditions [12, 13].

Outside the mycobacteria PhoH2 has been shown to play a role in regulating the infection process of *Synechococcus sp*. *strain WH8102* by Cyanomyovirus [15]. In *Corynebacterium glutamicum*, PhoH2 is implicated in the response to phosphate limitation [16] and there is evidence for reduced expression of *phoH2* during the transition from exponential growth to stationary phase growth [17]. This suggests that the expression of *phoH2* is not under control of the prominent sigma factor, SigB (analogous to SigF from *M*. *smegmatis*) during this phase of growth.

To determine the biological function of PhoH2 from *M*. *smegmatis* we initially examined growth profiles of *M*. *smegmatis mc*^*2*^*155* and *M*. *smegmatis mc*^*2*^*155 ΔphoH2* under rich, defined and nutrient limiting conditions. Under standard rich conditions, RNAseq was performed on cells from both strains harvested at points along the growth curve. At 48 hours, at the onset of stationary phase, the greatest difference to the expression profile between *M*. *smegmatis mc*^*2*^*155 ΔphoH2* and *M*. *smegmatis mc*^*2*^*155* was observed. The majority of genes upregulated in *ΔphoH2* compared to wild type were those belonging to the SigF regulon. This suggested dis-regulation of SigF and its associated genes in the absence of PhoH2. To investigate the involvement of PhoH2 in the regulation SigF and its regulatory cascade, biologically relevant RNA transcripts were used as targets in activity assays with PhoH2. PhoH2 showed activity toward *sigF* mRNA and we identified multiple copies of the preferred target site of PhoH2 within loops of the predicted *sigF* mRNA structure. This suggested that PhoH2 assembles and initiates its activity on specific single-stranded loops of mRNA. This work presents the first evidence for post-transcriptional regulation of SigF along with a biological function of PhoH2 from *M*. *smegmatis* and predicted mechanism for PhoH2 activity.

## Materials and methods

### Construction of *ΔphoH2* knockout in *M*. *smegmatis mc*^*2*^*155*

An unmarked deletion of *phoH2* was created by a two-step allelic exchange mutagenesis [18]. For this purpose a construct containing 822 bp regions flanking the *phoH2* gene on the left and right respectively (using primers listed in S1 Table), was cloned into pX33 to yield pX33-*phoH2* LFRF. This plasmid was transformed into *M*. *smegmatis mc*^*2*^*155* and transformants were selected at 28°C in the presence of 5 mg/ml gentamycin. For deletion of *phoH2*, strains carrying pX33-*phoH2* LFRF were grown in the presence of gentamycin at 42°C to select for integration of the plasmid into the chromosome of *M*. *smegmatis mc*^*2*^*155* via a single crossover event. Colonies were screened for integration by exposure to 250 mM catechol. Selected colonies were grown in LBT medium at 37°C and aliquots of these cultures were plated onto low salt (2 g/l NaCl) LBT plates containing 10% sucrose and incubated at 42°C to select for a second crossover event leading to the loss of the plasmid and deletion of *phoH2*. Colonies were screened for loss of the plasmid with 250mM catechol and candidate mutants were screened by PCR using primers that flanked the deletion site.

### Growth of *M*. *smegmatis mc*^*2*^*155* and *mc*^*2*^*155 ΔphoH2* in rich, defined and nutrient limiting conditions

*M*. *smegmatis* strains *mc*^*2*^*155* and *mc*^*2*^*155 ΔphoH2* were grown in LB media containing a final concentration of 0.05% tyloxapol. Three overnight starter cultures in LB media grown overnight at 37°C 200 rpm were used to seed three cultures at a starting OD_600_ of 0.01. For defined and nutrient limiting experiments, the overnight LB starter cultures were used to seed a second defined starter culture (Modified Sautons—0.5g/L MgSO_4_.7H_2_0, 2 g/L citric acid, 1g/L L-asparagine, 0.3 g/L KCl.H_2_0, 0.2% glycerol, 0.64 g/L FeCl_3_, 100 μM NH_4_Cl and 0.7 g/L K_2_HPO_4_.3H_2_0). The second starters were incubated overnight at 37°C 200 rpm. These were used to seed three cultures of defined and/or nutrient limiting media, at a starting OD_600_ of 0.002. For nutrient limiting cultures, the carbon, nitrogen or phosphate source was reduced to 0.05%, 0.05 g/L and 40 μM respectively. Cultures were incubated for up to 120 hours (5 days) and growth was monitored by optical density (OD_600_) at regular intervals and curves plotted and analysed for significance using an unpaired t-test (p = 0.05) using Prism V7.

The growth rate (G) of each culture was calculated using OD_600_ absorbance measurements from two time points, B and b, in exponential phase (B–absorbance measurement taken at the beginning and b–absorbance measurement taken at the end of exponential phase). These measurements were used to calculate n (number of generations) and G (generation time) using the following equations: G = t/n (t–time in hours), b assumed to equal B x 2^n^ (expression of growth by binary fission) therefore n = (logb–logB)/log2) and G = t/(3.3 logb/B).

### RNA isolation and sample preparation for RNAseq

Cells from each of the three cultures were harvested for RNA isolation at 24, 48 and 72 hours (at OD_600_ 0.932, 0.959, 0.945 and 0.926, 0.936, 0.934 for *mc*^*2*^*155* and *ΔphoH2* respectively at 24 hours; 2.81, 2.65, 2.43 and 2.73, 2.63, 2.6 for *mc*^*2*^*155* and *ΔphoH2* respectively at 48 hours; and 2.66, 2.53, 2.58 and 2.57, 2.41, 2.51 for *mc*^*2*^*155* and *ΔphoH2* respectively at 72 hours). These were immediately combined with 5 M GITC at a 1:4 ratio of cells to GITC. These were spun down and resuspended in 0.5 ml 5 M GITC and stored in a tube containing approximately 0.3 g 0.1 mm and 2.5 mm zirconia beads. Cells were ruptured using a Fast Prep cell disrupter (FP120 Thermo Savant) for increasing time periods. *The cells were incubated at RT with 50 μl 2 M sodium acetate pH 4 for 10 minutes, before incubation on ice with 100 μl 1-bromo, 3-chloro propionate for 5 minutes. Samples were spun to separate the phases and the top layer collected and the process repeated twice from *. The final top layer was incubated at -40°C overnight with an equal volume of isopropanol. Samples were spun at 13000 rpm for 15 minutes at 4°C. The precipitated RNA was dissolved in 100 μl 10 mM Tris-HCl pH 7, 0.5 mM MnCl_2_ and DNAse treated with 2.5 μl Promega DNAse for 30 minutes at 37°C. The samples were incubated in a solution of 5.2 M guanidium thiocyanate, 2 M guanidine HCl and 2 M urea for 5 minutes at RT, prior to incubation for 10 minutes at RT with 400 μl isopropanol. Samples were spun at 13000 rpm for 15 minutes at RT, the pellet was washed with 70% ethanol then dissolved in 200 μl of RNAse free H_2_0. To this, 200 μl of 5 M LiCl_2_ was added and incubated for 1 hour at -20°C. Samples were spun at 13000 rpm for 15 minutes at 4°C and the pellet resuspended in 100 μl RNAse free H_2_O. The RNA was precipitated with 10 μl 3 M sodium acetate pH 5.2 and 275 μl 100% ethanol at -20°C for 10 minutes. Samples were spun at 13000 rpm for 15 minutes at 4°C. The pellet was washed with 70% ethanol and spun at 13000 rpm for 15 minutes at 4°C. The final RNA pellet was resuspended in 25 μl 10 mM Tris-HCl pH 7, 0.5 mM MnCl_2_. The ‘best’ RNA samples as determined by A260/A280 and A260/A230 ratios and gel analysis from each strain/time point were pooled and sent for sequencing in 75% ethanol.

### Transcriptome analysis

The transcriptome of each RNA sample was sequenced at the Beijing Genomics Institute (BGI), China. RNA samples that met library construction requirements (RIN >0.8) had their rRNA removed and were fragmented for cDNA synthesis for sequencing on an IlluminaHiSeq2000/2500. Raw reads were filtered and the clean reads aligned with the genome of *M*. *smegmatis mc*^*2*^*155* (NC008596.1) using SOAP aligner/SOAP2. The alignment was used to calculate the distribution of reads and coverage. An initial table of differentially expressed genes between *M*. *smegmatis mc*^*2*^*155* and *M*. *smegmatis mc*^*2*^*155 ΔphoH2* was compiled that had a FDR ≤ 0.001 and an absolute value of Log2 ratio of ≥1 (S2 File). These genes were further manually curated and shortlisted based on the following criteria ≥2 Log2 ratio and ≥75 reads (S2 File).

### qRT-PCR

RNA was isolated as described for RNAseq from three cultures of *M*. *smegmatis mc*^*2*^*155* and *M*. *smegmatis mc*^*2*^*155 ΔphoH2* grown in LB media. cDNA was prepared from each RNA sample using qScript™ XLT (Quanta Biosciences) as per the manufacturer’s guidelines. Each cDNA sample was used as template in triplicate qPCR reactions with primers MSMEG_1773 Fwd and Rev, MSMEG_2758 Fwd and Rev, MSMEG_2415 Fwd and Rev, and 16s Fwd and Rev (S1 Table), HotFirePol DNA polymerase (Solis BioDyne) and SYTO82 (ThermoFisher). Real time PCR was performed using a MIC qPCR cycler (Bio Molecular Systems). Cq values were generated using LinReg within the MIC software. These Cq values were used to determine gene expression values using Livak’s formula [19] and p values were derived by t-test using Prism (V8.4). Fold change was calculated by taking log2 of the expression ratio.

### Protein expression and purification

PhoH2 and PhoH2 -R339A were expressed and purified as described in Andrews & Arcus (2015) [12]. Briefly, a single colony was used to inoculate an LB seeder culture supplemented with 50 mg/ml kanamycin. This culture was grown for 24 h at 37°C and was used at a 1:100 dilution to inoculate an LB expression culture supplemented with 50 mg/ml kanamycin. These cultures were incubated at 37°C and were induced with a final concentration of 0.75 mM IPTG at an OD_600_ of 0.4-0.6 and further incubated with shaking at 37°C overnight. Cells from large-scale expression cultures were harvested. For purification, cells were resuspended in lysis buffer, 50 mM TRIS pH 8, 200 mM NaCl, 5 mM MgCl_2_, sonicated on ice and harvested by centrifugation. The soluble fractions containing His-tagged PhoH2 or PhoH2-R339A were purified by IMAC on a HisTrap HP column (GE Healthcare, UK). The protein fractions were purified further by size exclusion chromatography, using an S200 10/300 Superdex™ column (GE Healthcare, UK) in the same buffer.

### Biological target assays

The DNA sequence of *sigF* and *rsbW-sigF* were amplified from *M*. *smegmatis mc*^*2*^*155* genomic DNA using SF Fwd and Rev and RS Fwd and SF Rev, respectively (S1 Table) in PCR reactions with either KAPA HiFi DNA polymerase with high GC buffers (KAPA Biosystems) or Hot FIREPol Blend Master mix (Solis BioDyne). These PCR products were used as DNA template for a second round of PCR using T7+SF Fwd and T7+RS Fwd that include the minimum promoter sequence necessary for efficient transcription as described in the preparation of DNA template of the MEGAscript T7 transcription kit (ThermoScientific) in place of the original forward primer to introduce the T7 promoter sequence to the 5’ end of the PCR product. The resulting +T7 PCR products were transcribed into RNA as per the MEGAscript T7 transcription kit. Purified PhoH2 or PhoH2-R339A (125 μM) was incubated with target RNA (25 μM) in a reaction containing 5 mM ATP made up to 15 μl with assay buffer (50 mM tris pH 7.5 20 mM NaCl 5 mM MgCl_2_) for 5, 15 and 30 minutes at 37°C (30 minutes only for PhoH2-R339A). Reactions were quenched with and equal volume of 2 x formamide stop solution (80% formamide (v/v), 5 mM EDTA, 0.1% (w/v) bromophenol blue). Prior to analysis by electrophoresis on a 1x TAE 1.5% gel, reactions were heated at 70°C for 5 min and cooled immediately on ice. The results were visualised by staining with 1x SYBR safe nucleic acid stain (Invitrogen).

## Results

### *M*. *smegmatis mc*^*2*^*155 ΔphoH2* adopts the same growth profile as *M*. *smegmatis mc*^*2*^*155* in rich, defined and nutrient limiting growth conditions

**Fig 2:**
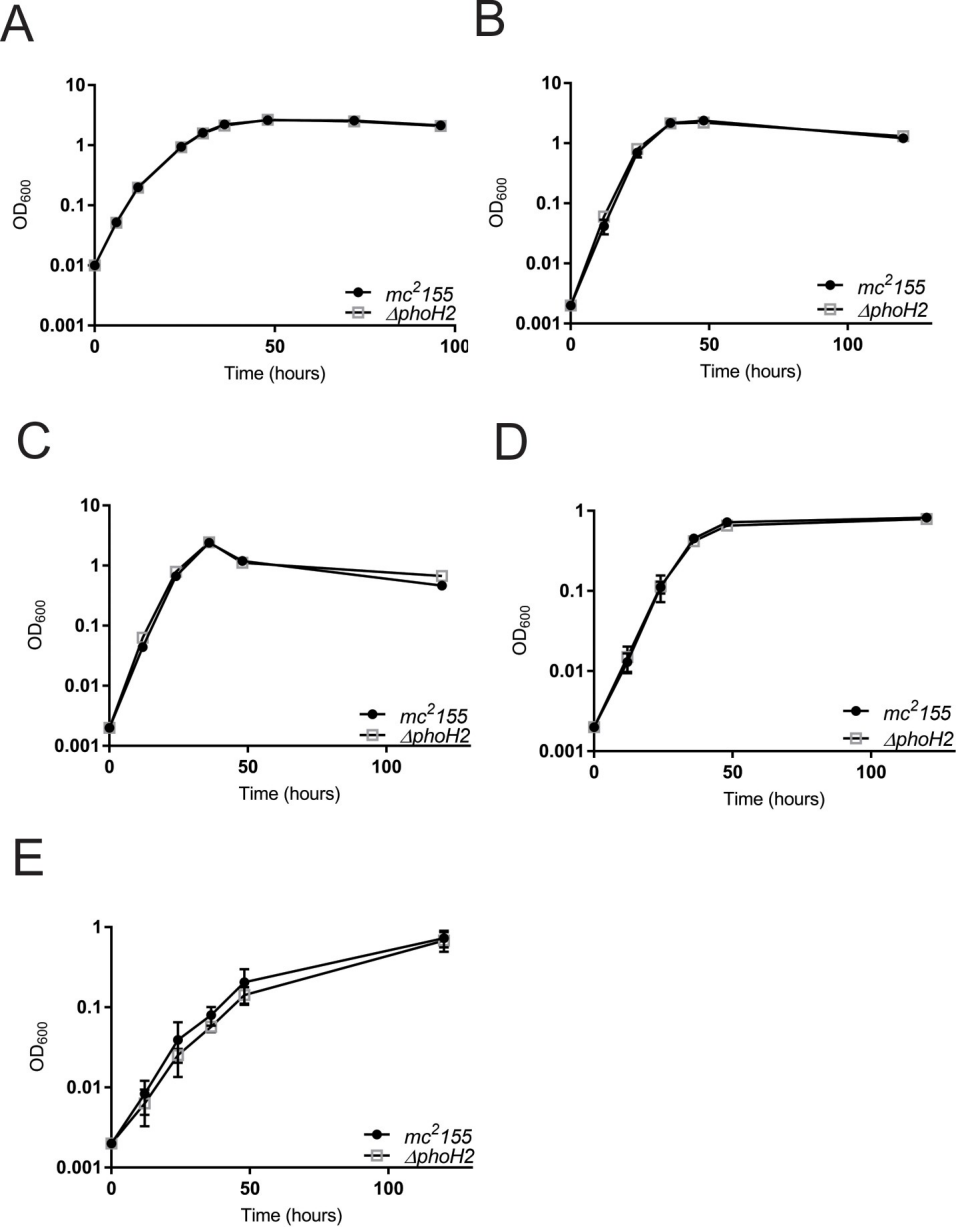
*M*. *smegmatis mc*^*2*^*155 ΔphoH2* adopts the same growth profile as *M*. *smegmatis mc*^*2*^*155* in rich, defined and nutrient limiting growth conditions. *M*. *smegmatis mc*^*2*^*155* (closed circles) and *M*. *smegmatis mc*^*2*^*155 ΔphoH2* (open squares) were cultured in (A) LB, (B) Sautons, (C) Sautons—carbon limiting, (D) Sautons—nitrogen limiting and (E) Sautons—phosphate limiting media. Growth was measured by monitoring optical density (OD_600_) at regular intervals. Data were plotted as the mean and SD of three biological replicates of each strain and an unpaired t-test was used to test for significant differences between the strains. Plots show that *M*. *smegmatis mc*^*2*^*155 ΔphoH2* adopts the same growth profile as its parent in rich, defined and nutrient poor conditions. (Plots generated and analysed in Prism V7).

To screen for differences in the growth profile of *M*. *smegmatis mc*^*2*^*155 ΔphoH2* (S1 Fig) compared with its parent, *M*. *smegmatis mc*^*2*^*155*, strains of *M*. *smegmatis* were grown in standard rich, defined and nutrient limiting conditions and growth measured by optical density (OD_600_). Fig 2 shows that *M*. *smegmatis mc*^*2*^*155 ΔphoH2* adopts the same growth profile as its parent in standard rich, defined and nutrient limiting conditions and that the growth rates also match (Table 1).

**Table 1:** Growth rates of *M*. *smegmatis mc*^*2*^*155* and *M*. *smegmatis mc*^*2*^*155 ΔphoH2* in rich, defined and limiting growth media.

Growth media	G (hours)	G (hours)
	*M*. *smegmatis*	*M*. *smegmatis*
		*mc*^*2*^*155 ΔphoH2*
	*mc*^*2*^*155*	
**LB—rich**	2.80 ± 0.07	2.80 ± 0.04
**Sautons—defined**	3.95 ± 0.14	3.82 ± 0.01
**Sautons—carbon limiting**	3.99 ± 0.04	3.84 ± 0.08
**Sautons—nitrogen limiting**	6.99 ± 0.44	7.08 ± 0.81
**Sautons—phosphate limiting**	11.61 ± 1.70	12.76 ± 0.92

Growth rate is presented in hours as the mean and ± SD of three cultures of each strain.

Both strains reached higher optical densities when growth in rich, defined and carbon limiting media than when grown in limiting nitrogen or phosphate media, indicating stunted growth when these nutrients are deficient. Growth was approximately 3x slower in phosphate limiting conditions compared with when phosphate was sufficient, further suggesting growth limitations when phosphate is reduced.

### RNAseq reveals an upregulation of SigF regulon genes in *M*. *smegmatis mc*^*2*^*155 ΔphoH2* at 48 hours

Due to the comparable growth profiles between *M*. *smegmatis mc*^*2*^*155* and *M*. *smegmatis mc*^*2*^*155 ΔphoH2* and identical growth rate in rich media, we used RNAseq to provide a snapshot of the differences between the transcriptomes of *M*. *smegmatis mc*^*2*^*155* and *M*. *smegmatis mc*^*2*^*155 ΔphoH2*. Cells of each strain were harvested after 24, 48, and 72 hours of growth and RNA was isolated from cells harvested from each of the three cultures and the ‘best’ RNA, as determined by A260/A280 and A260/A230 ratios and gel analysis were pooled and stored in 75% ethanol. The transcriptome sequencing and downstream analysis for differentially expressed genes (DEGs), were performed at the Beijing Genomics Institute (BGI). RNA harvested from cells upon entry into stationary phase (48 hours of growth) revealed the greatest change in gene expression between *M*. *smegmatis mc*^*2*^*155 ΔphoH2* and *M*. *smegmatis mc*^*2*^*155* (S2 File). These genes were curated to include those that had ≥2 Log2 ratio and ≥75 reads and in *M*. *smegmatis mc*^*2*^*155 ΔphoH2*, 87 genes were upregulated and 1 downregulated compared with *M*. *smegmatis mc*^*2*^*155* (S2 File).

Of these 87 upregulated genes, 78 belonged to the SigF regulon [6, 20] accounting for 70% of known SigF regulated genes exclusive to stationary phase [6] and 90% of all the upregulated genes identified in this study. The single downregulated gene (MSMEG_0586), is reported as a predicted STAS-domain containing anti-sigma factor antagonist [6]. The further 9 genes that were upregulated in this study, that were not part of the SigF regulon, encode for hypothetical proteins, an antigen 85-C protein and a cluster of genes (MSMEG_1974–1979). Real time PCR was used to confirm the trends observed with RNAseq (S2 Fig).

### PhoH2 is involved with regulation of the SigF regulon through its activity on *sigF* RNA

**Fig 3:**
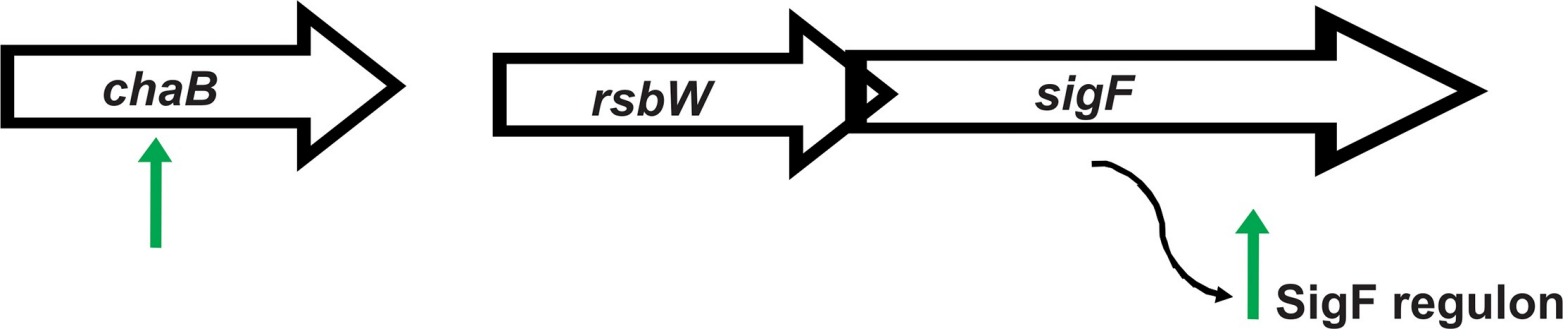
Schematic outlining changes to the expression of *chaB* and the SigF regulon genes observed in *M*. *smegmatis mc*^*2*^*155 ΔphoH2* compared with *M*. *smegmatis mc*^*2*^*155*. Green arrows indicate the changes in expression of genes in *M*. *smegmatis mc*^*2*^*155 ΔphoH2* relative to *M*. *smegmatis mc*^*2*^*155* at 48 hours of growth. The expression of *sigF* transcript gene *chaB* and SigF regulon genes increased in the absence of PhoH2.

With *M*. *smegmatis mc*^*2*^*155 ΔphoH2* we observed an increase in the expression profiles of predominantly SigF regulon genes (Fig 3 and S2 File).

During normal growth, constitutively expressed SigF is post-translationally regulated by its co-expressed cognate anti-sigma factor, RsbW, that sequesters the activity of SigF, preventing its interaction with RNAP. Upon entry into stationary phase, heat or oxidative stress, anti-sigma factor antagonists lift the repression caused by RsbW permitting SigF to bind to RNAP and direct transcription of SigF regulon genes. We predict that upon relief of stress, the expression of anti-sigma factor antagonists decreases enabling RsbW to bind to SigF, preventing its activity, until further stress is encountered. This suggests tightknit regulation of SigF and fine-tuning of the regulon.

In light of the dis-regulation of the expression of SigF regulon genes in the absence of PhoH2, that are under the control of SigF, we hypothesised that PhoH2 targets *sigF* mRNA directly, by way of its mRNAse -helicase activity to fine-tune transcript levels, overall contributing to the finely tuned regulation of SigF and its regulon.

**Fig 4:**
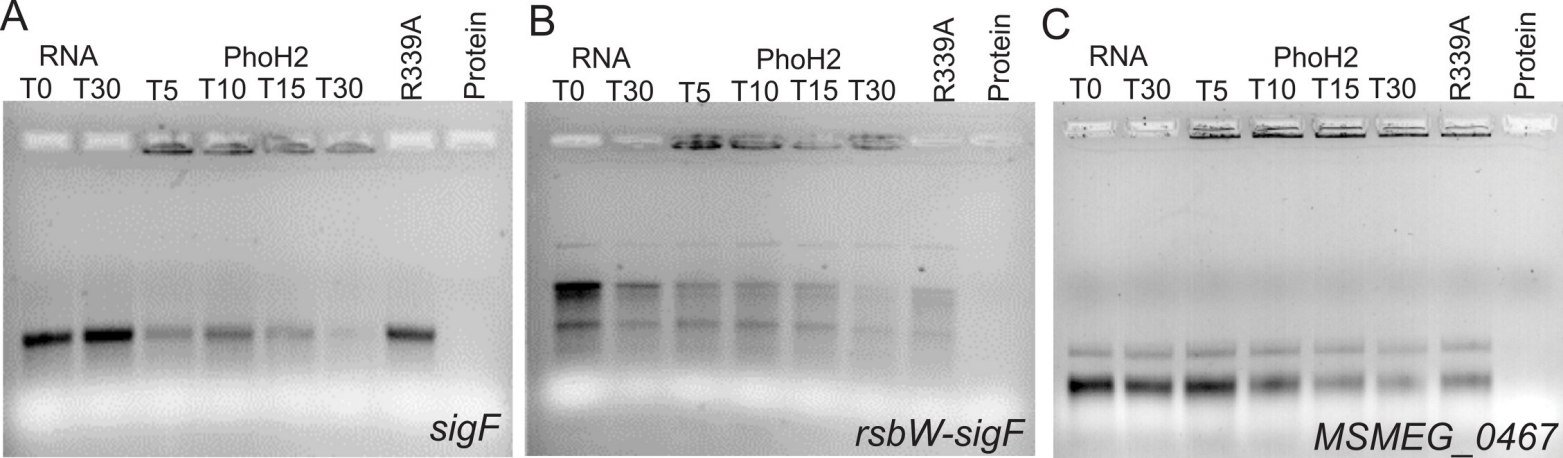
PhoH2 acts on *sigF* RNA. (A) *sigF*, (B) *rsbW-sigF* and (C) *MSMEG_0467* RNA transcripts were assayed with PhoH2 over time. RNA unwinding and degradation by PhoH2 of *sigF* RNA is shown by the decrease in RNA intensity with an increase in smearing of the RNA to elimination over time in the presence of PhoH2 compared with RNA only. RNA binding by PhoH2 is evident by retardation of the RNA in the wells. No activity was observed by PhoH2-R339A (RNA helicase mutant) [10] when used in place of PhoH2. (B) *rsbW-sigF* RNA shows intrinsic decay as shown by the loss in intensity of the RNA only control at T30. At the same time point (T30), in the presence of PhoH2, there is a greater loss of integrity with increased smearing suggesting degradation by PhoH2 of this RNA. In the presence of R339A there is some aspecific degradation. (C) There was no loss of the integrity of MSMEG_0467 RNA as evidenced by the retention of RNA integrity over the time course. Reactions/labels: RNA only at T_0_ and T_30_—RNA (25 μM) incubated in reactions containing 5 mM ATP made up to 15 μl with assay buffer (50 mM tris pH 7.5 20 mM NaCl 5 mM MgCl_2_) at 37°C. T_5_, T_15_, T_30_—RNA + PhoH2 (125 μM)—incubated in reactions as above for 5, 15 or 30 minutes. R339A - RNA + PhoH2-R339A in place of PhoH2. Protein—protein only. Reactions were analysed by 1.5% TAE electrophoresis.

To test this, two *sigF* mRNA transcripts; *rsbW-sigF* and *sigF* were amplified from *M*. *smegmatis mc*^*2*^*155* genomic DNA and transcribed into RNA using MEGAscript T7 transcription kit. These were presented as targets, for unwinding and degradation by PhoH2, in reactions over a time-course. RNA transcribed from MSMEG_0467 was used to confirm the preferred activity of PhoH2 for *sigF* transcripts. This gene did not show changes in its expression profile between *M*. *smegmatis mc*^*2*^*155* and *M*. *smegmatis mc*^*2*^*155 ΔphoH2* at 48 hours and was of similar length (740 bases) and GC content (64.4%) to the other RNA sequence. Fig 4 shows the unwinding and degradation of *sigF* RNA (Fig 4A) as shown by the decrease in RNA intensity combined with an increase in smearing of the RNA to elimination over time in the presence of PhoH2. This is the same mode of activity observed for PhoH2 by Andrews & Arcus (2015) on its preferred *in vitro* targets. Like, on *in vitro* targets, PhoH2 is also binding to the RNA, evident by the retardation of RNA in the wells, most intensively at earlier time points of the time course (Fig 4A). Activity was also observed on *rsbW-sigF* RNA (Fig 4B). The RNA in this assay is showing intrinsic decay as evidenced by the loss in intensity of the RNA only control at T30. At the same time point (T30), in the presence of PhoH2 there is a greater loss of integrity with increased smearing suggesting degradation by PhoH2 of this RNA. Binding of the RNA was also observed with this substrate. In this assay we also observe aspecific degradation by the PhoH2-R339A mutant. There was no loss of the integrity of MSMEG_0467 RNA over the time course (Fig 4C) and no abolition of the RNA as was observed by PhoH2 on *sigF or rsbW-sigF*. RNA binding was apparent by both PhoH2 and PhoH2-R339A with each RNA suggesting that PhoH2 binds RNA in a non-specific manner. Collectively, these observations suggest that PhoH2 acts on *sigF* RNA.

## Discussion

This is the first report experimentally validating a biological target of PhoH2. Three studies report on the possible function of PhoH2 [15–17]. One on PhoH2s involvement in regulating the infection process of *Synechococcus sp*. *strain WH8102* by Cyanomyovirus [15]. Another on PhoH2s involvement in the response of *C*. *glutamicum* to limiting phosphate [16] and evidence for a decrease in the expression of *phoH2* during transition from exponential growth to stationary phase growth of *C*. *glutamicum* [17]. This work suggested that this phase of growth is modulated by SigB [17] and infers that *phoH2* expression is not under the control of this sigma factor.

**Fig 5:**
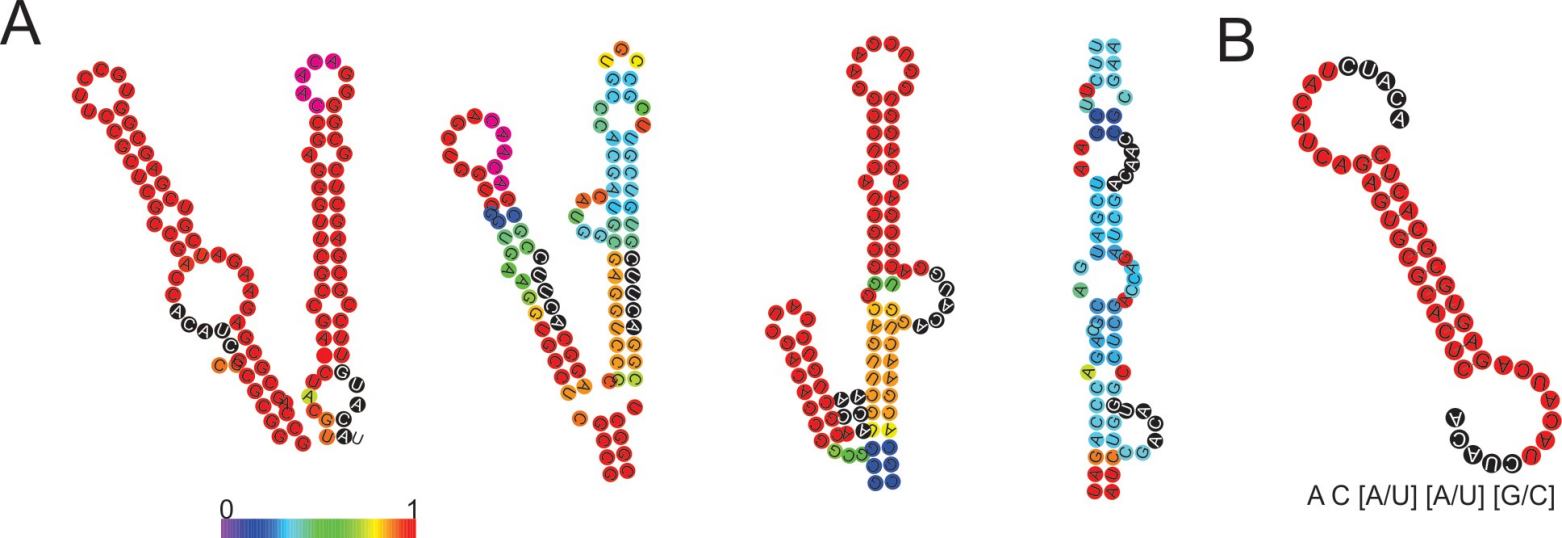
Preferred target sites identified in *sigF* are positioned within stems and loops. (A) Four major stem-loop secondary structures taken from the predicted secondary structure of *sigF* RNA and (B) predicted secondary structure of 5’5 RNA and the preferred target sequence [11]. The stem loops are coloured by base pair probability and the preferred target sequences are highlighted with black circles and white text. RNA secondary structure was modelled by RNAfold [21].

PhoH2 has sequence-specific RNA and ATP-dependent activity, with preference for double-stranded RNA with 5’- 3’ overhangs, and the terminal combination of RNA bases 5’- A C [A/U] [A/U] [G/C] [12]. The RNA tested here contain versions of this preferred sequence and the greatest number identified in the *sigF* transcript (Table 2 and Fig 5).

**Table 2:** Preferred target sequences and their occurrence in the RNA transcripts.

Sequence	*rsbW*	*sigF*	*MSMEG_0467*
**ACAAG**			
**ACAAC**		4	1
**ACAUG**		3	
**ACAUC**		1	
**ACUAG**			
**ACUUG**		1	1
**ACUUC**	1	2	
**ACUAC**			
**Total**	1	11	2

The locations of these target sites are distributed throughout *sigF* mRNA most frequently occurring in loops (9 of the 11 sites) (Fig 5). The number, location and position of these target sites most commonly occurring in the *sigF* transcript suggest that of the targets tested here, that *sigF* is a primary target of PhoH2.

This observation further compliments the *in vitro* biochemical activity of PhoH2 that is specific to single-stranded RNA [12] and suggests that *in vivo*, the PhoH2 hexamer assembles on RNA loops. The mechanism of substrate binding and translocation has been determined for the related hexameric RNA helicase Rho [22]. Rho encircles single-strands of RNA and tethers RNA via its Q and R loops [22]. These loops are responsible for interactions with incoming substrate and project a spiral staircase into the central pore of the hexamer [22, 23]. The Q and R loop staircase recognises and tracks the phosphodiester backbone of RNA, and in conjunction with sequential firing of the asymmetric subunits of the helicase ring, that are in different ATP binding states (nucleotide exchange, ATP-bound, hydrolysis competent and product state), together pull the phosphates and sugars through the ring [22, 23].

**Fig 6:**
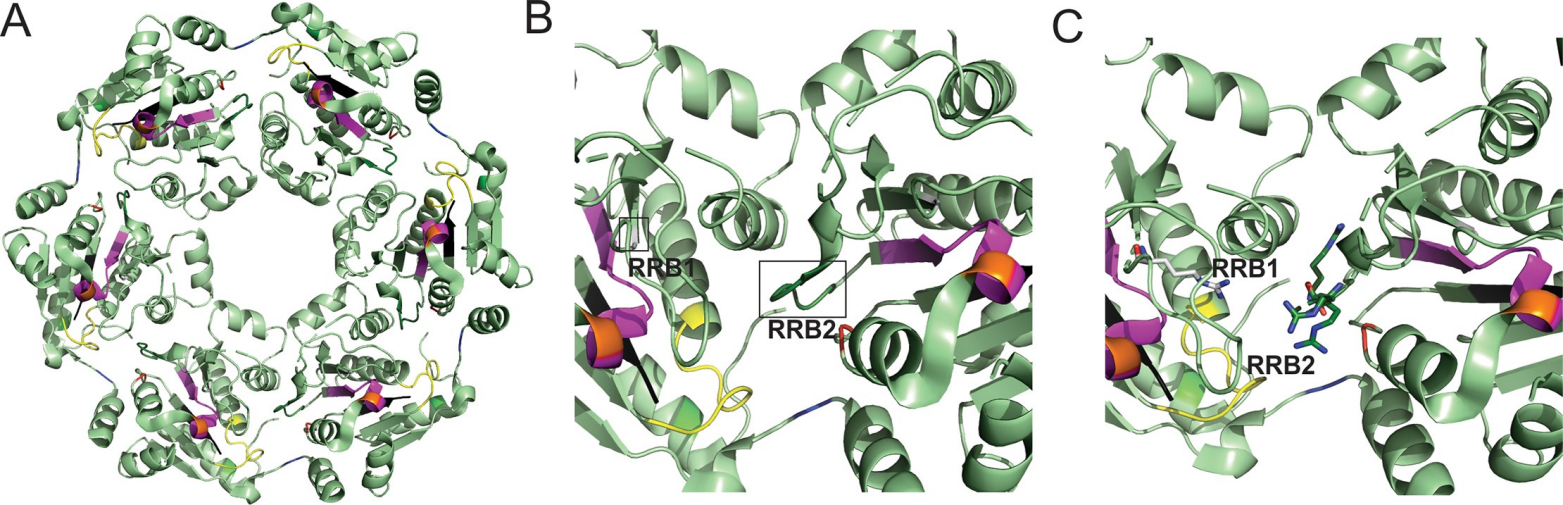
Hexameric structure and active site of the PhoH RNA helicase. Hexameric structure of the PhoH domain of PhoH2 (A) showing the central pore and position of conserved motifs: Q motif (green), Walker A (yellow), RNA Recognition and Binding 1 (RRB1) (light grey), RRB2 (dark green), Walker B (magenta), Sensor I (orange), Second Region of Homology (red), Motif III (black) and Sensor II (blue) [12]. (B) Magnified view of the PhoH active site that forms between two adjacent monomers. The positions of RRB1 and 2 are highlighted (boxed) and located on loops. (C) Magnified view of PhoH2 active site showing the position of highly conserved arginine residues (shown as sticks) critical for RNA recognition and binding. Images were made in PyMol V2X using PDB 3B85.

Evidence thus far suggests that PhoH2 adopts a hexameric quaternary structure [24]. The active site forming between neighbouring subunits, positioning the nucleic acid binding motifs (RRB1 and RRB2) adjacent to one another on loops near the central pore (Fig 6). The reported inherent flexibility of hexameric helicase subunits [22] may enable these motifs to project into the pore, reminiscent of the Q and R loops of Rho, facilitating RNA recognition and threading into the pore. The unwound RNA may then be fed into and degraded by the PIN-domain RNAse ring. This mechanism implies general RNAse activity of the PIN-domain of PhoH2 and may explain the conservation of PhoAT, the small protein expressed with PhoH2, that may function to sequester RNAse activity, reminiscent of VapB of VapBC toxin-antitoxin systems.

We propose that PhoH2, by way of its RNA unwinding and mRNAse activity, contributes, on a post-transcriptional level, to the regulation of SigF by modulating transcript levels. We predict that during growth, constitutively expressed *rsbW-sigF* leads to a pool of RsbW-SigF in the cell. Upon entry into stationary phase there is an increase in transcription from the promoter site located upstream of *chaB* [10] resulting in increased expression of *chaB-rsbW-sigF* mRNA and so a greater pool of *sigF*. Depending on the physiological change, we expect increased expression of anti-sigma factor antagonist mRNA in order to lift the repression of RsbW, permitting SigF to bind to RNAP and initiate transcription of its regulon as well as increasing the expression of its own mRNA transcript [10]. Upon established stationary phase and/or alleviation of stress, the expression of anti-sigma factor antagonists is decreased, permitting RsbW to remain bound to SigF, sequestering its activity. We propose that PhoH2 functions to moderate the pool of *sigF* mRNA, during this process and so modulating the SigF response. With controlled levels of *sigF* mRNA and SigF in the cells, this allows for fine control of the cells response to changing physiological conditions.

These results add further complexity and provide the first report of a post-transcriptional mechanism of regulation of SigF in mycobacteria. This mechanism likely operates in conjunction with the post-translational mechanisms of regulation to enable tightknit control of both SigF and its regulon of genes through the transition from exponential to stationary phase of growth and under changing environmental conditions.

## Supporting information

S1 Raw Images (PDF)

S1 Fig PCR confirmation of *phoH2* deletion from *M. smegmatis mc^2^155*. PCR using primers that flanked the deletion site were used with *M*. *smegmatis mc*^*2*^*155* or mc^2^155 *ΔphoH2* DNA as template to confirm the deletion of *phoH2*. Expected band sizes: *mc*^*2*^*155*–2914 bp and *mc*^*2*^*155 ΔphoH2–*1622 bp.
(TIF)

S2 Fig qRT-PCR validation of RNAseq data. qRT-PCR was used to validate the expression profile of three genes that showed a change, either up or down, in their expression in *M*. *smegmatis mc*^*2*^*155 ΔphoH2* compared with *M*. *smegmatis mc*^*2*^*155* with RNAseq. qRT-PCR confirmed that these genes followed the same direction of change in their expression as suggested by RNAseq. (A) Plot of log2 fold ratio of genes MSMEG_1773, MSMEG_2415 and MSMEG_2758 determined using qRT-PCR. (B) table comparing log2 ratio change between RNAseq and qRT-PCR.
(TIFF)

S1 Table Primers used in this study. (DOCX)

S1 File Biological target RNA sequences. (DOCX)

S2 File Summary of sequencing and alignment statistics and list of Differentially Expressed Genes (DEGS). (DOCX)
