# Supplementary figures and images for: Post-transcriptional modulation of the SigF regulon in Mycobacterium smegmatis by the PhoH2 toxin-antitoxin
Source: PLoS One. 2020 Jul 29;15(7):e0236551. doi: 10.1371/journal.pone.0236551 (PMC7390352; doi:10.1371/journal.pone.0236551)

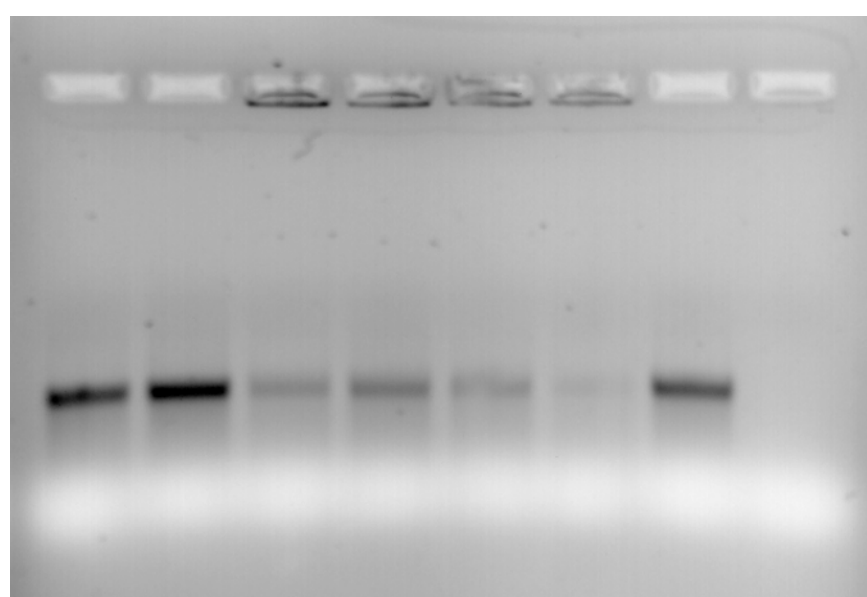

sigF

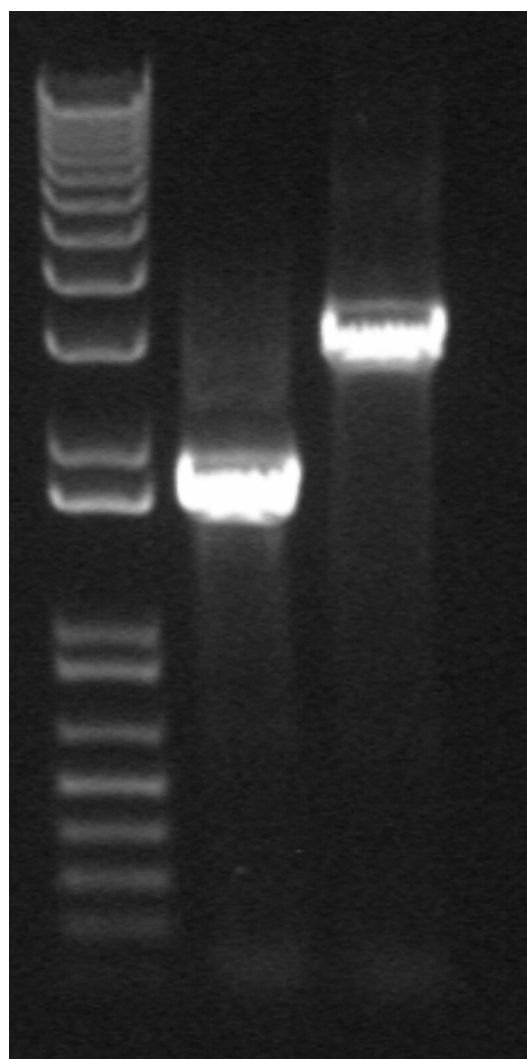

FigS1

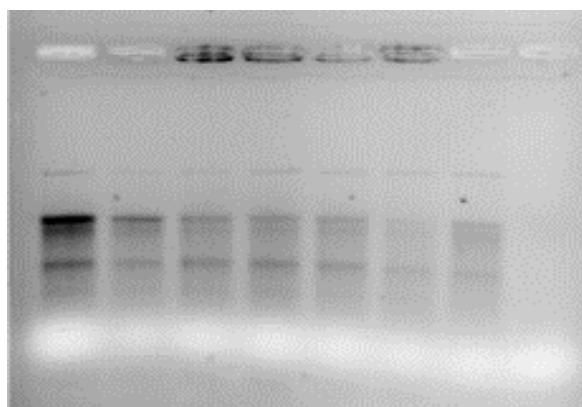

rsbW-sigF

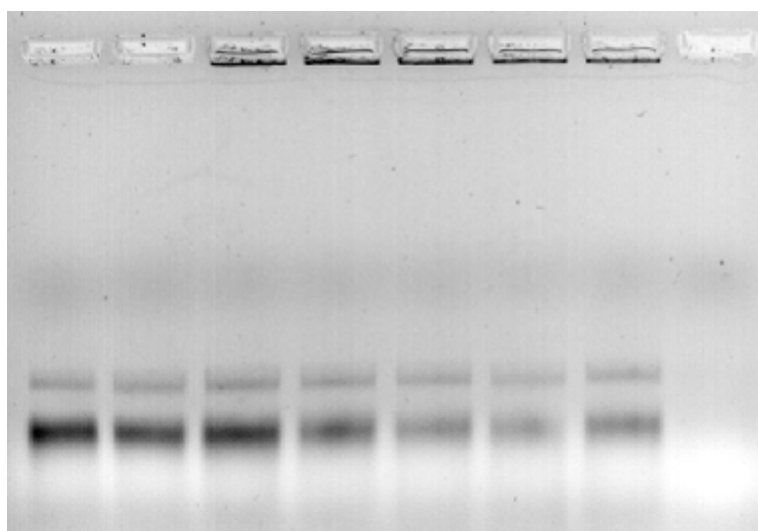

0467

Supplement: S1 Raw Images — (PDF) [file pone.0236551.s001.pdf]

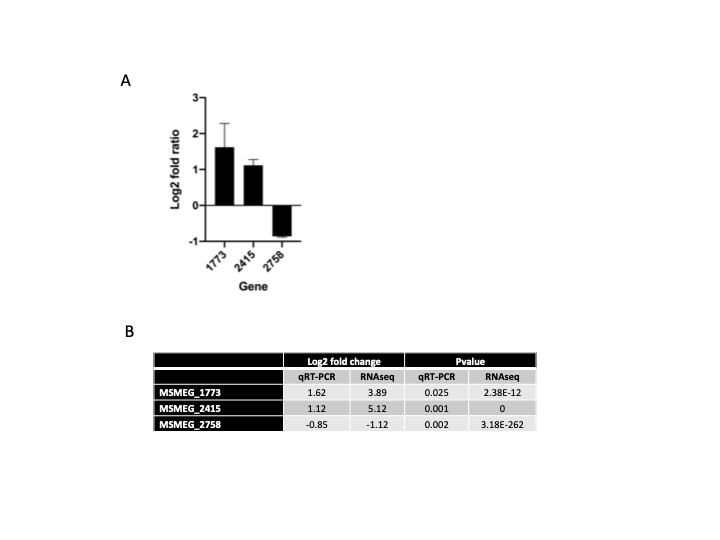

Supplement: S2 Fig — qRT-PCR was used to validate the expression profile of three genes that showed a change, either up or down, in their expression in M. smegmatis mc2155 ΔphoH2 compared with M. smegmatis mc2155 with RNAseq. qRT-PCR confirmed that these genes followed the same direction of change in their expression as suggested by RNAseq. (A) Plot of log2 fold ratio of genes MSMEG_1773, MSMEG_2415 and MSMEG_2758 determined using qRT-PCR. (B) table comparing log2 ratio change between RNAseq and qRT-PCR. (TIFF) [file pone.0236551.s003.tiff]
