# Supplementary material for: Post-transcriptional modulation of the SigF regulon in Mycobacterium smegmatis by the PhoH2 toxin-antitoxin
Source: PLoS One. 2020 Jul 29;15(7):e0236551. doi: 10.1371/journal.pone.0236551 (PMC7390352; doi:10.1371/journal.pone.0236551)
Supplement: S1 Table — (DOCX) [file pone.0236551.s004.docx]

| Primer name | Sequence 5’ to 3’ |
| --- | --- |
| phoH2 LF Fwd | CTAGTCTAGACGTCGCGCTGCGCAACTACCTGGTGGT |
| phoH2 LF Rev | TACGGACAGCTTGCTCAGTCAC |
| phoH2 LF olap Rev | AGGGCGCCGGGGCTGTACGGACAGCTTGCT |
| phoH2 RF Fwd | CAGCCCCGGCGCCCTGCCCTGA |
| phoH2 RF olap Fwd | AGCAAGCTGTCCGTACAGCCCCGGCGCCCT |
| phoH2 RF Rev | GGACTAGTCGCCGGTCGCCTGCTGCTGTGCGGGGCGACCGGGA |
| LF seq Fwd | CGTCGCGCTGCGCAA |
| RF seq Rev | CGCCGGTCGCCTGCT |
| MSMEG_1773 Fwd | TGATCTCCGAGATCAAAGGC |
| MSMEG_1773 Rev | CGGTAGTGCAGTTCGTAGCA |
| MSMEG_2758 Fwd | CGTTCCTCAACCTCATCCAG |
| MSMEG_2758 Rev | GATCACCTGGACCATCTGCA |
| MSMEG_2415 Fwd | TCGAAGAGGAACACAAGGCC |
| MSMEG_2415 Rev | CGGGAGAGCTTGACGAACTC |
| MSMEG_16s Fwd | CAGCTCGTGTCGTGAGATGT |
| MSMEG_16s Rev | AGACCGGCTTTGAAAGGATT |
| SF Fwd | GTGACGTCGGAATACGCAGA |
| SF Rev | GCTACTGCAGCTGGTCGCGC |
| T7 + SF Fwd | *TAATACGACTCACTATAGG*GTGACGTCGGAATACGCAGA |
| RS Fwd | GTGGCGGAACACCCGCTCGG |
| T7 + RS Fwd | *TAATACGACTCACTATAGG*GTGGCGGAACACCCGCTCGG |
| U0467 Fwd | CCGGCGAAGAACCGACGTGACACTC |
| 0467 Rev | TCACGTCAGCATCATGCCGAGGCCG |
| T7+U0467 Fwd | *TAATACGACTCACTATAGG*CCGGCGAAGAACCGACGTGACACTC |

**Table S1: Primers used in this study**

*Italic –* T7 promoter sequence
