# Supplementary material for: Post-transcriptional modulation of the SigF regulon in Mycobacterium smegmatis by the PhoH2 toxin-antitoxin
Source: PLoS One. 2020 Jul 29;15(7):e0236551. doi: 10.1371/journal.pone.0236551 (PMC7390352; doi:10.1371/journal.pone.0236551)
Supplement: S1 File — (DOCX) [file pone.0236551.s005.docx]

**File S1. Target sequences**

**Bold** - Translational start site

Underlined - preferred target

*Italic –* primer position

Green – T7 promoter

*sigF* (753 bp)

*TAATACGACTCACTATAGGGTGACGTCGGAATACGCAGA*CGTTCTCGACATGTTCCGCGAGCTCGCGGGGACAACCGAGGGTTCGCCGAGCTACGTCCGCCAGCGCGAGAAGATCGTCGAGCGGTGCCTTCCGCTCGCCGACCACATCGCGCGGCGCTTCGACGGTCGAGGGGAGCCCCGCGAGGATCTGGTGCAGGTCGCGCGAGTGGGCTTGGTGAACGCGGTGAACCGGTTCGACGTGGAAGCCGGCTCGGACTTCGTGTCGTTCGCCGTGCCCACGATCATGGGCGAGGTCCGCAGGCACTTCCGCGACAACAGCTGGTCGGTGAAGGTGCCTCGCCGGCTCAAGGAACTCCACTTGCGGCTCGGAGCCGCGACCGCCGAGCTCTCGCAGCGCTTGGGCCGGGCGCCCACCGCATCGGAGCTGGCGCACGAACTGGACATGGATCGCGAAGAGGTGGTCGAAGGCCTCATCGCGGGCAGTTCGTACAACACGCTGTCCATCGACAGCGGCGGCGGTGGCGACGAGGAAGCCCCCGCGATCGTCGACACGCTCGGCGATCTCGACATGGGCCTCGACCAGATCGACAACCGCGAATCGCTGCGCCCGCTGCTGGCCAGCCTGCCGGAGCGCGAGCGCACCGTGCTGGTGCTGCGATTCTTCGAATCGATGACGCAGACCCAGATCGCCGAGCGGGTCGGCATCTCGCAGATGCACGTCTCCCGTCTGCTGGCCAAGTCGCTGGCGCGGCTGC*GCGACCAGCTGCAGTAG*

*rsbW + sigF* (1166 bp)

*TAATACGACTCACTATAGG****G****TGGCGGAAACACCCGCTCGG*GGCGAGCGGTCGGTAGAGATCCGCGTCGCCGCGATGTTGGAGAACCTGGCGGTGGTGCGCACCGTGGTCGCTGCGATCGCAACGTTCGAGGACCTCGACTTCGACGTGGTCGCGGATCTGCGGCTGGCGGTCGACGAGGCATGCACCACGTTGATCAGGTCGGCGGTGCCTGACGCGACGCTCGTCCTGCGGGTGGATCCCGGTCCCGATGCCGTGGTTATCTCCACGTCCACGGTGTGTATCGGCGACAATGTCGTCGAGCCCGGCAGTTTCAGCTGGCACGTGCTGAGTTCTCTCACCGACGAGGTGAACACATTCACAGATGGGTCCGGGCCTGAAGAGGGGCAGGTGTTCGGCATCACGATGACCACGAGGCGAGCGAGCCTGCTGCG**G**TGACGTCGGAATACGCAGACGTTCTCGACATGTTCCGCGAGCTCGCGGGGACAACCGAGGGTTCGCCGAGCTACGTCCGCCAGCGCGAGAAGATCGTCGAGCGGTGCCTTCCGCTCGCCGACCACATCGCGCGGCGCTTCGACGGTCGAGGGGAGCCCCGCGAGGATCTGGTGCAGGTCGCGCGAGTGGGCTTGGTGAACGCGGTGAACCGGTTCGACGTGGAAGCCGGCTCGGACTTCGTGTCGTTCGCCGTGCCCACGATCATGGGCGAGGTCCGCAGGCACTTCCGCGACAACAGCTGGTCGGTGAAGGTGCCTCGCCGGCTCAAGGAACTCCACTTGCGGCTCGGAGCCGCGACCGCCGAGCTCTCGCAGCGCTTGGGCCGGGCGCCCACCGCATCGGAGCTGGCGCACGAACTGGACATGGATCGCGAAGAGGTGGTCGAAGGCCTCATCGCGGGCAGTTCGTACAACACGCTGTCCATCGACAGCGGCGGCGGTGGCGACGAGGAAGCCCCCGCGATCGTCGACACGCTCGGCGATCTCGACATGGGCCTCGACCAGATCGACAACCGCGAATCGCTGCGCCCGCTGCTGGCCAGCCTGCCGGAGCGCGAGCGCACCGTGCTGGTGCTGCGATTCTTCGAATCGATGACGCAGACCCAGATCGCCGAGCGGGTCGGCATCTCGCAGATGCACGTCTCCCGTCTGCTGGCCAAGTCGCTGGCGCGGCTGC*GCGACCAGCTGCAGTAG*

*MSMEG_0467 (with upstream)* (740 bp)

*TAATACGACTCACTATAGGCCGGCGAAGAACCGACGTGACACTC*ATATCTCTGACCTTAGTCAGAGATATTTTCGATGCCCAGTTTAAATAAGCAGCACTAATCCATATGTAGAATCATTAGCTGTACTTGATCGACACGCGCTCATAACGTCGACCGCGTGAACTCCCCGCTGGTCCTCGGTTTCCTCACCTCGATGGCACTCATCGCCGCGATCGGCGCGCAGAACGCGTTCGTGCTGCGCCAGGGCATCCGACGCGAGCATGTCCTGCCCGTGATCGCGGTCTGCACGGTGTCAGACCTGCTGCTGATCACCGCGGGCATTGCCGGCGTCGGCGCGGTGATCACCGCACACCCCGATGCCGTAACGGTCACGAAGTTCGGCGGCGCAGCGTTCCTCATCGGCTACGGCGTGCTCGCGGCACGCCGGGCTTTGCGTCCGTCGACGCTCAATCCGTCCGAGCGCACCCCGGCGCGCCTCGCCGAAGTGCTCGTCACGTGCCTCGCGCTGACCTGGCTGAACCCGCACGTCTACCTCGACACCGTGGTGCTGCTGGGCACCCTGGCCAACGAACAACGCGAGCAACGGTGGCTCTTCGGCGCCGGCGCGGTGCTGGCGAGCGCCATCTGGTTCCTGGGTCTCGGTCTGGGCGCCAAGCGCCTGGCCGGGCTGTTCGCCACTCCGATGACCTGGCGCATCCTCGACGGCGTGATCGCCGTGACGATGATCGCGCT*CGGCCTCGGCATGATGCTGACGTGA*
