# Supplementary material for: Post-transcriptional modulation of the SigF regulon in Mycobacterium smegmatis by the PhoH2 toxin-antitoxin
Source: PLoS One. 2020 Jul 29;15(7):e0236551. doi: 10.1371/journal.pone.0236551 (PMC7390352; doi:10.1371/journal.pone.0236551)
Supplement: S2 File — (DOCX) [file pone.0236551.s006.docx]

**File S2: Summary of sequencing statistics and list of differentially expressed genes (DEGS) (FDR ≤ 0.001 ≥2 Log2 ratio ≥75 reads)**

|  | **Total reads** | | **Total bp** | | **Total mapped reads** | | **Total unmapped reads** | |
| --- | --- | --- | --- | --- | --- | --- | --- | --- |
| ***mc^2^155*** | 10634238 | 100% | 1595135700 | 100% | 10027008 | 94.29% | 607230 | 5.71% |
| ***ΔphoH2*** | 10002748 | 100% | 1500412200 | 100% | 9310561 | 93.08% | 692187 | 6.92% |

| DEGs | Product | SigF regulon  Y/N | mc^2^155  reads | ∆phoH2  reads | mc^2^155  RPKM | ∆phoH2  RPKM | log2 Ratio | P-value | FDR |
| --- | --- | --- | --- | --- | --- | --- | --- | --- | --- |
| Upregulated |  |  |  |  |  |  |  |  |  |
| MSMEG_0266 | Arginine decarboxylase | Y | 225 | 5298 | 34.01036 | 928.5631 | 4.770953697 | 2.31E-14 | 1.17E-13 |
| MSMEG_0267 | Esterase | Y | 151 | 10432 | 32.72218 | 2621.221 | 6.323826174 | 0 | 0 |
| MSMEG_0280 | Alpha/beta hydrolase | Y | 194 | 5443 | 47.50567 | 1545.442 | 5.023776238 | 4.78E-13 | 2.05E-12 |
| MSMEG_0536 | Intracellular protease PfpI family protein | Y | 265 | 3593 | 103.0028 | 1619.314 | 3.97462743 | 5.21E-13 | 2.22E-12 |
| MSMEG_0600 | Dehydrogenase | Y | 135 | 2660 | 37.99778 | 868.1142 | 4.513897693 | 1.36E-13 | 6.28E-13 |
| MSMEG_0637 | Iron-Sulfur binding oxidoreductase | Y | 666 | 11295 | 93.54865 | 1839.587 | 4.297521043 | 0 | 0 |
| MSMEG_0670 | FAD dependent oxidoreductase | Y | 101 | 1447 | 23.62962 | 392.5316 | 4.054140483 | 0 | 0 |
| MSMEG_0671 | S-(hydroxymethyl)glutathione dehydrogenase | Y | 163 | 3876 | 31.26467 | 862.0267 | 4.785127461 | 1.61E-13 | 7.40E-13 |
| MSMEG_0672 | Hypothetical protein | Y | 539 | 26185 | 181.634 | 10231.32 | 5.815814281 | 6.06E-13 | 2.57E-12 |
| MSMEG_0685 | Oxidoreductase, molybdopterin-binding subunit | Y | 76 | 2971 | 16.81669 | 762.255 | 5.502308136 | 0 | 0 |
| MSMEG_0686 | Oxidoreductase | Y | 177 | 5500 | 74.72897 | 2692.459 | 5.171113113 | 0 | 0 |
| MSMEG_1076 | Hypothetical protein | Y | 486 | 10593 | 388.073 | 9807.693 | 4.659513862 | 2.07E-13 | 9.37E-13 |
| MSMEG_1097 | Glycosyl transferase family protein | Y | 562 | 18016 | 181.0783 | 6730.685 | 5.216067551 | 3.63E-13 | 1.59E-12 |
| MSMEG_1112 | Aconitate hydratase | Y | 673 | 10784 | 75.48119 | 1402.408 | 4.215644846 | 8.48E-13 | 3.51E-12 |
| MSMEG_1605 | Phosphate transporter regulatory protein PhoU | Y | 490 | 7417 | 163.6207 | 2871.715 | 4.133484874 | 0 | 0 |
| MSMEG_1766 | Hypothetical | Y | 105 | 3736 | 94.06768 | 3880.867 | 5.366535981 | 0 | 0 |
| MSMEG_1767 | Hypothetical | Y | 122 | 3794 | 68.94162 | 2485.935 | 5.172269385 | 1.93E-14 | 9.85E-14 |
| MSMEG_1768 | Hypothetical | Y | 147 | 2543 | 52.42218 | 1051.514 | 4.326146161 | 5.86E-14 | 2.86E-13 |
| MSMEG_1770 | Hypothetical protein | Y | 392 | 7756 | 355.5216 | 8156.212 | 4.519890003 | 0 | 0 |
| MSMEG_1771 | Methylase | Y | 1027 | 29152 | 338.3222 | 11135.23 | 5.040589537 | 0 | 0 |
| MSMEG_1772 | Hypothetical protein | Y | 85 | 1394 | 86.72642 | 1649.172 | 4.249126669 | 0 | 0 |
| MSMEG_1773 | Hypothetical protein | Y | 1180 | 15149 | 254.21 | 3784.123 | 3.895866558 | 2.38E-12 | 9.42E-12 |
| MSMEG_1774 | Hypothetical protein | Y | 246 | 6562 | 109.5257 | 3387.568 | 4.950908133 | 1.93E-13 | 8.74E-13 |
| MSMEG_1775 | Cytochrome P450 monooxygenase | Y | 105 | 4422 | 16.84181 | 822.4111 | 5.609740552 | 0 | 0 |
| MSMEG_1777 | UsfY protein | Y | 87 | 1715 | 62.65909 | 1432.187 | 4.514552125 | 0 | 0 |
| MSMEG_1782 | Oxidoreductase | Y | 221 | 13414 | 54.8486 | 3860.133 | 6.137052087 | 1.07E-13 | 5.03E-13 |
| MSMEG_1787 | RsbW protein | Y | 113 | 3114 | 42.57051 | 1360.253 | 4.997877026 | 2.41E-13 | 1.08E-12 |
| MSMEG_1788 | Hypothetical protein | Y | 878 | 16884 | 758.8231 | 16919.68 | 4.478794744 | 6.49E-13 | 2.72E-12 |
| MSMEG_1790 | Hypothetical protein | Y | 768 | 9181 | 656.0361 | 9093.42 | 3.792975845 | 0 | 0 |
| MSMEG_1792 | Hypothetical protein | Y | 147 | 3657 | 92.29888 | 2662.409 | 4.850275326 | 5.86E-14 | 2.86E-13 |
| MSMEG_1794 | Dehydrogenase | Y | 308 | 9476 | 67.34051 | 2402.268 | 5.156778702 | 0 | 0 |
| MSMEG_1802 | ChaB protein | Y | 172 | 7825 | 90.25378 | 4760.932 | 5.721113041 | 4.64E-14 | 2.30E-13 |
| MSMEG_1950 | Hypothetical protein | Y | 259 | 10055 | 100.1408 | 4507.795 | 5.492319933 | 0 | 0 |
| MSMEG_1951 | Hypothetical protein | Y | 273 | 5032 | 90.74764 | 1939.474 | 4.417661825 | 1.31E-13 | 6.05E-13 |
| MSMEG_1971 | Propane monooxygenase hydroxylase large subunit | N | 3407 | 36811 | 460.9324 | 5774.488 | 3.647065849 | 0 | 0 |
| MSMEG_1974 | Propane monooxygenase coupling protein | N | 125 | 1627 | 79.85041 | 1205.106 | 3.91571701 | 0 | 0 |
| MSMEG_1975 | Amidohydrolase | N | 510 | 9312 | 107.6604 | 2279.289 | 4.404024665 | 0 | 0 |
| MSMEG_1976 | Hypothetical, (Iron-sulfur cluster assembly protein) | N | 448 | 7637 | 132.7062 | 2623.052 | 4.304938146 | 0 | 0 |
| MSMEG_1977 | Alcohol dehydrogenase | N | 695 | 10176 | 149.2876 | 2534.467 | 4.085516547 | 7.13E-13 | 2.97E-12 |
| MSMEG_1978 | Molecular chaperone groEL | N | 1045 | 13888 | 139.5785 | 2150.863 | 3.945766765 | 0 | 0 |
| MSMEG_1979 | Antibiotic biosynthesis monooxygenase | N | 178 | 1949 | 114.7044 | 1456.273 | 3.666287704 | 4.91E-14 | 2.42E-13 |
| MSMEG_2115 | Hypothetical protein | Y | 250 | 2550 | 103.1775 | 1220.27 | 3.564000006 | 1.47E-13 | 6.73E-13 |
| MSMEG_2347 | Phytoene dehydrogenase | Y | 268 | 2184 | 38.00756 | 359.1357 | 3.24017071 | 3.75E-14 | 1.88E-13 |
| MSMEG_2376 | Hypothetical protein | Y | 161 | 6143 | 76.80158 | 3397.782 | 5.467313549 | 0 | 0 |
| MSMEG_2401 | Hypothetical (no conserved domains detected) | N | 1546 | 13688 | 540.823 | 5552.089 | 3.3598022 | 3.03E-12 | 1.18E-11 |
| MSMEG_2415 | Hemerythrin HHE cation binding protein | Y | 490 | 14702 | 186.5107 | 6488.657 | 5.120589627 | 0 | 0 |
| MSMEG_2913 | Hydrolase | Y | 130 | 3719 | 33.1601 | 1099.941 | 5.051833979 | 0 | 0 |
| MSMEG_2926 | Glycine betaine/carnitine/choline transport ATP-binding protein | Y | 1209 | 4491 | 224.8507 | 968.46 | 2.106725237 | 2.83E-12 | 1.11E-11 |
| MSMEG_2927 | ABC transporter permease | Y | 785 | 5001 | 266.9813 | 1972.145 | 2.884954805 | 0 | 0 |
| MSMEG_2958 | Hypothetical protein | Y | 539 | 9353 | 164.9843 | 3319.521 | 4.330574769 | 6.06E-13 | 2.57E-12 |
| MSMEG_3022 | Transglycosylase associated protein | Y | 415 | 7411 | 298.891 | 6188.884 | 4.371987743 | 0 | 0 |
| MSMEG_3186 | Glycogen debranching protein GlgX | Y | 379 | 7694 | 38.72356 | 911.5049 | 4.556966836 | 3.42E-14 | 1.72E-13 |
| MSMEG_3255 | DoxX subfamily protein; | Y | 1154 | 20551 | 290.3273 | 5994.954 | 4.367996227 | 5.68E-12 | 2.16E-11 |
| MSMEG_3289 | gp61 protein | Y | 229 | 12806 | 46.47206 | 3013.288 | 6.018831266 | 2.93E-13 | 1.30E-12 |
| MSMEG_3304 | Succinate semialdehyde dehydrogenase | Y | 139 | 3536 | 22.6414 | 667.8385 | 4.882464246 | 1.16E-13 | 5.43E-13 |
| MSMEG_3311 | Acyl carrier protein | Y | 139 | 3536 | 22.6414 | 667.8385 | 4.882464246 | 1.44E-14 | 7.43E-14 |
| MSMEG_3419 | Hypothetical protein | Y | 235 | 1333 | 120.7249 | 794.017 | 2.717446878 | 0 | 0 |
| MSMEG_3536 | Sugar transporter | Y | 528 | 19769 | 120.8353 | 5245.844 | 5.440060915 | 2.82E-14 | 1.43E-13 |
| MSMEG_3543 | Hypothetical protein | Y | 249 | 6153 | 29.26741 | 838.5764 | 4.840575105 | 4.03E-08 | 1.19E-07 |
| MSMEG_3580 | Antigen 85-C (Esterase family protein) | N | 313 | 4150 | 190.0308 | 2921.45 | 3.942379534 | 2.67E-11 | 9.72E-11 |
| MSMEG_4195 | Hypothetical protein | Y | 462 | 3226 | 46.17635 | 373.8632 | 3.017284441 | 1.99E-13 | 9.05E-13 |
| MSMEG_4618 | Isochorismatase | Y | 383 | 2697 | 275.844 | 2252.249 | 3.029441984 | 8.26E-14 | 3.94E-13 |
| MSMEG_4993 | Hypothetical protein | Y | 226 | 5431 | 92.2361 | 2570.059 | 4.800325945 | 1.21E-13 | 5.61E-13 |
| MSMEG_5078 | Glucose-1-phosphate adenylyltransferase | Y | 136 | 3740 | 109.7899 | 3500.789 | 4.994862473 | 0 | 0 |
| MSMEG_5188 | Caax amino protease | Y | 1114 | 6119 | 202.0669 | 1286.948 | 2.671049426 | 1.15E-13 | 5.38E-13 |
| MSMEG_5342 | Hypothetical protein | Y | 180 | 3397 | 34.88978 | 763.4701 | 4.451695166 | 2.66E-13 | 1.19E-12 |
| MSMEG_5400 | Dehydrogenase | Y | 168 | 5428 | 106.3938 | 3985.819 | 5.227390342 | 3.66E-09 | 1.16E-08 |
| MSMEG_5542 | Transcriptional regulator | Y | 273 | 12552 | 76.25562 | 4065.303 | 5.736375255 | 1.16E-13 | 5.42E-13 |
| MSMEG_5551 | Stas domain-containing protein | Y | 657 | 9446 | 423.3753 | 7057.954 | 4.059241019 | 1.14E-06 | 2.95E-06 |
| MSMEG_5606 | Cytochrome bd-I oxidase subunit II | Y | 142 | 3216 | 28.5018 | 748.4636 | 4.714807331 | 9.01E-07 | 2.37E-06 |
| MSMEG_5722 | hypothetical protein | Y | 143 | 3544 | 66.06994 | 1898.595 | 4.844794311 | 0 | 0 |
| MSMEG_5826 | Pyruvate decarboxylase | Y | 115 | 1418 | 18.12913 | 259.1946 | 3.837654526 | 6.25E-13 | 2.64E-12 |
| MSMEG_5936 | Hypothetical protein | Y | 272 | 6334 | 25.98409 | 701.5965 | 4.75494107 | 9.04E-05 | 0.0001799 |
| MSMEG_6213 | Manganese containing catalase | Y | 192 | 4806 | 61.05964 | 1772.179 | 4.859161192 | 9.07E-13 | 3.74E-12 |
| MSMEG_6232 | Catalase KatA | Y | 638 | 11398 | 240.3538 | 4978.858 | 4.372583223 | 0 | 0 |
| MSMEG_6305 | Hypothetical protein | Y | 807 | 20157 | 198.9401 | 5761.63 | 4.856071212 | 0 | 0 |
| MSMEG_6467 | Starvation-induced DNA protecting protein | Y | 281 | 7035 | 37.2616 | 1081.658 | 4.859411147 | 1.52E-12 | 6.11E-12 |
| MSMEG_6515 | Trehalose synthase | Y | 89 | 7192 | 27.2423 | 2552.549 | 6.549946634 | 1.61E-12 | 6.45E-12 |
| MSMEG_6541 | Anti-sigma factor antagonist | Y | 1680 | 44643 | 670.7434 | 20666.71 | 4.945403601 | 0 | 0 |
| MSMEG_6542 | B12-binding domain-containing protein | Y | 717 | 6154 | 84.81888 | 844.115 | 3.314982179 | 0 | 0 |
| MSMEG_6610 | Hypothetical protein | Y | 1258 | 7103 | 155.5819 | 1018.57 | 2.710799323 | 7.08E-08 | 2.05E-07 |
| MSMEG_6615 | Hypothetical protein | Y | 240 | 1419 | 48.17205 | 330.2456 | 2.777271038 | 8.44E-15 | 4.40E-14 |
| MSMEG_6663 | C5-O-methyltransferase | Y | 119 | 4539 | 26.17372 | 1157.576 | 5.466843769 | 5.34E-11 | 1.91E-10 |
| MSMEG_6665 | Integral membrane protein | Y | 121 | 1046 | 39.6828 | 397.7584 | 3.325306658 | 1.17E-13 | 5.45E-13 |
| MSMEG_6727 | Amino acid permease | Y | 796 | 26248 | 148.4164 | 5674.606 | 5.256798017 | 0 | 0 |
| MSMEG_6767 | Mycocerosic acid synthase | Y | 188 | 7284 | 150.1188 | 6744.004 | 5.489429115 | 0 | 0 |
| MSMEG_6768 | Halogenase | Y | 171 | 12876 | 21.51039 | 1878.036 | 6.448047106 | 2.96E-13 | 1.31E-12 |
| Downregulated |  |  |  |  |  |  |  |  |  |
| MSMEG_0586 | STAS domain-containing protein | Y | 35183 | 3963 | 19881.74 | 2596.668 | -2.936710873 | 0 | 0 |

SigF Y/N – regulons reported by Humpel et al., 2010 and Singh et al., 2015

**Raw list of all DEGs (FDR ≤ 0.001 ≥1 Log2 ratio) as received from the Beijing Genomics Institute (used as source of genes for “list of differentially expressed genes (DEGS) (FDR ≤ 0.001 ≥2 Log2 ratio ≥75 reads)”.**

| GeneID | mc^2^155 Reads | ΔphoH2 Reads | mc^2^155 RPKM | ΔphoH2 RPKM | log2 Ratio | P-value | FDR |
| --- | --- | --- | --- | --- | --- | --- | --- |
| MSMEG_3722 | 0 | 128 | 0 | 117.2364703 | 16.839062 | 0 | 0 |
| MSMEG_4636 | 0 | 13 | 0 | 15.16897395 | 13.888836 | 4.17E-05 | 8.80E-05 |
| MSMEG_0159 | 0 | 28 | 0 | 14.72240397 | 13.845726 | 4.03E-10 | 1.37E-09 |
| MSMEG_4157 | 0 | 24 | 0 | 14.29588078 | 13.803312 | 8.75E-09 | 2.71E-08 |
| MSMEG_4031 | 0 | 10 | 0 | 12.52641514 | 13.612686 | 0.0004199 | 0.0007604 |
| MSMEG_2844 | 0 | 75 | 0 | 11.87448275 | 13.535577 | 0 | 0 |
| MSMEG_0573 | 0 | 27 | 0 | 11.55703427 | 13.496484 | 8.69E-10 | 2.89E-09 |
| MSMEG_6442 | 0 | 16 | 0 | 11.26342122 | 13.459357 | 4.14E-06 | 1.01E-05 |
| MSMEG_4419 | 0 | 31 | 0 | 9.889768957 | 13.271721 | 4.00E-11 | 1.44E-10 |
| MSMEG_4423 | 0 | 37 | 0 | 9.757418109 | 13.252284 | 3.94E-13 | 1.71E-12 |
| MSMEG_0345 | 0 | 31 | 0 | 9.672411398 | 13.23966 | 4.00E-11 | 1.44E-10 |
| MSMEG_1744 | 0 | 33 | 0 | 9.18603777 | 13.165227 | 8.57E-12 | 3.22E-11 |
| MSMEG_6633 | 0 | 11 | 0 | 9.096852937 | 13.151152 | 0.0001944 | 0.0003667 |
| MSMEG_4860 | 0 | 29 | 0 | 8.917722259 | 13.12246 | 1.86E-10 | 6.46E-10 |
| MSMEG_6713 | 0 | 30 | 0 | 8.751331126 | 13.095287 | 8.63E-11 | 3.06E-10 |
| MSMEG_4413 | 0 | 10 | 0 | 8.517962296 | 13.056293 | 0.0004199 | 0.000761 |
| MSMEG_3554 | 0 | 33 | 0 | 8.267433993 | 13.013224 | 8.57E-12 | 3.22E-11 |
| MSMEG_4386 | 0 | 31 | 0 | 8.175134092 | 12.997027 | 4.00E-11 | 1.44E-10 |
| MSMEG_0649 | 0 | 30 | 0 | 7.935989716 | 12.954194 | 8.63E-11 | 3.05E-10 |
| MSMEG_4441 | 0 | 28 | 0 | 7.8713843 | 12.942402 | 4.03E-10 | 1.37E-09 |
| MSMEG_4033 | 0 | 20 | 0 | 7.814644308 | 12.931964 | 1.90E-07 | 5.31E-07 |
| MSMEG_2853 | 0 | 24 | 0 | 7.543582845 | 12.881034 | 8.75E-09 | 2.71E-08 |
| MSMEG_0723 | 0 | 16 | 0 | 7.529690427 | 12.878375 | 4.14E-06 | 1.01E-05 |
| MSMEG_4865 | 0 | 27 | 0 | 7.491367491 | 12.871013 | 8.69E-10 | 2.89E-09 |
| MSMEG_4843 | 0 | 13 | 0 | 7.481993908 | 12.869207 | 4.17E-05 | 8.80E-05 |
| MSMEG_1162 | 0 | 37 | 0 | 7.019256235 | 12.777102 | 3.94E-13 | 1.71E-12 |
| MSMEG_4428 | 0 | 12 | 0 | 7.0010649 | 12.773359 | 9.00E-05 | 0.0001799 |
| MSMEG_4869 | 0 | 24 | 0 | 6.92986763 | 12.758612 | 8.75E-09 | 2.71E-08 |
| MSMEG_0145 | 0 | 18 | 0 | 6.875485261 | 12.747246 | 8.88E-07 | 2.34E-06 |
| MSMEG_2508 | 0 | 10 | 0 | 6.814369836 | 12.734365 | 0.0004199 | 0.0007606 |
| MSMEG_2356 | 0 | 22 | 0 | 6.76516861 | 12.72391 | 4.08E-08 | 1.21E-07 |
| MSMEG_3912 | 0 | 14 | 0 | 6.343163412 | 12.630987 | 1.93E-05 | 4.30E-05 |
| MSMEG_4732 | 0 | 20 | 0 | 6.3096017 | 12.623333 | 1.90E-07 | 5.31E-07 |
| MSMEG_2978 | 0 | 17 | 0 | 6.268630261 | 12.613935 | 1.92E-06 | 4.85E-06 |
| MSMEG_6836 | 0 | 10 | 0 | 6.217490727 | 12.602117 | 0.0004199 | 0.0007608 |
| MSMEG_6530 | 0 | 14 | 0 | 6.211014174 | 12.600613 | 1.93E-05 | 4.29E-05 |
| MSMEG_5364 | 0 | 23 | 0 | 6.16079034 | 12.5889 | 1.89E-08 | 5.72E-08 |
| MSMEG_3700 | 0 | 32 | 0 | 6.139071925 | 12.583805 | 1.85E-11 | 6.80E-11 |
| MSMEG_6631 | 0 | 14 | 0 | 5.903538225 | 12.527364 | 1.93E-05 | 4.30E-05 |
| MSMEG_4501 | 0 | 31 | 0 | 5.790719982 | 12.499527 | 4.00E-11 | 1.44E-10 |
| MSMEG_5881 | 0 | 15 | 0 | 5.391115377 | 12.396368 | 8.94E-06 | 2.09E-05 |
| MSMEG_5624 | 0 | 19 | 0 | 5.271703049 | 12.364053 | 4.11E-07 | 1.10E-06 |
| MSMEG_1085 | 0 | 19 | 0 | 5.057540113 | 12.30422 | 4.11E-07 | 1.11E-06 |
| MSMEG_4875 | 0 | 16 | 0 | 4.920122626 | 12.264479 | 4.14E-06 | 1.01E-05 |
| MSMEG_0555 | 0 | 15 | 0 | 4.839751304 | 12.240717 | 8.94E-06 | 2.09E-05 |
| MSMEG_3362 | 0 | 15 | 0 | 4.839751304 | 12.240717 | 8.94E-06 | 2.09E-05 |
| MSMEG_5620 | 0 | 14 | 0 | 4.586595082 | 12.163208 | 1.93E-05 | 4.30E-05 |
| MSMEG_2854 | 0 | 15 | 0 | 4.530831008 | 12.14556 | 8.94E-06 | 2.09E-05 |
| MSMEG_4426 | 0 | 14 | 0 | 4.517101217 | 12.141182 | 1.93E-05 | 4.30E-05 |
| MSMEG_1081 | 0 | 13 | 0 | 4.376818571 | 12.095667 | 4.17E-05 | 8.79E-05 |
| MSMEG_2906 | 0 | 14 | 0 | 4.336417169 | 12.082288 | 1.93E-05 | 4.30E-05 |
| MSMEG_4374 | 0 | 16 | 0 | 4.258981148 | 12.056293 | 4.14E-06 | 1.01E-05 |
| MSMEG_0288 | 0 | 18 | 0 | 4.143873549 | 12.016764 | 8.88E-07 | 2.34E-06 |
| MSMEG_0198 | 0 | 16 | 0 | 3.605486686 | 11.815978 | 4.14E-06 | 1.01E-05 |
| MSMEG_2847 | 0 | 22 | 0 | 3.542441786 | 11.790528 | 4.08E-08 | 1.21E-07 |
| MSMEG_5618 | 0 | 12 | 0 | 2.980045118 | 11.541118 | 9.00E-05 | 0.00018 |
| MSMEG_1784 | 0 | 11 | 0 | 2.763940568 | 11.432511 | 0.0001944 | 0.0003666 |
| MSMEG_3727 | 2 | 2471 | 0.935826463 | 1340.629607 | 10.484382 | 0.0001311 | 0.0002547 |
| MSMEG_3726 | 14 | 9526 | 1.830023635 | 1443.809766 | 9.6238027 | 3.76E-07 | 1.02E-06 |
| MSMEG_6667 | 6 | 1199 | 2.49025008 | 577.0077284 | 7.8561562 | 2.46E-05 | 5.40E-05 |
| MSMEG_3724 | 8 | 1592 | 1.952488434 | 450.5181387 | 7.8501274 | 9.19E-06 | 2.14E-05 |
| MSMEG_3719 | 4 | 705 | 0.75346028 | 153.9785492 | 7.6749822 | 6.06E-05 | 0.0001248 |
| MSMEG_3721 | 4 | 561 | 0.779441669 | 126.7527015 | 7.3453597 | 6.06E-05 | 0.0001252 |
| MSMEG_6354 | 89 | 7192 | 27.24229827 | 2552.549368 | 6.5499466 | 0 | 0 |
| MSMEG_6768 | 171 | 12876 | 21.51038789 | 1878.03566 | 6.4480471 | 2.96E-13 | 1.31E-12 |
| MSMEG_3082 | 6 | 449 | 3.364689039 | 291.9515321 | 6.4391119 | 2.46E-05 | 5.40E-05 |
| MSMEG_0697 | 45 | 3301 | 23.95512305 | 2037.521271 | 6.4103371 | 6.42E-14 | 3.12E-13 |
| MSMEG_0267 | 151 | 10432 | 32.72217988 | 2621.220728 | 6.3238262 | 0 | 0 |
| MSMEG_5342 | 41 | 2718 | 27.38143156 | 2104.711047 | 6.2642805 | 2.66E-13 | 1.19E-12 |
| MSMEG_5243 | 12 | 731 | 5.956408974 | 420.7182728 | 6.1422679 | 1.12E-06 | 2.91E-06 |
| MSMEG_1782 | 221 | 13414 | 54.8485993 | 3860.133319 | 6.1370521 | 1.07E-13 | 5.03E-13 |
| MSMEG_3289 | 229 | 12806 | 46.47205639 | 3013.287988 | 6.0188313 | 2.93E-13 | 1.30E-12 |
| MSMEG_0672 | 539 | 26185 | 181.6340431 | 10231.32306 | 5.8158143 | 6.06E-13 | 2.57E-12 |
| MSMEG_0185 | 2 | 95 | 0.154009177 | 8.482247569 | 5.7833584 | 0.0001311 | 0.0002544 |
| MSMEG_6233 | 30 | 1407 | 8.192830187 | 445.5305929 | 5.7650188 | 4.28E-11 | 1.54E-10 |
| MSMEG_0450 | 3 | 140 | 3.060932389 | 165.6270446 | 5.7578233 | 9.04E-05 | 0.0001801 |
| MSMEG_3312 | 18 | 837 | 7.109262324 | 383.3083033 | 5.7526616 | 4.03E-08 | 1.20E-07 |
| MSMEG_1315 | 60 | 2769 | 14.45161522 | 773.3192655 | 5.7417615 | 0 | 0 |
| MSMEG_5402 | 273 | 12552 | 76.25562363 | 4065.302766 | 5.7363753 | 1.31E-13 | 6.05E-13 |
| MSMEG_1802 | 172 | 7825 | 90.25377788 | 4760.932497 | 5.721113 | 4.64E-14 | 2.30E-13 |
| MSMEG_0696 | 23 | 1031 | 14.82135683 | 770.352555 | 5.6997694 | 2.35E-09 | 7.61E-09 |
| MSMEG_5936 | 3 | 130 | 1.721774469 | 86.51055456 | 5.6509081 | 9.04E-05 | 0.0001799 |
| MSMEG_5722 | 62 | 2616 | 72.29630786 | 3536.982439 | 5.6124533 | 0 | 0 |
| MSMEG_1775 | 105 | 4422 | 16.8418114 | 822.4111195 | 5.6097406 | 0 | 0 |
| MSMEG_1785 | 4 | 168 | 0.779441669 | 37.95802827 | 5.6058202 | 6.06E-05 | 0.0001251 |
| MSMEG_0685 | 76 | 2971 | 16.81668879 | 762.2549994 | 5.5023081 | 0 | 0 |
| MSMEG_1950 | 259 | 10055 | 100.1408196 | 4507.79531 | 5.4923199 | 0 | 0 |
| MSMEG_6665 | 188 | 7284 | 150.1187711 | 6744.004061 | 5.4894291 | 1.17E-13 | 5.45E-13 |
| MSMEG_2376 | 161 | 6143 | 76.80157632 | 3397.781973 | 5.4673135 | 0 | 0 |
| MSMEG_6612 | 119 | 4539 | 26.17372127 | 1157.575774 | 5.4668438 | 0 | 0 |
| MSMEG_0265 | 44 | 1665 | 15.39211716 | 675.3527249 | 5.4553776 | 1.22E-13 | 5.66E-13 |
| MSMEG_3419 | 528 | 19769 | 120.8353123 | 5245.844132 | 5.4400609 | 0 | 0 |
| MSMEG_3273 | 55 | 2045 | 11.16141092 | 481.1942789 | 5.4300282 | 1.62E-14 | 8.31E-14 |
| MSMEG_5496 | 18 | 668 | 3.155901652 | 135.7994944 | 5.4272821 | 4.03E-08 | 1.20E-07 |
| MSMEG_5559 | 33 | 1191 | 5.136140789 | 214.9341757 | 5.3870663 | 7.98E-12 | 3.02E-11 |
| MSMEG_1360 | 60 | 2156 | 17.63097056 | 734.5890684 | 5.3807536 | 0 | 0 |
| MSMEG_1766 | 105 | 3736 | 94.06767831 | 3880.866724 | 5.366536 | 0 | 0 |
| MSMEG_5106 | 2 | 70 | 0.253318543 | 10.28029932 | 5.3427858 | 0.0001311 | 0.0002545 |
| MSMEG_5627 | 2 | 70 | 0.317332084 | 12.87812874 | 5.3427858 | 0.0001311 | 0.0002545 |
| MSMEG_6616 | 796 | 26248 | 148.4163766 | 5674.605948 | 5.256798 | 5.12E-13 | 2.18E-12 |
| MSMEG_3593 | 49 | 1601 | 14.81340119 | 561.2040179 | 5.2435505 | 4.73E-14 | 2.33E-13 |
| MSMEG_5343 | 168 | 5428 | 106.3937879 | 3985.818909 | 5.2273903 | 8.97E-14 | 4.26E-13 |
| MSMEG_1097 | 562 | 18016 | 181.0783161 | 6730.684593 | 5.2160676 | 3.63E-13 | 1.59E-12 |
| MSMEG_3543 | 18 | 574 | 7.470750239 | 276.2322236 | 5.2084847 | 4.03E-08 | 1.19E-07 |
| MSMEG_2837 | 99 | 3092 | 9.136652459 | 330.8736108 | 5.1784707 | 0 | 0 |
| MSMEG_1767 | 122 | 3794 | 68.94161566 | 2485.934535 | 5.1722694 | 1.93E-14 | 9.85E-14 |
| MSMEG_0686 | 177 | 5500 | 74.72897006 | 2692.459346 | 5.1711131 | 0 | 0 |
| MSMEG_1794 | 308 | 9476 | 67.34051257 | 2402.268176 | 5.1567787 | 0 | 0 |
| MSMEG_1358 | 33 | 997 | 12.96394894 | 454.1394871 | 5.1305583 | 7.98E-12 | 3.01E-11 |
| MSMEG_2415 | 490 | 14702 | 186.510699 | 6488.657081 | 5.1205896 | 0 | 0 |
| MSMEG_4521 | 2 | 60 | 0.377698598 | 13.13824519 | 5.1203934 | 0.0001311 | 0.0002551 |
| MSMEG_2037 | 120 | 3475 | 26.71359176 | 896.9672417 | 5.0694094 | 0 | 0 |
| MSMEG_6305 | 73 | 2100 | 43.59962233 | 1454.286246 | 5.0598518 | 0 | 0 |
| MSMEG_2913 | 130 | 3719 | 33.16010089 | 1099.941034 | 5.051834 | 0 | 0 |
| MSMEG_6769 | 19 | 540 | 3.268817727 | 107.721303 | 5.0423908 | 2.29E-08 | 6.88E-08 |
| MSMEG_1771 | 1027 | 29152 | 338.322249 | 11135.23035 | 5.0405895 | 0 | 0 |
| MSMEG_1783 | 64 | 1799 | 61.05963831 | 1990.105736 | 5.0264822 | 0 | 0 |
| MSMEG_0280 | 194 | 5443 | 47.50567068 | 1545.442292 | 5.0237762 | 4.78E-13 | 2.05E-12 |
| MSMEG_4064 | 2 | 56 | 0.439894475 | 14.28161343 | 5.0208577 | 0.0001311 | 0.0002548 |
| MSMEG_1844 | 13 | 360 | 5.457205174 | 175.2266529 | 5.0049161 | 6.50E-07 | 1.73E-06 |
| MSMEG_1787 | 113 | 3114 | 42.57050585 | 1360.253056 | 4.997877 | 2.41E-13 | 1.08E-12 |
| MSMEG_4993 | 136 | 3740 | 109.7899266 | 3500.7889 | 4.9948625 | 1.21E-13 | 5.61E-13 |
| MSMEG_6727 | 77 | 2084 | 12.59822507 | 395.3548647 | 4.9718558 | 0 | 0 |
| MSMEG_1774 | 246 | 6562 | 109.5257262 | 3387.567793 | 4.9509081 | 1.93E-13 | 8.74E-13 |
| MSMEG_6467 | 1680 | 44643 | 670.7434453 | 20666.70602 | 4.9454036 | 1.52E-12 | 6.11E-12 |
| MSMEG_6610 | 17 | 434 | 4.351430017 | 128.8082103 | 4.8875912 | 7.08E-08 | 2.05E-07 |
| MSMEG_3304 | 139 | 3536 | 22.6413979 | 667.8384629 | 4.8824642 | 1.16E-13 | 5.43E-13 |
| MSMEG_6232 | 281 | 7035 | 37.26160295 | 1081.658208 | 4.8594111 | 0 | 0 |
| MSMEG_6210 | 192 | 4806 | 61.05963831 | 1772.178649 | 4.8591612 | 0 | 0 |
| MSMEG_2859 | 2 | 50 | 0.370087543 | 10.72791221 | 4.8573589 | 0.0001311 | 0.000255 |
| MSMEG_0576 | 4 | 100 | 0.304823143 | 8.836060473 | 4.8573589 | 6.06E-05 | 0.0001247 |
| MSMEG_6213 | 807 | 20157 | 198.9400621 | 5761.629731 | 4.8560712 | 9.07E-13 | 3.74E-12 |
| MSMEG_1792 | 147 | 3657 | 92.29888436 | 2662.409241 | 4.8502753 | 5.86E-14 | 2.86E-13 |
| MSMEG_1758 | 50 | 1242 | 63.32963564 | 1824.018823 | 4.848096 | 5.26E-14 | 2.58E-13 |
| MSMEG_6242 | 61 | 1515 | 10.56887976 | 304.3564358 | 4.8478675 | 5.57E-14 | 2.73E-13 |
| MSMEG_6664 | 14 | 347 | 3.647068379 | 104.813224 | 4.8449397 | 3.76E-07 | 1.02E-06 |
| MSMEG_5617 | 143 | 3544 | 66.06993686 | 1898.594866 | 4.8447943 | 0 | 0 |
| MSMEG_3536 | 249 | 6153 | 29.26741113 | 838.5763521 | 4.8405751 | 2.82E-14 | 1.43E-13 |
| MSMEG_6151 | 57 | 1407 | 14.34025859 | 410.4374298 | 4.8390194 | 8.75E-14 | 4.16E-13 |
| MSMEG_0375 | 52 | 1274 | 7.194055785 | 204.3669297 | 4.8282126 | 7.62E-14 | 3.65E-13 |
| MSMEG_1001 | 187 | 4522 | 48.03309288 | 1346.791101 | 4.8093536 | 2.86E-13 | 1.27E-12 |
| MSMEG_4618 | 226 | 5431 | 92.236096 | 2570.058513 | 4.8003259 | 8.26E-14 | 3.94E-13 |
| MSMEG_0451 | 96 | 2300 | 84.96853283 | 2360.39919 | 4.7959584 | 0 | 0 |
| MSMEG_0671 | 163 | 3876 | 31.26466712 | 862.0266804 | 4.7851275 | 1.61E-13 | 7.40E-13 |
| MSMEG_0266 | 225 | 5298 | 34.01035988 | 928.5630502 | 4.7709537 | 2.31E-14 | 1.17E-13 |
| MSMEG_5347 | 2 | 47 | 0.333162709 | 9.078100406 | 4.7680916 | 0.0001311 | 0.0002552 |
| MSMEG_5386 | 2 | 47 | 0.749616095 | 20.42572591 | 4.7680916 | 0.0001311 | 0.0002542 |
| MSMEG_5826 | 272 | 6334 | 25.98409186 | 701.5965303 | 4.7549411 | 6.25E-13 | 2.64E-12 |
| MSMEG_0963 | 41 | 946 | 37.18466014 | 994.8138681 | 4.7416471 | 2.66E-13 | 1.19E-12 |
| MSMEG_5383 | 7 | 159 | 2.000920784 | 52.69867724 | 4.7190308 | 1.52E-05 | 3.42E-05 |
| MSMEG_5590 | 142 | 3216 | 28.50179667 | 748.4635722 | 4.7148073 | 1.85E-13 | 8.46E-13 |
| MSMEG_2966 | 2 | 45 | 0.315289173 | 8.225500071 | 4.7053559 | 0.0001311 | 0.0002542 |
| MSMEG_5938 | 178 | 3954 | 64.09952533 | 1650.981516 | 4.6868665 | 4.91E-14 | 2.41E-13 |
| MSMEG_4787 | 2 | 44 | 0.301693541 | 7.695900226 | 4.6729344 | 0.0001311 | 0.0002546 |
| MSMEG_1076 | 486 | 10593 | 388.0729934 | 9807.692891 | 4.6595139 | 2.07E-13 | 9.37E-13 |
| MSMEG_4549 | 2 | 43 | 0.292678794 | 7.296262524 | 4.6397675 | 0.0001311 | 0.0002552 |
| MSMEG_2112 | 257 | 5418 | 96.81964604 | 2366.683062 | 4.6114229 | 4.21E-13 | 1.81E-12 |
| MSMEG_5605 | 114 | 2396 | 18.08792876 | 440.7999495 | 4.6070249 | 9.59E-14 | 4.54E-13 |
| MSMEG_5529 | 2 | 42 | 0.454875402 | 11.07598812 | 4.6058202 | 0.0001311 | 0.0002549 |
| MSMEG_0781 | 2 | 42 | 0.279324629 | 6.801414761 | 4.6058202 | 0.0001311 | 0.0002549 |
| MSMEG_3186 | 379 | 7694 | 38.72356191 | 911.5048943 | 4.5569668 | 3.42E-14 | 1.72E-13 |
| MSMEG_6241 | 28 | 562 | 5.827044095 | 135.611751 | 4.5405742 | 1.34E-10 | 4.67E-10 |
| MSMEG_1770 | 392 | 7756 | 355.5216286 | 8156.211798 | 4.51989 | 0 | 0 |
| MSMEG_1777 | 87 | 1715 | 62.65908656 | 1432.186798 | 4.5145521 | 0 | 0 |
| MSMEG_0600 | 135 | 2660 | 37.99778139 | 868.114165 | 4.5138977 | 1.36E-13 | 6.28E-13 |
| MSMEG_0513 | 3 | 59 | 0.684432087 | 15.60744644 | 4.5111833 | 9.04E-05 | 0.0001801 |
| MSMEG_5305 | 2 | 39 | 0.313941784 | 7.098301913 | 4.498905 | 0.0001311 | 0.0002543 |
| MSMEG_1788 | 878 | 16884 | 758.8231448 | 16919.67946 | 4.4787947 | 6.49E-13 | 2.72E-12 |
| MSMEG_3438 | 50 | 960 | 64.44068188 | 1434.604176 | 4.4765372 | 5.26E-14 | 2.58E-13 |
| MSMEG_1084 | 2 | 38 | 0.27462571 | 6.050141444 | 4.4614303 | 0.0001311 | 0.000254 |
| MSMEG_1789 | 12 | 227 | 16.95285631 | 371.8418156 | 4.4550887 | 1.12E-06 | 2.91E-06 |
| MSMEG_5189 | 180 | 3397 | 34.88978344 | 763.4701298 | 4.4516952 | 2.56E-13 | 1.15E-12 |
| MSMEG_2706 | 11 | 204 | 3.544237504 | 76.21334686 | 4.4264965 | 1.92E-06 | 4.85E-06 |
| MSMEG_1951 | 273 | 5032 | 90.7476426 | 1939.474492 | 4.4176618 | 1.31E-13 | 6.05E-13 |
| MSMEG_1975 | 510 | 9312 | 107.6603806 | 2279.289221 | 4.4040247 | 0 | 0 |
| MSMEG_0705 | 4 | 72 | 0.664817895 | 13.87541369 | 4.3834278 | 6.06E-05 | 0.0001251 |
| MSMEG_3405 | 4 | 72 | 0.607127085 | 12.67134887 | 4.3834278 | 6.06E-05 | 0.0001248 |
| MSMEG_3369 | 2 | 36 | 0.33931814 | 7.081908606 | 4.3834278 | 0.0001311 | 0.0002539 |
| MSMEG_5623 | 2 | 36 | 0.382616549 | 7.985589652 | 4.3834278 | 0.0001311 | 0.0002538 |
| MSMEG_0344 | 2 | 36 | 0.571691652 | 11.93177598 | 4.3834278 | 0.0001311 | 0.0002541 |
| MSMEG_6212 | 638 | 11398 | 240.3538295 | 4978.858166 | 4.3725832 | 0 | 0 |
| MSMEG_3022 | 415 | 7411 | 298.8910451 | 6188.884174 | 4.3719877 | 0 | 0 |
| MSMEG_3255 | 1154 | 20551 | 290.3273406 | 5994.953532 | 4.3679962 | 5.68E-12 | 2.16E-11 |
| MSMEG_5385 | 54 | 938 | 8.623844297 | 173.6923616 | 4.3320594 | 6.86E-14 | 3.31E-13 |
| MSMEG_2958 | 539 | 9353 | 164.9842558 | 3319.52089 | 4.3305748 | 6.06E-13 | 2.57E-12 |
| MSMEG_3557 | 3 | 52 | 0.457234714 | 9.189502891 | 4.32898 | 9.04E-05 | 0.0001803 |
| MSMEG_1768 | 147 | 2543 | 52.42218189 | 1051.513501 | 4.3261462 | 5.86E-14 | 2.86E-13 |
| MSMEG_1976 | 448 | 7637 | 132.70623 | 2623.051534 | 4.3049381 | 0 | 0 |
| MSMEG_2899 | 2 | 34 | 0.633296356 | 12.48322061 | 4.3009656 | 0.0001311 | 0.0002538 |
| MSMEG_0637 | 666 | 11295 | 93.54864878 | 1839.586695 | 4.297521 | 0 | 0 |
| MSMEG_0512 | 4 | 66 | 0.898622353 | 17.19221748 | 4.2578969 | 6.06E-05 | 0.0001247 |
| MSMEG_2842 | 2 | 33 | 0.340103599 | 6.506776754 | 4.2578969 | 0.0001311 | 0.0002537 |
| MSMEG_1772 | 85 | 1394 | 86.7264177 | 1649.172144 | 4.2491267 | 0 | 0 |
| MSMEG_4032 | 3 | 49 | 0.678114252 | 12.84246623 | 4.2432501 | 9.04E-05 | 0.0001802 |
| MSMEG_5944 | 3 | 49 | 0.412709985 | 7.816107724 | 4.2432501 | 9.04E-05 | 0.0001803 |
| MSMEG_1112 | 673 | 10784 | 75.48119077 | 1402.407716 | 4.2156448 | 8.48E-13 | 3.51E-12 |
| MSMEG_0294 | 2 | 32 | 0.592438527 | 10.99091909 | 4.2135028 | 0.0001311 | 0.0002535 |
| MSMEG_2233 | 2 | 32 | 0.419785013 | 7.787851242 | 4.2135028 | 0.0001311 | 0.0002536 |
| MSMEG_3311 | 47 | 746 | 35.59517253 | 655.0927704 | 4.2019457 | 1.44E-14 | 7.43E-14 |
| MSMEG_5246 | 223 | 3530 | 49.64275802 | 911.1638456 | 4.1980553 | 1.88E-13 | 8.56E-13 |
| MSMEG_2215 | 3 | 47 | 0.439894475 | 7.990902752 | 4.1831291 | 9.04E-05 | 0.00018 |
| MSMEG_2982 | 3 | 47 | 0.521009768 | 9.464402551 | 4.1831291 | 9.04E-05 | 0.0001802 |
| MSMEG_4100 | 2 | 31 | 0.418589045 | 7.522986643 | 4.1676991 | 0.0001311 | 0.0002534 |
| MSMEG_3114 | 2 | 31 | 0.540164539 | 9.707971734 | 4.1676991 | 0.0001311 | 0.0002535 |
| MSMEG_1759 | 20 | 309 | 15.79836072 | 283.0161666 | 4.1630377 | 1.30E-08 | 3.97E-08 |
| MSMEG_4988 | 4 | 61 | 0.470912675 | 8.326854167 | 4.1442401 | 6.06E-05 | 0.000125 |
| MSMEG_1605 | 490 | 7417 | 163.6207495 | 2871.714834 | 4.1334849 | 0 | 0 |
| MSMEG_6048 | 2 | 30 | 0.38766426 | 6.742450366 | 4.1203934 | 0.0001311 | 0.0002533 |
| MSMEG_1229 | 6 | 88 | 0.688709788 | 11.71219816 | 4.0879719 | 2.46E-05 | 5.39E-05 |
| MSMEG_1977 | 695 | 10176 | 149.2875797 | 2534.467378 | 4.0855165 | 7.13E-13 | 2.97E-12 |
| MSMEG_0571 | 2 | 29 | 0.488122109 | 8.206674637 | 4.0714838 | 0.0001311 | 0.0002532 |
| MSMEG_0339 | 2 | 29 | 0.433406356 | 7.286752406 | 4.0714838 | 0.0001311 | 0.0002533 |
| MSMEG_5392 | 9 | 130 | 1.187004302 | 19.88034288 | 4.0659456 | 5.50E-06 | 1.32E-05 |
| MSMEG_4355 | 211 | 3036 | 47.54773503 | 793.2678997 | 4.0603596 | 2.50E-13 | 1.12E-12 |
| MSMEG_1972 | 502 | 7222 | 105.6679468 | 1762.65684 | 4.0601419 | 1.08E-12 | 4.40E-12 |
| MSMEG_5543 | 657 | 9446 | 423.37528 | 7057.953671 | 4.059241 | 0 | 0 |
| MSMEG_4156 | 8 | 115 | 0.792047195 | 13.20169358 | 4.0589928 | 9.19E-06 | 2.14E-05 |
| MSMEG_0670 | 101 | 1447 | 23.62961819 | 392.5315746 | 4.0541405 | 0 | 0 |
| MSMEG_6493 | 4 | 57 | 0.837178089 | 13.83258834 | 4.0463928 | 6.06E-05 | 0.0001251 |
| MSMEG_2555 | 30 | 424 | 15.97008203 | 261.7113053 | 4.0345326 | 4.28E-11 | 1.54E-10 |
| MSMEG_3940 | 6 | 84 | 1.499232191 | 24.33703513 | 4.0208577 | 2.46E-05 | 5.38E-05 |
| MSMEG_4887 | 3 | 42 | 0.549593845 | 8.921556519 | 4.0208577 | 9.04E-05 | 0.0001805 |
| MSMEG_4707 | 189 | 2634 | 48.7171555 | 787.2390416 | 4.0143 | 2.21E-13 | 9.98E-13 |
| MSMEG_6751 | 33 | 454 | 33.67025628 | 537.1048447 | 3.9956571 | 7.98E-12 | 3.01E-11 |
| MSMEG_4545 | 4 | 55 | 0.498050016 | 7.940473326 | 3.9948625 | 6.06E-05 | 0.0001248 |
| MSMEG_3402 | 3 | 41 | 0.485434212 | 7.69243291 | 3.9860923 | 9.04E-05 | 0.0001804 |
| MSMEG_0536 | 265 | 3593 | 103.0028042 | 1619.314208 | 3.9746274 | 5.21E-13 | 2.22E-12 |
| MSMEG_2980 | 2 | 27 | 0.390757326 | 6.116621861 | 3.9683903 | 0.0001312 | 0.0002532 |
| MSMEG_6116 | 2 | 27 | 0.83008336 | 12.99350181 | 3.9683903 | 0.0001312 | 0.0002531 |
| MSMEG_6333 | 2 | 27 | 0.398170067 | 6.232655338 | 3.9683903 | 0.0001312 | 0.0002533 |
| MSMEG_0808 | 2 | 27 | 0.40475139 | 6.335674435 | 3.9683903 | 0.0001312 | 0.0002531 |
| MSMEG_3141 | 327 | 4393 | 363.9726878 | 5669.607328 | 3.9613467 | 6.25E-13 | 2.64E-12 |
| MSMEG_2718 | 19 | 255 | 4.531770031 | 70.52209043 | 3.9599287 | 2.29E-08 | 6.89E-08 |
| MSMEG_1978 | 1045 | 13888 | 139.578517 | 2150.862916 | 3.9457668 | 0 | 0 |
| MSMEG_3560 | 313 | 4150 | 190.0307778 | 2921.449878 | 3.9423795 | 0 | 0 |
| MSMEG_4377 | 4 | 53 | 0.59604363 | 9.157241413 | 3.9414232 | 6.06E-05 | 0.000125 |
| MSMEG_1781 | 9 | 118 | 4.293255819 | 65.2675033 | 3.9262208 | 5.50E-06 | 1.32E-05 |
| MSMEG_1974 | 125 | 1627 | 79.85041016 | 1205.106492 | 3.915717 | 0 | 0 |
| MSMEG_4738 | 2 | 26 | 0.769239553 | 11.59513192 | 3.9139425 | 0.0001313 | 0.000253 |
| MSMEG_5876 | 18 | 234 | 11.70197161 | 176.3896617 | 3.9139425 | 4.03E-08 | 1.20E-07 |
| MSMEG_1490 | 2 | 26 | 0.430864383 | 6.494634008 | 3.9139425 | 0.0001313 | 0.0002531 |
| MSMEG_1159 | 2 | 26 | 0.411553935 | 6.203557974 | 3.9139425 | 0.0001313 | 0.0002531 |
| MSMEG_2165 | 2 | 26 | 0.510155398 | 7.689827072 | 3.9139425 | 0.0001313 | 0.0002528 |
| MSMEG_6451 | 2 | 26 | 1.121563013 | 16.90587937 | 3.9139425 | 0.0001313 | 0.0002529 |
| MSMEG_1773 | 1180 | 15149 | 254.2099861 | 3784.123484 | 3.8958666 | 2.38E-12 | 9.42E-12 |
| MSMEG_2716 | 13 | 166 | 4.36078039 | 64.5653763 | 3.8881025 | 6.50E-07 | 1.73E-06 |
| MSMEG_0433 | 4 | 51 | 0.625211722 | 9.242895257 | 3.8859281 | 6.06E-05 | 0.0001253 |
| MSMEG_2707 | 16 | 204 | 4.778040803 | 70.6367605 | 3.8859281 | 1.24E-07 | 3.50E-07 |
| MSMEG_4102 | 3 | 38 | 0.595640897 | 8.748177493 | 3.8764678 | 9.04E-05 | 0.0001799 |
| MSMEG_4533 | 38 | 481 | 7.975915255 | 117.061139 | 3.8754683 | 6.64E-13 | 2.78E-12 |
| MSMEG_6322 | 183 | 2313 | 29.3528713 | 430.1756941 | 3.8733525 | 2.51E-13 | 1.13E-12 |
| MSMEG_2713 | 11 | 139 | 5.019168639 | 73.54017137 | 3.8730122 | 1.92E-06 | 4.85E-06 |
| MSMEG_5596 | 2 | 25 | 0.583034741 | 8.45035942 | 3.8573589 | 0.0001316 | 0.0002528 |
| MSMEG_3401 | 2 | 25 | 0.552348702 | 8.005603661 | 3.8573589 | 0.0001316 | 0.0002531 |
| MSMEG_4539 | 2 | 25 | 0.398170067 | 5.770977165 | 3.8573589 | 0.0001316 | 0.000253 |
| MSMEG_4928 | 2 | 25 | 0.885088884 | 12.82825647 | 3.8573589 | 0.0001316 | 0.000253 |
| MSMEG_4030 | 2 | 25 | 0.895882651 | 12.98469862 | 3.8573589 | 0.0001316 | 0.0002529 |
| MSMEG_1711 | 587 | 7335 | 85.73044831 | 1242.132275 | 3.8568673 | 0 | 0 |
| MSMEG_3935 | 61 | 762 | 17.30194988 | 250.6056861 | 3.8564126 | 5.57E-14 | 2.73E-13 |
| MSMEG_5401 | 215 | 2679 | 98.71506956 | 1426.226312 | 3.8527888 | 0 | 0 |
| MSMEG_3996 | 6 | 74 | 0.903225951 | 12.91658217 | 3.8379936 | 2.46E-05 | 5.41E-05 |
| MSMEG_5799 | 115 | 1418 | 18.12912746 | 259.1946467 | 3.8376545 | 0 | 0 |
| MSMEG_2717 | 7 | 86 | 2.749928564 | 39.17351644 | 3.8324126 | 1.52E-05 | 3.42E-05 |
| MSMEG_2719 | 95 | 1162 | 11.65096135 | 165.2399364 | 3.8260415 | 0 | 0 |
| MSMEG_4737 | 5 | 60 | 1.147849646 | 15.9711793 | 3.7984653 | 3.92E-05 | 8.33E-05 |
| MSMEG_0114 | 2 | 24 | 0.428352054 | 5.960090236 | 3.7984653 | 0.0001321 | 0.0002536 |
| MSMEG_4543 | 2 | 24 | 0.447941325 | 6.232655338 | 3.7984653 | 0.0001321 | 0.0002535 |
| MSMEG_1240 | 6 | 72 | 0.833221671 | 11.59344585 | 3.7984653 | 2.46E-05 | 5.38E-05 |
| MSMEG_1714 | 64 | 767 | 20.26548341 | 281.6067707 | 3.7965855 | 0 | 0 |
| MSMEG_1790 | 768 | 9181 | 656.036114 | 9093.419981 | 3.7929758 | 0 | 0 |
| MSMEG_1715 | 108 | 1290 | 15.80465489 | 218.8878757 | 3.7917706 | 0 | 0 |
| MSMEG_3083 | 14 | 167 | 2.290586376 | 31.68150787 | 3.7898521 | 3.76E-07 | 1.02E-06 |
| MSMEG_1713 | 153 | 1813 | 19.92862364 | 273.8132206 | 3.7802781 | 0 | 0 |
| MSMEG_2703 | 4 | 47 | 3.09315273 | 42.14149767 | 3.7680916 | 6.06E-05 | 0.0001249 |
| MSMEG_1086 | 3 | 35 | 0.706369013 | 9.555406421 | 3.7578233 | 9.04E-05 | 0.0001798 |
| MSMEG_3310 | 66 | 768 | 17.50367114 | 236.165886 | 3.7540711 | 0 | 0 |
| MSMEG_1241 | 4 | 46 | 0.938816324 | 12.51841104 | 3.7370647 | 6.06E-05 | 0.0001246 |
| MSMEG_1160 | 2 | 23 | 0.548226697 | 7.310191522 | 3.7370647 | 0.000133 | 0.0002551 |
| MSMEG_1321 | 2 | 23 | 0.709781424 | 9.464402551 | 3.7370647 | 0.000133 | 0.0002552 |
| MSMEG_4548 | 2 | 23 | 0.447941325 | 5.972961366 | 3.7370647 | 0.000133 | 0.0002553 |
| MSMEG_0299 | 4 | 45 | 0.696325852 | 9.083135149 | 3.7053559 | 6.06E-05 | 0.0001246 |
| MSMEG_1847 | 25 | 280 | 6.02150634 | 78.19768665 | 3.6989296 | 7.47E-10 | 2.50E-09 |
| MSMEG_4101 | 4 | 44 | 0.539173412 | 6.876886991 | 3.6729344 | 6.06E-05 | 0.0001252 |
| MSMEG_4138 | 4 | 44 | 0.909750803 | 11.60341613 | 3.6729344 | 6.06E-05 | 0.0001249 |
| MSMEG_4798 | 3 | 33 | 0.546866333 | 6.975006346 | 3.6729344 | 9.04E-05 | 0.0001798 |
| MSMEG_4871 | 2 | 22 | 0.576175509 | 7.348830216 | 3.6729344 | 0.0001349 | 0.0002583 |
| MSMEG_1979 | 178 | 1949 | 114.7044137 | 1456.272677 | 3.6662877 | 4.91E-14 | 2.42E-13 |
| MSMEG_1971 | 3407 | 36811 | 460.9324486 | 5774.488215 | 3.6470658 | 0 | 0 |
| MSMEG_2873 | 6 | 64 | 0.831649555 | 10.28584126 | 3.6285403 | 2.46E-05 | 5.41E-05 |
| MSMEG_2705 | 32 | 340 | 6.566469483 | 80.89684862 | 3.6228937 | 1.39E-11 | 5.15E-11 |
| MSMEG_6374 | 5 | 53 | 1.077160958 | 13.23906163 | 3.6194951 | 3.92E-05 | 8.34E-05 |
| MSMEG_0178 | 2 | 21 | 0.562930095 | 6.853532882 | 3.6058202 | 0.0001387 | 0.0002652 |
| MSMEG_2857 | 4 | 42 | 0.81852231 | 9.965304078 | 3.6058202 | 6.06E-05 | 0.0001245 |
| MSMEG_3731 | 39 | 408 | 7.914455018 | 96.00355294 | 3.6005259 | 4.38E-13 | 1.88E-12 |
| MSMEG_2712 | 7 | 73 | 5.910765993 | 71.47255719 | 3.5959724 | 1.52E-05 | 3.42E-05 |
| MSMEG_1113 | 5 | 52 | 0.976893316 | 11.78016062 | 3.5920144 | 3.92E-05 | 8.33E-05 |
| MSMEG_3350 | 5 | 52 | 0.809057019 | 9.756256374 | 3.5920144 | 3.92E-05 | 8.33E-05 |
| MSMEG_6669 | 3 | 31 | 0.693041296 | 8.303673936 | 3.5827366 | 9.04E-05 | 0.0001798 |
| MSMEG_4864 | 4 | 41 | 0.499744064 | 5.939395478 | 3.5710548 | 6.06E-05 | 0.0001246 |
| MSMEG_2115 | 250 | 2550 | 103.1774963 | 1220.269879 | 3.564 | 1.47E-13 | 6.73E-13 |
| MSMEG_6875 | 6 | 61 | 1.169162504 | 13.78237931 | 3.5592776 | 2.46E-05 | 5.40E-05 |
| MSMEG_6838 | 3 | 30 | 1.281320535 | 14.85691098 | 3.5354309 | 9.04E-05 | 0.0001798 |
| MSMEG_4736 | 6 | 60 | 1.300219068 | 15.07603946 | 3.5354309 | 2.46E-05 | 5.40E-05 |
| MSMEG_3553 | 5 | 50 | 0.779855386 | 9.042422819 | 3.5354309 | 3.92E-05 | 8.33E-05 |
| MSMEG_5394 | 4 | 40 | 1.016780309 | 11.78956719 | 3.5354309 | 6.06E-05 | 0.0001245 |
| MSMEG_4823 | 6 | 60 | 1.027445837 | 11.91323398 | 3.5354309 | 2.46E-05 | 5.39E-05 |
| MSMEG_1710 | 64 | 637 | 13.13293897 | 151.5626252 | 3.5286523 | 0 | 0 |
| MSMEG_1295 | 14 | 139 | 9.70257814 | 111.6978075 | 3.5250889 | 3.76E-07 | 1.02E-06 |
| MSMEG_2710 | 14 | 139 | 4.232400341 | 48.72414646 | 3.5250889 | 3.76E-07 | 1.02E-06 |
| MSMEG_6655 | 18 | 178 | 5.105493406 | 58.54043585 | 3.5193112 | 4.03E-08 | 1.20E-07 |
| MSMEG_5526 | 570 | 5569 | 120.3263077 | 1363.118736 | 3.5018872 | 7.53E-13 | 3.13E-12 |
| MSMEG_6835 | 4 | 39 | 0.631934429 | 7.144097409 | 3.498905 | 6.06E-05 | 0.0001244 |
| MSMEG_1152 | 3 | 29 | 0.513722918 | 5.75806309 | 3.4865213 | 9.05E-05 | 0.0001799 |
| MSMEG_2846 | 3 | 29 | 0.682313102 | 7.647706086 | 3.4865213 | 9.05E-05 | 0.00018 |
| MSMEG_4318 | 27 | 258 | 7.265509847 | 80.49942389 | 3.4698425 | 2.37E-10 | 8.17E-10 |
| MSMEG_6573 | 11 | 105 | 1.453392358 | 16.08607987 | 3.4683167 | 1.92E-06 | 4.85E-06 |
| MSMEG_6325 | 2 | 19 | 0.816248637 | 8.991182423 | 3.4614303 | 0.0001608 | 0.0003057 |
| MSMEG_5626 | 4 | 38 | 0.751533272 | 8.278326528 | 3.4614303 | 6.06E-05 | 0.0001244 |
| MSMEG_1297 | 10 | 95 | 1.621686034 | 17.86327634 | 3.4614303 | 3.26E-06 | 8.07E-06 |
| MSMEG_1742 | 49 | 464 | 9.889166181 | 108.5806183 | 3.4567739 | 4.73E-14 | 2.33E-13 |
| MSMEG_6529 | 3 | 28 | 0.881548528 | 9.540117771 | 3.4358952 | 9.07E-05 | 0.0001802 |
| MSMEG_3950 | 604 | 5614 | 150.4111048 | 1621.011537 | 3.4299114 | 2.67E-13 | 1.19E-12 |
| MSMEG_2154 | 4 | 37 | 1.06854367 | 11.46053109 | 3.4229561 | 6.06E-05 | 0.0001244 |
| MSMEG_1161 | 5 | 46 | 1.365471698 | 14.56603218 | 3.4151366 | 3.92E-05 | 8.32E-05 |
| MSMEG_6859 | 6 | 55 | 1.756072765 | 18.66485762 | 3.4099 | 2.46E-05 | 5.40E-05 |
| MSMEG_4084 | 8 | 73 | 1.522536318 | 16.10909968 | 3.4033273 | 9.19E-06 | 2.14E-05 |
| MSMEG_1228 | 4 | 36 | 0.651551019 | 6.799260369 | 3.3834278 | 6.06E-05 | 0.0001243 |
| MSMEG_4135 | 2 | 18 | 0.798504102 | 8.332789202 | 3.3834278 | 0.0001897 | 0.0003583 |
| MSMEG_2708 | 3 | 27 | 3.443548938 | 35.93515343 | 3.3834278 | 9.10E-05 | 0.0001808 |
| MSMEG_4637 | 2 | 18 | 0.443881434 | 4.632124511 | 3.3834278 | 0.0001897 | 0.0003582 |
| MSMEG_3926 | 14 | 126 | 1.114272246 | 11.62798753 | 3.3834278 | 3.76E-07 | 1.02E-06 |
| MSMEG_6117 | 6 | 54 | 0.979498365 | 10.22155475 | 3.3834278 | 2.46E-05 | 5.38E-05 |
| MSMEG_1658 | 390 | 3457 | 82.80441377 | 851.0576779 | 3.3614773 | 6.66E-14 | 3.22E-13 |
| MSMEG_2401 | 1546 | 13688 | 540.8230256 | 5552.088948 | 3.3598022 | 3.03E-12 | 1.18E-11 |
| MSMEG_3729 | 18 | 159 | 1.91919128 | 19.65683607 | 3.3564607 | 4.03E-08 | 1.19E-07 |
| MSMEG_1778 | 35 | 308 | 22.55423866 | 230.1344199 | 3.3510063 | 2.73E-12 | 1.07E-11 |
| MSMEG_0263 | 4 | 35 | 0.693041296 | 7.031336801 | 3.3427858 | 6.06E-05 | 0.0001243 |
| MSMEG_6147 | 4 | 35 | 0.807278872 | 8.190348361 | 3.3427858 | 6.06E-05 | 0.0001242 |
| MSMEG_4731 | 4 | 35 | 0.508390155 | 5.157935646 | 3.3427858 | 6.06E-05 | 0.0001243 |
| MSMEG_1817 | 364 | 3161 | 119.3763632 | 1202.021376 | 3.3318734 | 0 | 0 |
| MSMEG_6615 | 121 | 1046 | 39.68280205 | 397.7584179 | 3.3253067 | 8.44E-15 | 4.40E-14 |
| MSMEG_6500 | 717 | 6154 | 84.81888012 | 844.1149753 | 3.3149822 | 3.78E-13 | 1.65E-12 |
| MSMEG_4892 | 2 | 17 | 0.890453059 | 8.776082365 | 3.3009656 | 0.0002461 | 0.0004589 |
| MSMEG_0113 | 2 | 17 | 0.511932943 | 5.045482893 | 3.3009656 | 0.0002461 | 0.0004583 |
| MSMEG_5619 | 2 | 17 | 0.374808048 | 3.694014261 | 3.3009656 | 0.0002461 | 0.0004587 |
| MSMEG_5809 | 2 | 17 | 0.489749182 | 4.826845301 | 3.3009656 | 0.0002461 | 0.0004586 |
| MSMEG_0554 | 4 | 34 | 1.01327417 | 9.986576484 | 3.3009656 | 6.06E-05 | 0.0001242 |
| MSMEG_2217 | 4 | 34 | 0.607127085 | 5.983692522 | 3.3009656 | 6.06E-05 | 0.0001242 |
| MSMEG_4387 | 2 | 17 | 0.515525455 | 5.08088979 | 3.3009656 | 0.0002461 | 0.0004585 |
| MSMEG_3924 | 2 | 17 | 0.353184506 | 3.480898053 | 3.3009656 | 0.0002461 | 0.000459 |
| MSMEG_4008 | 7 | 59 | 1.521410182 | 14.86863241 | 3.2887909 | 1.52E-05 | 3.41E-05 |
| MSMEG_4136 | 5 | 42 | 1.883650701 | 18.34638033 | 3.2838921 | 3.92E-05 | 8.32E-05 |
| MSMEG_3011 | 8 | 67 | 1.070490016 | 10.39532739 | 3.279592 | 9.19E-06 | 2.14E-05 |
| MSMEG_1154 | 9 | 75 | 1.678074609 | 16.21439523 | 3.2723964 | 5.50E-06 | 1.32E-05 |
| MSMEG_4427 | 6 | 50 | 0.912576116 | 8.817766352 | 3.2723964 | 2.46E-05 | 5.40E-05 |
| MSMEG_4638 | 4 | 33 | 0.935826463 | 8.951998591 | 3.2578969 | 6.07E-05 | 0.0001242 |
| MSMEG_2702 | 41 | 337 | 8.03188659 | 76.54808783 | 3.2525555 | 2.66E-13 | 1.19E-12 |
| MSMEG_1706 | 181 | 1487 | 31.65878643 | 301.576427 | 3.2518458 | 8.26E-14 | 3.94E-13 |
| MSMEG_2347 | 268 | 2184 | 38.00756202 | 359.1357076 | 3.2401707 | 3.75E-14 | 1.88E-13 |
| MSMEG_3444 | 49 | 396 | 7.19931298 | 67.46226138 | 3.2281495 | 4.73E-14 | 2.34E-13 |
| MSMEG_1705 | 355 | 2850 | 51.13557639 | 476.0037753 | 3.2185737 | 3.45E-13 | 1.51E-12 |
| MSMEG_6845 | 7 | 56 | 1.190362596 | 11.04180298 | 3.2135028 | 1.52E-05 | 3.41E-05 |
| MSMEG_2004 | 4 | 32 | 1.152351017 | 10.68920759 | 3.2135028 | 6.07E-05 | 0.0001242 |
| MSMEG_4417 | 3 | 24 | 1.159932274 | 10.75953132 | 3.2135028 | 9.53E-05 | 0.0001888 |
| MSMEG_2223 | 3 | 24 | 0.759955628 | 7.049348107 | 3.2135028 | 9.53E-05 | 0.0001887 |
| MSMEG_2893 | 4 | 32 | 0.834799743 | 7.743602087 | 3.2135028 | 6.07E-05 | 0.0001242 |
| MSMEG_1478 | 4 | 32 | 1.354145205 | 12.56105039 | 3.2135028 | 6.07E-05 | 0.0001242 |
| MSMEG_1690 | 3 | 24 | 1.044488777 | 9.688677493 | 3.2135028 | 9.53E-05 | 0.0001888 |
| MSMEG_6703 | 2 | 16 | 0.530414277 | 4.920122626 | 3.2135028 | 0.0003557 | 0.000651 |
| MSMEG_6527 | 3 | 24 | 1.091025406 | 10.12035124 | 3.2135028 | 9.53E-05 | 0.0001887 |
| MSMEG_3384 | 6 | 48 | 1.421852465 | 13.18910291 | 3.2135028 | 2.46E-05 | 5.39E-05 |
| MSMEG_0601 | 32 | 254 | 22.82326286 | 210.0546042 | 3.2021874 | 1.39E-11 | 5.15E-11 |
| MSMEG_2094 | 26 | 206 | 4.063876194 | 37.33404751 | 3.1995636 | 4.21E-10 | 1.43E-09 |
| MSMEG_2547 | 8 | 63 | 1.7595779 | 16.06681511 | 3.1907827 | 9.19E-06 | 2.14E-05 |
| MSMEG_5975 | 6 | 47 | 1.231213028 | 11.18279966 | 3.1831291 | 2.46E-05 | 5.39E-05 |
| MSMEG_6735 | 6 | 47 | 0.920196793 | 8.357917075 | 3.1831291 | 2.46E-05 | 5.39E-05 |
| MSMEG_1811 | 5 | 39 | 1.732603239 | 15.6698363 | 3.1769769 | 3.92E-05 | 8.32E-05 |
| MSMEG_5026 | 17 | 132 | 2.580290113 | 23.23080626 | 3.170434 | 7.08E-08 | 2.05E-07 |
| MSMEG_6718 | 4 | 31 | 0.644406819 | 5.790719982 | 3.1676991 | 6.08E-05 | 0.0001243 |
| MSMEG_0271 | 8 | 62 | 2.683557163 | 24.1147791 | 3.1676991 | 9.19E-06 | 2.14E-05 |
| MSMEG_1693 | 8 | 62 | 1.015024212 | 9.121134064 | 3.1676991 | 9.19E-06 | 2.14E-05 |
| MSMEG_6874 | 4 | 31 | 0.586525967 | 5.270595432 | 3.1676991 | 6.08E-05 | 0.0001243 |
| MSMEG_4991 | 99 | 765 | 93.24070971 | 835.4155328 | 3.1634621 | 0 | 0 |
| MSMEG_3367 | 7 | 54 | 2.048751559 | 18.32549657 | 3.1610353 | 1.52E-05 | 3.41E-05 |
| MSMEG_2886 | 3 | 23 | 1.064672135 | 9.464402551 | 3.1521022 | 9.99E-05 | 0.000197 |
| MSMEG_0569 | 6 | 46 | 1.039561944 | 9.241185509 | 3.1521022 | 2.46E-05 | 5.38E-05 |
| MSMEG_4814 | 9 | 69 | 1.197756152 | 10.64745287 | 3.1521022 | 5.50E-06 | 1.32E-05 |
| MSMEG_2535 | 6 | 46 | 1.721774469 | 15.3057135 | 3.1521022 | 2.46E-05 | 5.38E-05 |
| MSMEG_2239 | 3 | 23 | 0.892255595 | 7.931705781 | 3.1521022 | 9.99E-05 | 0.000197 |
| MSMEG_0764 | 68 | 518 | 9.461063749 | 83.56637252 | 3.1428482 | 0 | 0 |
| MSMEG_1709 | 64 | 486 | 13.74734547 | 121.0447274 | 3.1383153 | 0 | 0 |
| MSMEG_6656 | 71 | 538 | 10.36944094 | 91.10663449 | 3.135218 | 0 | 0 |
| MSMEG_3137 | 17 | 128 | 3.375298419 | 29.46754524 | 3.1260399 | 7.08E-08 | 2.05E-07 |
| MSMEG_0342 | 6 | 45 | 1.563029305 | 13.59249302 | 3.1203934 | 2.46E-05 | 5.37E-05 |
| MSMEG_4520 | 4 | 30 | 1.229495855 | 10.69200288 | 3.1203934 | 6.10E-05 | 0.0001247 |
| MSMEG_1155 | 4 | 30 | 1.092377358 | 9.499586203 | 3.1203934 | 6.10E-05 | 0.0001246 |
| MSMEG_3116 | 4 | 30 | 1.175398038 | 10.22155475 | 3.1203934 | 6.10E-05 | 0.0001246 |
| MSMEG_0338 | 12 | 90 | 2.325985562 | 20.2273511 | 3.1203934 | 1.12E-06 | 2.92E-06 |
| MSMEG_0511 | 8 | 58 | 1.54251711 | 12.96697672 | 3.0714838 | 9.19E-06 | 2.14E-05 |
| MSMEG_2238 | 8 | 58 | 1.154614968 | 9.706125995 | 3.0714838 | 9.19E-06 | 2.14E-05 |
| MSMEG_3267 | 62 | 448 | 14.32285344 | 120.0014814 | 3.0666614 | 0 | 0 |
| MSMEG_0346 | 9 | 65 | 1.391918729 | 11.65615893 | 3.0659456 | 5.50E-06 | 1.32E-05 |
| MSMEG_2858 | 5 | 36 | 0.755785775 | 6.3096017 | 3.0614997 | 3.92E-05 | 8.32E-05 |
| MSMEG_1488 | 11 | 79 | 1.400495929 | 11.66237472 | 3.0578519 | 1.92E-06 | 4.84E-06 |
| MSMEG_4551 | 6 | 43 | 0.956126386 | 7.945170905 | 3.054805 | 2.46E-05 | 5.37E-05 |
| MSMEG_5845 | 6 | 43 | 1.417280592 | 11.7772469 | 3.054805 | 2.46E-05 | 5.37E-05 |
| MSMEG_6240 | 20 | 143 | 2.950296279 | 24.459209 | 3.051446 | 1.30E-08 | 3.98E-08 |
| MSMEG_5136 | 328 | 2345 | 159.5739058 | 1322.822621 | 3.051323 | 1.44E-13 | 6.65E-13 |
| MSMEG_2663 | 27 | 192 | 3.425706716 | 28.24609259 | 3.0435778 | 2.37E-10 | 8.18E-10 |
| MSMEG_4195 | 383 | 2697 | 275.8440247 | 2252.249442 | 3.029442 | 1.99E-13 | 9.05E-13 |
| MSMEG_5429 | 4 | 28 | 1.379575161 | 11.19732133 | 3.0208577 | 6.22E-05 | 0.0001268 |
| MSMEG_4440 | 6 | 42 | 1.715074957 | 13.92040531 | 3.0208577 | 2.46E-05 | 5.37E-05 |
| MSMEG_1270 | 3 | 21 | 2.248848286 | 18.25277635 | 3.0208577 | 0.0001255 | 0.000245 |
| MSMEG_6671 | 3 | 21 | 0.633296356 | 5.140149661 | 3.0208577 | 0.0001255 | 0.0002451 |
| MSMEG_1232 | 6 | 42 | 0.90694293 | 7.361201984 | 3.0208577 | 2.46E-05 | 5.37E-05 |
| MSMEG_5097 | 6 | 42 | 1.851992706 | 15.03169817 | 3.0208577 | 2.46E-05 | 5.37E-05 |
| MSMEG_0499 | 14 | 98 | 2.160658157 | 17.5369812 | 3.0208577 | 3.76E-07 | 1.02E-06 |
| MSMEG_3993 | 5 | 35 | 1.407325237 | 11.4225548 | 3.0208577 | 3.92E-05 | 8.32E-05 |
| MSMEG_3673 | 462 | 3226 | 46.17635147 | 373.8632159 | 3.0172844 | 0 | 0 |
| MSMEG_4836 | 9 | 62 | 1.640598998 | 13.10455738 | 2.9977741 | 5.50E-06 | 1.32E-05 |
| MSMEG_2715 | 52 | 358 | 8.642632629 | 68.99164031 | 2.9968788 | 7.62E-14 | 3.66E-13 |
| MSMEG_6140 | 8 | 55 | 1.376344306 | 10.9716142 | 2.9948625 | 9.19E-06 | 2.14E-05 |
| MSMEG_0347 | 7 | 48 | 1.499232191 | 11.92018047 | 2.9911103 | 1.52E-05 | 3.41E-05 |
| MSMEG_1296 | 54 | 370 | 13.22322792 | 105.0548683 | 2.9899967 | 6.86E-14 | 3.31E-13 |
| MSMEG_4757 | 856 | 5846 | 20.35074272 | 161.1521281 | 2.9852699 | 1.14E-12 | 4.63E-12 |
| MSMEG_6755 | 5 | 34 | 1.192571061 | 9.402945391 | 2.9790375 | 3.93E-05 | 8.33E-05 |
| MSMEG_6798 | 9 | 61 | 1.985469658 | 15.60347448 | 2.9743151 | 5.50E-06 | 1.32E-05 |
| MSMEG_0934 | 300 | 2027 | 132.7633326 | 1040.115035 | 2.9698144 | 2.28E-13 | 1.03E-12 |
| MSMEG_2882 | 4 | 27 | 0.789918036 | 6.182391989 | 2.9683903 | 6.36E-05 | 0.0001295 |
| MSMEG_2213 | 4 | 27 | 0.73831535 | 5.778517135 | 2.9683903 | 6.36E-05 | 0.0001295 |
| MSMEG_3447 | 3 | 20 | 1.025056428 | 7.923685856 | 2.9504684 | 0.0001576 | 0.0002998 |
| MSMEG_0516 | 9 | 60 | 2.174873014 | 16.81176769 | 2.9504684 | 5.50E-06 | 1.32E-05 |
| MSMEG_4785 | 9 | 60 | 1.145860305 | 8.857499787 | 2.9504684 | 5.50E-06 | 1.32E-05 |
| MSMEG_4388 | 6 | 40 | 0.811738976 | 6.274742023 | 2.9504684 | 2.46E-05 | 5.36E-05 |
| MSMEG_1446 | 6 | 40 | 2.533185426 | 19.58152252 | 2.9504684 | 2.46E-05 | 5.37E-05 |
| MSMEG_6767 | 88 | 581 | 3.538417737 | 27.08777282 | 2.9364655 | 0 | 0 |
| MSMEG_3346 | 5 | 33 | 1.407325237 | 10.76983739 | 2.9359688 | 3.94E-05 | 8.35E-05 |
| MSMEG_4510 | 12 | 79 | 0.595640897 | 4.546750144 | 2.932321 | 1.12E-06 | 2.92E-06 |
| MSMEG_6023 | 20 | 131 | 3.673118867 | 27.89632652 | 2.9249977 | 1.30E-08 | 3.98E-08 |
| MSMEG_5218 | 58 | 379 | 31.7971484 | 240.9184858 | 2.9215758 | 3.64E-14 | 1.82E-13 |
| MSMEG_1080 | 15 | 98 | 1.352068295 | 10.24245773 | 2.921322 | 2.16E-07 | 5.99E-07 |
| MSMEG_3427 | 6 | 39 | 1.440438771 | 10.85622646 | 2.9139425 | 2.46E-05 | 5.36E-05 |
| MSMEG_4415 | 4 | 26 | 0.792047195 | 5.969461447 | 2.9139425 | 6.62E-05 | 0.0001343 |
| MSMEG_0297 | 4 | 26 | 0.699641689 | 5.273024278 | 2.9139425 | 6.62E-05 | 0.0001346 |
| MSMEG_1082 | 4 | 26 | 1.204301268 | 9.0765172 | 2.9139425 | 6.62E-05 | 0.0001345 |
| MSMEG_1968 | 4 | 26 | 1.239871348 | 9.344599987 | 2.9139425 | 6.62E-05 | 0.0001343 |
| MSMEG_3347 | 4 | 26 | 1.104697404 | 8.325827808 | 2.9139425 | 6.62E-05 | 0.0001346 |
| MSMEG_5983 | 4 | 26 | 1.002899349 | 7.558601354 | 2.9139425 | 6.62E-05 | 0.0001344 |
| MSMEG_4870 | 4 | 26 | 0.537202028 | 4.048757215 | 2.9139425 | 6.62E-05 | 0.0001344 |
| MSMEG_3812 | 4 | 26 | 1.034681371 | 7.798134496 | 2.9139425 | 6.62E-05 | 0.0001345 |
| MSMEG_6167 | 4 | 26 | 1.546576365 | 11.65615893 | 2.9139425 | 6.62E-05 | 0.0001344 |
| MSMEG_6759 | 229 | 1488 | 33.24680714 | 250.4886936 | 2.9134578 | 2.93E-13 | 1.30E-12 |
| MSMEG_0444 | 7 | 45 | 1.508025341 | 11.24071271 | 2.8980009 | 1.52E-05 | 3.41E-05 |
| MSMEG_6784 | 15 | 96 | 3.095324888 | 22.96978597 | 2.8915747 | 2.16E-07 | 5.98E-07 |
| MSMEG_2680 | 10 | 64 | 1.56636199 | 11.62365857 | 2.8915747 | 3.26E-06 | 8.07E-06 |
| MSMEG_0463 | 16 | 102 | 1.221827482 | 9.031519274 | 2.8859281 | 1.24E-07 | 3.51E-07 |
| MSMEG_1691 | 8 | 51 | 0.65445325 | 4.837595513 | 2.8859281 | 9.19E-06 | 2.14E-05 |
| MSMEG_2927 | 785 | 5001 | 266.9813251 | 1972.144881 | 2.8849548 | 0 | 0 |
| MSMEG_1712 | 282 | 1790 | 62.96775201 | 463.439286 | 2.8796953 | 6.98E-13 | 2.92E-12 |
| MSMEG_4874 | 6 | 38 | 1.214254171 | 8.91687513 | 2.8764678 | 2.47E-05 | 5.37E-05 |
| MSMEG_1171 | 3 | 19 | 1.608665197 | 11.81323238 | 2.8764678 | 0.0002181 | 0.0004089 |
| MSMEG_4023 | 13 | 82 | 1.925425213 | 14.08211509 | 2.870615 | 6.50E-07 | 1.73E-06 |
| MSMEG_2711 | 75 | 473 | 7.211620813 | 52.73555191 | 2.8703804 | 0 | 0 |
| MSMEG_5739 | 17 | 107 | 1.06376526 | 7.763389826 | 2.8675069 | 7.08E-08 | 2.05E-07 |
| MSMEG_4587 | 7 | 44 | 2.056946566 | 14.99161364 | 2.8655795 | 1.52E-05 | 3.41E-05 |
| MSMEG_0630 | 7 | 44 | 1.918793438 | 13.98471422 | 2.8655795 | 1.52E-05 | 3.41E-05 |
| MSMEG_3400 | 15 | 94 | 1.503322865 | 10.92344414 | 2.861201 | 2.16E-07 | 6.00E-07 |
| MSMEG_0827 | 8 | 50 | 1.015024212 | 7.355753278 | 2.8573589 | 9.19E-06 | 2.13E-05 |
| MSMEG_6872 | 4 | 25 | 0.839570027 | 6.084258783 | 2.8573589 | 7.11E-05 | 0.0001439 |
| MSMEG_0143 | 10 | 62 | 2.473480719 | 17.78160479 | 2.845771 | 3.26E-06 | 8.07E-06 |
| MSMEG_4378 | 5 | 31 | 1.70842738 | 12.28171308 | 2.845771 | 4.01E-05 | 8.48E-05 |
| MSMEG_1505 | 11 | 68 | 2.757973211 | 19.7686497 | 2.841534 | 1.92E-06 | 4.84E-06 |
| MSMEG_4091 | 11 | 68 | 1.815923934 | 13.01621205 | 2.841534 | 1.92E-06 | 4.85E-06 |
| MSMEG_1208 | 8 | 49 | 1.698552077 | 12.06301019 | 2.8282126 | 9.19E-06 | 2.13E-05 |
| MSMEG_2549 | 16 | 98 | 2.653268708 | 18.84334774 | 2.8282126 | 1.24E-07 | 3.50E-07 |
| MSMEG_4035 | 14 | 85 | 3.781151775 | 26.61863217 | 2.8155388 | 3.76E-07 | 1.02E-06 |
| MSMEG_3386 | 16 | 97 | 2.629525811 | 18.48416874 | 2.8134156 | 1.24E-07 | 3.51E-07 |
| MSMEG_3272 | 18 | 109 | 8.586511638 | 60.28947339 | 2.8117621 | 4.03E-08 | 1.20E-07 |
| MSMEG_1391 | 5 | 30 | 1.224372956 | 8.517962296 | 2.7984653 | 4.08E-05 | 8.63E-05 |
| MSMEG_0506 | 8 | 48 | 2.06210182 | 14.34604176 | 2.7984653 | 9.19E-06 | 2.13E-05 |
| MSMEG_0279 | 33 | 198 | 4.73487979 | 32.94055731 | 2.7984653 | 7.98E-12 | 3.01E-11 |
| MSMEG_2522 | 4 | 24 | 0.743922809 | 5.175470762 | 2.7984653 | 8.04E-05 | 0.000162 |
| MSMEG_3322 | 5 | 30 | 1.386082591 | 9.642976184 | 2.7984653 | 4.08E-05 | 8.63E-05 |
| MSMEG_6657 | 9 | 54 | 1.749104223 | 12.16851757 | 2.7984653 | 5.50E-06 | 1.32E-05 |
| MSMEG_6455 | 4 | 24 | 1.506920561 | 10.4836459 | 2.7984653 | 8.04E-05 | 0.0001621 |
| MSMEG_0486 | 14 | 84 | 2.989747915 | 20.79967537 | 2.7984653 | 3.76E-07 | 1.02E-06 |
| MSMEG_1717 | 4 | 24 | 0.629228071 | 4.37753951 | 2.7984653 | 8.04E-05 | 0.000162 |
| MSMEG_4740 | 5 | 30 | 0.885088884 | 6.157563105 | 2.7984653 | 4.08E-05 | 8.63E-05 |
| MSMEG_0575 | 4 | 24 | 2.114025247 | 14.70727303 | 2.7984653 | 8.04E-05 | 0.0001621 |
| MSMEG_2720 | 151 | 905 | 31.51369028 | 218.9987465 | 2.796872 | 0 | 0 |
| MSMEG_1761 | 137 | 820 | 22.92561662 | 159.1054461 | 2.7949508 | 1.42E-13 | 6.55E-13 |
| MSMEG_4532 | 19 | 113 | 4.880367726 | 33.65488599 | 2.7857542 | 2.29E-08 | 6.89E-08 |
| MSMEG_4246 | 106 | 630 | 24.25860436 | 167.1749609 | 2.7847903 | 0 | 0 |
| MSMEG_4127 | 12 | 71 | 1.883650701 | 12.92254964 | 2.7782874 | 1.12E-06 | 2.92E-06 |
| MSMEG_6542 | 240 | 1419 | 48.17205072 | 330.2455874 | 2.777271 | 0 | 0 |
| MSMEG_2584 | 119 | 700 | 14.40201467 | 98.23020769 | 2.7698961 | 0 | 0 |
| MSMEG_3951 | 8 | 47 | 1.871652926 | 12.74981617 | 2.7680916 | 9.19E-06 | 2.13E-05 |
| MSMEG_1732 | 8 | 47 | 1.530466195 | 10.42563093 | 2.7680916 | 9.19E-06 | 2.13E-05 |
| MSMEG_3270 | 70 | 411 | 13.60414395 | 92.61593925 | 2.7672143 | 0 | 0 |
| MSMEG_4588 | 5 | 29 | 1.236740359 | 8.317202241 | 2.7495557 | 4.23E-05 | 8.91E-05 |
| MSMEG_2817 | 5 | 29 | 3.710221078 | 24.95160672 | 2.7495557 | 4.23E-05 | 8.91E-05 |
| MSMEG_2870 | 10 | 58 | 1.898252645 | 12.76593832 | 2.7495557 | 3.26E-06 | 8.06E-06 |
| MSMEG_3928 | 9 | 52 | 1.53401716 | 10.27689186 | 2.7440175 | 5.50E-06 | 1.32E-05 |
| MSMEG_0772 | 4 | 23 | 0.767231095 | 5.115225399 | 2.7370647 | 9.77E-05 | 0.0001929 |
| MSMEG_0295 | 4 | 23 | 0.81852231 | 5.457190329 | 2.7370647 | 9.77E-05 | 0.000193 |
| MSMEG_2186 | 4 | 23 | 1.412738026 | 9.418900615 | 2.7370647 | 9.77E-05 | 0.0001929 |
| MSMEG_1780 | 17 | 97 | 3.046001012 | 20.15225226 | 2.7259528 | 7.08E-08 | 2.05E-07 |
| MSMEG_2231 | 10 | 57 | 1.352898294 | 8.941507382 | 2.7244647 | 3.26E-06 | 8.06E-06 |
| MSMEG_4222 | 300 | 1707 | 57.09511193 | 376.6881254 | 2.7219314 | 2.28E-13 | 1.03E-12 |
| MSMEG_3418 | 235 | 1333 | 120.7248858 | 794.0170448 | 2.7174469 | 2.27E-13 | 1.02E-12 |
| MSMEG_4845 | 3 | 17 | 0.819283019 | 5.383098849 | 2.7160031 | 0.0005436 | 0.0009739 |
| MSMEG_1088 | 6 | 34 | 0.943842107 | 6.201514305 | 2.7160031 | 2.51E-05 | 5.46E-05 |
| MSMEG_4385 | 12 | 68 | 1.594120304 | 10.47416702 | 2.7160031 | 1.12E-06 | 2.91E-06 |
| MSMEG_5643 | 6 | 34 | 1.579836072 | 10.38031247 | 2.7160031 | 2.51E-05 | 5.46E-05 |
| MSMEG_6515 | 1258 | 7103 | 155.5819372 | 1018.570475 | 2.7107993 | 1.61E-12 | 6.45E-12 |
| MSMEG_2187 | 8 | 45 | 0.871957001 | 5.687066815 | 2.7053559 | 9.19E-06 | 2.13E-05 |
| MSMEG_1751 | 8 | 45 | 1.713408218 | 11.17516919 | 2.7053559 | 9.19E-06 | 2.13E-05 |
| MSMEG_0517 | 5 | 28 | 1.188711608 | 7.718541886 | 2.6989296 | 4.50E-05 | 9.44E-05 |
| MSMEG_6808 | 5 | 28 | 0.949126322 | 6.162866777 | 2.6989296 | 4.50E-05 | 9.45E-05 |
| MSMEG_2342 | 5 | 28 | 0.88722678 | 5.760940683 | 2.6989296 | 4.50E-05 | 9.44E-05 |
| MSMEG_3909 | 12 | 67 | 4.924852113 | 31.88287563 | 2.6946294 | 1.12E-06 | 2.91E-06 |
| MSMEG_6822 | 609 | 3395 | 147.1664072 | 951.265855 | 2.6924002 | 3.77E-13 | 1.64E-12 |
| MSMEG_5815 | 9 | 50 | 1.354838926 | 8.727420385 | 2.6874339 | 5.50E-06 | 1.32E-05 |
| MSMEG_1536 | 18 | 100 | 1.091025406 | 7.028021696 | 2.6874339 | 4.03E-08 | 1.20E-07 |
| MSMEG_4077 | 9 | 50 | 2.613286151 | 16.83391758 | 2.6874339 | 5.50E-06 | 1.32E-05 |
| MSMEG_5733 | 649 | 3587 | 162.7204194 | 1042.796271 | 2.6799901 | 2.10E-12 | 8.37E-12 |
| MSMEG_3266 | 131 | 723 | 20.13299463 | 128.8386347 | 2.6779316 | 0 | 0 |
| MSMEG_0627 | 6 | 33 | 0.75604505 | 4.821488092 | 2.6729344 | 2.55E-05 | 5.55E-05 |
| MSMEG_6847 | 8 | 44 | 1.526490958 | 9.734814052 | 2.6729344 | 9.20E-06 | 2.13E-05 |
| MSMEG_6537 | 4 | 22 | 1.530466195 | 9.76016513 | 2.6729344 | 0.0001297 | 0.0002526 |
| MSMEG_4877 | 4 | 22 | 0.816248637 | 5.205421403 | 2.6729344 | 0.0001297 | 0.0002525 |
| MSMEG_5078 | 1114 | 6119 | 202.0668848 | 1286.948427 | 2.6710494 | 0 | 0 |
| MSMEG_3584 | 15 | 81 | 1.147849646 | 7.187030687 | 2.6464622 | 2.16E-07 | 5.99E-07 |
| MSMEG_2880 | 5 | 27 | 1.407325237 | 8.811685133 | 2.6464622 | 4.99E-05 | 0.0001041 |
| MSMEG_2569 | 5 | 27 | 1.130190421 | 7.076460984 | 2.6464622 | 4.99E-05 | 0.0001041 |
| MSMEG_5421 | 28 | 151 | 2.105370077 | 13.16491614 | 2.6445526 | 1.34E-10 | 4.67E-10 |
| MSMEG_5661 | 181 | 975 | 20.80859202 | 129.9689083 | 2.6429153 | 8.26E-14 | 3.95E-13 |
| MSMEG_0275 | 8 | 43 | 2.895069058 | 18.04297432 | 2.6397675 | 9.21E-06 | 2.13E-05 |
| MSMEG_2894 | 8 | 43 | 1.086319813 | 6.770284265 | 2.6397675 | 9.21E-06 | 2.13E-05 |
| MSMEG_0817 | 70 | 375 | 16.69599485 | 103.7089565 | 2.6349665 | 0 | 0 |
| MSMEG_0568 | 6 | 32 | 1.25935504 | 7.787851242 | 2.6285403 | 2.63E-05 | 5.71E-05 |
| MSMEG_0527 | 9 | 48 | 2.665973371 | 16.48637864 | 2.6285403 | 5.50E-06 | 1.32E-05 |
| MSMEG_2465 | 31 | 164 | 5.595414491 | 34.32299303 | 2.6168585 | 2.43E-11 | 8.86E-11 |
| MSMEG_1550 | 7 | 37 | 4.510847732 | 27.64601798 | 2.6156012 | 1.54E-05 | 3.47E-05 |
| MSMEG_5387 | 8 | 42 | 2.019584257 | 12.2939662 | 2.6058202 | 9.23E-06 | 2.13E-05 |
| MSMEG_6622 | 8 | 42 | 1.480350173 | 9.011446257 | 2.6058202 | 9.23E-06 | 2.13E-05 |
| MSMEG_2524 | 4 | 21 | 1.21929257 | 7.422290797 | 2.6058202 | 0.0001885 | 0.0003562 |
| MSMEG_3790 | 8 | 42 | 2.322921023 | 14.14049077 | 2.6058202 | 9.23E-06 | 2.13E-05 |
| MSMEG_6739 | 250 | 1307 | 123.2590224 | 747.1796457 | 2.5997619 | 1.47E-13 | 6.74E-13 |
| MSMEG_6390 | 9 | 47 | 1.616531531 | 9.78836743 | 2.5981666 | 5.51E-06 | 1.32E-05 |
| MSMEG_5147 | 18 | 94 | 4.407742641 | 26.68961519 | 2.5981666 | 4.03E-08 | 1.20E-07 |
| MSMEG_6935 | 1065 | 5547 | 197.0716168 | 1190.154581 | 2.5943571 | 0 | 0 |
| MSMEG_1875 | 231 | 1200 | 32.07903434 | 193.2240974 | 2.5905724 | 0 | 0 |
| MSMEG_3547 | 6 | 31 | 1.5741938 | 9.430601113 | 2.5827366 | 2.77E-05 | 6.01E-05 |
| MSMEG_4866 | 6 | 31 | 0.781514653 | 4.681858709 | 2.5827366 | 2.77E-05 | 6.01E-05 |
| MSMEG_0160 | 10 | 51 | 1.378281001 | 8.150395442 | 2.564 | 3.26E-06 | 8.06E-06 |
| MSMEG_2169 | 12 | 61 | 1.752581567 | 10.32993439 | 2.5592776 | 1.12E-06 | 2.91E-06 |
| MSMEG_6758 | 92 | 467 | 24.84756881 | 146.2458968 | 2.5572195 | 0 | 0 |
| MSMEG_3420 | 15 | 76 | 4.287687394 | 25.18930484 | 2.5545397 | 2.16E-07 | 5.99E-07 |
| MSMEG_1181 | 16 | 81 | 2.647292877 | 15.53952581 | 2.5533528 | 1.24E-07 | 3.51E-07 |
| MSMEG_4036 | 19 | 96 | 2.705010019 | 15.84737171 | 2.5505377 | 2.29E-08 | 6.89E-08 |
| MSMEG_5730 | 20 | 101 | 2.820052873 | 16.5127484 | 2.5497861 | 1.30E-08 | 3.98E-08 |
| MSMEG_3421 | 119 | 596 | 18.40425875 | 106.8780111 | 2.5378535 | 0 | 0 |
| MSMEG_0276 | 24 | 120 | 3.33919897 | 19.35900522 | 2.5354309 | 1.32E-09 | 4.37E-09 |
| MSMEG_0646 | 11 | 55 | 1.516109101 | 8.789642144 | 2.5354309 | 1.92E-06 | 4.84E-06 |
| MSMEG_4542 | 4 | 20 | 0.938816324 | 5.442787409 | 2.5354309 | 0.000296 | 0.0005467 |
| MSMEG_4730 | 6 | 30 | 0.966610228 | 5.603922563 | 2.5354309 | 3.03E-05 | 6.53E-05 |
| MSMEG_3445 | 4 | 20 | 0.869377247 | 5.040214376 | 2.5354309 | 0.000296 | 0.0005463 |
| MSMEG_4316 | 5 | 25 | 1.953786632 | 11.32707752 | 2.5354309 | 7.55E-05 | 0.0001524 |
| MSMEG_1266 | 4 | 20 | 1.277606563 | 7.406923735 | 2.5354309 | 0.000296 | 0.0005464 |
| MSMEG_2860 | 9 | 45 | 1.332986686 | 7.727989986 | 2.5354309 | 5.52E-06 | 1.32E-05 |
| MSMEG_1392 | 6 | 30 | 1.141902239 | 6.62017795 | 2.5354309 | 3.03E-05 | 6.53E-05 |
| MSMEG_6118 | 4 | 20 | 1.10055996 | 6.380496102 | 2.5354309 | 0.000296 | 0.0005461 |
| MSMEG_0722 | 4 | 20 | 1.749104223 | 10.1404313 | 2.5354309 | 0.000296 | 0.0005466 |
| MSMEG_3268 | 63 | 310 | 15.90422602 | 90.74117909 | 2.5123472 | 0 | 0 |
| MSMEG_3010 | 8 | 39 | 1.786319206 | 10.09728053 | 2.498905 | 9.45E-06 | 2.18E-05 |
| MSMEG_2603 | 115 | 555 | 21.06776408 | 117.8919969 | 2.4843567 | 0 | 0 |
| MSMEG_6504 | 17 | 82 | 2.89758797 | 16.20586794 | 2.4835919 | 7.08E-08 | 2.05E-07 |
| MSMEG_1448 | 11 | 53 | 1.616172302 | 9.029040033 | 2.4819916 | 1.92E-06 | 4.84E-06 |
| MSMEG_0427 | 22 | 106 | 1.879270118 | 10.49888376 | 2.4819916 | 4.16E-09 | 1.32E-08 |
| MSMEG_2171 | 11 | 53 | 1.985469658 | 11.09218677 | 2.4819916 | 1.92E-06 | 4.84E-06 |
| MSMEG_3265 | 115 | 554 | 23.27320494 | 129.9986532 | 2.4817549 | 0 | 0 |
| MSMEG_2884 | 21 | 101 | 2.406723751 | 13.42143825 | 2.4793968 | 7.36E-09 | 2.29E-08 |
| MSMEG_1427 | 10 | 48 | 1.399283378 | 7.787851242 | 2.4765372 | 3.27E-06 | 8.09E-06 |
| MSMEG_0116 | 5 | 24 | 1.380871755 | 7.685379515 | 2.4765372 | 0.0001054 | 0.0002072 |
| MSMEG_0532 | 5 | 24 | 1.783067411 | 9.923839568 | 2.4765372 | 0.0001054 | 0.0002072 |
| MSMEG_2466 | 22 | 105 | 4.040430754 | 22.35965103 | 2.4683167 | 4.16E-09 | 1.32E-08 |
| MSMEG_5188 | 48 | 229 | 15.46576365 | 85.55321779 | 2.467744 | 1.15E-13 | 5.38E-13 |
| MSMEG_1704 | 580 | 2759 | 116.4157892 | 642.1054091 | 2.4635234 | 4.09E-14 | 2.03E-13 |
| MSMEG_0142 | 4 | 19 | 1.605735024 | 8.84378599 | 2.4614303 | 0.0004909 | 0.0008839 |
| MSMEG_1733 | 4 | 19 | 0.979498365 | 5.394709454 | 2.4614303 | 0.0004909 | 0.0008842 |
| MSMEG_3830 | 8 | 38 | 2.418514481 | 13.32027026 | 2.4614303 | 9.66E-06 | 2.23E-05 |
| MSMEG_2509 | 12 | 57 | 1.817625831 | 10.01080105 | 2.4614303 | 1.12E-06 | 2.91E-06 |
| MSMEG_4003 | 38 | 180 | 8.778523079 | 48.21488092 | 2.4574283 | 6.64E-13 | 2.78E-12 |
| MSMEG_5473 | 151 | 712 | 18.21480949 | 99.58602881 | 2.4508315 | 0 | 0 |
| MSMEG_4462 | 17 | 80 | 2.51786374 | 13.73864886 | 2.447968 | 7.08E-08 | 2.05E-07 |
| MSMEG_0497 | 23 | 108 | 2.237926727 | 12.18463481 | 2.4448283 | 2.35E-09 | 7.61E-09 |
| MSMEG_1810 | 69 | 323 | 72.41291481 | 393.0431174 | 2.4403687 | 0 | 0 |
| MSMEG_3910 | 6 | 28 | 2.60813174 | 14.11260025 | 2.4358952 | 4.34E-05 | 9.12E-05 |
| MSMEG_4389 | 9 | 42 | 1.446742661 | 7.82832421 | 2.4358952 | 5.64E-06 | 1.35E-05 |
| MSMEG_4104 | 9 | 42 | 1.577950826 | 8.538291561 | 2.4358952 | 5.64E-06 | 1.35E-05 |
| MSMEG_4418 | 6 | 28 | 2.671359176 | 14.4547239 | 2.4358952 | 4.34E-05 | 9.13E-05 |
| MSMEG_5328 | 1296 | 6043 | 952.0724104 | 5147.404615 | 2.434702 | 4.02E-12 | 1.55E-11 |
| MSMEG_0446 | 11 | 51 | 1.803763729 | 9.696787435 | 2.4264965 | 1.92E-06 | 4.85E-06 |
| MSMEG_2594 | 1870 | 8659 | 228.1970858 | 1225.199926 | 2.4246649 | 4.99E-12 | 1.91E-11 |
| MSMEG_6879 | 13 | 60 | 3.141483242 | 16.81176769 | 2.4199536 | 6.51E-07 | 1.73E-06 |
| MSMEG_3271 | 103 | 474 | 13.02345072 | 69.4924979 | 2.4157455 | 0 | 0 |
| MSMEG_4815 | 5 | 23 | 1.228467849 | 6.552278689 | 2.4151366 | 0.0001592 | 0.0003028 |
| MSMEG_5362 | 127 | 583 | 36.5871448 | 194.7440007 | 2.4121701 | 0 | 0 |
| MSMEG_3995 | 12 | 55 | 2.008083208 | 10.67170675 | 2.4099 | 1.12E-06 | 2.91E-06 |
| MSMEG_6765 | 7 | 32 | 1.830023635 | 9.700170586 | 2.4061478 | 2.05E-05 | 4.55E-05 |
| MSMEG_2107 | 153 | 698 | 23.07955592 | 122.0849627 | 2.4031981 | 0 | 0 |
| MSMEG_5390 | 293 | 1329 | 160.6304221 | 844.8038724 | 2.3948713 | 5.91E-13 | 2.51E-12 |
| MSMEG_0336 | 17 | 77 | 4.712680811 | 24.75030554 | 2.3928265 | 7.08E-08 | 2.05E-07 |
| MSMEG_0340 | 8 | 36 | 2.341430354 | 12.21699771 | 2.3834278 | 1.07E-05 | 2.46E-05 |
| MSMEG_4559 | 52 | 234 | 11.23542242 | 58.62362286 | 2.3834278 | 7.62E-14 | 3.65E-13 |
| MSMEG_0357 | 181 | 810 | 24.9001691 | 129.2050461 | 2.375435 | 8.26E-14 | 3.94E-13 |
| MSMEG_2399 | 340 | 1517 | 109.5491592 | 566.7433685 | 2.3711172 | 8.97E-14 | 4.26E-13 |
| MSMEG_0337 | 27 | 120 | 3.390571262 | 17.47274317 | 2.3655059 | 2.37E-10 | 8.18E-10 |
| MSMEG_0103 | 9 | 40 | 1.65290349 | 8.517962296 | 2.3655059 | 5.94E-06 | 1.42E-05 |
| MSMEG_1156 | 9 | 40 | 2.233653365 | 11.51075986 | 2.3655059 | 5.94E-06 | 1.42E-05 |
| MSMEG_0490 | 9 | 40 | 2.533185426 | 13.05434835 | 2.3655059 | 5.94E-06 | 1.42E-05 |
| MSMEG_5960 | 14 | 62 | 2.431378919 | 12.48495656 | 2.3603441 | 3.76E-07 | 1.02E-06 |
| MSMEG_2875 | 7 | 31 | 1.572589117 | 8.075132451 | 2.3603441 | 2.48E-05 | 5.39E-05 |
| MSMEG_5350 | 12 | 53 | 1.859807021 | 9.524303833 | 2.3564607 | 1.13E-06 | 2.92E-06 |
| MSMEG_5801 | 12 | 53 | 1.958996729 | 10.0322667 | 2.3564607 | 1.13E-06 | 2.92E-06 |
| MSMEG_4786 | 5 | 22 | 0.786535089 | 4.01274455 | 2.3510063 | 0.0002553 | 0.0004744 |
| MSMEG_2548 | 10 | 44 | 2.836385226 | 14.47066954 | 2.3510063 | 3.39E-06 | 8.36E-06 |
| MSMEG_6732 | 5 | 22 | 1.864527344 | 9.512445203 | 2.3510063 | 0.0002553 | 0.0004745 |
| MSMEG_0531 | 20 | 88 | 3.636751354 | 18.55397728 | 2.3510063 | 1.30E-08 | 3.97E-08 |
| MSMEG_5594 | 18 | 79 | 2.435216929 | 12.39261549 | 2.3473585 | 4.03E-08 | 1.19E-07 |
| MSMEG_4876 | 8 | 35 | 1.277606563 | 6.481058268 | 2.3427858 | 1.19E-05 | 2.73E-05 |
| MSMEG_2911 | 19 | 83 | 3.101744821 | 15.71090823 | 2.3406147 | 2.29E-08 | 6.88E-08 |
| MSMEG_4011 | 11 | 48 | 1.849167393 | 9.356114192 | 2.3390336 | 1.95E-06 | 4.92E-06 |
| MSMEG_5674 | 23 | 100 | 3.875308897 | 19.53661077 | 2.333797 | 2.35E-09 | 7.60E-09 |
| MSMEG_6885 | 6 | 26 | 1.602815506 | 8.05334617 | 2.32898 | 8.52E-05 | 0.0001706 |
| MSMEG_4889 | 12 | 52 | 1.998976254 | 10.04385577 | 2.32898 | 1.13E-06 | 2.93E-06 |
| MSMEG_0669 | 21 | 91 | 9.12846109 | 45.86595082 | 2.32898 | 7.36E-09 | 2.29E-08 |
| MSMEG_1406 | 6 | 26 | 1.552022057 | 7.798134496 | 2.32898 | 8.52E-05 | 0.0001707 |
| MSMEG_3555 | 9 | 39 | 2.33625935 | 11.73853461 | 2.32898 | 6.29E-06 | 1.49E-05 |
| MSMEG_5468 | 379 | 1641 | 72.31750913 | 363.064315 | 2.3278082 | 3.42E-14 | 1.72E-13 |
| MSMEG_6708 | 10 | 43 | 2.432529051 | 12.12822446 | 2.3178394 | 3.49E-06 | 8.58E-06 |
| MSMEG_1153 | 10 | 43 | 1.573070179 | 7.843091621 | 2.3178394 | 3.49E-06 | 8.59E-06 |
| MSMEG_3977 | 10 | 43 | 2.577627275 | 12.85166241 | 2.3178394 | 3.49E-06 | 8.58E-06 |
| MSMEG_2073 | 24 | 103 | 4.396750764 | 21.87905527 | 2.3150408 | 1.32E-09 | 4.36E-09 |
| MSMEG_1508 | 14 | 60 | 2.165206911 | 10.75953132 | 2.3130384 | 3.77E-07 | 1.02E-06 |
| MSMEG_4859 | 7 | 30 | 1.911660377 | 9.499586203 | 2.3130384 | 3.23E-05 | 6.94E-05 |
| MSMEG_1118 | 14 | 60 | 2.008736881 | 9.981987065 | 2.3130384 | 3.77E-07 | 1.02E-06 |
| MSMEG_4228 | 141 | 600 | 18.2042095 | 89.82034055 | 2.3027701 | 1.02E-14 | 5.31E-14 |
| MSMEG_2216 | 12 | 51 | 1.780906117 | 8.776082365 | 2.3009656 | 1.14E-06 | 2.95E-06 |
| MSMEG_5551 | 12 | 51 | 3.470663497 | 17.10299516 | 2.3009656 | 1.14E-06 | 2.95E-06 |
| MSMEG_2184 | 8 | 34 | 1.143383305 | 5.634449768 | 2.3009656 | 1.41E-05 | 3.19E-05 |
| MSMEG_0328 | 12 | 51 | 5.876990188 | 28.9610718 | 2.3009656 | 1.14E-06 | 2.95E-06 |
| MSMEG_3587 | 12 | 51 | 1.836559434 | 9.050334939 | 2.3009656 | 1.14E-06 | 2.95E-06 |
| MSMEG_1158 | 12 | 51 | 1.851992706 | 9.126388174 | 2.3009656 | 1.14E-06 | 2.95E-06 |
| MSMEG_5747 | 8 | 34 | 3.12605861 | 15.40482543 | 2.3009656 | 1.41E-05 | 3.19E-05 |
| MSMEG_6840 | 8 | 34 | 1.998976254 | 9.850704696 | 2.3009656 | 1.41E-05 | 3.19E-05 |
| MSMEG_0474 | 14 | 59 | 1.725626313 | 8.432210997 | 2.2887909 | 3.77E-07 | 1.02E-06 |
| MSMEG_5986 | 14 | 59 | 1.725626313 | 8.432210997 | 2.2887909 | 3.77E-07 | 1.02E-06 |
| MSMEG_5814 | 5 | 21 | 1.355394416 | 6.600634989 | 2.2838921 | 0.0004258 | 0.0007704 |
| MSMEG_0483 | 16 | 67 | 2.566371261 | 12.46077454 | 2.279592 | 1.24E-07 | 3.50E-07 |
| MSMEG_5363 | 80 | 334 | 47.0159215 | 227.5999525 | 2.275279 | 0 | 0 |
| MSMEG_5508 | 18 | 75 | 2.504399228 | 12.09937826 | 2.2723964 | 4.03E-08 | 1.19E-07 |
| MSMEG_4812 | 7 | 29 | 1.190362596 | 5.718076541 | 2.2641288 | 4.55E-05 | 9.55E-05 |
| MSMEG_5660 | 151 | 622 | 17.03966049 | 81.38513898 | 2.2558688 | 0 | 0 |
| MSMEG_3075 | 94 | 387 | 42.89107746 | 204.7485347 | 2.2551037 | 0 | 0 |
| MSMEG_3730 | 130 | 535 | 20.62658543 | 98.42569823 | 2.25453 | 0 | 0 |
| MSMEG_6406 | 178 | 732 | 24.35065767 | 116.110771 | 2.2534692 | 4.91E-14 | 2.42E-13 |
| MSMEG_3957 | 9 | 37 | 1.252199614 | 5.969026609 | 2.2530311 | 7.97E-06 | 1.87E-05 |
| MSMEG_2681 | 10 | 41 | 2.369754108 | 11.26569207 | 2.2491267 | 3.97E-06 | 9.69E-06 |
| MSMEG_0326 | 47 | 192 | 6.441663685 | 30.51210375 | 2.2438764 | 1.44E-14 | 7.44E-14 |
| MSMEG_3583 | 12 | 49 | 2.631488144 | 12.45910903 | 2.2432501 | 1.18E-06 | 3.05E-06 |
| MSMEG_5926 | 13 | 53 | 1.917692581 | 9.065301238 | 2.2409835 | 6.67E-07 | 1.77E-06 |
| MSMEG_2604 | 26 | 106 | 14.14830971 | 66.88177802 | 2.2409835 | 4.21E-10 | 1.43E-09 |
| MSMEG_1793 | 71 | 289 | 27.74377017 | 130.9410161 | 2.2386813 | 0 | 0 |
| MSMEG_5685 | 221 | 890 | 58.61077759 | 273.681821 | 2.2232617 | 1.07E-13 | 5.03E-13 |
| MSMEG_3955 | 42 | 169 | 9.210208503 | 42.97121277 | 2.2220648 | 1.88E-13 | 8.55E-13 |
| MSMEG_4103 | 12 | 48 | 2.428508342 | 11.26342122 | 2.2135028 | 1.22E-06 | 3.16E-06 |
| MSMEG_0376 | 20 | 80 | 4.209878358 | 19.525415 | 2.2135028 | 1.30E-08 | 3.97E-08 |
| MSMEG_0341 | 14 | 56 | 3.033844492 | 14.07097016 | 2.2135028 | 3.84E-07 | 1.04E-06 |
| MSMEG_6632 | 10 | 40 | 1.362938355 | 6.321307826 | 2.2135028 | 4.50E-06 | 1.09E-05 |
| MSMEG_2879 | 9 | 36 | 1.721774469 | 7.985589652 | 2.2135028 | 9.84E-06 | 2.26E-05 |
| MSMEG_0551 | 9 | 36 | 2.533185426 | 11.74891351 | 2.2135028 | 9.84E-06 | 2.26E-05 |
| MSMEG_5311 | 6 | 24 | 1.695285631 | 7.862734427 | 2.2135028 | 0.0002154 | 0.0004042 |
| MSMEG_2034 | 7 | 28 | 1.416629866 | 6.570329043 | 2.2135028 | 6.88E-05 | 0.0001392 |
| MSMEG_0461 | 8 | 32 | 2.866824482 | 13.29633139 | 2.2135028 | 2.44E-05 | 5.36E-05 |
| MSMEG_2485 | 15 | 60 | 2.055850112 | 9.53503242 | 2.2135028 | 2.19E-07 | 6.05E-07 |
| MSMEG_6846 | 6 | 24 | 2.592789789 | 12.02535853 | 2.2135028 | 0.0002154 | 0.0004041 |
| MSMEG_0206 | 95 | 378 | 9.801862146 | 45.22176612 | 2.2058896 | 0 | 0 |
| MSMEG_1444 | 94 | 370 | 88.53158295 | 404.0571858 | 2.1902954 | 0 | 0 |
| MSMEG_4219 | 674 | 2646 | 230.2960108 | 1048.303639 | 2.1864953 | 0 | 0 |
| MSMEG_4373 | 12 | 47 | 2.958216537 | 13.43437006 | 2.1831291 | 1.30E-06 | 3.36E-06 |
| MSMEG_0293 | 11 | 43 | 1.914896092 | 8.679440254 | 2.1803359 | 2.54E-06 | 6.35E-06 |
| MSMEG_2256 | 10 | 39 | 2.092945224 | 9.464402551 | 2.1769769 | 5.43E-06 | 1.31E-05 |
| MSMEG_2534 | 28 | 109 | 7.142175575 | 32.23812119 | 2.1743322 | 1.34E-10 | 4.67E-10 |
| MSMEG_2973 | 27 | 105 | 6.217818772 | 28.03717997 | 2.1728608 | 2.37E-10 | 8.17E-10 |
| MSMEG_2602 | 104 | 404 | 41.07573787 | 185.0138047 | 2.1712745 | 0 | 0 |
| MSMEG_5810 | 8 | 31 | 1.250423444 | 5.61823045 | 2.1676991 | 3.58E-05 | 7.66E-05 |
| MSMEG_3403 | 8 | 31 | 1.7595779 | 7.905893149 | 2.1676991 | 3.58E-05 | 7.66E-05 |
| MSMEG_4050 | 8 | 31 | 2.566371261 | 11.53086599 | 2.1676991 | 3.58E-05 | 7.66E-05 |
| MSMEG_3269 | 80 | 310 | 15.88375726 | 71.36671113 | 2.1676991 | 0 | 0 |
| MSMEG_4436 | 15 | 58 | 1.70842738 | 7.659562994 | 2.1645932 | 2.24E-07 | 6.19E-07 |
| MSMEG_6723 | 7 | 27 | 2.972466135 | 13.29392959 | 2.1610353 | 0.0001091 | 0.000214 |
| MSMEG_3470 | 14 | 54 | 3.383135799 | 15.13059092 | 2.1610353 | 4.02E-07 | 1.08E-06 |
| MSMEG_4063 | 14 | 54 | 3.394301263 | 15.18052686 | 2.1610353 | 4.02E-07 | 1.08E-06 |
| MSMEG_0272 | 7 | 27 | 5.470602568 | 24.46648744 | 2.1610353 | 0.0001091 | 0.000214 |
| MSMEG_3870 | 7 | 27 | 1.714122138 | 7.666166066 | 2.1610353 | 0.0001091 | 0.0002139 |
| MSMEG_2235 | 6 | 23 | 1.069840447 | 4.755173126 | 2.1521022 | 0.0003598 | 0.0006579 |
| MSMEG_3893 | 12 | 46 | 2.441962682 | 10.85391317 | 2.1521022 | 1.43E-06 | 3.68E-06 |
| MSMEG_5297 | 12 | 46 | 1.52781374 | 6.790749837 | 2.1521022 | 1.43E-06 | 3.68E-06 |
| MSMEG_0748 | 12 | 46 | 2.928732652 | 13.01748391 | 2.1521022 | 1.43E-06 | 3.68E-06 |
| MSMEG_0805 | 6 | 23 | 1.281320535 | 5.695149209 | 2.1521022 | 0.0003598 | 0.0006577 |
| MSMEG_0161 | 35 | 134 | 2.735301284 | 12.1426271 | 2.1503089 | 2.73E-12 | 1.07E-11 |
| MSMEG_4233 | 358 | 1365 | 40.52316038 | 179.1528279 | 2.1443722 | 3.25E-13 | 1.43E-12 |
| MSMEG_6541 | 72 | 274 | 45.20761683 | 199.4804845 | 2.1416098 | 0 | 0 |
| MSMEG_3718 | 20 | 76 | 10.06333936 | 44.3400777 | 2.1395022 | 1.31E-08 | 3.98E-08 |
| MSMEG_2046 | 35 | 133 | 11.42748092 | 50.35062157 | 2.1395022 | 2.73E-12 | 1.07E-11 |
| MSMEG_1179 | 39 | 148 | 5.180890988 | 22.79671645 | 2.1375539 | 4.38E-13 | 1.88E-12 |
| MSMEG_2247 | 9 | 34 | 1.944592341 | 8.517962296 | 2.1310406 | 1.87E-05 | 4.17E-05 |
| MSMEG_6562 | 17 | 64 | 1.658513167 | 7.239702349 | 2.1260399 | 7.28E-08 | 2.10E-07 |
| MSMEG_6523 | 40 | 150 | 10.23865886 | 44.5189667 | 2.1203934 | 3.06E-13 | 1.35E-12 |
| MSMEG_4863 | 12 | 45 | 2.01727352 | 8.771357055 | 2.1203934 | 1.66E-06 | 4.25E-06 |
| MSMEG_0018 | 8 | 30 | 0.996100032 | 4.331167269 | 2.1203934 | 5.55E-05 | 0.0001152 |
| MSMEG_6115 | 8 | 30 | 1.754325429 | 7.628025936 | 2.1203934 | 5.55E-05 | 0.0001152 |
| MSMEG_4356 | 127 | 476 | 31.84205434 | 138.3805479 | 2.1196358 | 0 | 0 |
| MSMEG_2276 | 2132 | 7988 | 437.4910293 | 1900.600079 | 2.1191297 | 0 | 0 |
| MSMEG_1477 | 773 | 2891 | 119.2991968 | 517.3409453 | 2.116531 | 8.31E-13 | 3.44E-12 |
| MSMEG_4085 | 30 | 112 | 5.149232057 | 22.28999479 | 2.1139671 | 4.28E-11 | 1.54E-10 |
| MSMEG_5507 | 15 | 56 | 2.487439414 | 10.76762728 | 2.1139671 | 2.39E-07 | 6.60E-07 |
| MSMEG_5300 | 11 | 41 | 3.325457411 | 14.37187054 | 2.1116231 | 3.80E-06 | 9.30E-06 |
| MSMEG_0980 | 18 | 67 | 2.430740427 | 10.49087268 | 2.1096669 | 4.13E-08 | 1.22E-07 |
| MSMEG_2926 | 1209 | 4491 | 224.8506689 | 968.4599663 | 2.1067252 | 2.83E-12 | 1.11E-11 |
| MSMEG_1457 | 7 | 26 | 3.451252627 | 14.86355837 | 2.1065876 | 0.0001789 | 0.000339 |
| MSMEG_1348 | 7 | 26 | 1.254235711 | 5.401634626 | 2.1065876 | 0.0001789 | 0.0003389 |
| MSMEG_3316 | 14 | 52 | 2.086152704 | 8.984463273 | 2.1065876 | 4.55E-07 | 1.22E-06 |
| MSMEG_0666 | 28 | 104 | 3.042820363 | 13.10455738 | 2.1065876 | 1.34E-10 | 4.67E-10 |
| MSMEG_4083 | 24 | 89 | 3.735375119 | 16.06141196 | 2.1042737 | 1.33E-09 | 4.37E-09 |
| MSMEG_0518 | 17 | 63 | 3.384445569 | 14.54286246 | 2.1033198 | 7.42E-08 | 2.14E-07 |
| MSMEG_6843 | 10 | 37 | 2.681108662 | 11.50235784 | 2.101028 | 9.79E-06 | 2.25E-05 |
| MSMEG_5844 | 10 | 37 | 2.448745912 | 10.50548683 | 2.101028 | 9.79E-06 | 2.25E-05 |
| MSMEG_2254 | 10 | 37 | 2.104939179 | 9.030504439 | 2.101028 | 9.79E-06 | 2.25E-05 |
| MSMEG_2867 | 23 | 85 | 3.230659042 | 13.84372457 | 2.0993317 | 2.36E-09 | 7.62E-09 |
| MSMEG_5503 | 13 | 48 | 2.752192811 | 11.78277205 | 2.0980255 | 9.21E-07 | 2.42E-06 |
| MSMEG_0515 | 42 | 155 | 7.242769597 | 30.99258582 | 2.0973097 | 1.88E-13 | 8.56E-13 |
| MSMEG_2985 | 58 | 213 | 7.803695762 | 33.22941335 | 2.0902314 | 3.64E-14 | 1.82E-13 |
| MSMEG_4058 | 27 | 99 | 4.50791861 | 19.16541517 | 2.0879719 | 2.37E-10 | 8.17E-10 |
| MSMEG_5519 | 15 | 55 | 2.92290626 | 12.42673544 | 2.0879719 | 2.56E-07 | 7.03E-07 |
| MSMEG_6194 | 9 | 33 | 2.421836616 | 10.29643794 | 2.0879719 | 2.83E-05 | 6.12E-05 |
| MSMEG_6761 | 456 | 1667 | 58.87318817 | 249.5508462 | 2.0836511 | 1.45E-12 | 5.83E-12 |
| MSMEG_5165 | 23 | 84 | 14.5658162 | 61.68179593 | 2.0822582 | 2.36E-09 | 7.63E-09 |
| MSMEG_4039 | 17 | 62 | 3.286474776 | 13.89772796 | 2.0802362 | 7.66E-08 | 2.21E-07 |
| MSMEG_6521 | 31 | 113 | 6.415024501 | 27.11351379 | 2.0794854 | 2.43E-11 | 8.86E-11 |
| MSMEG_3943 | 25 | 91 | 5.721368952 | 24.14749436 | 2.0774412 | 7.48E-10 | 2.50E-09 |
| MSMEG_6855 | 11 | 40 | 2.720828791 | 11.47200309 | 2.0759992 | 5.15E-06 | 1.25E-05 |
| MSMEG_5954 | 8 | 29 | 2.772165183 | 11.65192956 | 2.0714838 | 8.93E-05 | 0.0001787 |
| MSMEG_4541 | 8 | 29 | 1.088331516 | 4.574461233 | 2.0714838 | 8.93E-05 | 0.0001786 |
| MSMEG_1874 | 1824 | 6604 | 585.1326475 | 2456.446419 | 2.0697372 | 1.20E-12 | 4.87E-12 |
| MSMEG_0763 | 84 | 304 | 9.279458191 | 38.93925621 | 2.0691129 | 0 | 0 |
| MSMEG_5435 | 868 | 3140 | 116.7863435 | 489.8608353 | 2.0685004 | 0 | 0 |
| MSMEG_2550 | 13 | 47 | 2.690171565 | 11.27730219 | 2.0676519 | 1.12E-06 | 2.91E-06 |
| MSMEG_0141 | 33 | 119 | 7.505444125 | 31.38196635 | 2.0639264 | 7.98E-12 | 3.01E-11 |
| MSMEG_6304 | 45 | 162 | 9.31213234 | 38.87070118 | 2.0614997 | 6.42E-14 | 3.12E-13 |
| MSMEG_3939 | 17 | 61 | 4.730531875 | 19.6816553 | 2.0567773 | 8.07E-08 | 2.31E-07 |
| MSMEG_4734 | 26 | 93 | 3.09065018 | 12.81829277 | 2.0522219 | 4.22E-10 | 1.43E-09 |
| MSMEG_4987 | 7 | 25 | 1.187613491 | 4.917992088 | 2.050004 | 0.0002985 | 0.00055 |
| MSMEG_4807 | 7 | 25 | 1.465061656 | 6.066924712 | 2.050004 | 0.0002985 | 0.0005505 |
| MSMEG_3275 | 7 | 25 | 2.460462399 | 10.18895011 | 2.050004 | 0.0002985 | 0.0005502 |
| MSMEG_4408 | 7 | 25 | 2.972466135 | 12.30919407 | 2.050004 | 0.0002985 | 0.0005503 |
| MSMEG_3202 | 21 | 75 | 3.042820363 | 12.60053594 | 2.050004 | 7.58E-09 | 2.36E-08 |
| MSMEG_1491 | 14 | 50 | 3.042820363 | 12.60053594 | 2.050004 | 6.07E-07 | 1.62E-06 |
| MSMEG_0349 | 21 | 75 | 3.554631162 | 14.71998093 | 2.050004 | 7.58E-09 | 2.36E-08 |
| MSMEG_0019 | 152 | 539 | 1.484088431 | 6.102049013 | 2.0397167 | 6.53E-14 | 3.17E-13 |
| MSMEG_0652 | 22 | 78 | 5.611709381 | 23.06948122 | 2.0394734 | 4.27E-09 | 1.35E-08 |
| MSMEG_2146 | 28 | 99 | 1.692960136 | 6.94056187 | 2.0355045 | 1.34E-10 | 4.67E-10 |
| MSMEG_2096 | 15 | 53 | 4.443288952 | 18.20370974 | 2.0345326 | 3.31E-07 | 9.04E-07 |
| MSMEG_3359 | 17 | 60 | 8.21618694 | 33.62353538 | 2.0329305 | 8.76E-08 | 2.50E-07 |
| MSMEG_0383 | 717 | 2528 | 466.1285359 | 1905.611388 | 2.0314542 | 3.78E-13 | 1.65E-12 |
| MSMEG_4076 | 29 | 102 | 5.326022358 | 21.72080385 | 2.0279471 | 7.57E-11 | 2.69E-10 |
| MSMEG_1306 | 35 | 123 | 5.061384266 | 20.62420005 | 2.0267342 | 2.73E-12 | 1.07E-11 |
| MSMEG_1438 | 172 | 604 | 125.1042466 | 509.3910125 | 2.0256427 | 4.64E-14 | 2.30E-13 |
| MSMEG_2968 | 8 | 28 | 2.798566756 | 11.35728306 | 2.0208577 | 0.0001469 | 0.0002802 |
| MSMEG_5981 | 12 | 42 | 2.555213125 | 10.36969323 | 2.0208577 | 3.86E-06 | 9.45E-06 |
| MSMEG_4728 | 14 | 49 | 2.138198093 | 8.677342047 | 2.0208577 | 7.70E-07 | 2.03E-06 |
| MSMEG_0478 | 12 | 42 | 1.7595779 | 7.140806715 | 2.0208577 | 3.86E-06 | 9.45E-06 |
| MSMEG_6832 | 8 | 28 | 2.825476052 | 11.46648771 | 2.0208577 | 0.0001469 | 0.0002803 |
| MSMEG_0333 | 50 | 175 | 6.891405004 | 27.96704318 | 2.0208577 | 5.26E-14 | 2.58E-13 |
| MSMEG_4092 | 8 | 28 | 1.646215739 | 6.680754742 | 2.0208577 | 0.0001469 | 0.0002803 |
| MSMEG_6405 | 2127 | 7412 | 510.6355445 | 2063.239756 | 2.0145456 | 2.23E-12 | 8.85E-12 |
| MSMEG_2994 | 19 | 66 | 3.404354072 | 13.71184174 | 2.0099694 | 2.71E-08 | 8.10E-08 |
| MSMEG_1707 | 50 | 173 | 16.69599485 | 66.98215805 | 2.0042748 | 5.26E-14 | 2.58E-13 |
| MSMEG_4078 | 48 | 166 | 7.566940156 | 30.34295582 | 2.0035797 | 1.15E-13 | 5.37E-13 |
| MSMEG_4012 | 18 | 62 | 2.789710532 | 11.14163845 | 1.9977741 | 5.45E-08 | 1.59E-07 |
| MSMEG_1641 | 9 | 31 | 3.443548938 | 13.75295996 | 1.9977741 | 7.26E-05 | 0.0001469 |
| MSMEG_3432 | 18 | 62 | 2.698617943 | 10.77782984 | 1.9977741 | 5.45E-08 | 1.59E-07 |
| MSMEG_1473 | 34 | 117 | 40.28581983 | 160.7421917 | 1.9964046 | 4.63E-12 | 1.78E-11 |
| MSMEG_1615 | 16 | 55 | 2.238853405 | 8.923579548 | 1.9948625 | 2.21E-07 | 6.12E-07 |
| MSMEG_6800 | 23 | 79 | 7.786334926 | 31.01009315 | 1.9937216 | 2.50E-09 | 8.08E-09 |
| MSMEG_4879 | 14 | 48 | 3.224054178 | 12.81699656 | 1.9911103 | 1.05E-06 | 2.73E-06 |
| MSMEG_6691 | 12 | 41 | 3.540355535 | 14.02556041 | 1.9860923 | 5.78E-06 | 1.38E-05 |
| MSMEG_1150 | 29 | 99 | 7.77521512 | 30.7765791 | 1.9848784 | 7.64E-11 | 2.71E-10 |
| MSMEG_5348 | 10 | 34 | 1.380871755 | 5.44381049 | 1.9790375 | 3.61E-05 | 7.71E-05 |
| MSMEG_2841 | 10 | 34 | 1.524115713 | 6.008521121 | 1.9790375 | 3.61E-05 | 7.70E-05 |
| MSMEG_2956 | 30 | 102 | 1.885262036 | 7.432268213 | 1.9790375 | 4.32E-11 | 1.55E-10 |
| MSMEG_0189 | 10 | 34 | 1.888492991 | 7.445005605 | 1.9790375 | 3.61E-05 | 7.71E-05 |
| MSMEG_5138 | 23 | 78 | 7.932557178 | 31.19253798 | 1.975343 | 2.61E-09 | 8.40E-09 |
| MSMEG_1235 | 18 | 61 | 2.649945475 | 10.41273948 | 1.9743151 | 6.43E-08 | 1.87E-07 |
| MSMEG_3994 | 18 | 61 | 5.027843316 | 19.7564905 | 1.9743151 | 6.43E-08 | 1.87E-07 |
| MSMEG_4589 | 16 | 54 | 2.264736103 | 8.86261973 | 1.9683903 | 2.88E-07 | 7.89E-07 |
| MSMEG_2275 | 2027 | 6834 | 404.6419535 | 1581.84115 | 1.9668869 | 4.90E-12 | 1.88E-11 |
| MSMEG_5770 | 696 | 2345 | 334.181795 | 1305.530822 | 1.9659315 | 4.69E-13 | 2.01E-12 |
| MSMEG_6858 | 11 | 37 | 2.166450806 | 8.449453215 | 1.9635245 | 1.80E-05 | 4.02E-05 |
| MSMEG_6522 | 14 | 47 | 3.753552127 | 14.61110321 | 1.9607367 | 1.51E-06 | 3.86E-06 |
| MSMEG_2536 | 14 | 47 | 4.081243186 | 15.88667571 | 1.9607367 | 1.51E-06 | 3.86E-06 |
| MSMEG_4459 | 14 | 47 | 3.213979009 | 12.51075712 | 1.9607367 | 1.51E-06 | 3.86E-06 |
| MSMEG_0348 | 20 | 67 | 3.316585885 | 12.88269693 | 1.9576639 | 1.89E-08 | 5.73E-08 |
| MSMEG_1443 | 293 | 981 | 154.8523494 | 601.159785 | 1.9568552 | 5.91E-13 | 2.51E-12 |
| MSMEG_4004 | 32 | 107 | 9.4031843 | 36.45687863 | 1.9549697 | 1.41E-11 | 5.21E-11 |
| MSMEG_5946 | 15 | 50 | 2.504399228 | 9.679502609 | 1.9504684 | 7.75E-07 | 2.04E-06 |
| MSMEG_1870 | 60 | 200 | 3.481629258 | 13.45649652 | 1.9504684 | 0 | 0 |
| MSMEG_6375 | 16 | 53 | 2.759150323 | 10.59746483 | 1.9414232 | 4.00E-07 | 1.08E-06 |
| MSMEG_6880 | 23 | 76 | 3.929382974 | 15.05500313 | 1.9378683 | 3.08E-09 | 9.86E-09 |
| MSMEG_3744 | 45 | 148 | 10.36303129 | 39.51907272 | 1.931103 | 6.42E-14 | 3.12E-13 |
| MSMEG_1178 | 14 | 46 | 3.3610238 | 12.80477992 | 1.9297098 | 2.28E-06 | 5.71E-06 |
| MSMEG_1587 | 14 | 46 | 3.145178235 | 11.98245461 | 1.9297098 | 2.28E-06 | 5.71E-06 |
| MSMEG_4550 | 147 | 482 | 26.86310813 | 102.1307917 | 1.9267198 | 5.86E-14 | 2.86E-13 |
| MSMEG_1298 | 22 | 72 | 3.631847869 | 13.78187158 | 1.9239961 | 6.62E-09 | 2.06E-08 |
| MSMEG_3354 | 11 | 36 | 3.980719955 | 15.10574594 | 1.9239961 | 2.90E-05 | 6.27E-05 |
| MSMEG_4837 | 15 | 49 | 2.550776991 | 9.661577604 | 1.921322 | 1.15E-06 | 2.98E-06 |
| MSMEG_5322 | 23 | 75 | 3.779943353 | 14.29188305 | 1.9187595 | 3.57E-09 | 1.14E-08 |
| MSMEG_4229 | 121 | 394 | 17.99382117 | 67.9367843 | 1.9166913 | 8.44E-15 | 4.40E-14 |
| MSMEG_5982 | 24 | 78 | 3.883473692 | 14.63438456 | 1.9139425 | 1.93E-09 | 6.30E-09 |
| MSMEG_0262 | 12 | 39 | 2.389020401 | 9.002724377 | 1.9139425 | 1.44E-05 | 3.25E-05 |
| MSMEG_2843 | 8 | 26 | 1.300219068 | 4.899712825 | 1.9139425 | 0.0004083 | 0.0007414 |
| MSMEG_4005 | 32 | 104 | 8.05067149 | 30.3379479 | 1.9139425 | 1.47E-11 | 5.44E-11 |
| MSMEG_5593 | 33 | 107 | 3.71818781 | 13.9788645 | 1.9105756 | 8.40E-12 | 3.16E-11 |
| MSMEG_1308 | 54 | 175 | 6.984099255 | 26.24372186 | 1.9098264 | 6.86E-14 | 3.31E-13 |
| MSMEG_0730 | 22 | 71 | 4.030354867 | 15.08167888 | 1.9038183 | 8.26E-09 | 2.56E-08 |
| MSMEG_5807 | 9 | 29 | 2.092282899 | 7.817117297 | 1.9015588 | 0.0001983 | 0.0003736 |
| MSMEG_5951 | 27 | 87 | 5.289291169 | 19.76167253 | 1.9015588 | 3.12E-10 | 1.07E-09 |
| MSMEG_4095 | 9 | 29 | 2.160658157 | 8.072578646 | 1.9015588 | 0.0001983 | 0.0003735 |
| MSMEG_2464 | 14 | 45 | 6.085640727 | 22.68096469 | 1.8980009 | 3.56E-06 | 8.76E-06 |
| MSMEG_2113 | 19 | 61 | 16.23006011 | 60.41810465 | 1.8963126 | 7.88E-08 | 2.26E-07 |
| MSMEG_0439 | 19 | 61 | 3.682810474 | 13.70964908 | 1.8963126 | 7.88E-08 | 2.26E-07 |
| MSMEG_3737 | 20 | 64 | 5.629300946 | 20.88695735 | 1.8915747 | 4.07E-08 | 1.20E-07 |
| MSMEG_2170 | 10 | 32 | 2.246555882 | 8.335620595 | 1.8915747 | 9.67E-05 | 0.0001912 |
| MSMEG_2463 | 40 | 128 | 9.994881271 | 37.08500591 | 1.8915747 | 3.09E-13 | 1.36E-12 |
| MSMEG_6711 | 20 | 64 | 3.222034094 | 11.9550348 | 1.8915747 | 4.07E-08 | 1.20E-07 |
| MSMEG_0720 | 10 | 32 | 2.206077398 | 8.185429233 | 1.8915747 | 9.67E-05 | 0.0001913 |
| MSMEG_5166 | 118 | 377 | 15.93485391 | 59.03073135 | 1.8892804 | 0 | 0 |
| MSMEG_5945 | 16 | 51 | 6.090145272 | 22.50860503 | 1.8859281 | 8.96E-07 | 2.36E-06 |
| MSMEG_4027 | 16 | 51 | 3.508650858 | 12.96764409 | 1.8859281 | 8.96E-07 | 2.35E-06 |
| MSMEG_2752 | 7784 | 24783 | 1786.972329 | 6596.895612 | 1.88427 | 4.12E-12 | 1.59E-11 |
| MSMEG_0482 | 22 | 70 | 2.86048195 | 10.55322762 | 1.8833542 | 1.10E-08 | 3.38E-08 |
| MSMEG_2631 | 29 | 92 | 4.671949436 | 17.18536253 | 1.8790837 | 1.06E-10 | 3.75E-10 |
| MSMEG_4986 | 19 | 60 | 2.198086881 | 8.048468311 | 1.8724658 | 1.16E-07 | 3.28E-07 |
| MSMEG_5978 | 19 | 60 | 6.259126321 | 22.9182842 | 1.8724658 | 1.16E-07 | 3.28E-07 |
| MSMEG_0968 | 505 | 1593 | 76.49175373 | 279.7755451 | 1.8708937 | 8.33E-14 | 3.97E-13 |
| MSMEG_0523 | 142 | 447 | 35.00556236 | 127.7694344 | 1.8678867 | 1.85E-13 | 8.44E-13 |
| MSMEG_3947 | 21 | 66 | 4.632762535 | 16.88244779 | 1.8655795 | 3.01E-08 | 8.98E-08 |
| MSMEG_3056 | 14 | 44 | 3.097811093 | 11.28886569 | 1.8655795 | 5.69E-06 | 1.36E-05 |
| MSMEG_1440 | 86 | 270 | 67.21026012 | 244.6648744 | 1.8640536 | 0 | 0 |
| MSMEG_2601 | 208 | 653 | 61.3661626 | 223.382706 | 1.8640022 | 2.76E-13 | 1.23E-12 |
| MSMEG_6719 | 15 | 47 | 3.852921889 | 13.99804993 | 1.861201 | 2.82E-06 | 7.03E-06 |
| MSMEG_3715 | 114 | 357 | 3.252314958 | 11.80936909 | 1.860393 | 9.59E-14 | 4.53E-13 |
| MSMEG_5600 | 16 | 50 | 3.060932389 | 11.09109674 | 1.8573589 | 1.41E-06 | 3.62E-06 |
| MSMEG_1964 | 16 | 50 | 2.516912286 | 9.119873978 | 1.8573589 | 1.41E-06 | 3.61E-06 |
| MSMEG_0432 | 16 | 50 | 3.045072636 | 11.03362992 | 1.8573589 | 1.41E-06 | 3.62E-06 |
| MSMEG_2944 | 33 | 103 | 12.36866557 | 44.76276104 | 1.8556092 | 1.11E-11 | 4.16E-11 |
| MSMEG_3142 | 239 | 745 | 85.64638139 | 309.5552151 | 1.8537326 | 3.71E-13 | 1.62E-12 |
| MSMEG_6138 | 26 | 81 | 4.282560114 | 15.46984184 | 1.852913 | 1.11E-09 | 3.66E-09 |
| MSMEG_0334 | 68 | 211 | 7.455883074 | 26.82522454 | 1.8471391 | 0 | 0 |
| MSMEG_4338 | 118 | 366 | 23.6845916 | 85.17962296 | 1.8465595 | 0 | 0 |
| MSMEG_0870 | 30 | 93 | 3.052453352 | 10.97189049 | 1.845771 | 8.47E-11 | 3.00E-10 |
| MSMEG_6295 | 20 | 62 | 3.243372068 | 11.65813824 | 1.845771 | 8.90E-08 | 2.54E-07 |
| MSMEG_4002 | 55 | 170 | 11.19232896 | 40.11228782 | 1.841534 | 1.62E-14 | 8.31E-14 |
| MSMEG_0374 | 23 | 71 | 2.712094188 | 9.707469069 | 1.8396879 | 1.16E-08 | 3.55E-08 |
| MSMEG_1614 | 12 | 37 | 3.314092211 | 11.84829342 | 1.8379936 | 3.80E-05 | 8.11E-05 |
| MSMEG_3624 | 12 | 37 | 4.158247774 | 14.86625495 | 1.8379936 | 3.80E-05 | 8.12E-05 |
| MSMEG_3708 | 12 | 37 | 2.448745912 | 8.754572359 | 1.8379936 | 3.80E-05 | 8.12E-05 |
| MSMEG_6379 | 37 | 114 | 6.121864779 | 21.87044373 | 1.8369394 | 1.34E-12 | 5.41E-12 |
| MSMEG_2889 | 14 | 43 | 2.49025008 | 8.868580598 | 1.8324126 | 9.19E-06 | 2.13E-05 |
| MSMEG_2486 | 46 | 141 | 7.205265155 | 25.60837279 | 1.8294922 | 1.01E-13 | 4.77E-13 |
| MSMEG_6712 | 16 | 49 | 3.246955905 | 11.52983847 | 1.8282126 | 2.25E-06 | 5.64E-06 |
| MSMEG_0659 | 16 | 49 | 3.743305852 | 13.29236154 | 1.8282126 | 2.25E-06 | 5.63E-06 |
| MSMEG_4573 | 17 | 52 | 2.472990921 | 8.770971077 | 1.8264796 | 1.11E-06 | 2.91E-06 |
| MSMEG_4094 | 17 | 52 | 3.091238651 | 10.96371385 | 1.8264796 | 1.11E-06 | 2.91E-06 |
| MSMEG_0315 | 19 | 58 | 2.556383094 | 9.048384856 | 1.8235562 | 2.77E-07 | 7.60E-07 |
| MSMEG_0807 | 21 | 64 | 3.187417199 | 11.26342122 | 1.8211853 | 6.93E-08 | 2.01E-07 |
| MSMEG_1551 | 42 | 128 | 47.46799767 | 167.7383344 | 1.8211853 | 2.05E-13 | 9.28E-13 |
| MSMEG_2724 | 185 | 559 | 71.90761804 | 251.9333822 | 1.8088258 | 0 | 0 |
| MSMEG_0658 | 17 | 51 | 4.135299387 | 14.38463831 | 1.7984653 | 1.79E-06 | 4.54E-06 |
| MSMEG_6169 | 9 | 27 | 1.712853358 | 5.958160155 | 1.7984653 | 0.0005443 | 0.0009748 |
| MSMEG_5729 | 16 | 48 | 4.980500159 | 17.32466908 | 1.7984653 | 3.62E-06 | 8.89E-06 |
| MSMEG_2885 | 10 | 30 | 2.481837072 | 8.633069894 | 1.7984653 | 0.0002631 | 0.0004885 |
| MSMEG_2907 | 13 | 39 | 1.973163028 | 6.863647304 | 1.7984653 | 3.04E-05 | 6.56E-05 |
| MSMEG_2493 | 22 | 66 | 4.040430754 | 14.05463779 | 1.7984653 | 5.44E-08 | 1.59E-07 |
| MSMEG_6766 | 9 | 27 | 2.369754108 | 8.243189318 | 1.7984653 | 0.0005443 | 0.0009745 |
| MSMEG_3146 | 207 | 618 | 66.11613961 | 228.8739434 | 1.7914788 | 9.57E-14 | 4.53E-13 |
| MSMEG_2074 | 37 | 110 | 6.629531614 | 22.85306957 | 1.7854091 | 3.31E-12 | 1.29E-11 |
| MSMEG_1876 | 88 | 261 | 11.03189284 | 37.93836449 | 1.7819771 | 0 | 0 |
| MSMEG_0277 | 29 | 86 | 4.98924811 | 17.15561493 | 1.7817865 | 6.81E-10 | 2.28E-09 |
| MSMEG_5393 | 28 | 83 | 2.872830399 | 9.874174169 | 1.7811873 | 1.35E-09 | 4.45E-09 |
| MSMEG_0915 | 52 | 154 | 12.90555278 | 44.31642546 | 1.7798496 | 7.62E-14 | 3.65E-13 |
| MSMEG_0325 | 51 | 151 | 21.90983184 | 75.21709396 | 1.7794822 | 2.98E-14 | 1.50E-13 |
| MSMEG_1633 | 49 | 145 | 3.284358111 | 11.26920194 | 1.778702 | 4.82E-14 | 2.38E-13 |
| MSMEG_3490 | 48 | 142 | 8.70665213 | 29.86544805 | 1.7782874 | 1.16E-13 | 5.42E-13 |
| MSMEG_3020 | 162 | 478 | 42.80901126 | 146.4599272 | 1.7745196 | 0 | 0 |
| MSMEG_4852 | 20 | 59 | 4.605791683 | 15.75422494 | 1.7742177 | 3.49E-07 | 9.51E-07 |
| MSMEG_6717 | 19 | 56 | 3.90976238 | 13.36150948 | 1.7729302 | 7.05E-07 | 1.87E-06 |
| MSMEG_6339 | 33 | 97 | 3.866440913 | 13.17770881 | 1.7690215 | 6.84E-11 | 2.44E-10 |
| MSMEG_1989 | 15 | 44 | 3.309116097 | 11.2549652 | 1.7660438 | 1.19E-05 | 2.73E-05 |
| MSMEG_2274 | 420 | 1232 | 354.6459596 | 1206.221787 | 1.7660438 | 4.22E-14 | 2.10E-13 |
| MSMEG_4062 | 15 | 44 | 2.642531559 | 8.987777962 | 1.7660438 | 1.19E-05 | 2.72E-05 |
| MSMEG_6458 | 29 | 85 | 4.356664505 | 14.80627393 | 1.7649127 | 1.06E-09 | 3.52E-09 |
| MSMEG_4509 | 14 | 41 | 2.358883676 | 8.010010416 | 1.7636998 | 2.43E-05 | 5.34E-05 |
| MSMEG_4317 | 14 | 41 | 4.321316314 | 14.67380059 | 1.7636998 | 2.43E-05 | 5.34E-05 |
| MSMEG_6815 | 79 | 231 | 22.1508695 | 75.10111795 | 1.7614711 | 0 | 0 |
| MSMEG_1445 | 226 | 660 | 167.7019927 | 567.864153 | 1.759646 | 8.26E-14 | 3.95E-13 |
| MSMEG_4592 | 23 | 67 | 4.071408865 | 13.75189094 | 1.75603 | 6.85E-08 | 1.99E-07 |
| MSMEG_6663 | 34 | 99 | 10.07145496 | 34.00315594 | 1.7553965 | 5.34E-11 | 1.91E-10 |
| MSMEG_5542 | 138 | 401 | 107.8490221 | 363.3726469 | 1.7524367 | 1.16E-13 | 5.42E-13 |
| MSMEG_6129 | 665 | 1924 | 327.8689996 | 1099.903319 | 1.7461853 | 4.03E-13 | 1.75E-12 |
| MSMEG_5631 | 28 | 81 | 4.025335745 | 13.50205374 | 1.7459978 | 3.35E-09 | 1.07E-08 |
| MSMEG_6389 | 105 | 303 | 7.122391155 | 23.83141806 | 1.7424312 | 0 | 0 |
| MSMEG_4150 | 26 | 75 | 5.504385622 | 18.41058133 | 1.7418817 | 1.35E-08 | 4.11E-08 |
| MSMEG_4512 | 25 | 72 | 1.840239913 | 6.145223299 | 1.7395716 | 2.71E-08 | 8.10E-08 |
| MSMEG_5043 | 25 | 72 | 10.20310796 | 34.07184918 | 1.7395716 | 2.71E-08 | 8.10E-08 |
| MSMEG_1442 | 449 | 1281 | 119.5094472 | 395.3445544 | 1.7259859 | 1.02E-12 | 4.20E-12 |
| MSMEG_1906 | 53 | 151 | 4.227476655 | 13.96538878 | 1.723987 | 6.99E-14 | 3.37E-13 |
| MSMEG_0378 | 13 | 37 | 4.613579254 | 15.22534323 | 1.7225164 | 8.07E-05 | 0.0001624 |
| MSMEG_5501 | 55 | 156 | 5.316356255 | 17.4842384 | 1.7175453 | 1.64E-14 | 8.41E-14 |
| MSMEG_2462 | 103 | 292 | 9.725738903 | 31.96972995 | 1.7168268 | 0 | 0 |
| MSMEG_3474 | 18 | 51 | 6.09365342 | 20.01917406 | 1.7160031 | 3.71E-06 | 9.09E-06 |
| MSMEG_0514 | 18 | 51 | 3.019001809 | 9.918175276 | 1.7160031 | 3.71E-06 | 9.09E-06 |
| MSMEG_6887 | 35 | 99 | 6.180728863 | 20.27111219 | 1.7135764 | 1.05E-10 | 3.70E-10 |
| MSMEG_4213 | 11 | 31 | 2.093487437 | 6.840850548 | 1.7082675 | 0.0003412 | 0.0006256 |
| MSMEG_2228 | 11 | 31 | 3.03791786 | 9.92694854 | 1.7082675 | 0.0003412 | 0.0006258 |
| MSMEG_2035 | 586 | 1651 | 94.40559896 | 308.4025384 | 1.7078703 | 0 | 0 |
| MSMEG_1695 | 777 | 2185 | 105.9003102 | 345.30144 | 1.7051495 | 0 | 0 |
| MSMEG_2461 | 89 | 250 | 24.85989195 | 80.96922334 | 1.7035536 | 0 | 0 |
| MSMEG_3954 | 47 | 132 | 3.806760458 | 12.39659342 | 1.703308 | 1.17E-13 | 5.44E-13 |
| MSMEG_0530 | 456 | 1280 | 143.1574533 | 465.9398179 | 1.7025408 | 1.45E-12 | 5.83E-12 |
| MSMEG_4727 | 51 | 143 | 1.773949453 | 5.767370304 | 1.7009488 | 4.00E-14 | 1.99E-13 |
| MSMEG_6816 | 30 | 84 | 3.056686991 | 9.923839568 | 1.6989296 | 3.38E-09 | 1.08E-08 |
| MSMEG_4560 | 142 | 397 | 30.68134583 | 99.45973622 | 1.6967508 | 1.85E-13 | 8.45E-13 |
| MSMEG_1769 | 83 | 232 | 60.9737732 | 197.6167253 | 1.6964443 | 1.04E-13 | 4.90E-13 |
| MSMEG_1470 | 432 | 1206 | 176.3097056 | 570.7034738 | 1.6946294 | 8.06E-13 | 3.34E-12 |
| MSMEG_6852 | 33 | 92 | 7.870969001 | 25.443264 | 1.6926706 | 6.69E-10 | 2.24E-09 |
| MSMEG_5141 | 59 | 164 | 10.57141528 | 34.07184918 | 1.6884117 | 4.95E-14 | 2.43E-13 |
| MSMEG_5943 | 18 | 50 | 4.607396489 | 14.83965557 | 1.6874339 | 5.96E-06 | 1.42E-05 |
| MSMEG_2163 | 36 | 100 | 5.498223668 | 17.70886132 | 1.6874339 | 1.33E-10 | 4.65E-10 |
| MSMEG_3326 | 81 | 224 | 14.9885455 | 48.06104671 | 1.6810077 | 1.27E-14 | 6.54E-14 |
| MSMEG_1114 | 17 | 47 | 4.5413106 | 14.55797192 | 1.6806288 | 1.21E-05 | 2.77E-05 |
| MSMEG_1187 | 46 | 127 | 6.352010071 | 20.3342333 | 1.6786255 | 6.23E-13 | 2.63E-12 |
| MSMEG_2984 | 46 | 127 | 12.4237844 | 39.77136807 | 1.6786255 | 6.23E-13 | 2.64E-12 |
| MSMEG_1474 | 327 | 902 | 162.3121445 | 519.1352696 | 1.6773396 | 6.25E-13 | 2.63E-12 |
| MSMEG_6459 | 82 | 226 | 3.90402783 | 12.47608217 | 1.6761297 | 0 | 0 |
| MSMEG_6876 | 12 | 33 | 3.65787771 | 11.66359982 | 1.6729344 | 0.0002685 | 0.0004981 |
| MSMEG_5917 | 12 | 33 | 3.115012467 | 9.93260621 | 1.6729344 | 0.0002685 | 0.000498 |
| MSMEG_2432 | 36 | 99 | 9.582049219 | 30.55356041 | 1.6729344 | 2.12E-10 | 7.32E-10 |
| MSMEG_3789 | 31 | 85 | 4.908046762 | 15.60402576 | 1.6686974 | 4.29E-09 | 1.35E-08 |
| MSMEG_1749 | 27 | 74 | 6.746544858 | 21.43976904 | 1.6680686 | 4.38E-08 | 1.29E-07 |
| MSMEG_2148 | 23 | 63 | 3.100247117 | 9.846451828 | 1.6672207 | 4.51E-07 | 1.21E-06 |
| MSMEG_2513 | 15 | 41 | 3.94959018 | 12.51743563 | 1.6641642 | 5.03E-05 | 0.0001049 |
| MSMEG_4093 | 26 | 71 | 5.552388985 | 17.58067799 | 1.6628102 | 8.80E-08 | 2.51E-07 |
| MSMEG_3669 | 11 | 30 | 2.226132647 | 7.039638261 | 1.6609617 | 0.0005534 | 0.0009902 |
| MSMEG_2271 | 560 | 1522 | 158.2266589 | 498.6284082 | 1.6559724 | 0 | 0 |
| MSMEG_1437 | 451 | 1225 | 153.3867231 | 483.0788802 | 1.6550852 | 1.51E-12 | 6.08E-12 |
| MSMEG_4040 | 14 | 38 | 4.632762535 | 14.58029582 | 1.6540754 | 0.0001028 | 0.0002023 |
| MSMEG_4670 | 21 | 57 | 3.451252627 | 10.86183112 | 1.6540754 | 1.84E-06 | 4.67E-06 |
| MSMEG_5084 | 821 | 2225 | 198.3967493 | 623.4363851 | 1.651854 | 0 | 0 |
| MSMEG_4082 | 41 | 111 | 6.708145816 | 21.0577687 | 1.6503666 | 2.67E-11 | 9.72E-11 |
| MSMEG_4225 | 17 | 46 | 3.739103039 | 11.73132532 | 1.6496019 | 1.94E-05 | 4.32E-05 |
| MSMEG_2100 | 40 | 108 | 6.618232193 | 20.71936775 | 1.6464622 | 5.32E-11 | 1.90E-10 |
| MSMEG_5754 | 30 | 81 | 5.441657581 | 17.03592459 | 1.6464622 | 1.37E-08 | 4.17E-08 |
| MSMEG_6591 | 43 | 116 | 5.474665903 | 17.12449959 | 1.645219 | 1.07E-11 | 4.01E-11 |
| MSMEG_0152 | 36 | 97 | 7.325888046 | 22.88759952 | 1.6434906 | 5.34E-10 | 1.80E-09 |
| MSMEG_2865 | 13 | 35 | 5.000057097 | 15.60883143 | 1.6423461 | 0.0002106 | 0.0003954 |
| MSMEG_0327 | 90 | 242 | 13.19683425 | 41.14464821 | 1.6405129 | 0 | 0 |
| MSMEG_6638 | 182 | 488 | 17.31885062 | 53.84411399 | 1.6364455 | 0 | 0 |
| MSMEG_5514 | 25 | 67 | 8.424584558 | 26.17905843 | 1.6357358 | 2.82E-07 | 7.74E-07 |
| MSMEG_5953 | 31 | 83 | 9.07304262 | 28.16696695 | 1.6343459 | 1.08E-08 | 3.34E-08 |
| MSMEG_3867 | 68 | 182 | 6.750596837 | 20.94958294 | 1.6338346 | 0 | 0 |
| MSMEG_6867 | 21 | 56 | 5.60985427 | 17.34566867 | 1.6285403 | 2.93E-06 | 7.29E-06 |
| MSMEG_5786 | 12 | 32 | 5.687409859 | 17.58547055 | 1.6285403 | 0.0004326 | 0.000782 |
| MSMEG_0445 | 29 | 77 | 3.901847881 | 12.01251093 | 1.6223083 | 4.36E-08 | 1.28E-07 |
| MSMEG_0158 | 95 | 252 | 16.57702102 | 50.98637764 | 1.6209271 | 0 | 0 |
| MSMEG_2162 | 17 | 45 | 4.460215767 | 13.68958226 | 1.617893 | 3.11E-05 | 6.68E-05 |
| MSMEG_4511 | 50 | 132 | 2.01819718 | 6.177862764 | 1.6140407 | 8.88E-13 | 3.67E-12 |
| MSMEG_3933 | 63 | 166 | 10.23922516 | 31.28278188 | 1.6112623 | 0 | 0 |
| MSMEG_2517 | 166 | 437 | 41.47875728 | 126.610528 | 1.6099528 | 1.38E-13 | 6.37E-13 |
| MSMEG_3318 | 19 | 50 | 3.412677676 | 10.41315684 | 1.6094314 | 1.20E-05 | 2.74E-05 |
| MSMEG_6781 | 38 | 100 | 5.685479306 | 17.34819205 | 1.6094314 | 5.30E-10 | 1.78E-09 |
| MSMEG_5872 | 275 | 722 | 84.17564071 | 256.2486991 | 1.60607 | 5.34E-13 | 2.28E-12 |
| MSMEG_5606 | 24 | 63 | 5.294585755 | 16.1150638 | 1.6058202 | 9.01E-07 | 2.37E-06 |
| MSMEG_1203 | 484 | 1269 | 120.5281038 | 366.416751 | 1.6041159 | 1.82E-12 | 7.26E-12 |
| MSMEG_1611 | 29 | 76 | 9.426588243 | 28.64447498 | 1.6034493 | 6.89E-08 | 2.00E-07 |
| MSMEG_4565 | 152 | 398 | 32.74569313 | 99.41785905 | 1.6021999 | 6.53E-14 | 3.17E-13 |
| MSMEG_1919 | 590 | 1544 | 509.9153251 | 1547.262798 | 1.6013887 | 0 | 0 |
| MSMEG_3748 | 1578 | 4126 | 290.5354172 | 880.8298855 | 1.6001494 | 9.45E-13 | 3.89E-12 |
| MSMEG_2753 | 986 | 2576 | 506.5307976 | 1534.424537 | 1.5989758 | 0 | 0 |
| MSMEG_2005 | 18 | 47 | 4.279361787 | 12.95612388 | 1.5981666 | 2.43E-05 | 5.35E-05 |
| MSMEG_0351 | 36 | 94 | 5.331946743 | 16.14291242 | 1.5981666 | 2.11E-09 | 6.85E-09 |
| MSMEG_4155 | 18 | 47 | 3.810728508 | 11.53729763 | 1.5981666 | 2.43E-05 | 5.35E-05 |
| MSMEG_2526 | 28 | 73 | 3.12605861 | 9.45001896 | 1.5959724 | 1.38E-07 | 3.90E-07 |
| MSMEG_1471 | 266 | 693 | 152.6640029 | 461.1678024 | 1.5949319 | 1.08E-13 | 5.09E-13 |
| MSMEG_0703 | 333 | 867 | 117.6104407 | 355.0516015 | 1.5940126 | 4.19E-13 | 1.81E-12 |
| MSMEG_2075 | 35 | 91 | 6.986910889 | 21.06343937 | 1.5920144 | 4.21E-09 | 1.33E-08 |
| MSMEG_6823 | 20 | 52 | 5.996928763 | 18.07894038 | 1.5920144 | 9.42E-06 | 2.17E-05 |
| MSMEG_1564 | 32 | 83 | 4.778040803 | 14.36973314 | 1.5885422 | 2.14E-08 | 6.45E-08 |
| MSMEG_4372 | 17 | 44 | 3.421535383 | 10.26822852 | 1.5854715 | 4.94E-05 | 0.000103 |
| MSMEG_4467 | 24 | 62 | 6.653196439 | 19.92881745 | 1.5827366 | 1.42E-06 | 3.66E-06 |
| MSMEG_5958 | 19 | 49 | 5.650952103 | 16.89798188 | 1.5802851 | 1.90E-05 | 4.24E-05 |
| MSMEG_4412 | 14 | 36 | 5.142366414 | 15.33233213 | 1.5760728 | 0.0002625 | 0.0004875 |
| MSMEG_6756 | 44 | 113 | 6.388032813 | 19.02232687 | 1.5742501 | 8.14E-11 | 2.89E-10 |
| MSMEG_4202 | 30 | 77 | 4.963674145 | 14.77214182 | 1.5733987 | 8.54E-08 | 2.44E-07 |
| MSMEG_0489 | 16 | 41 | 3.006133088 | 8.931878622 | 1.5710548 | 0.0001003 | 0.0001977 |
| MSMEG_2273 | 1997 | 5110 | 192.2730899 | 570.4690345 | 1.5689917 | 1.46E-12 | 5.90E-12 |
| MSMEG_0281 | 27 | 69 | 3.785275169 | 11.21640073 | 1.5671397 | 4.36E-07 | 1.17E-06 |
| MSMEG_5930 | 36 | 92 | 7.033631873 | 20.84182264 | 1.5671397 | 5.21E-09 | 1.63E-08 |
| MSMEG_4065 | 45 | 115 | 6.345118965 | 18.80164422 | 1.5671397 | 6.41E-11 | 2.29E-10 |
| MSMEG_3992 | 47 | 120 | 7.522291362 | 22.26918247 | 1.5658045 | 2.54E-11 | 9.25E-11 |
| MSMEG_4329 | 1151 | 2934 | 178.0109396 | 526.1410816 | 1.5634838 | 2.86E-12 | 1.12E-11 |
| MSMEG_5329 | 55 | 140 | 16.35801925 | 48.27994823 | 1.5614261 | 6.47E-13 | 2.72E-12 |
| MSMEG_4460 | 24 | 61 | 3.258959439 | 9.604356747 | 1.5592776 | 2.24E-06 | 5.63E-06 |
| MSMEG_5961 | 30 | 76 | 7.018698472 | 20.61672403 | 1.5545397 | 1.34E-07 | 3.78E-07 |
| MSMEG_5021 | 81 | 205 | 17.05000735 | 50.03387595 | 1.5531329 | 1.27E-14 | 6.53E-14 |
| MSMEG_0269 | 49 | 124 | 13.84483265 | 40.62412787 | 1.5529892 | 1.58E-11 | 5.82E-11 |
| MSMEG_5373 | 38 | 96 | 8.162486372 | 23.9100696 | 1.5505377 | 3.21E-09 | 1.03E-08 |
| MSMEG_4635 | 59 | 149 | 9.631733919 | 28.2039196 | 1.5500282 | 2.05E-13 | 9.29E-13 |
| MSMEG_3047 | 257 | 649 | 16.91741127 | 49.53546174 | 1.5499529 | 4.21E-13 | 1.81E-12 |
| MSMEG_5434 | 425 | 1073 | 215.3207612 | 630.3292099 | 1.5496181 | 9.99E-13 | 4.10E-12 |
| MSMEG_5719 | 50 | 126 | 9.114438877 | 26.63184241 | 1.5469265 | 1.24E-11 | 4.62E-11 |
| MSMEG_5523 | 31 | 78 | 3.685005983 | 10.7508262 | 1.5447087 | 1.05E-07 | 2.98E-07 |
| MSMEG_3944 | 33 | 83 | 11.12045162 | 32.43077388 | 1.5441481 | 4.12E-08 | 1.22E-07 |
| MSMEG_4921 | 709 | 1777 | 329.6507945 | 958.0012025 | 1.5390889 | 1.58E-12 | 6.33E-12 |
| MSMEG_0157 | 30 | 75 | 3.81292616 | 11.05271924 | 1.5354309 | 2.09E-07 | 5.83E-07 |
| MSMEG_0449 | 22 | 55 | 2.423046929 | 7.02380699 | 1.5354309 | 9.05E-06 | 2.11E-05 |
| MSMEG_3410 | 26 | 65 | 9.845473252 | 28.53956439 | 1.5354309 | 1.37E-06 | 3.53E-06 |
| MSMEG_1990 | 14 | 35 | 2.872830399 | 8.327616769 | 1.5354309 | 0.0004157 | 0.0007539 |
| MSMEG_0175 | 18 | 45 | 3.488978344 | 10.11367555 | 1.5354309 | 6.07E-05 | 0.0001242 |
| MSMEG_0410 | 163 | 407 | 11.93855185 | 34.5644133 | 1.5336596 | 1.61E-13 | 7.40E-13 |
| MSMEG_1439 | 587 | 1464 | 154.560629 | 446.964043 | 1.5319859 | 0 | 0 |
| MSMEG_1183 | 33 | 82 | 4.707297966 | 13.56258074 | 1.5266606 | 6.43E-08 | 1.87E-07 |
| MSMEG_4098 | 23 | 57 | 3.049882092 | 8.763968427 | 1.5228308 | 7.05E-06 | 1.67E-05 |
| MSMEG_2525 | 42 | 104 | 6.335564371 | 18.19030963 | 1.5216251 | 1.21E-09 | 4.01E-09 |
| MSMEG_2255 | 38 | 94 | 5.389131929 | 15.4573061 | 1.5201641 | 7.79E-09 | 2.42E-08 |
| MSMEG_2690 | 1721 | 4257 | 136.3848451 | 391.1646763 | 1.5200928 | 4.72E-12 | 1.81E-11 |
| MSMEG_5834 | 331 | 816 | 96.49224961 | 275.8197315 | 1.5152407 | 0 | 0 |
| MSMEG_5984 | 24 | 59 | 3.397104155 | 9.683232667 | 1.5111833 | 5.49E-06 | 1.32E-05 |
| MSMEG_2723 | 722 | 1774 | 151.5423898 | 431.7390032 | 1.510438 | 7.24E-14 | 3.48E-13 |
| MSMEG_2567 | 22 | 54 | 3.200341191 | 9.108316118 | 1.5089586 | 1.41E-05 | 3.20E-05 |
| MSMEG_1318 | 40 | 98 | 9.666102282 | 27.45922056 | 1.5062845 | 4.76E-09 | 1.50E-08 |
| MSMEG_2014 | 25 | 61 | 5.073368601 | 14.35347238 | 1.5003839 | 4.27E-06 | 1.04E-05 |
| MSMEG_0505 | 76 | 185 | 12.21693803 | 34.48190426 | 1.4969567 | 0 | 0 |
| MSMEG_2848 | 21 | 51 | 3.282361541 | 9.242895257 | 1.4936107 | 2.84E-05 | 6.13E-05 |
| MSMEG_4327 | 2153 | 5228 | 379.2913631 | 1067.911436 | 1.4934136 | 0 | 0 |
| MSMEG_6928 | 57 | 138 | 5.227659811 | 14.67514103 | 1.4891372 | 5.47E-12 | 2.09E-11 |
| MSMEG_1861 | 19 | 46 | 5.150498781 | 14.45853379 | 1.4891372 | 7.36E-05 | 0.0001488 |
| MSMEG_4147 | 24 | 58 | 4.148463662 | 11.62451325 | 1.4865213 | 8.53E-06 | 2.00E-05 |
| MSMEG_1441 | 123 | 297 | 58.67449619 | 164.2749871 | 1.4853074 | 0 | 0 |
| MSMEG_3387 | 51 | 123 | 7.232782326 | 20.22604947 | 1.4835919 | 8.60E-11 | 3.05E-10 |
| MSMEG_2537 | 61 | 147 | 9.657769435 | 26.98578572 | 1.4824378 | 1.37E-12 | 5.51E-12 |
| MSMEG_2370 | 976 | 2351 | 255.1575811 | 712.6594077 | 1.4818242 | 1.73E-13 | 7.91E-13 |
| MSMEG_1320 | 27 | 65 | 4.570240065 | 12.7573168 | 1.4809831 | 2.58E-06 | 6.44E-06 |
| MSMEG_4001 | 79 | 190 | 17.32396361 | 48.31083093 | 1.4795776 | 0 | 0 |
| MSMEG_0833 | 57 | 137 | 40.26303374 | 112.2077725 | 1.4786448 | 8.37E-12 | 3.15E-11 |
| MSMEG_4916 | 1707 | 4100 | 180.9527823 | 503.9487072 | 1.4776636 | 4.49E-12 | 1.73E-11 |
| MSMEG_6496 | 15 | 36 | 4.338329371 | 12.07270247 | 1.4765372 | 0.0005035 | 0.0009051 |
| MSMEG_3300 | 25 | 60 | 5.073368601 | 14.11816955 | 1.4765372 | 6.62E-06 | 1.57E-05 |
| MSMEG_6813 | 25 | 60 | 5.775344131 | 16.07162697 | 1.4765372 | 6.62E-06 | 1.57E-05 |
| MSMEG_0502 | 51 | 122 | 8.162486372 | 22.64033551 | 1.4718148 | 1.32E-10 | 4.62E-10 |
| MSMEG_1468 | 44 | 105 | 52.13459037 | 144.2558131 | 1.4683167 | 2.72E-09 | 8.76E-09 |
| MSMEG_3250 | 34 | 81 | 8.763932736 | 24.20894547 | 1.4658899 | 1.86E-07 | 5.19E-07 |
| MSMEG_4047 | 113 | 269 | 25.78027528 | 71.15937446 | 1.4647862 | 2.41E-13 | 1.08E-12 |
| MSMEG_6081 | 552 | 1314 | 154.7756958 | 427.198567 | 1.4647279 | 0 | 0 |
| MSMEG_2496 | 16 | 38 | 3.358280107 | 9.248073349 | 1.4614303 | 0.0003876 | 0.0007057 |
| MSMEG_0510 | 28 | 66 | 4.664277927 | 12.74797078 | 1.450542 | 3.09E-06 | 7.65E-06 |
| MSMEG_2277 | 330 | 777 | 47.53447946 | 129.7736609 | 1.4489513 | 2.35E-13 | 1.06E-12 |
| MSMEG_6940 | 48 | 113 | 14.94150048 | 40.78515845 | 1.4487192 | 1.00E-09 | 3.33E-09 |
| MSMEG_0323 | 65 | 153 | 11.58993817 | 31.63223862 | 1.4485228 | 1.09E-12 | 4.44E-12 |
| MSMEG_5971 | 17 | 40 | 7.854468018 | 21.42883596 | 1.447968 | 0.0002983 | 0.0005502 |
| MSMEG_3556 | 20 | 47 | 2.803907532 | 7.640157021 | 1.4461635 | 8.82E-05 | 0.0001766 |
| MSMEG_6734 | 20 | 47 | 3.354446454 | 9.140279176 | 1.4461635 | 8.82E-05 | 0.0001766 |
| MSMEG_0456 | 89 | 209 | 9.157075047 | 24.93353109 | 1.4451285 | 0 | 0 |
| MSMEG_3364 | 23 | 54 | 8.084376454 | 22.00813225 | 1.4448283 | 2.63E-05 | 5.71E-05 |
| MSMEG_4234 | 323 | 758 | 62.2791283 | 169.4649717 | 1.4441664 | 1.18E-13 | 5.48E-13 |
| MSMEG_3005 | 577 | 1347 | 134.1385814 | 363.0916206 | 1.4366094 | 3.48E-13 | 1.52E-12 |
| MSMEG_5449 | 21 | 49 | 4.564230545 | 12.34852522 | 1.4358952 | 6.80E-05 | 0.0001378 |
| MSMEG_0117 | 36 | 84 | 9.182797168 | 24.8440567 | 1.4358952 | 1.72E-07 | 4.82E-07 |
| MSMEG_0526 | 24 | 56 | 3.307874402 | 8.949453819 | 1.4358952 | 2.03E-05 | 4.51E-05 |
| MSMEG_2425 | 24 | 56 | 3.917993458 | 10.60013086 | 1.4358952 | 2.03E-05 | 4.51E-05 |
| MSMEG_1254 | 909 | 2116 | 40.03435312 | 108.0576032 | 1.4324902 | 2.01E-12 | 8.00E-12 |
| MSMEG_0937 | 95 | 221 | 30.88020287 | 83.29511802 | 1.4315497 | 0 | 0 |
| MSMEG_5942 | 43 | 100 | 3.614281723 | 9.745952283 | 1.4310942 | 1.25E-08 | 3.84E-08 |
| MSMEG_6392 | 1079 | 2503 | 43.62460383 | 117.3387982 | 1.4274662 | 0 | 0 |
| MSMEG_1319 | 22 | 51 | 4.197850134 | 11.28353447 | 1.4264965 | 5.25E-05 | 0.0001092 |
| MSMEG_4963 | 19 | 44 | 3.323298023 | 8.923579548 | 1.4250069 | 0.0001766 | 0.0003349 |
| MSMEG_3586 | 19 | 44 | 3.165045736 | 8.498647188 | 1.4250069 | 0.0001766 | 0.0003348 |
| MSMEG_3279 | 29 | 67 | 7.77521512 | 20.82859393 | 1.421611 | 3.67E-06 | 9.00E-06 |
| MSMEG_0115 | 26 | 60 | 8.414193 | 22.51443779 | 1.4199536 | 1.21E-05 | 2.77E-05 |
| MSMEG_3045 | 23 | 53 | 10.11757293 | 27.03305399 | 1.4178613 | 4.05E-05 | 8.56E-05 |
| MSMEG_4235 | 226 | 520 | 50.77216294 | 135.4538347 | 1.4156916 | 8.26E-14 | 3.95E-13 |
| MSMEG_3899 | 30 | 69 | 16.82344519 | 44.86560293 | 1.4151366 | 2.83E-06 | 7.06E-06 |
| MSMEG_4203 | 34 | 78 | 4.067949234 | 10.82086415 | 1.4114421 | 6.67E-07 | 1.77E-06 |
| MSMEG_2955 | 48 | 110 | 23.6657323 | 62.8842854 | 1.4099 | 3.54E-09 | 1.13E-08 |
| MSMEG_1472 | 534 | 1221 | 182.4600442 | 483.7410215 | 1.4066543 | 0 | 0 |
| MSMEG_3742 | 35 | 80 | 8.542136901 | 22.63910245 | 1.4061478 | 5.16E-07 | 1.38E-06 |
| MSMEG_5137 | 42 | 96 | 12.64516331 | 33.51329428 | 1.4061478 | 3.74E-08 | 1.11E-07 |
| MSMEG_6324 | 21 | 48 | 2.58843947 | 6.860103862 | 1.4061478 | 0.0001045 | 0.0002056 |
| MSMEG_5131 | 46 | 105 | 8.53350848 | 22.58550609 | 1.4041863 | 8.92E-09 | 2.76E-08 |
| MSMEG_1467 | 445 | 1014 | 173.8870102 | 459.4262642 | 1.4016832 | 0 | 0 |
| MSMEG_1418 | 36 | 82 | 15.11226048 | 39.91273761 | 1.4011298 | 3.99E-07 | 1.08E-06 |
| MSMEG_0171 | 33 | 75 | 7.820188556 | 20.6079733 | 1.3979273 | 1.31E-06 | 3.38E-06 |
| MSMEG_1846 | 22 | 50 | 4.739508216 | 12.48968079 | 1.3979273 | 8.04E-05 | 0.0001621 |
| MSMEG_3611 | 605 | 1373 | 56.1880383 | 147.8528727 | 1.3958273 | 1.86E-13 | 8.47E-13 |
| MSMEG_0624 | 26 | 59 | 4.152221328 | 10.92521251 | 1.3957061 | 1.85E-05 | 4.14E-05 |
| MSMEG_4575 | 34 | 77 | 5.918769739 | 15.54225348 | 1.3928265 | 1.01E-06 | 2.65E-06 |
| MSMEG_3623 | 46 | 104 | 14.75663475 | 38.68419558 | 1.3903805 | 1.35E-08 | 4.11E-08 |
| MSMEG_6805 | 27 | 61 | 3.91219761 | 10.2484359 | 1.3893526 | 1.43E-05 | 3.23E-05 |
| MSMEG_4326 | 665 | 1497 | 488.5248093 | 1275.138956 | 1.3841507 | 4.03E-13 | 1.75E-12 |
| MSMEG_0611 | 28 | 63 | 4.22370958 | 11.01912987 | 1.3834278 | 1.10E-05 | 2.53E-05 |
| MSMEG_2876 | 20 | 45 | 5.014496747 | 13.08219465 | 1.3834278 | 0.000208 | 0.0003909 |
| MSMEG_3481 | 20 | 45 | 4.185890447 | 10.92046448 | 1.3834278 | 0.000208 | 0.000391 |
| MSMEG_2021 | 56 | 126 | 6.076651597 | 15.85322377 | 1.3834278 | 4.65E-10 | 1.57E-09 |
| MSMEG_0245 | 49 | 110 | 29.26549992 | 76.17689858 | 1.3801526 | 6.28E-09 | 1.96E-08 |
| MSMEG_2272 | 164 | 368 | 107.5699097 | 279.875904 | 1.3795127 | 7.53E-14 | 3.62E-13 |
| MSMEG_6069 | 21 | 47 | 3.856774811 | 10.0086057 | 1.3757742 | 0.0001596 | 0.0003036 |
| MSMEG_4006 | 26 | 58 | 3.874283592 | 10.02113211 | 1.371044 | 2.82E-05 | 6.10E-05 |
| MSMEG_3434 | 35 | 78 | 12.24372956 | 31.63814567 | 1.369622 | 1.18E-06 | 3.06E-06 |
| MSMEG_2912 | 22 | 49 | 3.521072553 | 9.093249509 | 1.368781 | 0.0001225 | 0.0002395 |
| MSMEG_6220 | 31 | 69 | 5.16402199 | 13.327424 | 1.3678309 | 5.05E-06 | 1.22E-05 |
| MSMEG_1525 | 452 | 1006 | 166.0249728 | 428.4535035 | 1.3677384 | 2.95E-13 | 1.31E-12 |
| MSMEG_5957 | 40 | 89 | 5.912464977 | 15.25349385 | 1.3673081 | 2.16E-07 | 6.00E-07 |
| MSMEG_1372 | 80 | 178 | 23.22921023 | 59.92874659 | 1.3673081 | 1.15E-13 | 5.37E-13 |
| MSMEG_2928 | 89 | 198 | 18.11122322 | 46.71901758 | 1.3671259 | 0 | 0 |
| MSMEG_0476 | 18 | 40 | 3.060932389 | 7.887002126 | 1.3655059 | 0.0005405 | 0.0009685 |
| MSMEG_4097 | 27 | 60 | 4.837766313 | 12.46531068 | 1.3655059 | 2.17E-05 | 4.80E-05 |
| MSMEG_2416 | 2049 | 4545 | 611.8878503 | 1573.745473 | 1.3628631 | 7.85E-12 | 2.97E-11 |
| MSMEG_0218 | 65 | 144 | 28.93972441 | 74.33858003 | 1.3610599 | 4.71E-11 | 1.69E-10 |
| MSMEG_6388 | 113 | 250 | 7.679230934 | 19.69926525 | 1.3591081 | 2.41E-13 | 1.08E-12 |
| MSMEG_6243 | 19 | 42 | 8.834083352 | 22.64268458 | 1.3578927 | 0.0004136 | 0.0007502 |
| MSMEG_4531 | 20 | 44 | 5.342718352 | 13.62873967 | 1.3510063 | 0.0003166 | 0.000582 |
| MSMEG_5578 | 60 | 132 | 8.940654444 | 22.80671446 | 1.3510063 | 3.79E-10 | 1.29E-09 |
| MSMEG_3113 | 35 | 77 | 5.183837111 | 13.22344953 | 1.3510063 | 1.79E-06 | 4.54E-06 |
| MSMEG_3091 | 66 | 145 | 9.396350591 | 23.93613436 | 1.3490177 | 5.45E-11 | 1.95E-10 |
| MSMEG_4300 | 31 | 68 | 10.07669778 | 25.62926708 | 1.3467693 | 7.62E-06 | 1.80E-05 |
| MSMEG_1812 | 63 | 138 | 48.7171555 | 123.7346102 | 1.3447473 | 1.76E-10 | 6.11E-10 |
| MSMEG_5581 | 53 | 116 | 16.15562655 | 40.99932059 | 1.3435633 | 5.09E-09 | 1.60E-08 |
| MSMEG_0749 | 27 | 59 | 8.549500812 | 21.66205929 | 1.3412583 | 3.28E-05 | 7.04E-05 |
| MSMEG_0180 | 22 | 48 | 9.506895892 | 24.05071707 | 1.3390336 | 0.0001857 | 0.0003516 |
| MSMEG_0390 | 77 | 168 | 15.66926054 | 39.64037855 | 1.3390336 | 2.19E-12 | 8.68E-12 |
| MSMEG_3248 | 50 | 109 | 10.61595048 | 26.83404307 | 1.3378309 | 1.65E-08 | 4.98E-08 |
| MSMEG_6899 | 39 | 85 | 5.162221111 | 13.04552784 | 1.3374915 | 6.32E-07 | 1.68E-06 |
| MSMEG_3580 | 5870 | 12764 | 1306.739864 | 3294.644568 | 1.3341509 | 2.67E-11 | 9.72E-11 |
| MSMEG_6121 | 35 | 76 | 7.474369788 | 18.81875391 | 1.3321473 | 2.68E-06 | 6.69E-06 |
| MSMEG_0148 | 24 | 52 | 8.395700268 | 21.09209711 | 1.32898 | 0.0001091 | 0.000214 |
| MSMEG_1419 | 25 | 54 | 7.749195923 | 19.40801536 | 1.3245341 | 8.37E-05 | 0.0001679 |
| MSMEG_4601 | 25 | 54 | 7.118447417 | 17.82829318 | 1.3245341 | 8.37E-05 | 0.0001679 |
| MSMEG_4344 | 26 | 56 | 4.143214341 | 10.34719932 | 1.320418 | 6.42E-05 | 0.0001307 |
| MSMEG_6919 | 4495 | 9670 | 1048.296464 | 2614.879219 | 1.3186975 | 2.84E-11 | 1.03E-10 |
| MSMEG_6693 | 53 | 114 | 8.427502163 | 21.01834852 | 1.3184723 | 1.13E-08 | 3.48E-08 |
| MSMEG_5077 | 34 | 73 | 11.10098147 | 27.63605545 | 1.3158645 | 5.21E-06 | 1.26E-05 |
| MSMEG_3028 | 34 | 73 | 9.017042707 | 22.44805948 | 1.3158645 | 5.21E-06 | 1.26E-05 |
| MSMEG_6196 | 41 | 88 | 6.354340656 | 15.81393844 | 1.3153824 | 5.62E-07 | 1.50E-06 |
| MSMEG_3006 | 1279 | 2745 | 220.5595789 | 548.8686972 | 1.3152926 | 4.65E-12 | 1.79E-11 |
| MSMEG_1313 | 69 | 148 | 6.490274055 | 16.14159308 | 1.3144317 | 8.39E-11 | 2.98E-10 |
| MSMEG_4096 | 21 | 45 | 3.744441564 | 9.303599595 | 1.3130384 | 0.000366 | 0.0006678 |
| MSMEG_0147 | 43 | 92 | 10.3569909 | 25.69352561 | 1.3108 | 3.34E-07 | 9.10E-07 |
| MSMEG_0182 | 65 | 139 | 12.37060758 | 30.67349117 | 1.310076 | 3.48E-10 | 1.19E-09 |
| MSMEG_6871 | 37 | 79 | 7.229010537 | 17.89678248 | 1.3078301 | 2.38E-06 | 5.94E-06 |
| MSMEG_2389 | 6490 | 13855 | 2281.200139 | 5646.716153 | 1.3076191 | 3.29E-11 | 1.19E-10 |
| MSMEG_5102 | 23 | 49 | 3.123169462 | 7.714975092 | 1.3046506 | 0.0002141 | 0.000402 |
| MSMEG_4000 | 100 | 213 | 14.57586852 | 35.99853113 | 1.3043562 | 0 | 0 |
| MSMEG_0798 | 31 | 66 | 10.21225873 | 25.21011262 | 1.3037006 | 1.71E-05 | 3.83E-05 |
| MSMEG_3280 | 98 | 208 | 18.08872608 | 44.51598386 | 1.2992326 | 0 | 0 |
| MSMEG_2779 | 124 | 263 | 46.95533397 | 115.4754682 | 1.2982254 | 7.82E-14 | 3.74E-13 |
| MSMEG_1169 | 25 | 53 | 4.425444418 | 10.87836149 | 1.297567 | 0.0001255 | 0.0002451 |
| MSMEG_5877 | 268 | 567 | 55.14822725 | 135.2852835 | 1.2946185 | 3.75E-14 | 1.88E-13 |
| MSMEG_5245 | 240 | 507 | 62.74366748 | 153.6870777 | 1.2924541 | 0 | 0 |
| MSMEG_1466 | 162 | 342 | 112.2726899 | 274.8248212 | 1.2915053 | 0 | 0 |
| MSMEG_3314 | 29 | 61 | 5.434716691 | 13.25499235 | 1.2862591 | 4.33E-05 | 9.11E-05 |
| MSMEG_5537 | 196 | 412 | 21.49048651 | 52.37911143 | 1.2852934 | 6.64E-14 | 3.21E-13 |
| MSMEG_3970 | 59 | 124 | 9.261282614 | 22.56895993 | 1.285056 | 5.29E-09 | 1.66E-08 |
| MSMEG_6296 | 30 | 63 | 5.845812521 | 14.2342606 | 1.2838921 | 3.32E-05 | 7.13E-05 |
| MSMEG_1908 | 30 | 63 | 4.843673232 | 11.79410164 | 1.2838921 | 3.32E-05 | 7.13E-05 |
| MSMEG_6165 | 32 | 67 | 12.57110201 | 30.51890234 | 1.279592 | 1.96E-05 | 4.35E-05 |
| MSMEG_0965 | 1776 | 3717 | 615.4206706 | 1493.455936 | 1.2790099 | 4.74E-12 | 1.82E-11 |
| MSMEG_3981 | 99 | 207 | 18.4588207 | 44.75173084 | 1.2776331 | 3.80E-14 | 1.90E-13 |
| MSMEG_6944 | 33 | 69 | 20.89877976 | 50.66718952 | 1.2776331 | 1.50E-05 | 3.39E-05 |
| MSMEG_1548 | 1319 | 2756 | 482.0740086 | 1167.935527 | 1.2766341 | 2.88E-12 | 1.13E-11 |
| MSMEG_6561 | 35 | 73 | 6.317403457 | 15.27791763 | 1.2740443 | 8.87E-06 | 2.08E-05 |
| MSMEG_2078 | 2419 | 5044 | 545.1088675 | 1317.932571 | 1.2736603 | 0 | 0 |
| MSMEG_1063 | 111 | 231 | 75.50299894 | 182.1897491 | 1.2708359 | 0 | 0 |
| MSMEG_6654 | 37 | 77 | 5.807922995 | 14.01459608 | 1.2708359 | 5.24E-06 | 1.26E-05 |
| MSMEG_1983 | 28 | 58 | 4.56085713 | 10.95436393 | 1.2641288 | 8.40E-05 | 0.0001684 |
| MSMEG_4328 | 2650 | 5464 | 465.7303827 | 1113.448468 | 1.2574679 | 0 | 0 |
| MSMEG_4149 | 34 | 70 | 8.00551548 | 19.11081284 | 1.2553229 | 1.71E-05 | 3.83E-05 |
| MSMEG_3145 | 1239 | 2550 | 182.7708545 | 436.16072 | 1.2548238 | 1.25E-12 | 5.07E-12 |
| MSMEG_1547 | 3318 | 6820 | 426.8794536 | 1017.381836 | 1.2529606 | 3.81E-12 | 1.48E-11 |
| MSMEG_1207 | 23 | 47 | 4.299324883 | 10.18687603 | 1.2445297 | 0.0004764 | 0.000859 |
| MSMEG_5816 | 46 | 94 | 8.007747294 | 18.97366009 | 1.2445297 | 7.29E-07 | 1.93E-06 |
| MSMEG_5595 | 24 | 49 | 11.15884213 | 26.41646535 | 1.2432501 | 0.0003637 | 0.0006641 |
| MSMEG_0006 | 901 | 1835 | 78.51672834 | 185.414719 | 1.2396838 | 2.75E-13 | 1.22E-12 |
| MSMEG_1253 | 416 | 847 | 26.97294702 | 63.67797056 | 1.2392812 | 0 | 0 |
| MSMEG_0350 | 31 | 63 | 5.992983415 | 14.12188486 | 1.2365864 | 5.58E-05 | 0.0001159 |
| MSMEG_3544 | 62 | 126 | 15.08168012 | 35.53851819 | 1.2365864 | 1.13E-08 | 3.48E-08 |
| MSMEG_0998 | 31 | 63 | 6.15495594 | 14.50355742 | 1.2365864 | 5.58E-05 | 0.0001158 |
| MSMEG_5051 | 33 | 67 | 5.547502179 | 13.05957606 | 1.2351978 | 3.28E-05 | 7.04E-05 |
| MSMEG_5059 | 745 | 1512 | 196.1629789 | 461.6186018 | 1.2346486 | 3.66E-13 | 1.60E-12 |
| MSMEG_6648 | 103 | 209 | 6.684297585 | 15.7266265 | 1.2343614 | 1.46E-13 | 6.70E-13 |
| MSMEG_1435 | 188 | 381 | 135.4012445 | 318.1709446 | 1.2325611 | 1.17E-13 | 5.45E-13 |
| MSMEG_1436 | 415 | 841 | 139.8481037 | 328.6057931 | 1.2324972 | 0 | 0 |
| MSMEG_3931 | 38 | 77 | 7.711520274 | 18.11831759 | 1.2323618 | 8.74E-06 | 2.05E-05 |
| MSMEG_3985 | 76 | 154 | 12.57464117 | 29.54428364 | 1.2323618 | 3.02E-10 | 1.04E-09 |
| MSMEG_5909 | 40 | 81 | 8.904530587 | 20.90772563 | 1.2314247 | 5.16E-06 | 1.25E-05 |
| MSMEG_6197 | 43 | 87 | 6.867135274 | 16.11005912 | 1.2301815 | 2.35E-06 | 5.87E-06 |
| MSMEG_2044 | 50 | 101 | 8.386116135 | 19.64187653 | 1.2278581 | 3.75E-07 | 1.02E-06 |
| MSMEG_5908 | 54 | 109 | 7.702851217 | 18.02830855 | 1.2267996 | 1.32E-07 | 3.73E-07 |
| MSMEG_4597 | 80 | 161 | 25.44151596 | 59.367616 | 1.2224915 | 1.57E-10 | 5.45E-10 |
| MSMEG_1465 | 218 | 438 | 130.2016119 | 303.3225598 | 1.2201055 | 2.66E-14 | 1.35E-13 |
| MSMEG_4461 | 26 | 52 | 8.340706598 | 19.34209779 | 1.2135028 | 0.0003131 | 0.0005761 |
| MSMEG_1281 | 123 | 246 | 8.351083561 | 19.36616197 | 1.2135028 | 0 | 0 |
| MSMEG_0560 | 32 | 64 | 6.621960775 | 15.35632639 | 1.2135028 | 6.28E-05 | 0.0001281 |
| MSMEG_1992 | 33 | 66 | 6.23202687 | 14.4520697 | 1.2135028 | 4.82E-05 | 0.0001006 |
| MSMEG_6143 | 38 | 76 | 4.747568604 | 11.00961113 | 1.2135028 | 1.28E-05 | 2.91E-05 |
| MSMEG_1066 | 38 | 76 | 26.84202249 | 62.24664754 | 1.2135028 | 1.28E-05 | 2.91E-05 |
| MSMEG_0784 | 50 | 100 | 9.743020868 | 22.59406444 | 1.2135028 | 5.47E-07 | 1.46E-06 |
| MSMEG_2494 | 35 | 70 | 6.609725468 | 15.32795272 | 1.2135028 | 2.83E-05 | 6.12E-05 |
| MSMEG_0638 | 309 | 616 | 35.24825248 | 81.47616109 | 1.2088263 | 7.04E-13 | 2.94E-12 |
| MSMEG_0270 | 91 | 181 | 19.95545176 | 46.02242315 | 1.205554 | 1.92E-11 | 7.03E-11 |
| MSMEG_1453 | 93 | 184 | 12.58195413 | 28.8638133 | 1.1979059 | 1.67E-11 | 6.16E-11 |
| MSMEG_3112 | 42 | 83 | 11.64309377 | 26.67890077 | 1.1962248 | 6.50E-06 | 1.54E-05 |
| MSMEG_0005 | 921 | 1820 | 100.0870555 | 229.3297541 | 1.1961681 | 0 | 0 |
| MSMEG_0997 | 40 | 79 | 13.99283378 | 32.04376292 | 1.1953554 | 1.10E-05 | 2.52E-05 |
| MSMEG_4840 | 33 | 65 | 5.411291188 | 12.35865065 | 1.1914765 | 7.04E-05 | 0.0001424 |
| MSMEG_0335 | 32 | 63 | 9.291684091 | 21.21073615 | 1.1907827 | 9.18E-05 | 0.0001823 |
| MSMEG_5252 | 63 | 124 | 14.7863571 | 33.74528194 | 1.1904191 | 3.89E-08 | 1.16E-07 |
| MSMEG_2120 | 29 | 57 | 2.440330977 | 5.561556138 | 1.1884118 | 0.0002048 | 0.0003854 |
| MSMEG_3026 | 58 | 114 | 24.91706366 | 56.7864153 | 1.1884118 | 1.43E-07 | 4.02E-07 |
| MSMEG_1417 | 159 | 312 | 27.2909299 | 62.09355692 | 1.186022 | 1.60E-14 | 8.21E-14 |
| MSMEG_1382 | 129 | 253 | 9.789924254 | 22.262856 | 1.1852691 | 0 | 0 |
| MSMEG_3046 | 101 | 198 | 19.37258515 | 44.03541866 | 1.1846479 | 4.36E-12 | 1.68E-11 |
| MSMEG_1963 | 74 | 145 | 4.798072307 | 10.9011874 | 1.1839585 | 3.24E-09 | 1.04E-08 |
| MSMEG_2532 | 66 | 129 | 25.25269221 | 57.23005917 | 1.1803359 | 2.58E-08 | 7.72E-08 |
| MSMEG_0324 | 41 | 80 | 19.81550968 | 44.8313805 | 1.1778789 | 1.23E-05 | 2.80E-05 |
| MSMEG_6865 | 37 | 72 | 5.524609678 | 12.46531068 | 1.1739744 | 3.53E-05 | 7.57E-05 |
| MSMEG_5583 | 88 | 171 | 10.22893862 | 23.04701824 | 1.1719237 | 1.81E-10 | 6.27E-10 |
| MSMEG_3952 | 296 | 575 | 68.16571691 | 153.5369379 | 1.1714675 | 4.06E-13 | 1.76E-12 |
| MSMEG_2149 | 34 | 66 | 16.43237388 | 36.98588892 | 1.170434 | 7.84E-05 | 0.0001581 |
| MSMEG_3986 | 100 | 194 | 13.50411348 | 30.37655672 | 1.1695594 | 1.18E-11 | 4.41E-11 |
| MSMEG_0167 | 431 | 836 | 67.65445435 | 152.1584718 | 1.1693178 | 0 | 0 |
| MSMEG_3462 | 65 | 126 | 21.50925463 | 48.34519141 | 1.1684149 | 4.81E-08 | 1.41E-07 |
| MSMEG_0708 | 46 | 89 | 10.6266332 | 23.83958001 | 1.1656742 | 4.76E-06 | 1.15E-05 |
| MSMEG_1469 | 413 | 798 | 228.1200139 | 511.0777377 | 1.1637497 | 4.08E-13 | 1.76E-12 |
| MSMEG_3144 | 86 | 166 | 31.43166394 | 70.3473503 | 1.1622774 | 4.34E-10 | 1.47E-09 |
| MSMEG_4336 | 1213 | 2337 | 521.1103142 | 1164.121514 | 1.1595809 | 5.00E-12 | 1.91E-11 |
| MSMEG_4111 | 77 | 148 | 15.00425214 | 33.43921538 | 1.1561696 | 4.43E-09 | 1.40E-08 |
| MSMEG_1980 | 1126 | 2164 | 211.0169308 | 470.2262859 | 1.1559964 | 2.57E-12 | 1.01E-11 |
| MSMEG_6309 | 37 | 71 | 10.53530218 | 23.44090399 | 1.1537965 | 5.11E-05 | 0.0001065 |
| MSMEG_6318 | 48 | 92 | 6.090145272 | 13.53458603 | 1.1521022 | 4.05E-06 | 9.89E-06 |
| MSMEG_5004 | 153 | 293 | 29.19413957 | 64.82501176 | 1.1508718 | 0 | 0 |
| MSMEG_2943 | 47 | 90 | 18.56307385 | 41.21594659 | 1.150767 | 5.27E-06 | 1.27E-05 |
| MSMEG_5823 | 58 | 111 | 7.384433078 | 16.38637461 | 1.1499376 | 4.27E-07 | 1.15E-06 |
| MSMEG_5187 | 69 | 132 | 12.06881914 | 26.77073864 | 1.1493724 | 3.51E-08 | 1.05E-07 |
| MSMEG_1699 | 34 | 65 | 6.521464307 | 14.45607178 | 1.1484077 | 0.0001135 | 0.0002221 |
| MSMEG_6088 | 68 | 130 | 27.29749541 | 60.51011467 | 1.1484077 | 4.55E-08 | 1.34E-07 |
| MSMEG_1244 | 1153 | 2197 | 73.46237735 | 162.3067057 | 1.1436451 | 1.36E-12 | 5.50E-12 |
| MSMEG_5931 | 31 | 59 | 8.725416466 | 19.25516381 | 1.1419495 | 0.0002527 | 0.0004698 |
| MSMEG_3213 | 62 | 118 | 8.118836712 | 17.91656953 | 1.1419495 | 2.16E-07 | 5.99E-07 |
| MSMEG_4365 | 149 | 282 | 15.33038407 | 33.64237209 | 1.1338856 | 2.25E-13 | 1.02E-12 |
| MSMEG_4046 | 120 | 227 | 22.77903174 | 49.96324137 | 1.1331607 | 6.88E-13 | 2.88E-12 |
| MSMEG_6281 | 723 | 1367 | 200.4275427 | 439.3982814 | 1.1324485 | 3.13E-14 | 1.58E-13 |
| MSMEG_3499 | 72 | 136 | 26.31488144 | 57.63397374 | 1.1310406 | 3.30E-08 | 9.82E-08 |
| MSMEG_3608 | 54 | 102 | 8.112409768 | 17.76752871 | 1.1310406 | 1.73E-06 | 4.42E-06 |
| MSMEG_0438 | 61 | 115 | 14.09183968 | 30.8039517 | 1.1282555 | 4.00E-07 | 1.08E-06 |
| MSMEG_2701 | 129 | 243 | 139.3624511 | 304.3918879 | 1.127088 | 1.80E-14 | 9.19E-14 |
| MSMEG_6594 | 94 | 177 | 23.17269621 | 50.59326598 | 1.1265195 | 3.28E-10 | 1.12E-09 |
| MSMEG_4590 | 34 | 64 | 5.600270919 | 12.22308491 | 1.1260399 | 0.0001634 | 0.0003105 |
| MSMEG_4458 | 41 | 77 | 5.814589713 | 12.66183584 | 1.1227373 | 3.67E-05 | 7.83E-05 |
| MSMEG_1586 | 30 | 56 | 5.876990188 | 12.72015703 | 1.1139671 | 0.0004758 | 0.0008581 |
| MSMEG_4112 | 172 | 321 | 22.72577141 | 49.17744419 | 1.1136675 | 4.64E-14 | 2.30E-13 |
| MSMEG_5410 | 81 | 151 | 11.37753837 | 24.59296953 | 1.1120575 | 9.28E-09 | 2.86E-08 |
| MSMEG_6391 | 366 | 681 | 51.90584963 | 111.9832495 | 1.1093139 | 0 | 0 |
| MSMEG_4665 | 129 | 240 | 30.86855595 | 66.58993326 | 1.1091661 | 3.32E-13 | 1.46E-12 |
| MSMEG_4611 | 50 | 93 | 8.443951419 | 18.21081594 | 1.1088054 | 7.03E-06 | 1.67E-05 |
| MSMEG_2460 | 57 | 106 | 14.79630922 | 31.9047351 | 1.1085332 | 1.61E-06 | 4.12E-06 |
| MSMEG_5544 | 71 | 132 | 20.86331517 | 44.97484092 | 1.1081498 | 8.63E-08 | 2.46E-07 |
| MSMEG_1144 | 104 | 193 | 21.70479331 | 46.70360009 | 1.1055201 | 1.02E-10 | 3.59E-10 |
| MSMEG_2778 | 69 | 128 | 12.18486547 | 26.20911476 | 1.1049783 | 1.45E-07 | 4.07E-07 |
| MSMEG_2097 | 75 | 139 | 15.01274741 | 32.26149207 | 1.1036251 | 4.35E-08 | 1.28E-07 |
| MSMEG_1904 | 94 | 174 | 17.35041073 | 37.23933265 | 1.1018574 | 9.32E-10 | 3.10E-09 |
| MSMEG_2826 | 572 | 1058 | 102.9913722 | 220.8824536 | 1.1007553 | 0 | 0 |
| MSMEG_5139 | 126 | 233 | 16.86021775 | 36.15091466 | 1.100409 | 1.52E-12 | 6.10E-12 |
| MSMEG_4493 | 99 | 183 | 24.08203761 | 51.61546689 | 1.099846 | 3.66E-10 | 1.25E-09 |
| MSMEG_0684 | 291 | 537 | 30.32276852 | 64.88150004 | 1.0974057 | 2.36E-13 | 1.06E-12 |
| MSMEG_0615 | 45 | 83 | 5.603062679 | 11.98289611 | 1.0966891 | 2.61E-05 | 5.66E-05 |
| MSMEG_1147 | 45 | 83 | 7.741936723 | 16.55716324 | 1.0966891 | 2.61E-05 | 5.66E-05 |
| MSMEG_0485 | 32 | 59 | 4.609404069 | 9.854113244 | 1.0961458 | 0.0003992 | 0.0007259 |
| MSMEG_6757 | 108 | 199 | 31.23597147 | 66.73521641 | 1.0952399 | 7.29E-11 | 2.59E-10 |
| MSMEG_2459 | 209 | 385 | 57.50425792 | 122.82455 | 1.0948583 | 1.59E-13 | 7.31E-13 |
| MSMEG_6025 | 50 | 92 | 8.367013365 | 17.85085492 | 1.0932085 | 1.00E-05 | 2.30E-05 |
| MSMEG_0812 | 43 | 79 | 7.76138139 | 16.53363689 | 1.0910188 | 4.41E-05 | 9.26E-05 |
| MSMEG_5932 | 53 | 97 | 10.35506915 | 21.97453039 | 1.0854951 | 6.48E-06 | 1.54E-05 |
| MSMEG_4236 | 1946 | 3561 | 992.762405 | 2106.421093 | 1.0852735 | 6.34E-12 | 2.41E-11 |
| MSMEG_6062 | 35 | 64 | 9.73933033 | 20.64960557 | 1.0842197 | 0.0002562 | 0.000476 |
| MSMEG_3422 | 217 | 395 | 46.34109269 | 97.80799729 | 1.0776604 | 2.71E-13 | 1.21E-12 |
| MSMEG_6011 | 59 | 107 | 16.48015309 | 34.65482759 | 1.0723267 | 2.72E-06 | 6.78E-06 |
| MSMEG_5893 | 42 | 76 | 19.04580153 | 39.96081077 | 1.0691129 | 8.12E-05 | 0.0001634 |
| MSMEG_5400 | 94 | 170 | 13.0046393 | 27.27031243 | 1.0683048 | 3.66E-09 | 1.16E-08 |
| MSMEG_5341 | 103 | 186 | 18.9639721 | 39.70779416 | 1.066161 | 7.24E-10 | 2.42E-09 |
| MSMEG_0747 | 87 | 156 | 25.98059687 | 54.01634626 | 1.0559615 | 2.18E-08 | 6.58E-08 |
| MSMEG_6752 | 105 | 188 | 24.56544465 | 50.9992647 | 1.0538461 | 8.50E-10 | 2.83E-09 |
| MSMEG_6568 | 228 | 408 | 44.66512543 | 92.67542978 | 1.0530381 | 3.93E-13 | 1.71E-12 |
| MSMEG_1177 | 52 | 93 | 7.975038877 | 16.53800613 | 1.0522219 | 1.67E-05 | 3.74E-05 |
| MSMEG_1552 | 622 | 1112 | 97.01401 | 201.1034835 | 1.0516731 | 0 | 0 |
| MSMEG_2839 | 141 | 252 | 12.99648081 | 26.9325784 | 1.0512313 | 1.35E-12 | 5.43E-12 |
| MSMEG_0731 | 108 | 193 | 38.1439267 | 79.03686168 | 1.0510723 | 5.54E-10 | 1.86E-09 |
| MSMEG_4430 | 56 | 100 | 5.413017278 | 11.20784513 | 1.050004 | 8.31E-06 | 1.95E-05 |
| MSMEG_5565 | 42 | 75 | 14.76277439 | 30.56685034 | 1.050004 | 0.0001147 | 0.0002243 |
| MSMEG_1193 | 148 | 263 | 19.24324221 | 39.64998378 | 1.0429684 | 5.40E-13 | 2.30E-12 |
| MSMEG_4116 | 120 | 213 | 29.68176862 | 61.08841646 | 1.0413218 | 9.96E-11 | 3.51E-10 |
| MSMEG_6136 | 339 | 601 | 77.10138056 | 158.4921158 | 1.0395825 | 1.14E-13 | 5.35E-13 |
| MSMEG_2458 | 258 | 456 | 57.96114176 | 118.7825935 | 1.0351655 | 1.16E-13 | 5.43E-13 |
| MSMEG_1145 | 77 | 136 | 16.30144973 | 33.38452081 | 1.0341791 | 2.81E-07 | 7.71E-07 |
| MSMEG_1524 | 981 | 1728 | 205.3179264 | 419.3458361 | 1.0302809 | 1.69E-12 | 6.76E-12 |
| MSMEG_0247 | 196 | 345 | 35.55216286 | 72.56041955 | 1.0292455 | 6.64E-14 | 3.22E-13 |
| MSMEG_5562 | 54 | 95 | 24.79355235 | 50.57540113 | 1.0284709 | 1.95E-05 | 4.34E-05 |
| MSMEG_1401 | 2200 | 3858 | 407.0962976 | 827.7657062 | 1.0238524 | 0 | 0 |
| MSMEG_6307 | 81 | 142 | 12.50095077 | 25.41072786 | 1.0233999 | 1.96E-07 | 5.46E-07 |
| MSMEG_1205 | 1118 | 1958 | 266.6588892 | 541.4990316 | 1.0219633 | 0 | 0 |
| MSMEG_1149 | 44 | 77 | 14.11504194 | 28.64118326 | 1.0208577 | 0.0001339 | 0.0002564 |
| MSMEG_3689 | 5399 | 9418 | 687.3888653 | 1390.332217 | 1.0162313 | 1.11E-11 | 4.14E-11 |
| MSMEG_5474 | 39 | 68 | 9.582049219 | 19.37195438 | 1.0155634 | 0.0003536 | 0.0006475 |
| MSMEG_3007 | 1741 | 3034 | 254.7768904 | 514.8107093 | 1.0148076 | 2.99E-12 | 1.17E-11 |
| MSMEG_1243 | 248 | 432 | 50.8901385 | 102.7865841 | 1.014194 | 0 | 0 |
| MSMEG_1708 | 116 | 202 | 25.43771872 | 51.3620413 | 1.0137332 | 7.28E-10 | 2.44E-09 |
| MSMEG_5221 | 58 | 101 | 23.03144803 | 46.50346983 | 1.0137332 | 1.35E-05 | 3.07E-05 |
| MSMEG_5633 | 57 | 99 | 8.528218959 | 17.17471013 | 1.0099694 | 1.75E-05 | 3.91E-05 |
| MSMEG_6583 | 884 | 1535 | 161.9469865 | 326.061649 | 1.0096231 | 0 | 0 |
| MSMEG_3888 | 106 | 184 | 24.41069592 | 49.13182014 | 1.0091443 | 4.73E-09 | 1.49E-08 |
| MSMEG_4958 | 153 | 265 | 23.66261839 | 47.52126333 | 1.0059635 | 2.26E-12 | 8.95E-12 |
| MSMEG_0673 | 164 | 284 | 66.56259605 | 133.6520051 | 1.0056979 | 4.68E-13 | 2.01E-12 |
| MSMEG_1199 | 163 | 282 | 15.11915089 | 30.32910817 | 1.004326 | 6.66E-13 | 2.79E-12 |
| MSMEG_1803 | 1846 | 3189 | 975.6226517 | 1954.228904 | 1.0022043 | 3.84E-12 | 1.49E-11 |
| MSMEG_5976 | 66 | 114 | 14.1356178 | 28.31042862 | 1.0019987 | 4.66E-06 | 1.13E-05 |
| MSMEG_3388 | 44 | 76 | 7.770059142 | 15.56166189 | 1.0019987 | 0.0001868 | 0.0003534 |
| MSMEG_4114 | 124 | 214 | 35.1711768 | 70.38007457 | 1.0007734 | 3.54E-10 | 1.21E-09 |
| MSMEG_5524 | 645 | 1113 | 157.41938 | 314.9665128 | 1.0005853 | 5.57E-13 | 2.37E-12 |
| MSMEG_5247 | 1598 | 0 | 268.0202717 | 0 | -18.03198 | 0 | 0 |
| MSMEG_3291 | 35 | 0 | 44.33074495 | 0 | -15.43602 | 3.78E-10 | 1.29E-09 |
| MSMEG_0853 | 2491 | 40 | 2204.756409 | 41.0504207 | -5.747078 | 0 | 0 |
| MSMEG_0852 | 6354 | 111 | 3050.849318 | 61.79698136 | -5.625531 | 0 | 0 |
| MSMEG_0854 | 11850 | 454 | 2695.136754 | 119.7261573 | -4.492548 | 0 | 0 |
| MSMEG_0856 | 1553 | 86 | 528.180889 | 33.91410914 | -3.961075 | 8.00E-304 | 1.76E-302 |
| MSMEG_0851 | 1005 | 57 | 172.0971777 | 11.31757228 | -3.926587 | 6.46E-196 | 1.20E-194 |
| MSMEG_5405 | 131 | 11 | 81.5556901 | 7.940473326 | -3.360489 | 8.95E-24 | 5.93E-23 |
| MSMEG_0541 | 595 | 57 | 446.0215767 | 49.54325009 | -3.170353 | 1.18E-97 | 1.62E-96 |
| MSMEG_0063 | 2567 | 259 | 1924.264517 | 225.117575 | -3.095557 | 0 | 0 |
| MSMEG_0051 | 604 | 62 | 452.7681216 | 53.88914922 | -3.070706 | 3.57E-96 | 4.87E-95 |
| MSMEG_6192 | 29 | 3 | 39.45201746 | 4.732201275 | -3.059516 | 9.59E-06 | 2.21E-05 |
| MSMEG_3415 | 93 | 10 | 42.17284625 | 5.258001417 | -3.003728 | 1.10E-15 | 5.89E-15 |
| MSMEG_4486 | 1023 | 115 | 653.4957567 | 85.17962296 | -2.939598 | 6.21E-155 | 1.06E-153 |
| MSMEG_0586 | 35183 | 3963 | 19881.74479 | 2596.668044 | -2.936711 | 0 | 0 |
| MSMEG_6930 | 26 | 3 | 6.241901343 | 0.835094343 | -2.901974 | 4.67E-05 | 9.77E-05 |
| MSMEG_5787 | 34 | 4 | 40.94624311 | 5.585549046 | -2.87396 | 3.04E-06 | 7.55E-06 |
| MSMEG_0064 | 13604 | 1610 | 2313.384679 | 317.4518356 | -2.865396 | 0 | 0 |
| MSMEG_0584 | 4620 | 555 | 691.2345893 | 96.28246587 | -2.84383 | 0 | 0 |
| MSMEG_0465 | 699 | 86 | 250.4887891 | 35.73389061 | -2.809381 | 1.20E-101 | 1.70E-100 |
| MSMEG_1518 | 707 | 91 | 312.8789204 | 46.69485355 | -2.744269 | 2.58E-100 | 3.62E-99 |
| MSMEG_6383 | 4128 | 536 | 1969.173336 | 296.469337 | -2.731635 | 0 | 0 |
| MSMEG_4718 | 532 | 70 | 193.475172 | 29.51769112 | -2.712497 | 5.59E-75 | 6.66E-74 |
| MSMEG_0077 | 437 | 58 | 302.8590462 | 46.60771822 | -2.700006 | 1.39E-61 | 1.49E-60 |
| MSMEG_6253 | 2713 | 362 | 1285.828579 | 198.9356355 | -2.692325 | 0 | 0 |
| MSMEG_1126 | 446 | 60 | 344.8865294 | 53.7976566 | -2.680507 | 2.44E-62 | 2.62E-61 |
| MSMEG_5989 | 3457 | 468 | 688.2369607 | 108.0326925 | -2.671437 | 0 | 0 |
| MSMEG_1127 | 13638 | 1868 | 5034.572373 | 799.5755562 | -2.654563 | 0 | 0 |
| MSMEG_0842 | 500 | 69 | 386.6440913 | 61.86730509 | -2.643757 | 1.13E-68 | 1.29E-67 |
| MSMEG_6788 | 2214 | 313 | 284.3456354 | 46.61052795 | -2.608918 | 9.71E-294 | 2.13E-292 |
| MSMEG_0855 | 106 | 15 | 51.56961588 | 8.461551949 | -2.607527 | 1.78E-15 | 9.46E-15 |
| MSMEG_3566 | 314 | 45 | 109.3231587 | 18.1662703 | -2.589265 | 9.25E-43 | 8.19E-42 |
| MSMEG_5282 | 167 | 24 | 57.0614745 | 9.508423028 | -2.585239 | 2.16E-23 | 1.42E-22 |
| MSMEG_6931 | 100 | 15 | 35.14946284 | 6.113370069 | -2.523463 | 3.37E-14 | 1.69E-13 |
| MSMEG_6395 | 1007 | 154 | 468.2064176 | 83.02317681 | -2.495559 | 3.92E-128 | 6.17E-127 |
| MSMEG_0078 | 895 | 142 | 342.4418111 | 62.99742948 | -2.442494 | 2.47E-111 | 3.66E-110 |
| MSMEG_0055 | 1453 | 231 | 202.9293427 | 37.40778118 | -2.439567 | 2.30E-179 | 4.12E-178 |
| MSMEG_2544 | 323 | 52 | 79.62532846 | 14.86355837 | -2.421448 | 7.82E-41 | 6.78E-40 |
| MSMEG_1960 | 168 | 28 | 70.92919192 | 13.70706576 | -2.37146 | 1.87E-21 | 1.17E-20 |
| MSMEG_3537 | 30 | 5 | 27.5483915 | 5.323726435 | -2.37146 | 8.23E-05 | 0.0001654 |
| MSMEG_6005 | 143 | 24 | 49.31981202 | 9.597703995 | -2.361406 | 2.20E-18 | 1.29E-17 |
| MSMEG_1129 | 64573 | 10864 | 11187.9389 | 2182.526943 | -2.357873 | 0 | 0 |
| MSMEG_2157 | 1784 | 301 | 432.530961 | 84.61738122 | -2.353777 | 8.32E-211 | 1.62E-209 |
| MSMEG_5589 | 586 | 99 | 105.51214 | 20.66858498 | -2.351897 | 2.25E-70 | 2.58E-69 |
| MSMEG_0436 | 9430 | 1598 | 3283.17639 | 645.104443 | -2.347488 | 0 | 0 |
| MSMEG_0728 | 1020 | 173 | 727.4915038 | 143.0686871 | -2.346222 | 7.24E-121 | 1.11E-119 |
| MSMEG_0981 | 35 | 6 | 21.42652673 | 4.258981148 | -2.330818 | 2.39E-05 | 5.27E-05 |
| MSMEG_0364 | 728 | 127 | 534.8061071 | 108.1781212 | -2.305607 | 5.19E-85 | 6.56E-84 |
| MSMEG_5838 | 370 | 65 | 149.3465913 | 30.42129391 | -2.295511 | 8.58E-44 | 7.69E-43 |
| MSMEG_5344 | 187 | 33 | 178.4086307 | 36.5055527 | -2.288998 | 8.37E-23 | 5.40E-22 |
| MSMEG_1623 | 1347 | 239 | 145.7346425 | 29.98222369 | -2.281165 | 8.37E-154 | 1.42E-152 |
| MSMEG_0828 | 1720 | 309 | 353.9363838 | 73.72690054 | -2.263227 | 5.10E-194 | 9.39E-193 |
| MSMEG_2036 | 393 | 71 | 93.43273235 | 19.57201693 | -2.255136 | 1.83E-45 | 1.68E-44 |
| MSMEG_2192 | 170 | 31 | 93.19853842 | 19.70573367 | -2.241692 | 2.24E-20 | 1.36E-19 |
| MSMEG_5101 | 1097 | 202 | 290.9322309 | 62.11654815 | -2.227634 | 4.61E-122 | 7.13E-121 |
| MSMEG_3153 | 1133 | 212 | 285.0440874 | 61.84273995 | -2.204509 | 2.02E-124 | 3.15E-123 |
| MSMEG_0179 | 117 | 22 | 38.37097388 | 8.365855826 | -2.19743 | 3.86E-14 | 1.92E-13 |
| MSMEG_0065 | 3462 | 653 | 2518.086637 | 550.7157801 | -2.192948 | 0 | 0 |
| MSMEG_5204 | 115 | 22 | 33.13009174 | 7.348830216 | -2.172556 | 9.54E-14 | 4.52E-13 |
| MSMEG_1277 | 99 | 19 | 110.193566 | 24.52140661 | -2.167926 | 5.45E-12 | 2.08E-11 |
| MSMEG_0056 | 177 | 34 | 122.6683093 | 27.32176585 | -2.16664 | 2.37E-20 | 1.44E-19 |
| MSMEG_3678 | 134 | 26 | 91.99961275 | 20.69785231 | -2.152147 | 1.26E-15 | 6.71E-15 |
| MSMEG_5988 | 2458 | 483 | 663.8622188 | 151.2564628 | -2.133887 | 4.08E-257 | 8.44E-256 |
| MSMEG_4804 | 3862 | 759 | 2445.790529 | 557.3390847 | -2.133674 | 0 | 0 |
| MSMEG_1101 | 2702 | 532 | 510.2708061 | 116.4924406 | -2.131027 | 5.75E-282 | 1.23E-280 |
| MSMEG_4765 | 335 | 66 | 111.3569973 | 25.43825844 | -2.13012 | 1.93E-36 | 1.58E-35 |
| MSMEG_4174 | 327 | 65 | 90.30901275 | 20.81456952 | -2.117276 | 2.38E-35 | 1.92E-34 |
| MSMEG_4447 | 80 | 16 | 83.95700268 | 19.4696281 | -2.108425 | 1.21E-09 | 4.00E-09 |
| MSMEG_4487 | 533 | 107 | 285.8061834 | 66.52715078 | -2.103022 | 3.85E-56 | 3.88E-55 |
| MSMEG_3297 | 422 | 85 | 76.16983597 | 17.78935615 | -2.098205 | 1.15E-44 | 1.05E-43 |
| MSMEG_0977 | 417 | 84 | 336.6352896 | 78.62734427 | -2.098083 | 3.76E-44 | 3.39E-43 |
| MSMEG_2798 | 455 | 92 | 123.3408919 | 28.91706757 | -2.092658 | 7.11E-48 | 6.69E-47 |
| MSMEG_5007 | 1392 | 283 | 574.4922992 | 135.4260298 | -2.084782 | 3.09E-142 | 5.06E-141 |
| MSMEG_0542 | 840 | 171 | 262.5889233 | 61.98176819 | -2.08289 | 1.88E-86 | 2.39E-85 |
| MSMEG_1305 | 343 | 70 | 124.1260859 | 29.37228378 | -2.079279 | 3.36E-36 | 2.75E-35 |
| MSMEG_0052 | 563 | 115 | 159.0743017 | 37.67560246 | -2.077998 | 2.45E-58 | 2.53E-57 |
| MSMEG_5803 | 298 | 61 | 182.4315704 | 43.29964167 | -2.074928 | 1.42E-31 | 1.08E-30 |
| MSMEG_4720 | 1782 | 367 | 488.4699867 | 116.6452299 | -2.066143 | 1.66E-179 | 2.98E-178 |
| MSMEG_0066 | 4762 | 982 | 3644.04001 | 871.3165598 | -2.06427 | 0 | 0 |
| MSMEG_3284 | 87 | 18 | 45.65162021 | 10.95166581 | -2.059516 | 4.11E-10 | 1.40E-09 |
| MSMEG_5032 | 308 | 64 | 104.2691807 | 25.12210078 | -2.053284 | 3.61E-32 | 2.78E-31 |
| MSMEG_3613 | 404 | 84 | 145.4843159 | 35.07396239 | -2.052391 | 1.10E-41 | 9.68E-41 |
| MSMEG_2063 | 305 | 64 | 182.1628056 | 44.32110463 | -2.039163 | 1.33E-31 | 1.01E-30 |
| MSMEG_0595 | 867 | 182 | 258.910086 | 63.01907064 | -2.038591 | 8.52E-87 | 1.09E-85 |
| MSMEG_2445 | 1314 | 276 | 199.0300285 | 48.47335244 | -2.037722 | 1.52E-130 | 2.42E-129 |
| MSMEG_5039 | 261 | 55 | 84.83929419 | 20.72955426 | -2.033044 | 3.65E-27 | 2.56E-26 |
| MSMEG_4964 | 117 | 25 | 32.07126175 | 7.94586035 | -2.013006 | 8.25E-13 | 3.42E-12 |
| MSMEG_4898 | 1778 | 380 | 1044.928855 | 258.9460538 | -2.012681 | 3.29E-173 | 5.85E-172 |
| MSMEG_0434 | 4029 | 866 | 1402.748428 | 349.5997795 | -2.00448 | 0 | 0 |
| MSMEG_1476 | 8656 | 1869 | 1068.723258 | 267.5642274 | -1.997931 | 0 | 0 |
| MSMEG_5549 | 1524 | 331 | 273.0650319 | 68.7669639 | -1.989457 | 1.57E-146 | 2.60E-145 |
| MSMEG_0435 | 12230 | 2659 | 3045.575847 | 767.7715845 | -1.987966 | 0 | 0 |
| MSMEG_2632 | 210 | 46 | 74.52704948 | 18.9288051 | -1.977181 | 2.05E-21 | 1.28E-20 |
| MSMEG_2910 | 362 | 80 | 112.6838161 | 28.87444846 | -1.964415 | 1.57E-35 | 1.27E-34 |
| MSMEG_3315 | 95 | 21 | 71.21352906 | 18.25277635 | -1.964035 | 2.34E-10 | 8.07E-10 |
| MSMEG_5860 | 54 | 12 | 19.83484188 | 5.110777377 | -1.956422 | 2.15E-06 | 5.40E-06 |
| MSMEG_6898 | 117 | 26 | 44.76613619 | 11.53474061 | -1.956422 | 2.14E-12 | 8.52E-12 |
| MSMEG_5263 | 3062 | 682 | 1363.283633 | 352.0757749 | -1.953128 | 1.72E-285 | 3.71E-284 |
| MSMEG_5639 | 3158 | 705 | 950.7958511 | 246.1132549 | -1.949813 | 1.01E-293 | 2.20E-292 |
| MSMEG_4647 | 85 | 19 | 22.706553 | 5.885137586 | -1.947961 | 2.57E-09 | 8.28E-09 |
| MSMEG_1900 | 411 | 92 | 162.3281564 | 42.13185652 | -1.94593 | 1.19E-39 | 1.02E-38 |
| MSMEG_3244 | 2079 | 466 | 321.5332263 | 83.56569326 | -1.943985 | 2.74E-193 | 5.01E-192 |
| MSMEG_5281 | 437 | 98 | 204.4780822 | 53.16944618 | -1.943277 | 5.53E-42 | 4.87E-41 |
| MSMEG_6032 | 164 | 37 | 59.34891569 | 15.52535 | -1.934596 | 1.34E-16 | 7.41E-16 |
| MSMEG_4681 | 6537 | 1483 | 1759.060662 | 462.7156807 | -1.926607 | 0 | 0 |
| MSMEG_4811 | 163 | 37 | 77.25398392 | 20.33320032 | -1.925772 | 2.04E-16 | 1.12E-15 |
| MSMEG_5498 | 35 | 8 | 21.24944799 | 5.631710609 | -1.91578 | 0.0001866 | 0.000353 |
| MSMEG_3874 | 140 | 32 | 47.83596664 | 12.67789737 | -1.91578 | 3.30E-14 | 1.66E-13 |
| MSMEG_6677 | 3697 | 846 | 946.3080455 | 251.0869722 | -1.914123 | 0 | 0 |
| MSMEG_6258 | 218 | 50 | 77.36617518 | 20.57478815 | -1.910825 | 2.88E-21 | 1.79E-20 |
| MSMEG_5560 | 39 | 9 | 13.70829051 | 3.668022041 | -1.901974 | 8.37E-05 | 0.000168 |
| MSMEG_1402 | 1022 | 237 | 245.3547374 | 65.97245307 | -1.894933 | 8.09E-93 | 1.08E-91 |
| MSMEG_6231 | 210 | 49 | 61.21864779 | 16.56270446 | -1.886033 | 3.27E-20 | 1.98E-19 |
| MSMEG_2897 | 34 | 8 | 23.34318532 | 6.368569941 | -1.87396 | 0.0002866 | 0.0005303 |
| MSMEG_6223 | 2643 | 624 | 1021.900333 | 279.7478143 | -1.869056 | 9.41E-233 | 1.91E-231 |
| MSMEG_6565 | 1118 | 265 | 357.0910342 | 98.14173949 | -1.863353 | 3.81E-99 | 5.31E-98 |
| MSMEG_3804 | 734 | 174 | 634.369235 | 174.3676988 | -1.86319 | 1.09E-65 | 1.20E-64 |
| MSMEG_4029 | 333 | 79 | 121.1038201 | 33.31282284 | -1.862095 | 1.14E-30 | 8.62E-30 |
| MSMEG_2789 | 267 | 64 | 110.8161285 | 30.79941169 | -1.847193 | 1.22E-24 | 8.18E-24 |
| MSMEG_4659 | 413 | 99 | 123.3331782 | 34.27960436 | -1.847139 | 2.88E-37 | 2.39E-36 |
| MSMEG_6215 | 329 | 79 | 99.87240556 | 27.80657113 | -1.84466 | 5.90E-30 | 4.40E-29 |
| MSMEG_6722 | 435 | 105 | 106.5204472 | 29.81286803 | -1.837123 | 6.83E-39 | 5.77E-38 |
| MSMEG_1530 | 2017 | 488 | 411.5933753 | 115.4657111 | -1.833755 | 1.33E-173 | 2.37E-172 |
| MSMEG_2380 | 533 | 129 | 95.03749302 | 26.67031884 | -1.833262 | 3.71E-47 | 3.48E-46 |
| MSMEG_0986 | 789 | 191 | 238.5259906 | 66.95188471 | -1.83295 | 5.60E-69 | 6.40E-68 |
| MSMEG_0552 | 132 | 32 | 58.76990188 | 16.51968445 | -1.830891 | 9.14E-13 | 3.77E-12 |
| MSMEG_3132 | 613 | 149 | 168.0314825 | 47.35732769 | -1.827072 | 9.59E-54 | 9.54E-53 |
| MSMEG_6482 | 218 | 53 | 71.4946351 | 20.15410722 | -1.826761 | 4.14E-20 | 2.50E-19 |
| MSMEG_2682 | 654 | 159 | 180.6180255 | 50.91563929 | -1.826761 | 3.26E-57 | 3.33E-56 |
| MSMEG_0067 | 5096 | 1241 | 788.1353157 | 222.5429728 | -1.824359 | 0 | 0 |
| MSMEG_4745 | 2858 | 696 | 523.5797368 | 147.8429366 | -1.824344 | 1.81E-243 | 3.70E-242 |
| MSMEG_3070 | 4610 | 1123 | 1428.951728 | 403.6148379 | -1.823906 | 0 | 0 |
| MSMEG_6749 | 41 | 10 | 42.42193621 | 11.99712999 | -1.822121 | 8.78E-05 | 0.0001758 |
| MSMEG_5890 | 451 | 110 | 602.3914942 | 170.3592459 | -1.822121 | 7.78E-40 | 6.65E-39 |
| MSMEG_6202 | 2452 | 600 | 547.5068366 | 155.3427774 | -1.817422 | 4.29E-208 | 8.29E-207 |
| MSMEG_1130 | 125262 | 30679 | 24473.5221 | 6950.068225 | -1.816123 | 0 | 0 |
| MSMEG_6199 | 2457 | 603 | 1516.782026 | 431.624476 | -1.813165 | 7.46E-208 | 1.44E-206 |
| MSMEG_5796 | 2555 | 628 | 521.378817 | 148.59112 | -1.810984 | 1.03E-215 | 2.03E-214 |
| MSMEG_4474 | 10243 | 2518 | 1173.908161 | 334.6057576 | -1.810785 | 0 | 0 |
| MSMEG_0839 | 907 | 223 | 318.805628 | 90.88543502 | -1.810556 | 9.48E-78 | 1.15E-76 |
| MSMEG_0415 | 8025 | 1975 | 3639.108507 | 1038.45528 | -1.809146 | 0 | 0 |
| MSMEG_6071 | 666 | 164 | 155.320455 | 44.34748624 | -1.808324 | 1.86E-57 | 1.91E-56 |
| MSMEG_4890 | 3205 | 791 | 1322.735502 | 378.5229312 | -1.805072 | 6.98E-269 | 1.47E-267 |
| MSMEG_0098 | 738 | 183 | 227.7951029 | 65.49525631 | -1.798274 | 5.16E-63 | 5.55E-62 |
| MSMEG_3527 | 149 | 37 | 44.86022223 | 12.91658217 | -1.796212 | 6.63E-14 | 3.21E-13 |
| MSMEG_0092 | 3140 | 784 | 1088.074834 | 315.0038887 | -1.788336 | 3.77E-260 | 7.84E-259 |
| MSMEG_3029 | 52 | 13 | 25.81110555 | 7.481993908 | -1.786497 | 1.22E-05 | 2.79E-05 |
| MSMEG_2591 | 659 | 165 | 221.0580213 | 64.17642825 | -1.78431 | 7.20E-56 | 7.23E-55 |
| MSMEG_3199 | 1119 | 281 | 236.2195409 | 68.78009785 | -1.780065 | 2.39E-93 | 3.18E-92 |
| MSMEG_6438 | 382 | 96 | 172.1633629 | 50.1671399 | -1.778964 | 6.09E-33 | 4.73E-32 |
| MSMEG_6901 | 691 | 174 | 311.4263972 | 90.92794107 | -1.776096 | 3.86E-58 | 3.97E-57 |
| MSMEG_2794 | 638 | 161 | 212.0769084 | 62.05393347 | -1.772993 | 1.10E-53 | 1.09E-52 |
| MSMEG_0540 | 293 | 74 | 103.4830604 | 30.30428894 | -1.771801 | 1.71E-25 | 1.17E-24 |
| MSMEG_0120 | 162 | 41 | 54.09502332 | 15.87438428 | -1.768795 | 1.03E-14 | 5.34E-14 |
| MSMEG_3154 | 1395 | 354 | 706.7587338 | 207.9557691 | -1.764941 | 1.38E-114 | 2.06E-113 |
| MSMEG_4902 | 2374 | 603 | 689.3268135 | 203.017046 | -1.763587 | 1.85E-193 | 3.39E-192 |
| MSMEG_3667 | 7665 | 1947 | 1080.785264 | 318.3200113 | -1.76353 | 0 | 0 |
| MSMEG_2361 | 334 | 85 | 176.5211082 | 52.08825864 | -1.760811 | 1.29E-28 | 9.38E-28 |
| MSMEG_4891 | 6756 | 1720 | 2532.20317 | 747.4946504 | -1.760258 | 0 | 0 |
| MSMEG_2744 | 3871 | 988 | 1236.403751 | 365.9020325 | -1.756621 | 0 | 0 |
| MSMEG_3493 | 1782 | 455 | 1148.332951 | 339.9713021 | -1.756056 | 7.50E-145 | 1.24E-143 |
| MSMEG_1926 | 622 | 159 | 223.988229 | 66.39000024 | -1.754385 | 1.20E-51 | 1.18E-50 |
| MSMEG_2159 | 1003 | 257 | 258.5360157 | 76.8110986 | -1.750979 | 5.19E-82 | 6.42E-81 |
| MSMEG_3528 | 2084 | 534 | 588.8292092 | 174.945841 | -1.750941 | 2.13E-168 | 3.76E-167 |
| MSMEG_2341 | 35 | 9 | 18.76776064 | 5.595741654 | -1.745855 | 0.0004493 | 0.000811 |
| MSMEG_3223 | 1048 | 270 | 801.964286 | 239.5676896 | -1.743105 | 4.35E-85 | 5.51E-84 |
| MSMEG_6590 | 128 | 33 | 71.2362447 | 21.29490574 | -1.742103 | 1.07E-11 | 4.01E-11 |
| MSMEG_0988 | 21614 | 5583 | 5475.226979 | 1639.854603 | -1.739351 | 0 | 0 |
| MSMEG_5776 | 4714 | 1218 | 1552.922183 | 465.2411693 | -1.738935 | 0 | 0 |
| MSMEG_1383 | 1087 | 281 | 318.1418493 | 95.36045438 | -1.738207 | 7.52E-88 | 9.71E-87 |
| MSMEG_4492 | 1071 | 277 | 148.4494455 | 44.51840671 | -1.737498 | 1.55E-86 | 1.98E-85 |
| MSMEG_3675 | 626 | 162 | 153.2914941 | 45.9969964 | -1.736666 | 2.88E-51 | 2.82E-50 |
| MSMEG_6235 | 5447 | 1412 | 1762.773433 | 529.8397692 | -1.734219 | 0 | 0 |
| MSMEG_1124 | 81 | 21 | 51.74306578 | 15.55453984 | -1.73403 | 7.76E-08 | 2.23E-07 |
| MSMEG_1880 | 54 | 14 | 21.91695236 | 6.588479124 | -1.73403 | 1.25E-05 | 2.85E-05 |
| MSMEG_5793 | 420 | 109 | 328.2361541 | 98.77211598 | -1.732558 | 7.97E-35 | 6.37E-34 |
| MSMEG_4805 | 404 | 105 | 75.71122563 | 22.81597043 | -1.730463 | 1.66E-33 | 1.30E-32 |
| MSMEG_1957 | 4174 | 1085 | 652.408432 | 196.6380658 | -1.730233 | 0 | 0 |
| MSMEG_4684 | 796 | 207 | 365.4753273 | 110.2011372 | -1.729635 | 2.26E-64 | 2.47E-63 |
| MSMEG_6441 | 715 | 186 | 240.9431184 | 72.67619206 | -1.729138 | 5.65E-58 | 5.82E-57 |
| MSMEG_5777 | 69 | 18 | 29.47037231 | 8.914146588 | -1.725097 | 8.07E-07 | 2.13E-06 |
| MSMEG_3520 | 42 | 11 | 15.741938 | 4.780489043 | -1.719383 | 0.0001356 | 0.0002594 |
| MSMEG_5441 | 878 | 230 | 124.9999366 | 37.9676614 | -1.719084 | 3.09E-70 | 3.54E-69 |
| MSMEG_1317 | 351 | 92 | 112.1099759 | 34.07184918 | -1.718263 | 4.81E-29 | 3.52E-28 |
| MSMEG_1184 | 7791 | 2043 | 1953.397208 | 593.9316372 | -1.717617 | 0 | 0 |
| MSMEG_6886 | 183 | 48 | 111.1042566 | 33.79026365 | -1.717235 | 7.71E-16 | 4.17E-15 |
| MSMEG_5052 | 118 | 31 | 99.63862674 | 30.3513599 | -1.714944 | 1.11E-10 | 3.88E-10 |
| MSMEG_4708 | 251 | 66 | 91.73660057 | 27.96942843 | -1.713647 | 3.96E-21 | 2.46E-20 |
| MSMEG_4142 | 403 | 106 | 141.6523353 | 43.20114848 | -1.713213 | 5.51E-33 | 4.29E-32 |
| MSMEG_4183 | 1610 | 424 | 523.3381749 | 159.8060183 | -1.711422 | 1.67E-126 | 2.62E-125 |
| MSMEG_3276 | 1819 | 480 | 452.9764895 | 138.5973526 | -1.708536 | 2.39E-142 | 3.93E-141 |
| MSMEG_3836 | 269 | 71 | 129.1593432 | 39.52779889 | -1.708212 | 1.92E-22 | 1.22E-21 |
| MSMEG_0081 | 106 | 28 | 70.15326125 | 21.48675174 | -1.707063 | 1.12E-09 | 3.71E-09 |
| MSMEG_5063 | 624 | 165 | 305.6034898 | 93.69758525 | -1.705577 | 7.04E-50 | 6.79E-49 |
| MSMEG_5691 | 1964 | 521 | 550.6874393 | 169.3839067 | -1.700937 | 1.35E-152 | 2.26E-151 |
| MSMEG_3193 | 275 | 73 | 98.06870762 | 30.18501202 | -1.69996 | 9.11E-23 | 5.87E-22 |
| MSMEG_6674 | 158 | 42 | 38.94985108 | 12.00518176 | -1.697961 | 1.13E-13 | 5.31E-13 |
| MSMEG_6903 | 2584 | 693 | 1026.090719 | 319.0782633 | -1.685176 | 1.42E-197 | 2.64E-196 |
| MSMEG_5068 | 2807 | 753 | 542.6549821 | 168.7901476 | -1.684805 | 2.18E-214 | 4.28E-213 |
| MSMEG_6737 | 450 | 121 | 105.6168364 | 32.92886383 | -1.681415 | 9.73E-36 | 7.90E-35 |
| MSMEG_2642 | 468 | 126 | 373.6999195 | 116.6590488 | -1.679582 | 4.76E-37 | 3.94E-36 |
| MSMEG_0606 | 104 | 28 | 36.38136783 | 11.35728306 | -1.679582 | 2.45E-09 | 7.93E-09 |
| MSMEG_5238 | 837 | 227 | 295.6154319 | 92.9604539 | -1.669033 | 2.20E-64 | 2.41E-63 |
| MSMEG_2919 | 254 | 69 | 55.86659834 | 17.59698798 | -1.666657 | 1.32E-20 | 8.10E-20 |
| MSMEG_3121 | 16312 | 4434 | 4249.355671 | 1339.313646 | -1.66575 | 0 | 0 |
| MSMEG_4242 | 423 | 115 | 230.1821157 | 72.56041955 | -1.665521 | 2.91E-33 | 2.27E-32 |
| MSMEG_5116 | 253 | 69 | 112.6423119 | 35.6205696 | -1.660966 | 1.94E-20 | 1.18E-19 |
| MSMEG_0127 | 1893 | 519 | 382.0447261 | 121.4511657 | -1.653365 | 1.90E-141 | 3.10E-140 |
| MSMEG_2643 | 798 | 219 | 311.8243464 | 99.22519908 | -1.651955 | 1.33E-60 | 1.41E-59 |
| MSMEG_5173 | 881 | 242 | 231.9725966 | 73.88340056 | -1.650632 | 1.19E-66 | 1.34E-65 |
| MSMEG_2158 | 447 | 123 | 232.8913665 | 74.30562854 | -1.648114 | 1.45E-34 | 1.15E-33 |
| MSMEG_6394 | 1755 | 483 | 381.439267 | 121.7211772 | -1.647873 | 1.15E-130 | 1.84E-129 |
| MSMEG_3807 | 178 | 49 | 65.05623466 | 20.76518172 | -1.647521 | 1.18E-14 | 6.11E-14 |
| MSMEG_5738 | 264 | 73 | 167.1902381 | 53.60441789 | -1.641067 | 6.29E-21 | 3.87E-20 |
| MSMEG_1676 | 4838 | 1338 | 968.4222932 | 310.5458733 | -1.64083 | 0 | 0 |
| MSMEG_3881 | 3510 | 971 | 685.7791077 | 219.9718455 | -1.640425 | 1.03E-257 | 2.13E-256 |
| MSMEG_1542 | 3084 | 854 | 534.334839 | 171.5646179 | -1.638992 | 2.11E-226 | 4.19E-225 |
| MSMEG_0916 | 981 | 272 | 306.6663497 | 98.59088274 | -1.637144 | 3.54E-73 | 4.16E-72 |
| MSMEG_6098 | 894 | 248 | 207.8334346 | 66.84983067 | -1.636432 | 8.38E-67 | 9.43E-66 |
| MSMEG_0387 | 1129 | 314 | 338.5266287 | 109.1689862 | -1.632706 | 1.25E-83 | 1.55E-82 |
| MSMEG_6627 | 115 | 32 | 37.7150598 | 12.16851757 | -1.631987 | 7.78E-10 | 2.60E-09 |
| MSMEG_1601 | 4716 | 1313 | 2528.82169 | 816.3565324 | -1.631194 | 0 | 0 |
| MSMEG_1517 | 1659 | 462 | 231.2601215 | 74.67359735 | -1.630846 | 7.82E-122 | 1.20E-120 |
| MSMEG_6913 | 642 | 179 | 282.412253 | 91.30031443 | -1.629111 | 3.71E-48 | 3.52E-47 |
| MSMEG_5873 | 1106 | 309 | 297.6168108 | 96.41210071 | -1.62617 | 1.66E-81 | 2.05E-80 |
| MSMEG_5871 | 1220 | 341 | 626.7419606 | 203.1206394 | -1.625535 | 1.13E-89 | 1.48E-88 |
| MSMEG_4475 | 757 | 212 | 347.5688728 | 112.8630004 | -1.622726 | 3.57E-56 | 3.59E-55 |
| MSMEG_2418 | 989 | 277 | 314.5207411 | 102.141799 | -1.622582 | 7.56E-73 | 8.86E-72 |
| MSMEG_4717 | 3927 | 1100 | 561.2582798 | 182.2910219 | -1.622421 | 1.29E-283 | 2.77E-282 |
| MSMEG_0609 | 414 | 116 | 104.1555624 | 33.83848035 | -1.622003 | 1.91E-31 | 1.45E-30 |
| MSMEG_6127 | 1216 | 341 | 744.4187571 | 242.0520952 | -1.620797 | 5.09E-89 | 6.61E-88 |
| MSMEG_2942 | 228 | 64 | 116.3154308 | 37.8576102 | -1.619387 | 5.83E-18 | 3.38E-17 |
| MSMEG_4953 | 431 | 121 | 156.7439833 | 51.02343751 | -1.619178 | 1.35E-32 | 1.04E-31 |
| MSMEG_2735 | 1162 | 327 | 292.3400085 | 95.38950927 | -1.615745 | 8.93E-85 | 1.13E-83 |
| MSMEG_0069 | 1898 | 535 | 595.8615051 | 194.7482833 | -1.613366 | 5.17E-137 | 8.39E-136 |
| MSMEG_6022 | 39 | 11 | 7.271656641 | 2.378111301 | -1.612468 | 0.0004476 | 0.0008083 |
| MSMEG_0762 | 3220 | 910 | 588.4299877 | 192.8195445 | -1.609619 | 2.57E-230 | 5.15E-229 |
| MSMEG_1175 | 92 | 26 | 56.32115596 | 18.45558497 | -1.609619 | 5.38E-08 | 1.57E-07 |
| MSMEG_3776 | 329 | 93 | 70.25907601 | 23.02821202 | -1.609282 | 4.81E-25 | 3.28E-24 |
| MSMEG_3780 | 134 | 38 | 37.71631634 | 12.40163093 | -1.604659 | 5.25E-11 | 1.88E-10 |
| MSMEG_6176 | 67 | 19 | 23.55014011 | 7.743602087 | -1.604659 | 3.88E-06 | 9.48E-06 |
| MSMEG_0906 | 379 | 108 | 58.12576412 | 19.20542647 | -1.597664 | 2.46E-28 | 1.77E-27 |
| MSMEG_1125 | 235 | 67 | 79.19109484 | 26.17905843 | -1.596925 | 3.93E-18 | 2.29E-17 |
| MSMEG_6381 | 788 | 225 | 95.21110748 | 31.52206442 | -1.594768 | 5.21E-57 | 5.28E-56 |
| MSMEG_2575 | 970 | 277 | 838.335365 | 277.5853595 | -1.594596 | 9.16E-70 | 1.05E-68 |
| MSMEG_5794 | 4097 | 1172 | 2388.693333 | 792.3056992 | -1.592093 | 5.97E-288 | 1.30E-286 |
| MSMEG_2195 | 226 | 65 | 70.95084308 | 23.66100638 | -1.584308 | 2.60E-17 | 1.48E-16 |
| MSMEG_1111 | 333 | 96 | 104.9912947 | 35.09546697 | -1.580913 | 9.91E-25 | 6.68E-24 |
| MSMEG_4497 | 763 | 220 | 160.6068594 | 53.69489126 | -1.580677 | 1.42E-54 | 1.41E-53 |
| MSMEG_3353 | 52 | 15 | 31.31183297 | 10.47290446 | -1.580046 | 6.03E-05 | 0.0001245 |
| MSMEG_1944 | 707 | 204 | 399.5223137 | 133.6664853 | -1.579638 | 1.20E-50 | 1.16E-49 |
| MSMEG_2804 | 584 | 169 | 117.5398038 | 39.43933227 | -1.575442 | 5.67E-42 | 4.99E-41 |
| MSMEG_2782 | 1548 | 449 | 490.1713799 | 164.8519427 | -1.572115 | 4.65E-108 | 6.75E-107 |
| MSMEG_0958 | 131 | 38 | 72.90584418 | 24.52140661 | -1.571993 | 1.62E-10 | 5.64E-10 |
| MSMEG_0215 | 1382 | 401 | 424.790818 | 142.9164385 | -1.571581 | 1.27E-96 | 1.74E-95 |
| MSMEG_4192 | 1888 | 549 | 707.637594 | 238.5898623 | -1.568478 | 6.04E-131 | 9.65E-130 |
| MSMEG_3363 | 79 | 23 | 28.87327269 | 9.74692203 | -1.566716 | 7.96E-07 | 2.10E-06 |
| MSMEG_0316 | 312 | 91 | 57.87944882 | 19.57410528 | -1.564105 | 6.19E-23 | 4.03E-22 |
| MSMEG_2587 | 1887 | 551 | 484.6975736 | 164.1047981 | -1.562467 | 3.67E-130 | 5.82E-129 |
| MSMEG_0431 | 178 | 52 | 56.36337572 | 19.09198446 | -1.561791 | 1.11E-13 | 5.20E-13 |
| MSMEG_5924 | 263 | 77 | 91.56684949 | 31.08450696 | -1.55863 | 1.78E-19 | 1.06E-18 |
| MSMEG_1103 | 157 | 46 | 35.81861256 | 12.16851757 | -1.557556 | 3.44E-12 | 1.34E-11 |
| MSMEG_0625 | 464 | 136 | 85.21635772 | 28.9610718 | -1.557015 | 3.85E-33 | 3.00E-32 |
| MSMEG_2658 | 880 | 258 | 219.887388 | 74.74946504 | -1.55663 | 2.42E-61 | 2.57E-60 |
| MSMEG_2211 | 457 | 134 | 119.9010945 | 40.76453384 | -1.556458 | 1.19E-32 | 9.25E-32 |
| MSMEG_2786 | 1232 | 363 | 170.7653753 | 58.34000591 | -1.549458 | 1.30E-84 | 1.63E-83 |
| MSMEG_4369 | 414 | 122 | 221.9957972 | 75.85338687 | -1.549247 | 1.54E-29 | 1.14E-28 |
| MSMEG_4747 | 105 | 31 | 142.8435115 | 48.89941318 | -1.546546 | 1.61E-08 | 4.88E-08 |
| MSMEG_5667 | 893 | 264 | 126.1575057 | 43.24503935 | -1.544619 | 1.52E-61 | 1.61E-60 |
| MSMEG_3630 | 7155 | 2117 | 3864.877279 | 1325.921043 | -1.543428 | 0 | 0 |
| MSMEG_6457 | 152 | 45 | 66.46596046 | 22.81597043 | -1.542572 | 1.06E-11 | 3.98E-11 |
| MSMEG_0118 | 152 | 45 | 66.86396022 | 22.95259301 | -1.542572 | 1.06E-11 | 3.98E-11 |
| MSMEG_3168 | 496 | 147 | 193.8156338 | 66.60321582 | -1.541021 | 8.17E-35 | 6.52E-34 |
| MSMEG_5505 | 529 | 157 | 199.2902442 | 68.58051694 | -1.539 | 5.90E-37 | 4.88E-36 |
| MSMEG_4558 | 1405 | 417 | 486.8615102 | 167.5467112 | -1.538948 | 2.63E-95 | 3.54E-94 |
| MSMEG_4555 | 245 | 73 | 69.76078469 | 24.10121115 | -1.533311 | 7.50E-18 | 4.34E-17 |
| MSMEG_4288 | 708 | 211 | 329.1858428 | 113.7525345 | -1.533004 | 1.24E-48 | 1.18E-47 |
| MSMEG_0949 | 550 | 164 | 136.0414395 | 47.03521268 | -1.532233 | 4.02E-38 | 3.36E-37 |
| MSMEG_1366 | 8081 | 2410 | 1644.458369 | 568.6506685 | -1.531998 | 0 | 0 |
| MSMEG_1575 | 570 | 170 | 107.36809 | 37.12957924 | -1.531924 | 1.94E-39 | 1.65E-38 |
| MSMEG_4309 | 392 | 117 | 175.5929995 | 60.76838955 | -1.530842 | 1.35E-27 | 9.54E-27 |
| MSMEG_4272 | 7770 | 2321 | 4796.661109 | 1661.360545 | -1.529665 | 0 | 0 |
| MSMEG_0924 | 1633 | 488 | 449.3036038 | 155.6841049 | -1.529069 | 1.86E-109 | 2.74E-108 |
| MSMEG_0987 | 31401 | 9385 | 18603.16219 | 6446.860979 | -1.528879 | 0 | 0 |
| MSMEG_4237 | 6654 | 1992 | 3647.900439 | 1266.252305 | -1.526501 | 0 | 0 |
| MSMEG_3224 | 420 | 126 | 223.5811484 | 77.77269922 | -1.523463 | 2.94E-29 | 2.16E-28 |
| MSMEG_4296 | 1936 | 581 | 278.3232144 | 96.84806446 | -1.522966 | 1.13E-128 | 1.79E-127 |
| MSMEG_0562 | 953 | 286 | 212.7952754 | 74.04672391 | -1.522958 | 2.91E-64 | 3.17E-63 |
| MSMEG_5568 | 1326 | 398 | 383.5083164 | 133.4704328 | -1.522738 | 1.07E-88 | 1.39E-87 |
| MSMEG_0737 | 672 | 202 | 190.6050872 | 66.43352833 | -1.520603 | 1.02E-45 | 9.41E-45 |
| MSMEG_0233 | 7451 | 2241 | 1577.429895 | 550.1081702 | -1.519789 | 0 | 0 |
| MSMEG_5259 | 1413 | 425 | 157.7543149 | 55.01723367 | -1.519724 | 3.97E-94 | 5.33E-93 |
| MSMEG_6923 | 289 | 87 | 82.28925214 | 28.72336123 | -1.518479 | 1.61E-20 | 9.81E-20 |
| MSMEG_3637 | 2109 | 635 | 335.3509823 | 117.0758887 | -1.518228 | 2.27E-139 | 3.71E-138 |
| MSMEG_1559 | 993 | 299 | 160.678724 | 56.09847415 | -1.518145 | 1.39E-66 | 1.56E-65 |
| MSMEG_4479 | 488 | 147 | 398.3293349 | 139.1267175 | -1.517562 | 1.48E-33 | 1.17E-32 |
| MSMEG_3822 | 199 | 60 | 59.66944119 | 20.86031583 | -1.516231 | 1.48E-14 | 7.64E-14 |
| MSMEG_0909 | 1024 | 309 | 265.8143972 | 93.0053127 | -1.515034 | 2.09E-68 | 2.37E-67 |
| MSMEG_6384 | 95554 | 28878 | 9485.978385 | 3324.077232 | -1.512843 | 0 | 0 |
| MSMEG_5307 | 327 | 99 | 100.9336025 | 35.43185997 | -1.510287 | 7.52E-23 | 4.85E-22 |
| MSMEG_1631 | 317 | 96 | 81.71078463 | 28.69208352 | -1.509874 | 3.44E-22 | 2.18E-21 |
| MSMEG_5224 | 4250 | 1289 | 991.1590594 | 348.5604254 | -1.507708 | 5.04E-276 | 1.07E-274 |
| MSMEG_3408 | 438 | 133 | 162.5076832 | 57.21661542 | -1.506002 | 5.81E-30 | 4.35E-29 |
| MSMEG_2767 | 135 | 41 | 42.38214078 | 14.92463479 | -1.505761 | 3.16E-10 | 1.08E-09 |
| MSMEG_4692 | 2604 | 791 | 1210.734371 | 426.4372263 | -1.505477 | 1.55E-169 | 2.75E-168 |
| MSMEG_5352 | 551 | 168 | 389.2093261 | 137.5978525 | -1.500088 | 4.51E-37 | 3.74E-36 |
| MSMEG_2421 | 267 | 82 | 140.1032482 | 49.89092202 | -1.489641 | 1.39E-18 | 8.15E-18 |
| MSMEG_2849 | 179 | 55 | 98.13257869 | 34.96178554 | -1.488953 | 6.34E-13 | 2.67E-12 |
| MSMEG_5082 | 335 | 103 | 117.1899829 | 41.77857697 | -1.488014 | 6.69E-23 | 4.33E-22 |
| MSMEG_5569 | 1112 | 342 | 319.1022016 | 113.7946525 | -1.487586 | 3.26E-72 | 3.81E-71 |
| MSMEG_2784 | 4184 | 1287 | 2227.294107 | 794.3925706 | -1.487368 | 2.00E-266 | 4.21E-265 |
| MSMEG_6174 | 328 | 101 | 92.32053551 | 32.96222957 | -1.485838 | 2.07E-22 | 1.32E-21 |
| MSMEG_5651 | 1405 | 433 | 367.3118867 | 131.2554332 | -1.484628 | 1.67E-90 | 2.19E-89 |
| MSMEG_0057 | 1101 | 340 | 285.8023938 | 102.3359428 | -1.481705 | 4.13E-71 | 4.78E-70 |
| MSMEG_5892 | 9862 | 3046 | 1437.472154 | 514.7958959 | -1.481462 | 0 | 0 |
| MSMEG_3454 | 1107 | 342 | 893.6577112 | 320.1256159 | -1.481084 | 1.91E-71 | 2.22E-70 |
| MSMEG_0220 | 123 | 38 | 32.15612959 | 11.51895257 | -1.481084 | 3.04E-09 | 9.75E-09 |
| MSMEG_0834 | 401 | 124 | 185.2730397 | 66.42939149 | -1.479759 | 6.70E-27 | 4.67E-26 |
| MSMEG_5479 | 236 | 73 | 162.0291687 | 58.11320071 | -1.479316 | 1.92E-16 | 1.06E-15 |
| MSMEG_3123 | 4374 | 1353 | 795.3575211 | 285.2674006 | -1.479289 | 3.26E-276 | 6.95E-275 |
| MSMEG_5340 | 158 | 49 | 57.46067139 | 20.66238379 | -1.475568 | 1.95E-11 | 7.16E-11 |
| MSMEG_0755 | 877 | 272 | 216.1963253 | 77.74784377 | -1.475467 | 1.18E-56 | 1.19E-55 |
| MSMEG_0935 | 1985 | 616 | 587.995238 | 211.5751925 | -1.474634 | 7.49E-126 | 1.17E-124 |
| MSMEG_0923 | 696 | 216 | 163.877611 | 58.9705082 | -1.474553 | 2.83E-45 | 2.59E-44 |
| MSMEG_3406 | 103 | 32 | 38.2152771 | 13.76640371 | -1.472998 | 6.73E-08 | 1.96E-07 |
| MSMEG_5206 | 45 | 14 | 16.4468009 | 5.932909062 | -1.470995 | 0.0004074 | 0.0007399 |
| MSMEG_5489 | 90 | 28 | 113.9933442 | 41.12119729 | -1.470995 | 4.75E-07 | 1.27E-06 |
| MSMEG_5339 | 4575 | 1424 | 938.799934 | 338.8150366 | -1.47032 | 3.07E-286 | 6.65E-285 |
| MSMEG_0101 | 372 | 116 | 147.7189426 | 53.40992575 | -1.467675 | 8.75E-25 | 5.90E-24 |
| MSMEG_5696 | 372 | 116 | 182.1866958 | 65.87224175 | -1.467675 | 8.75E-25 | 5.91E-24 |
| MSMEG_0864 | 218 | 68 | 88.97110145 | 32.17896867 | -1.467219 | 3.93E-15 | 2.06E-14 |
| MSMEG_3976 | 125 | 39 | 42.31703764 | 15.30878016 | -1.466879 | 2.93E-09 | 9.41E-09 |
| MSMEG_2750 | 7036 | 2197 | 2237.581329 | 810.1282755 | -1.465718 | 0 | 0 |
| MSMEG_3514 | 432 | 135 | 417.5756186 | 151.3059092 | -1.464569 | 1.91E-28 | 1.38E-27 |
| MSMEG_5375 | 255 | 80 | 80.39873915 | 29.24622247 | -1.458923 | 2.54E-17 | 1.45E-16 |
| MSMEG_2582 | 204 | 64 | 83.25736099 | 30.28608816 | -1.458923 | 3.81E-14 | 1.90E-13 |
| MSMEG_6300 | 261 | 82 | 84.83929419 | 30.9058809 | -1.456851 | 1.16E-17 | 6.71E-17 |
| MSMEG_1201 | 70 | 22 | 22.65359654 | 8.255293855 | -1.456349 | 1.08E-05 | 2.49E-05 |
| MSMEG_6605 | 70 | 22 | 25.84103726 | 9.416842739 | -1.456349 | 1.08E-05 | 2.49E-05 |
| MSMEG_2434 | 426 | 134 | 215.8273983 | 78.71772052 | -1.455118 | 8.07E-28 | 5.72E-27 |
| MSMEG_4188 | 1748 | 550 | 433.8251203 | 158.2729481 | -1.454699 | 7.22E-109 | 1.05E-107 |
| MSMEG_0257 | 1023 | 322 | 148.8158654 | 54.31255167 | -1.454171 | 1.97E-64 | 2.16E-63 |
| MSMEG_3060 | 54 | 17 | 17.70968025 | 6.464524956 | -1.453922 | 0.0001188 | 0.0002323 |
| MSMEG_0921 | 165 | 52 | 89.78735009 | 32.80992884 | -1.45238 | 1.22E-11 | 4.55E-11 |
| MSMEG_5308 | 3265 | 1030 | 650.0126342 | 237.7642592 | -1.450936 | 6.67E-201 | 1.26E-199 |
| MSMEG_6685 | 786 | 248 | 128.0297752 | 46.83934921 | -1.450686 | 9.84E-50 | 9.48E-49 |
| MSMEG_4973 | 453 | 143 | 259.9879448 | 95.16161002 | -1.449993 | 2.45E-29 | 1.81E-28 |
| MSMEG_5849 | 810 | 256 | 104.7614888 | 38.39081598 | -1.448275 | 4.46E-51 | 4.35E-50 |
| MSMEG_3465 | 1601 | 506 | 189.6988163 | 69.51756325 | -1.448261 | 3.18E-99 | 4.44E-98 |
| MSMEG_0989 | 1455 | 460 | 1303.509257 | 477.8369093 | -1.447811 | 2.65E-90 | 3.47E-89 |
| MSMEG_0699 | 1441 | 457 | 171.5709656 | 63.09090387 | -1.443302 | 4.80E-89 | 6.25E-88 |
| MSMEG_3882 | 435 | 138 | 236.7121048 | 87.07250347 | -1.442844 | 4.87E-28 | 3.46E-27 |
| MSMEG_1060 | 8668 | 2750 | 4716.828791 | 1735.140468 | -1.442765 | 0 | 0 |
| MSMEG_6506 | 526 | 167 | 210.0065787 | 77.30976649 | -1.441712 | 1.56E-33 | 1.22E-32 |
| MSMEG_4244 | 3083 | 979 | 487.063461 | 179.3351632 | -1.441451 | 5.48E-188 | 9.94E-187 |
| MSMEG_0227 | 148 | 47 | 57.22332551 | 21.07074884 | -1.441362 | 1.77E-10 | 6.16E-10 |
| MSMEG_1852 | 1230 | 391 | 272.987082 | 100.620038 | -1.439915 | 5.18E-76 | 6.20E-75 |
| MSMEG_0068 | 2210 | 704 | 317.7139999 | 117.3511831 | -1.436896 | 1.11E-134 | 1.79E-133 |
| MSMEG_1332 | 727 | 232 | 186.7382809 | 69.09675708 | -1.434328 | 2.21E-45 | 2.03E-44 |
| MSMEG_3014 | 194 | 62 | 112.2181197 | 41.58375294 | -1.432214 | 3.41E-13 | 1.50E-12 |
| MSMEG_0926 | 122 | 39 | 54.64884168 | 20.25612985 | -1.431832 | 8.50E-09 | 2.63E-08 |
| MSMEG_1475 | 469 | 150 | 105.3634709 | 39.07322154 | -1.431123 | 9.21E-30 | 6.83E-29 |
| MSMEG_2788 | 234 | 75 | 49.68264826 | 18.4637911 | -1.428043 | 1.47E-15 | 7.82E-15 |
| MSMEG_0889 | 14147 | 4538 | 2037.78873 | 757.9316254 | -1.426865 | 0 | 0 |
| MSMEG_5768 | 140 | 45 | 52.74221963 | 19.65683607 | -1.423927 | 7.89E-10 | 2.63E-09 |
| MSMEG_5430 | 448 | 144 | 113.0967184 | 42.15074126 | -1.423927 | 2.68E-28 | 1.92E-27 |
| MSMEG_6054 | 712 | 229 | 199.6382163 | 74.45089182 | -1.423027 | 5.60E-44 | 5.03E-43 |
| MSMEG_0545 | 87 | 28 | 36.3137888 | 13.55130365 | -1.422086 | 1.38E-06 | 3.56E-06 |
| MSMEG_6807 | 87 | 28 | 31.63973678 | 11.80707645 | -1.422086 | 1.38E-06 | 3.56E-06 |
| MSMEG_0129 | 447 | 144 | 226.4667771 | 84.59217728 | -1.420703 | 3.78E-28 | 2.69E-27 |
| MSMEG_5083 | 239 | 77 | 137.1680327 | 51.24086693 | -1.420578 | 9.49E-16 | 5.10E-15 |
| MSMEG_0766 | 1179 | 380 | 185.4649741 | 69.31104223 | -1.41999 | 1.82E-71 | 2.11E-70 |
| MSMEG_0146 | 1013 | 327 | 272.5911658 | 102.0283396 | -1.417769 | 1.63E-61 | 1.73E-60 |
| MSMEG_5292 | 362 | 117 | 260.7194176 | 97.7060381 | -1.415978 | 5.52E-23 | 3.60E-22 |
| MSMEG_6556 | 99 | 32 | 35.47695296 | 13.29633139 | -1.415854 | 2.79E-07 | 7.66E-07 |
| MSMEG_5170 | 1457 | 471 | 723.2073229 | 271.0783947 | -1.415699 | 1.67E-87 | 2.15E-86 |
| MSMEG_2645 | 429 | 139 | 161.6172302 | 60.71778252 | -1.41239 | 7.43E-27 | 5.16E-26 |
| MSMEG_0365 | 2240 | 727 | 262.4493226 | 98.76488977 | -1.409969 | 1.01E-132 | 1.62E-131 |
| MSMEG_4784 | 77 | 25 | 33.27413562 | 12.52641514 | -1.409428 | 6.59E-06 | 1.56E-05 |
| MSMEG_5219 | 80 | 26 | 31.59672144 | 11.90682902 | -1.407986 | 4.40E-06 | 1.07E-05 |
| MSMEG_0938 | 749 | 244 | 206.0798526 | 77.84205244 | -1.404582 | 2.59E-45 | 2.37E-44 |
| MSMEG_2840 | 89 | 29 | 28.06073641 | 10.60175565 | -1.40425 | 1.32E-06 | 3.39E-06 |
| MSMEG_2402 | 850 | 277 | 112.5099473 | 42.51307308 | -1.404074 | 3.62E-51 | 3.54E-50 |
| MSMEG_5665 | 254 | 83 | 160.8572745 | 60.94748884 | -1.400142 | 2.55E-16 | 1.39E-15 |
| MSMEG_3806 | 997 | 326 | 185.42276 | 70.30014452 | -1.399219 | 2.04E-59 | 2.13E-58 |
| MSMEG_6173 | 862 | 282 | 215.3896914 | 81.70290365 | -1.39849 | 1.44E-51 | 1.42E-50 |
| MSMEG_5266 | 1742 | 570 | 520.2091924 | 197.367419 | -1.398208 | 2.32E-102 | 3.29E-101 |
| MSMEG_5697 | 822 | 269 | 440.7742641 | 167.2505005 | -1.398029 | 3.14E-49 | 3.01E-48 |
| MSMEG_5804 | 238 | 78 | 75.36226641 | 28.63797668 | -1.395913 | 2.54E-15 | 1.35E-14 |
| MSMEG_0952 | 4590 | 1508 | 746.0006903 | 284.1833438 | -1.392355 | 7.52E-265 | 1.58E-263 |
| MSMEG_3819 | 785 | 258 | 316.8569572 | 120.7491358 | -1.391819 | 8.71E-47 | 8.14E-46 |
| MSMEG_2420 | 2008 | 660 | 569.5461533 | 217.0600431 | -1.391719 | 6.78E-117 | 1.02E-115 |
| MSMEG_2585 | 666 | 219 | 249.6221598 | 95.17519096 | -1.391089 | 6.44E-40 | 5.52E-39 |
| MSMEG_6620 | 371 | 122 | 252.3568703 | 96.22142593 | -1.391035 | 6.12E-23 | 3.99E-22 |
| MSMEG_6053 | 386 | 127 | 128.8930803 | 49.17187325 | -1.39027 | 8.71E-24 | 5.77E-23 |
| MSMEG_5662 | 1623 | 534 | 503.0777993 | 191.9237074 | -1.390249 | 1.08E-94 | 1.45E-93 |
| MSMEG_3491 | 881 | 290 | 387.5470326 | 147.9167105 | -1.389586 | 3.54E-52 | 3.50E-51 |
| MSMEG_3227 | 10206 | 3362 | 1585.109986 | 605.4416329 | -1.388523 | 0 | 0 |
| MSMEG_3087 | 692 | 228 | 643.4932294 | 245.8348612 | -1.388235 | 2.74E-41 | 2.39E-40 |
| MSMEG_2026 | 2114 | 697 | 560.6478906 | 214.3328419 | -1.387242 | 2.23E-122 | 3.46E-121 |
| MSMEG_4217 | 8289 | 2734 | 2230.511523 | 853.0442826 | -1.386682 | 0 | 0 |
| MSMEG_6409 | 970 | 320 | 269.9185834 | 103.2480278 | -1.38641 | 4.41E-57 | 4.48E-56 |
| MSMEG_5284 | 639 | 211 | 137.6611705 | 52.70645291 | -1.38507 | 3.97E-38 | 3.33E-37 |
| MSMEG_5784 | 1099 | 363 | 308.1494378 | 118.0160425 | -1.384647 | 2.55E-64 | 2.78E-63 |
| MSMEG_2363 | 112 | 37 | 37.39902847 | 14.32566386 | -1.384399 | 7.59E-08 | 2.19E-07 |
| MSMEG_6110 | 811 | 268 | 307.1030311 | 117.6708193 | -1.383966 | 6.96E-48 | 6.56E-47 |
| MSMEG_6188 | 139 | 46 | 104.1966373 | 39.982272 | -1.381876 | 2.12E-09 | 6.88E-09 |
| MSMEG_6356 | 296 | 98 | 103.056226 | 39.56209976 | -1.381241 | 2.00E-18 | 1.17E-17 |
| MSMEG_3862 | 634 | 210 | 275.5925872 | 105.8445019 | -1.380591 | 1.15E-37 | 9.60E-37 |
| MSMEG_5050 | 329 | 109 | 94.78087116 | 36.41011334 | -1.380257 | 2.71E-20 | 1.64E-19 |
| MSMEG_5567 | 479 | 159 | 175.0670584 | 67.38089577 | -1.377496 | 9.27E-29 | 6.74E-28 |
| MSMEG_1136 | 2144 | 712 | 900.0190687 | 346.5593803 | -1.376853 | 1.05E-122 | 1.63E-121 |
| MSMEG_3389 | 686 | 228 | 93.32442752 | 35.96472969 | -1.375672 | 2.08E-40 | 1.80E-39 |
| MSMEG_6418 | 474 | 158 | 111.9651668 | 43.27453514 | -1.37146 | 2.68E-28 | 1.92E-27 |
| MSMEG_2929 | 207 | 69 | 72.41291481 | 27.9875904 | -1.37146 | 3.41E-13 | 1.50E-12 |
| MSMEG_3526 | 48 | 16 | 16.71182044 | 6.459118328 | -1.37146 | 0.0005246 | 0.0009417 |
| MSMEG_2757 | 240 | 80 | 144.5161522 | 55.85549046 | -1.37146 | 4.53E-15 | 2.38E-14 |
| MSMEG_1482 | 111 | 37 | 26.73548815 | 10.33326574 | -1.37146 | 1.07E-07 | 3.04E-07 |
| MSMEG_5175 | 431 | 144 | 131.3787744 | 50.89570832 | -1.368116 | 8.76E-26 | 6.05E-25 |
| MSMEG_3205 | 1053 | 352 | 173.4436846 | 67.226967 | -1.367355 | 1.43E-60 | 1.50E-59 |
| MSMEG_6257 | 1259 | 421 | 219.1685618 | 84.97777551 | -1.366883 | 4.87E-72 | 5.68E-71 |
| MSMEG_6380 | 245 | 82 | 131.3743244 | 50.98342396 | -1.365583 | 2.90E-15 | 1.53E-14 |
| MSMEG_3495 | 230 | 77 | 106.266332 | 41.25050923 | -1.365201 | 2.06E-14 | 1.05E-13 |
| MSMEG_6363 | 2350 | 787 | 432.6731498 | 168.0109355 | -1.364722 | 1.51E-132 | 2.42E-131 |
| MSMEG_1268 | 206 | 69 | 67.86210643 | 26.35602683 | -1.364473 | 4.78E-13 | 2.04E-12 |
| MSMEG_3071 | 283 | 95 | 103.9492639 | 40.4603209 | -1.3613 | 2.49E-17 | 1.42E-16 |
| MSMEG_5683 | 137 | 46 | 39.46802234 | 15.36573591 | -1.360967 | 4.19E-09 | 1.33E-08 |
| MSMEG_2061 | 247 | 83 | 75.92136906 | 29.58120797 | -1.359825 | 2.74E-15 | 1.45E-14 |
| MSMEG_4308 | 574 | 193 | 182.542877 | 71.16739061 | -1.358947 | 1.75E-33 | 1.37E-32 |
| MSMEG_6078 | 2054 | 691 | 806.90761 | 314.7546495 | -1.358176 | 2.92E-115 | 4.37E-114 |
| MSMEG_2960 | 520 | 175 | 353.7077428 | 138.0225372 | -1.357654 | 1.92E-30 | 1.44E-29 |
| MSMEG_6676 | 7066 | 2380 | 2532.122724 | 988.914647 | -1.356429 | 0 | 0 |
| MSMEG_5163 | 742 | 250 | 217.1676653 | 84.84026191 | -1.355988 | 1.15E-42 | 1.02E-41 |
| MSMEG_3922 | 92 | 31 | 27.92784593 | 10.9114393 | -1.355863 | 1.66E-06 | 4.25E-06 |
| MSMEG_6280 | 587 | 198 | 418.6642282 | 163.7433529 | -1.354357 | 4.87E-34 | 3.86E-33 |
| MSMEG_6476 | 157 | 53 | 88.04269651 | 34.46198486 | -1.353198 | 3.68E-10 | 1.25E-09 |
| MSMEG_5445 | 154 | 52 | 40.54912585 | 15.87577202 | -1.352844 | 5.46E-10 | 1.84E-09 |
| MSMEG_0592 | 74 | 25 | 16.27609558 | 6.375720281 | -1.352094 | 1.87E-05 | 4.17E-05 |
| MSMEG_0976 | 547 | 185 | 730.6167347 | 286.5132772 | -1.350513 | 1.07E-31 | 8.13E-31 |
| MSMEG_0024 | 2841 | 961 | 1185.833034 | 465.1001003 | -1.350288 | 2.66E-157 | 4.57E-156 |
| MSMEG_1891 | 1199 | 406 | 334.9102298 | 131.4940187 | -1.348777 | 2.39E-67 | 2.70E-66 |
| MSMEG_2444 | 425 | 144 | 131.7363307 | 51.75470762 | -1.347891 | 6.51E-25 | 4.42E-24 |
| MSMEG_6532 | 121 | 41 | 43.15023135 | 16.95322593 | -1.347808 | 4.29E-08 | 1.26E-07 |
| MSMEG_4090 | 301 | 102 | 85.7061069 | 33.67566489 | -1.347692 | 4.39E-18 | 2.55E-17 |
| MSMEG_0230 | 286 | 97 | 205.9827443 | 81.00415124 | -1.346456 | 3.11E-17 | 1.76E-16 |
| MSMEG_4254 | 3778 | 1284 | 462.5681027 | 182.2843931 | -1.343475 | 6.16E-207 | 1.18E-205 |
| MSMEG_5169 | 147 | 50 | 189.4556047 | 74.71896751 | -1.342313 | 1.69E-09 | 5.52E-09 |
| MSMEG_2357 | 1725 | 587 | 314.4481412 | 124.0705674 | -1.341661 | 2.43E-95 | 3.29E-94 |
| MSMEG_4196 | 523 | 178 | 110.7228339 | 43.69444636 | -1.341431 | 4.38E-30 | 3.28E-29 |
| MSMEG_0969 | 4098 | 1395 | 666.0372176 | 262.8884381 | -1.341152 | 1.05E-223 | 2.08E-222 |
| MSMEG_3455 | 376 | 128 | 215.7957335 | 85.17962296 | -1.341086 | 4.64E-22 | 2.94E-21 |
| MSMEG_0260 | 282 | 96 | 76.16320004 | 30.06339634 | -1.341086 | 6.41E-17 | 3.59E-16 |
| MSMEG_5548 | 94 | 32 | 27.95734199 | 11.03541674 | -1.341086 | 1.56E-06 | 4.00E-06 |
| MSMEG_6077 | 14669 | 4996 | 6611.163272 | 2610.781572 | -1.340422 | 0 | 0 |
| MSMEG_4974 | 267 | 91 | 124.9328328 | 49.37162859 | -1.339399 | 4.54E-16 | 2.47E-15 |
| MSMEG_1067 | 211 | 72 | 125.0045292 | 49.45913591 | -1.337671 | 5.62E-13 | 2.39E-12 |
| MSMEG_6864 | 82 | 28 | 26.19093453 | 10.36969323 | -1.336694 | 7.82E-06 | 1.84E-05 |
| MSMEG_1195 | 688 | 235 | 125.1042466 | 49.54755296 | -1.336245 | 7.66E-39 | 6.45E-38 |
| MSMEG_3897 | 3658 | 1251 | 538.5278083 | 213.5465097 | -1.334471 | 2.12E-198 | 3.96E-197 |
| MSMEG_4271 | 552 | 189 | 186.014827 | 73.84838871 | -1.332779 | 2.28E-31 | 1.73E-30 |
| MSMEG_2095 | 1162 | 398 | 277.1535145 | 110.0697725 | -1.332267 | 3.85E-64 | 4.18E-63 |
| MSMEG_3886 | 849 | 291 | 196.1306867 | 77.94739082 | -1.331243 | 2.81E-47 | 2.64E-46 |
| MSMEG_1834 | 2202 | 755 | 341.9960992 | 135.9632459 | -1.330763 | 1.05E-119 | 1.59E-118 |
| MSMEG_3174 | 1402 | 481 | 436.4163264 | 173.6076214 | -1.329875 | 8.19E-77 | 9.85E-76 |
| MSMEG_4899 | 775 | 266 | 283.2504599 | 112.7252722 | -1.329267 | 3.29E-43 | 2.92E-42 |
| MSMEG_2917 | 419 | 144 | 130.4268479 | 51.97400723 | -1.327379 | 4.73E-24 | 3.15E-23 |
| MSMEG_5236 | 622 | 214 | 166.1585408 | 66.28523386 | -1.325801 | 7.13E-35 | 5.71E-34 |
| MSMEG_5088 | 1092 | 376 | 115.9261793 | 46.2825697 | -1.324666 | 7.15E-60 | 7.48E-59 |
| MSMEG_2623 | 90 | 31 | 36.12903804 | 14.42933504 | -1.324154 | 3.28E-06 | 8.09E-06 |
| MSMEG_0753 | 293 | 101 | 80.61601709 | 32.22150531 | -1.323043 | 3.41E-17 | 1.93E-16 |
| MSMEG_6153 | 513 | 177 | 93.51414287 | 37.41139768 | -1.321707 | 6.48E-29 | 4.73E-28 |
| MSMEG_3810 | 2271 | 784 | 709.9279104 | 284.1737208 | -1.320899 | 5.07E-122 | 7.83E-121 |
| MSMEG_0681 | 1028 | 355 | 178.5326806 | 71.4864448 | -1.320447 | 3.37E-56 | 3.40E-55 |
| MSMEG_6330 | 388 | 134 | 90.48699178 | 36.23514119 | -1.320321 | 3.23E-22 | 2.06E-21 |
| MSMEG_0356 | 755 | 261 | 560.2433828 | 224.5644605 | -1.318924 | 1.17E-41 | 1.03E-40 |
| MSMEG_4276 | 9754 | 3376 | 1941.875416 | 779.3127564 | -1.317176 | 0 | 0 |
| MSMEG_0464 | 852 | 295 | 232.6763773 | 93.41259767 | -1.316636 | 1.13E-46 | 1.06E-45 |
| MSMEG_4121 | 950 | 329 | 282.5476052 | 113.4578784 | -1.316337 | 7.95E-52 | 7.84E-51 |
| MSMEG_3122 | 8413 | 2914 | 1292.968579 | 519.2749399 | -1.316117 | 0 | 0 |
| MSMEG_4278 | 2713 | 940 | 544.54489 | 218.7673376 | -1.315654 | 1.61E-144 | 2.65E-143 |
| MSMEG_6428 | 729 | 253 | 300.8655791 | 121.0699135 | -1.313279 | 5.01E-40 | 4.31E-39 |
| MSMEG_4917 | 2992 | 1039 | 740.065431 | 297.9852803 | -1.312412 | 1.38E-158 | 2.38E-157 |
| MSMEG_3469 | 95 | 33 | 35.60676453 | 14.34146713 | -1.311959 | 2.05E-06 | 5.17E-06 |
| MSMEG_0874 | 308 | 107 | 96.2826052 | 38.78391343 | -1.311817 | 8.74E-18 | 5.05E-17 |
| MSMEG_0978 | 449 | 156 | 95.05650556 | 38.29400917 | -1.311667 | 3.16E-25 | 2.16E-24 |
| MSMEG_6187 | 636 | 221 | 179.7002769 | 72.40267951 | -1.311478 | 4.64E-35 | 3.73E-34 |
| MSMEG_5005 | 938 | 326 | 368.4904275 | 148.4949577 | -1.311213 | 6.74E-51 | 6.57E-50 |
| MSMEG_4252 | 161 | 56 | 90.98032887 | 36.69276066 | -1.310059 | 5.95E-10 | 2.00E-09 |
| MSMEG_5071 | 802 | 279 | 442.9836589 | 178.6850737 | -1.309834 | 1.07E-43 | 9.58E-43 |
| MSMEG_2790 | 549 | 191 | 230.4619724 | 92.9674742 | -1.309731 | 1.99E-30 | 1.49E-29 |
| MSMEG_4893 | 296 | 103 | 45.11382509 | 18.20228457 | -1.30945 | 4.17E-17 | 2.35E-16 |
| MSMEG_0331 | 873 | 304 | 182.714118 | 73.7738045 | -1.308408 | 2.44E-47 | 2.29E-46 |
| MSMEG_2612 | 511 | 178 | 109.1257989 | 44.07550258 | -1.307943 | 2.24E-28 | 1.62E-27 |
| MSMEG_4695 | 823 | 287 | 458.0267921 | 185.2011499 | -1.306339 | 1.28E-44 | 1.16E-43 |
| MSMEG_0412 | 1648 | 575 | 315.2760361 | 127.5476125 | -1.30558 | 1.64E-87 | 2.11E-86 |
| MSMEG_1809 | 3741 | 1307 | 928.4552488 | 376.1140784 | -1.303662 | 8.77E-196 | 1.62E-194 |
| MSMEG_5053 | 1033 | 361 | 275.9514029 | 111.8176141 | -1.303267 | 2.25E-55 | 2.26E-54 |
| MSMEG_2369 | 1730 | 605 | 348.1915419 | 141.1881422 | -1.302262 | 2.01E-91 | 2.65E-90 |
| MSMEG_2417 | 2013 | 704 | 724.9008117 | 293.9532086 | -1.302197 | 4.58E-106 | 6.62E-105 |
| MSMEG_3895 | 5225 | 1829 | 1365.981928 | 554.425375 | -1.300873 | 9.62E-272 | 2.04E-270 |
| MSMEG_1134 | 856 | 300 | 212.4452534 | 86.33069894 | -1.299146 | 5.79E-46 | 5.35E-45 |
| MSMEG_4934 | 2599 | 911 | 999.6267996 | 406.2755838 | -1.298931 | 7.23E-136 | 1.17E-134 |
| MSMEG_0947 | 1974 | 692 | 408.4922053 | 166.0402791 | -1.298775 | 1.24E-103 | 1.78E-102 |
| MSMEG_6425 | 821 | 288 | 529.0579983 | 215.1906264 | -1.297811 | 4.36E-44 | 3.92E-43 |
| MSMEG_1647 | 1202 | 422 | 310.921752 | 126.5697214 | -1.296619 | 1.17E-63 | 1.27E-62 |
| MSMEG_1410 | 917 | 322 | 237.2007043 | 96.57689645 | -1.296358 | 5.82E-49 | 5.56E-48 |
| MSMEG_6929 | 3544 | 1246 | 213.9282377 | 87.20937568 | -1.294572 | 1.31E-183 | 2.36E-182 |
| MSMEG_3430 | 182 | 64 | 66.18887464 | 26.98760331 | -1.294292 | 6.79E-11 | 2.42E-10 |
| MSMEG_5682 | 4015 | 1412 | 904.7590339 | 368.937508 | -1.294157 | 1.22E-207 | 2.33E-206 |
| MSMEG_4473 | 162 | 57 | 48.77420135 | 19.89851848 | -1.293457 | 7.73E-10 | 2.58E-09 |
| MSMEG_5613 | 54 | 19 | 30.28220135 | 12.35429646 | -1.293457 | 0.0004274 | 0.000773 |
| MSMEG_5249 | 4170 | 1469 | 623.9065449 | 254.8449412 | -1.29171 | 5.89E-215 | 1.16E-213 |
| MSMEG_6097 | 732 | 258 | 170.7125721 | 69.76616737 | -1.29097 | 3.50E-39 | 2.97E-38 |
| MSMEG_4287 | 2221 | 783 | 650.0396019 | 265.7197003 | -1.290622 | 2.94E-115 | 4.39E-114 |
| MSMEG_4633 | 1267 | 447 | 140.175952 | 57.34230642 | -1.289567 | 1.86E-66 | 2.08E-65 |
| MSMEG_0029 | 751 | 265 | 245.2010906 | 100.322667 | -1.289318 | 4.43E-40 | 3.81E-39 |
| MSMEG_6434 | 487 | 172 | 163.3615423 | 66.8990646 | -1.28801 | 1.61E-26 | 1.11E-25 |
| MSMEG_1529 | 424 | 150 | 146.9247547 | 60.26860115 | -1.285599 | 3.14E-23 | 2.05E-22 |
| MSMEG_6678 | 26562 | 9419 | 12045.10906 | 4952.511535 | -1.282215 | 0 | 0 |
| MSMEG_3039 | 877 | 311 | 159.8672579 | 65.73415072 | -1.28216 | 3.87E-46 | 3.59E-45 |
| MSMEG_4901 | 1056 | 375 | 298.3702711 | 122.8552254 | -1.280145 | 4.48E-55 | 4.48E-54 |
| MSMEG_5538 | 4942 | 1755 | 751.6585276 | 309.5035989 | -1.280121 | 7.03E-251 | 1.45E-249 |
| MSMEG_3638 | 1802 | 640 | 362.6827506 | 149.3560512 | -1.279952 | 1.07E-92 | 1.43E-91 |
| MSMEG_3666 | 684 | 243 | 163.1437211 | 67.20340383 | -1.279537 | 3.04E-36 | 2.49E-35 |
| MSMEG_6106 | 971 | 345 | 220.1603963 | 90.70052444 | -1.279372 | 9.80E-51 | 9.53E-50 |
| MSMEG_5684 | 1514 | 539 | 299.7898633 | 123.7515277 | -1.276505 | 7.43E-78 | 9.01E-77 |
| MSMEG_3762 | 73 | 26 | 21.03040606 | 8.684981164 | -1.275882 | 4.80E-05 | 0.0001003 |
| MSMEG_6251 | 539 | 192 | 181.6340431 | 75.02058536 | -1.275676 | 8.92E-29 | 6.49E-28 |
| MSMEG_6889 | 101 | 36 | 72.03592342 | 29.7715187 | -1.274784 | 1.66E-06 | 4.25E-06 |
| MSMEG_0919 | 1638 | 584 | 516.4436656 | 213.4974241 | -1.274392 | 7.16E-84 | 8.97E-83 |
| MSMEG_4302 | 401 | 143 | 57.87507528 | 23.93062099 | -1.274084 | 9.05E-22 | 5.67E-21 |
| MSMEG_0847 | 143 | 51 | 67.34051257 | 27.84718443 | -1.273943 | 1.14E-08 | 3.51E-08 |
| MSMEG_0368 | 367 | 131 | 55.13434046 | 22.819081 | -1.27271 | 5.06E-20 | 3.05E-19 |
| MSMEG_0750 | 702 | 251 | 213.985846 | 88.71404714 | -1.270281 | 9.32E-37 | 7.68E-36 |
| MSMEG_6906 | 727 | 260 | 175.1054044 | 72.6121376 | -1.269941 | 5.43E-38 | 4.54E-37 |
| MSMEG_3551 | 852 | 305 | 146.9247547 | 60.9854108 | -1.268541 | 3.65E-44 | 3.30E-43 |
| MSMEG_0862 | 659 | 236 | 121.3325982 | 50.38193237 | -1.267989 | 1.63E-34 | 1.30E-33 |
| MSMEG_1959 | 9438 | 3382 | 690.5756149 | 286.9297658 | -1.267102 | 0 | 0 |
| MSMEG_4693 | 1955 | 701 | 1795.236846 | 746.3864462 | -1.266179 | 9.08E-99 | 1.25E-97 |
| MSMEG_0259 | 78 | 28 | 18.9737266 | 7.897448486 | -1.264545 | 2.97E-05 | 6.42E-05 |
| MSMEG_0743 | 707 | 254 | 134.206462 | 55.90600576 | -1.263379 | 1.04E-36 | 8.56E-36 |
| MSMEG_3041 | 462 | 166 | 150.1753024 | 62.56556376 | -1.263207 | 1.54E-24 | 1.03E-23 |
| MSMEG_1561 | 809 | 291 | 200.1045901 | 83.45882249 | -1.261618 | 1.12E-41 | 9.77E-41 |
| MSMEG_5597 | 100 | 36 | 35.48907118 | 14.81384747 | -1.260428 | 2.31E-06 | 5.77E-06 |
| MSMEG_3207 | 722 | 260 | 256.2310939 | 106.9888984 | -1.259984 | 2.64E-37 | 2.20E-36 |
| MSMEG_5735 | 1482 | 534 | 195.8115885 | 81.80920622 | -1.259131 | 1.11E-74 | 1.32E-73 |
| MSMEG_5201 | 258 | 93 | 71.79277786 | 30.00645809 | -1.258566 | 2.73E-14 | 1.38E-13 |
| MSMEG_4830 | 391 | 141 | 170.9749378 | 71.4900407 | -1.257971 | 7.03E-21 | 4.32E-20 |
| MSMEG_2694 | 574 | 207 | 373.1628725 | 156.0370084 | -1.257917 | 6.42E-30 | 4.78E-29 |
| MSMEG_4944 | 97 | 35 | 37.30811834 | 15.60883143 | -1.257127 | 3.44E-06 | 8.47E-06 |
| MSMEG_4505 | 3080 | 1112 | 657.7445413 | 275.3480835 | -1.256271 | 1.19E-152 | 2.00E-151 |
| MSMEG_6475 | 155 | 56 | 68.18364364 | 28.56322686 | -1.255267 | 4.18E-09 | 1.32E-08 |
| MSMEG_3661 | 4776 | 1726 | 1518.858503 | 636.450343 | -1.254868 | 3.39E-235 | 6.90E-234 |
| MSMEG_6416 | 570 | 206 | 182.8539523 | 76.62446432 | -1.254815 | 1.30E-29 | 9.60E-29 |
| MSMEG_2635 | 152 | 55 | 37.47074281 | 15.72107135 | -1.253065 | 6.20E-09 | 1.94E-08 |
| MSMEG_5772 | 548 | 199 | 263.1201489 | 110.7891828 | -1.247905 | 2.65E-28 | 1.91E-27 |
| MSMEG_0358 | 2930 | 1064 | 670.5444412 | 282.339934 | -1.2479 | 7.93E-144 | 1.31E-142 |
| MSMEG_4584 | 2200 | 799 | 380.2758357 | 160.1376912 | -1.247733 | 1.89E-108 | 2.75E-107 |
| MSMEG_2802 | 1361 | 495 | 724.5093882 | 305.5356041 | -1.245664 | 1.28E-67 | 1.45E-66 |
| MSMEG_3125 | 4105 | 1494 | 726.6579735 | 306.6466426 | -1.244699 | 6.49E-200 | 1.22E-198 |
| MSMEG_6423 | 865 | 315 | 225.3367248 | 95.14745117 | -1.243846 | 1.63E-43 | 1.45E-42 |
| MSMEG_3851 | 722 | 263 | 230.6079845 | 97.40104712 | -1.243433 | 1.41E-36 | 1.16E-35 |
| MSMEG_3196 | 118 | 43 | 38.35646251 | 16.20674242 | -1.242876 | 3.74E-07 | 1.02E-06 |
| MSMEG_0863 | 570 | 208 | 135.9531009 | 57.52390122 | -1.240876 | 3.94E-29 | 2.88E-28 |
| MSMEG_4673 | 4771 | 1743 | 1718.083345 | 727.7847197 | -1.239216 | 1.95E-230 | 3.91E-229 |
| MSMEG_3124 | 3201 | 1171 | 911.4460073 | 386.6098391 | -1.237279 | 6.80E-155 | 1.16E-153 |
| MSMEG_1123 | 153 | 56 | 33.75298419 | 14.32450116 | -1.23653 | 7.91E-09 | 2.46E-08 |
| MSMEG_0927 | 112 | 41 | 48.68512581 | 20.66487894 | -1.2363 | 8.28E-07 | 2.18E-06 |
| MSMEG_2776 | 7105 | 2601 | 816.8234602 | 346.7170568 | -1.236265 | 0 | 0 |
| MSMEG_5485 | 2489 | 912 | 1021.496409 | 433.9877996 | -1.234958 | 1.59E-120 | 2.44E-119 |
| MSMEG_5463 | 2025 | 742 | 507.7177956 | 215.710854 | -1.234928 | 2.10E-98 | 2.89E-97 |
| MSMEG_6508 | 60 | 22 | 28.25476052 | 12.01251093 | -1.233956 | 0.0003382 | 0.0006206 |
| MSMEG_5418 | 319 | 117 | 75.3520848 | 32.04506716 | -1.233545 | 7.76E-17 | 4.33E-16 |
| MSMEG_4490 | 578 | 212 | 144.4260344 | 61.42204104 | -1.233502 | 2.93E-29 | 2.16E-28 |
| MSMEG_0775 | 537 | 197 | 158.4309102 | 67.39110732 | -1.233224 | 2.75E-27 | 1.93E-26 |
| MSMEG_3181 | 218 | 80 | 119.5134199 | 50.85350624 | -1.232753 | 5.98E-12 | 2.27E-11 |
| MSMEG_6597 | 166 | 61 | 48.01084504 | 20.45652362 | -1.230799 | 2.09E-09 | 6.79E-09 |
| MSMEG_3634 | 3800 | 1397 | 582.7913025 | 248.425748 | -1.230165 | 7.06E-182 | 1.27E-180 |
| MSMEG_4904 | 1020 | 375 | 340.598295 | 145.1925391 | -1.230104 | 3.71E-50 | 3.59E-49 |
| MSMEG_4022 | 92 | 34 | 33.79269358 | 14.4805359 | -1.222596 | 9.75E-06 | 2.25E-05 |
| MSMEG_2392 | 284 | 105 | 93.97889714 | 40.28765951 | -1.221999 | 5.97E-15 | 3.12E-14 |
| MSMEG_6328 | 576 | 213 | 250.380647 | 107.3565662 | -1.221713 | 9.41E-29 | 6.84E-28 |
| MSMEG_4627 | 473 | 175 | 248.1978892 | 106.4745287 | -1.220983 | 7.14E-24 | 4.74E-23 |
| MSMEG_0693 | 200 | 74 | 79.85041016 | 34.25702228 | -1.2209 | 6.21E-11 | 2.22E-10 |
| MSMEG_2083 | 281 | 104 | 79.09167829 | 33.9413057 | -1.220484 | 8.80E-15 | 4.58E-14 |
| MSMEG_3674 | 945 | 350 | 506.7295372 | 217.6121754 | -1.219457 | 5.47E-46 | 5.07E-45 |
| MSMEG_3303 | 297 | 110 | 45.17251775 | 19.39908597 | -1.219457 | 1.60E-15 | 8.49E-15 |
| MSMEG_5688 | 62 | 23 | 31.62963469 | 13.60507867 | -1.217132 | 0.0003114 | 0.000573 |
| MSMEG_2032 | 442 | 164 | 128.3413865 | 55.21524966 | -1.216848 | 2.68E-22 | 1.70E-21 |
| MSMEG_1287 | 304 | 113 | 152.9627583 | 65.92669448 | -1.214246 | 9.30E-16 | 5.01E-15 |
| MSMEG_5250 | 1739 | 647 | 302.727664 | 130.5952987 | -1.212918 | 1.83E-82 | 2.27E-81 |
| MSMEG_6446 | 258 | 96 | 53.54037671 | 23.09955877 | -1.212762 | 1.44E-13 | 6.62E-13 |
| MSMEG_5255 | 1333 | 496 | 215.2205473 | 92.85514942 | -1.212762 | 1.28E-63 | 1.38E-62 |
| MSMEG_2930 | 86 | 32 | 43.87336425 | 18.9288051 | -1.212762 | 2.17E-05 | 4.80E-05 |
| MSMEG_4919 | 2482 | 924 | 1424.481411 | 614.8904032 | -1.212036 | 9.10E-117 | 1.37E-115 |
| MSMEG_6269 | 341 | 127 | 54.81547194 | 23.6713613 | -1.21144 | 1.94E-17 | 1.11E-16 |
| MSMEG_3636 | 2201 | 820 | 461.9734072 | 199.5636881 | -1.210961 | 1.28E-103 | 1.83E-102 |
| MSMEG_3177 | 59 | 22 | 20.15944309 | 8.716054442 | -1.209709 | 0.0004676 | 0.000844 |
| MSMEG_4925 | 1823 | 681 | 261.0563624 | 113.0747042 | -1.207085 | 1.01E-85 | 1.29E-84 |
| MSMEG_5203 | 214 | 80 | 89.83399287 | 38.93925621 | -1.206036 | 2.08E-11 | 7.61E-11 |
| MSMEG_2627 | 559 | 209 | 145.1076641 | 62.906506 | -1.205843 | 2.04E-27 | 1.44E-26 |
| MSMEG_1967 | 115 | 43 | 40.22939712 | 17.44154184 | -1.205723 | 9.69E-07 | 2.54E-06 |
| MSMEG_5272 | 1400 | 524 | 323.4192713 | 140.3588756 | -1.204285 | 5.13E-66 | 5.71E-65 |
| MSMEG_1215 | 911 | 341 | 314.198243 | 136.3673776 | -1.204177 | 1.47E-43 | 1.32E-42 |
| MSMEG_5067 | 561 | 210 | 94.95943247 | 41.21594659 | -1.204109 | 1.89E-27 | 1.33E-26 |
| MSMEG_3155 | 5036 | 1887 | 904.5391988 | 392.9925392 | -1.202681 | 4.58E-232 | 9.27E-231 |
| MSMEG_0633 | 1211 | 454 | 182.3011044 | 79.24497709 | -1.201932 | 3.59E-57 | 3.66E-56 |
| MSMEG_4480 | 128 | 48 | 146.9247547 | 63.88471722 | -1.201535 | 2.52E-07 | 6.94E-07 |
| MSMEG_5715 | 3510 | 1317 | 779.0119169 | 338.9171101 | -1.200713 | 6.06E-162 | 1.06E-160 |
| MSMEG_5031 | 165 | 62 | 41.6539253 | 18.14823582 | -1.198623 | 4.93E-09 | 1.55E-08 |
| MSMEG_1194 | 93 | 35 | 48.45390846 | 21.14387804 | -1.196373 | 1.23E-05 | 2.81E-05 |
| MSMEG_0913 | 3338 | 1257 | 842.6715312 | 367.9408456 | -1.195497 | 4.59E-153 | 7.77E-152 |
| MSMEG_2404 | 69 | 26 | 21.56980441 | 9.424128497 | -1.194582 | 0.0001753 | 0.0003326 |
| MSMEG_5085 | 483 | 182 | 121.5148228 | 53.09140883 | -1.194582 | 1.41E-23 | 9.34E-23 |
| MSMEG_1185 | 390 | 147 | 84.01855474 | 36.71966151 | -1.194155 | 2.54E-19 | 1.51E-18 |
| MSMEG_5310 | 236 | 89 | 64.21155946 | 28.07772757 | -1.193407 | 2.95E-12 | 1.16E-11 |
| MSMEG_5638 | 1102 | 417 | 217.6224189 | 95.4836096 | -1.188502 | 2.69E-51 | 2.64E-50 |
| MSMEG_0736 | 3615 | 1369 | 951.8512333 | 417.9602288 | -1.18737 | 7.53E-164 | 1.32E-162 |
| MSMEG_3226 | 330 | 125 | 48.38839226 | 21.25240094 | -1.187035 | 1.94E-16 | 1.07E-15 |
| MSMEG_1940 | 615 | 233 | 177.8715042 | 78.13721318 | -1.186754 | 2.68E-29 | 1.98E-28 |
| MSMEG_1480 | 161 | 61 | 39.55666472 | 17.37778261 | -1.186677 | 9.92E-09 | 3.06E-08 |
| MSMEG_2173 | 754 | 286 | 239.786288 | 105.4604856 | -1.185047 | 1.78E-35 | 1.44E-34 |
| MSMEG_5217 | 203 | 77 | 131.9722354 | 58.04275193 | -1.185047 | 1.24E-10 | 4.33E-10 |
| MSMEG_0788 | 614 | 233 | 102.9815061 | 45.31244783 | -1.184406 | 3.63E-29 | 2.66E-28 |
| MSMEG_4286 | 3498 | 1328 | 815.7822094 | 359.1064739 | -1.183772 | 7.34E-158 | 1.26E-156 |
| MSMEG_3372 | 79 | 30 | 51.35865319 | 22.61405919 | -1.183387 | 6.61E-05 | 0.0001345 |
| MSMEG_3429 | 795 | 302 | 155.3260372 | 68.41554823 | -1.182904 | 3.27E-37 | 2.71E-36 |
| MSMEG_0043 | 179 | 68 | 47.81732925 | 21.06259768 | -1.18285 | 1.63E-09 | 5.33E-09 |
| MSMEG_1091 | 121 | 46 | 58.09769712 | 25.60955984 | -1.181799 | 7.55E-07 | 2.00E-06 |
| MSMEG_4632 | 1315 | 500 | 231.6619334 | 102.1338405 | -1.18156 | 2.49E-60 | 2.62E-59 |
| MSMEG_2636 | 71 | 27 | 15.162293 | 6.685609941 | -1.181357 | 0.0001608 | 0.0003056 |
| MSMEG_4968 | 347 | 132 | 63.09763599 | 27.83096592 | -1.180895 | 4.43E-17 | 2.50E-16 |
| MSMEG_5215 | 205 | 78 | 91.27143852 | 40.26673085 | -1.180575 | 1.14E-10 | 3.99E-10 |
| MSMEG_5129 | 155 | 59 | 39.12944498 | 17.27009538 | -1.179979 | 2.16E-08 | 6.53E-08 |
| MSMEG_3761 | 155 | 59 | 60.24692322 | 26.59046431 | -1.179979 | 2.16E-08 | 6.53E-08 |
| MSMEG_3592 | 63 | 24 | 22.14416159 | 9.78139211 | -1.178815 | 0.000393 | 0.0007152 |
| MSMEG_2246 | 244 | 93 | 81.10778313 | 35.84481871 | -1.178076 | 2.13E-12 | 8.47E-12 |
| MSMEG_0187 | 118 | 45 | 54.5192486 | 24.10744046 | -1.177287 | 1.12E-06 | 2.92E-06 |
| MSMEG_0890 | 254 | 97 | 93.29721923 | 41.31211713 | -1.175269 | 8.34E-13 | 3.45E-12 |
| MSMEG_0404 | 377 | 144 | 202.1555931 | 89.53186646 | -1.174993 | 2.71E-18 | 1.58E-17 |
| MSMEG_2408 | 6415 | 2451 | 1707.467937 | 756.4320865 | -1.174576 | 2.69E-284 | 5.80E-283 |
| MSMEG_1602 | 5167 | 1975 | 738.4826921 | 327.2952438 | -1.173971 | 3.30E-229 | 6.59E-228 |
| MSMEG_5702 | 361 | 138 | 159.7585435 | 70.81197571 | -1.173828 | 1.48E-17 | 8.48E-17 |
| MSMEG_4171 | 544 | 208 | 115.5015413 | 51.20624733 | -1.17352 | 1.14E-25 | 7.87E-25 |
| MSMEG_1510 | 481 | 184 | 174.9277401 | 77.58935952 | -1.172828 | 7.39E-23 | 4.78E-22 |
| MSMEG_0384 | 512 | 196 | 130.1478796 | 57.76887924 | -1.171787 | 3.37E-24 | 2.25E-23 |
| MSMEG_4367 | 4162 | 1595 | 729.7145931 | 324.2517867 | -1.170218 | 4.28E-184 | 7.73E-183 |
| MSMEG_3618 | 3793 | 1454 | 787.126546 | 349.8620672 | -1.169809 | 7.37E-168 | 1.30E-166 |
| MSMEG_3890 | 4729 | 1813 | 766.8953255 | 340.9065263 | -1.169653 | 9.68E-209 | 1.87E-207 |
| MSMEG_0076 | 1578 | 605 | 222.5021717 | 98.91299787 | -1.169587 | 6.49E-71 | 7.49E-70 |
| MSMEG_1636 | 542 | 208 | 256.8813453 | 114.3055585 | -1.168207 | 2.08E-25 | 1.43E-24 |
| MSMEG_5795 | 500 | 192 | 126.6592713 | 56.39478485 | -1.167319 | 1.55E-23 | 1.02E-22 |
| MSMEG_6108 | 538 | 207 | 116.5863098 | 52.01233614 | -1.164473 | 4.10E-25 | 2.80E-24 |
| MSMEG_0840 | 612 | 236 | 206.23383 | 92.21280283 | -1.161242 | 3.13E-28 | 2.23E-27 |
| MSMEG_6862 | 197 | 76 | 74.21583763 | 33.19821202 | -1.160622 | 4.53E-10 | 1.53E-09 |
| MSMEG_2480 | 189 | 73 | 56.2121025 | 25.17454444 | -1.158915 | 1.07E-09 | 3.54E-09 |
| MSMEG_6742 | 132 | 51 | 51.57996707 | 23.10723814 | -1.158466 | 3.62E-07 | 9.86E-07 |
| MSMEG_3013 | 370 | 143 | 173.1278957 | 77.58398779 | -1.158007 | 1.32E-17 | 7.58E-17 |
| MSMEG_3894 | 2354 | 910 | 700.1232238 | 313.8196635 | -1.157673 | 3.22E-103 | 4.60E-102 |
| MSMEG_1883 | 9068 | 3506 | 1076.18229 | 482.4551827 | -1.157456 | 0 | 0 |
| MSMEG_0416 | 1595 | 617 | 2169.86096 | 973.2560623 | -1.156711 | 1.93E-70 | 2.22E-69 |
| MSMEG_3217 | 1406 | 544 | 196.7392429 | 88.26231407 | -1.156415 | 2.78E-62 | 2.99E-61 |
| MSMEG_4476 | 248 | 96 | 170.26794 | 76.42283929 | -1.155731 | 3.03E-12 | 1.18E-11 |
| MSMEG_5128 | 248 | 96 | 134.953108 | 60.57217632 | -1.155731 | 3.03E-12 | 1.18E-11 |
| MSMEG_3063 | 204 | 79 | 83.72248591 | 37.59324142 | -1.155142 | 2.59E-10 | 8.91E-10 |
| MSMEG_0407 | 604 | 234 | 122.2349199 | 54.90917843 | -1.154537 | 1.21E-27 | 8.56E-27 |
| MSMEG_3156 | 271 | 105 | 184.3361506 | 82.81352232 | -1.154401 | 3.15E-13 | 1.39E-12 |
| MSMEG_5042 | 1810 | 702 | 228.4654691 | 102.7424318 | -1.152944 | 2.74E-79 | 3.35E-78 |
| MSMEG_2065 | 116 | 45 | 49.25801024 | 22.15654932 | -1.152625 | 2.06E-06 | 5.19E-06 |
| MSMEG_3182 | 701 | 272 | 481.2815563 | 216.531378 | -1.152305 | 9.90E-32 | 7.57E-31 |
| MSMEG_6729 | 384 | 149 | 97.27432035 | 43.76470283 | -1.152291 | 4.37E-18 | 2.54E-17 |
| MSMEG_0865 | 451 | 175 | 66.26306437 | 29.81286803 | -1.15227 | 5.63E-21 | 3.47E-20 |
| MSMEG_5642 | 662 | 257 | 99.24917103 | 44.67584306 | -1.15156 | 5.01E-30 | 3.75E-29 |
| MSMEG_1663 | 255 | 99 | 119.317874 | 53.71199155 | -1.151494 | 1.74E-12 | 6.95E-12 |
| MSMEG_5554 | 922 | 358 | 283.3987946 | 127.5912344 | -1.151304 | 3.70E-41 | 3.23E-40 |
| MSMEG_0740 | 800 | 311 | 149.1621875 | 67.23569223 | -1.149583 | 7.50E-36 | 6.10E-35 |
| MSMEG_6933 | 2117 | 823 | 498.4610668 | 224.6885567 | -1.149554 | 5.32E-92 | 7.05E-91 |
| MSMEG_4910 | 725 | 282 | 527.3289463 | 237.8282542 | -1.148783 | 1.31E-32 | 1.01E-31 |
| MSMEG_3162 | 2730 | 1063 | 475.2423937 | 214.563837 | -1.147257 | 1.01E-117 | 1.53E-116 |
| MSMEG_3127 | 624 | 243 | 402.1098549 | 181.567091 | -1.147087 | 3.14E-28 | 2.24E-27 |
| MSMEG_4593 | 172 | 67 | 77.04590795 | 34.79899231 | -1.146673 | 8.07E-09 | 2.50E-08 |
| MSMEG_3876 | 249 | 97 | 51.38239314 | 23.20905457 | -1.146586 | 3.74E-12 | 1.45E-11 |
| MSMEG_0104 | 77 | 30 | 19.24014645 | 8.691798261 | -1.146393 | 0.000123 | 0.0002403 |
| MSMEG_1526 | 469 | 183 | 149.1508873 | 67.47996104 | -1.144242 | 1.58E-21 | 9.86E-21 |
| MSMEG_2393 | 748 | 292 | 165.5116212 | 74.91701778 | -1.143567 | 2.32E-33 | 1.81E-32 |
| MSMEG_0580 | 292 | 114 | 80.94722334 | 36.6433095 | -1.143432 | 5.96E-14 | 2.91E-13 |
| MSMEG_2521 | 338 | 132 | 50.98620851 | 23.08770068 | -1.142983 | 6.55E-16 | 3.54E-15 |
| MSMEG_3200 | 796 | 311 | 109.9173917 | 49.79485477 | -1.142351 | 2.44E-35 | 1.96E-34 |
| MSMEG_6180 | 693 | 271 | 224.2706057 | 101.6902107 | -1.14106 | 6.32E-31 | 4.77E-30 |
| MSMEG_0983 | 404 | 158 | 136.1412865 | 61.73569003 | -1.140928 | 1.12E-18 | 6.62E-18 |
| MSMEG_3482 | 212 | 83 | 183.2238117 | 83.17539653 | -1.139378 | 1.83E-10 | 6.36E-10 |
| MSMEG_3676 | 309 | 121 | 61.35101243 | 27.85603886 | -1.139097 | 1.34E-14 | 6.92E-14 |
| MSMEG_0651 | 74 | 29 | 23.94808777 | 10.88197826 | -1.13797 | 0.0001831 | 0.0003468 |
| MSMEG_2403 | 1362 | 534 | 132.6999442 | 60.32615207 | -1.137312 | 7.48E-59 | 7.76E-58 |
| MSMEG_4285 | 1241 | 487 | 398.1083419 | 181.1461851 | -1.136007 | 1.10E-53 | 1.09E-52 |
| MSMEG_0469 | 275 | 108 | 69.42320883 | 31.61305594 | -1.134898 | 4.40E-13 | 1.89E-12 |
| MSMEG_5843 | 239 | 94 | 81.28476012 | 37.06890999 | -1.132775 | 1.59E-11 | 5.87E-11 |
| MSMEG_1644 | 150 | 59 | 36.48793577 | 16.64105217 | -1.132673 | 9.81E-08 | 2.79E-07 |
| MSMEG_4366 | 747 | 294 | 104.3277488 | 47.60990333 | -1.131789 | 8.43E-33 | 6.54E-32 |
| MSMEG_2105 | 320 | 126 | 73.00608929 | 33.33115681 | -1.131145 | 6.46E-15 | 3.38E-14 |
| MSMEG_4932 | 3876 | 1527 | 679.5708224 | 310.427886 | -1.130366 | 1.79E-162 | 3.13E-161 |
| MSMEG_5450 | 137 | 54 | 70.87567392 | 32.39225098 | -1.129642 | 3.76E-07 | 1.02E-06 |
| MSMEG_6278 | 558 | 220 | 172.962053 | 79.0696922 | -1.129259 | 7.99E-25 | 5.41E-24 |
| MSMEG_0126 | 700 | 276 | 137.8650513 | 63.02835372 | -1.129184 | 9.88E-31 | 7.45E-30 |
| MSMEG_3887 | 4923 | 1942 | 4410.430289 | 2017.302778 | -1.128492 | 2.81E-205 | 5.33E-204 |
| MSMEG_5114 | 1863 | 736 | 291.8132388 | 133.6720736 | -1.126347 | 1.22E-78 | 1.49E-77 |
| MSMEG_1188 | 1387 | 548 | 160.2080462 | 73.39376318 | -1.126217 | 5.37E-59 | 5.58E-58 |
| MSMEG_5220 | 372 | 147 | 86.4810265 | 39.62469802 | -1.125984 | 5.49E-17 | 3.08E-16 |
| MSMEG_2017 | 172 | 68 | 93.5965104 | 42.90529156 | -1.125299 | 1.34E-08 | 4.08E-08 |
| MSMEG_6480 | 96 | 38 | 33.42364088 | 15.34040603 | -1.123532 | 2.38E-05 | 5.24E-05 |
| MSMEG_2144 | 2240 | 887 | 826.9131922 | 379.6699777 | -1.12299 | 9.69E-94 | 1.30E-92 |
| MSMEG_6216 | 2155 | 854 | 442.2106793 | 203.1938492 | -1.121877 | 4.10E-90 | 5.36E-89 |
| MSMEG_3209 | 545 | 216 | 154.5829948 | 71.03783227 | -1.121722 | 4.85E-24 | 3.23E-23 |
| MSMEG_3192 | 474 | 188 | 137.0912081 | 63.0463351 | -1.120652 | 4.42E-21 | 2.73E-20 |
| MSMEG_6410 | 1785 | 708 | 252.6596215 | 116.198792 | -1.1206 | 7.99E-75 | 9.50E-74 |
| MSMEG_2104 | 723 | 287 | 118.0295529 | 54.32567064 | -1.119442 | 2.83E-31 | 2.14E-30 |
| MSMEG_1322 | 529 | 210 | 92.30783282 | 42.48864803 | -1.119376 | 2.62E-23 | 1.72E-22 |
| MSMEG_6510 | 68 | 27 | 18.63970768 | 8.581529178 | -1.119073 | 0.0004065 | 0.0007384 |
| MSMEG_0042 | 141 | 56 | 55.09678301 | 25.37265365 | -1.118694 | 3.13E-07 | 8.56E-07 |
| MSMEG_2613 | 2427 | 964 | 348.9103519 | 160.6911087 | -1.118566 | 8.81E-101 | 1.24E-99 |
| MSMEG_2892 | 83 | 33 | 15.47557695 | 7.134333902 | -1.117143 | 9.32E-05 | 0.0001848 |
| MSMEG_6421 | 191 | 76 | 22.48608025 | 10.37444126 | -1.115999 | 2.70E-09 | 8.68E-09 |
| MSMEG_0188 | 103 | 41 | 49.78042675 | 22.97608251 | -1.115446 | 1.33E-05 | 3.03E-05 |
| MSMEG_6580 | 309 | 123 | 151.3324973 | 69.84729082 | -1.115446 | 3.63E-14 | 1.82E-13 |
| MSMEG_1635 | 7373 | 2935 | 2531.019197 | 1168.234548 | -1.115388 | 9.36E-301 | 2.06E-299 |
| MSMEG_6790 | 422 | 168 | 100.9808575 | 46.61295328 | -1.115279 | 8.40E-19 | 4.97E-18 |
| MSMEG_3210 | 193 | 77 | 51.18497772 | 23.67809014 | -1.112168 | 2.46E-09 | 7.96E-09 |
| MSMEG_5906 | 739 | 295 | 138.1391778 | 63.93890273 | -1.111357 | 1.40E-31 | 1.07E-30 |
| MSMEG_5233 | 273 | 109 | 55.70896949 | 25.79049695 | -1.11107 | 1.30E-12 | 5.26E-12 |
| MSMEG_4165 | 1033 | 413 | 157.7684736 | 73.13759726 | -1.109124 | 2.39E-43 | 2.13E-42 |
| MSMEG_1344 | 1560 | 624 | 790.3538528 | 366.5661015 | -1.108425 | 1.64E-64 | 1.81E-63 |
| MSMEG_4471 | 70 | 28 | 34.28244276 | 15.90019629 | -1.108425 | 0.0003694 | 0.0006736 |
| MSMEG_4182 | 390 | 156 | 57.4154853 | 26.62930096 | -1.108425 | 2.47E-17 | 1.41E-16 |
| MSMEG_3764 | 115 | 46 | 120.6881914 | 55.9751808 | -1.108425 | 4.63E-06 | 1.12E-05 |
| MSMEG_2758 | 6494 | 2599 | 1021.551774 | 474.0510494 | -1.107648 | 3.18E-262 | 6.65E-261 |
| MSMEG_6073 | 437 | 175 | 101.9144727 | 47.32201275 | -1.106776 | 3.37E-19 | 2.01E-18 |
| MSMEG_0793 | 367 | 147 | 106.5640019 | 49.49171769 | -1.106461 | 2.34E-16 | 1.29E-15 |
| MSMEG_2923 | 262 | 105 | 72.0866774 | 33.49760453 | -1.105675 | 4.45E-12 | 1.72E-11 |
| MSMEG_2366 | 963 | 386 | 205.4528637 | 95.48693454 | -1.105432 | 2.56E-40 | 2.21E-39 |
| MSMEG_3345 | 207 | 83 | 65.54617289 | 30.47374442 | -1.104945 | 7.97E-10 | 2.66E-09 |
| MSMEG_5545 | 1483 | 595 | 376.9712997 | 175.369812 | -1.104054 | 4.90E-61 | 5.18E-60 |
| MSMEG_4950 | 461 | 185 | 94.07265543 | 43.7728618 | -1.103739 | 4.36E-20 | 2.63E-19 |
| MSMEG_5103 | 289 | 116 | 59.63659284 | 27.75515804 | -1.103442 | 3.87E-13 | 1.69E-12 |
| MSMEG_0214 | 670 | 269 | 114.9995159 | 53.53579106 | -1.103052 | 1.86E-28 | 1.34E-27 |
| MSMEG_2059 | 391 | 157 | 116.7633721 | 54.36260489 | -1.102901 | 3.02E-17 | 1.71E-16 |
| MSMEG_3225 | 1162 | 467 | 56.19702599 | 26.18754702 | -1.101613 | 4.88E-48 | 4.62E-47 |
| MSMEG_0420 | 1161 | 467 | 861.5133343 | 401.8069083 | -1.100371 | 6.48E-48 | 6.13E-47 |
| MSMEG_0391 | 2816 | 1133 | 786.5781544 | 366.9525202 | -1.099997 | 1.39E-113 | 2.06E-112 |
| MSMEG_0791 | 211 | 85 | 46.27033319 | 21.61274015 | -1.098205 | 6.64E-10 | 2.23E-09 |
| MSMEG_5440 | 201 | 81 | 51.81030823 | 24.20894547 | -1.097699 | 1.71E-09 | 5.59E-09 |
| MSMEG_4990 | 196 | 79 | 57.59450384 | 26.91676085 | -1.097426 | 2.75E-09 | 8.82E-09 |
| MSMEG_0768 | 233 | 94 | 124.9396637 | 58.44441283 | -1.096095 | 9.16E-11 | 3.24E-10 |
| MSMEG_3393 | 575 | 232 | 128.783131 | 60.2490016 | -1.095934 | 2.08E-24 | 1.39E-23 |
| MSMEG_0480 | 171 | 69 | 57.10030239 | 26.7154272 | -1.095825 | 2.94E-08 | 8.79E-08 |
| MSMEG_5997 | 104 | 42 | 60.15816727 | 28.16963909 | -1.09462 | 1.63E-05 | 3.67E-05 |
| MSMEG_3206 | 1143 | 462 | 220.9670982 | 103.560489 | -1.093358 | 9.86E-47 | 9.21E-46 |
| MSMEG_1196 | 519 | 210 | 57.42014133 | 26.93933859 | -1.091842 | 4.64E-22 | 2.93E-21 |
| MSMEG_1604 | 1892 | 766 | 240.0532261 | 112.6901402 | -1.090993 | 6.14E-76 | 7.33E-75 |
| MSMEG_3105 | 1121 | 454 | 266.5091424 | 125.1506434 | -1.090519 | 1.11E-45 | 1.02E-44 |
| MSMEG_0366 | 772 | 313 | 322.2327006 | 151.4842158 | -1.088935 | 6.58E-32 | 5.04E-31 |
| MSMEG_2768 | 1240 | 503 | 734.6237735 | 345.5270189 | -1.088207 | 3.51E-50 | 3.40E-49 |
| MSMEG_6666 | 382 | 155 | 183.4158702 | 86.29308208 | -1.087802 | 1.51E-16 | 8.35E-16 |
| MSMEG_0941 | 69 | 28 | 17.97483701 | 8.457551215 | -1.087667 | 0.0004985 | 0.0008968 |
| MSMEG_3319 | 101 | 41 | 38.24587687 | 18.00187908 | -1.087157 | 2.41E-05 | 5.31E-05 |
| MSMEG_0866 | 224 | 91 | 28.92016261 | 13.62275165 | -1.086058 | 2.86E-10 | 9.83E-10 |
| MSMEG_2511 | 1607 | 653 | 405.6839876 | 191.1419031 | -1.085712 | 2.79E-64 | 3.04E-63 |
| MSMEG_3534 | 113 | 46 | 56.47107918 | 26.654848 | -1.083114 | 8.33E-06 | 1.96E-05 |
| MSMEG_5490 | 140 | 57 | 18.07510163 | 8.532932352 | -1.08289 | 6.87E-07 | 1.82E-06 |
| MSMEG_6279 | 658 | 268 | 236.9521779 | 111.9026419 | -1.082352 | 3.49E-27 | 2.45E-26 |
| MSMEG_4347 | 189 | 77 | 38.46091224 | 18.16850684 | -1.081953 | 7.85E-09 | 2.44E-08 |
| MSMEG_6748 | 481 | 196 | 63.21181306 | 29.86620054 | -1.08168 | 2.84E-20 | 1.72E-19 |
| MSMEG_2153 | 522 | 213 | 193.6735403 | 91.63262469 | -1.079694 | 8.26E-22 | 5.18E-21 |
| MSMEG_6142 | 495 | 202 | 115.0755594 | 54.45026531 | -1.07957 | 9.30E-21 | 5.71E-20 |
| MSMEG_1334 | 294 | 120 | 87.79649975 | 41.55103559 | -1.079279 | 6.34E-13 | 2.67E-12 |
| MSMEG_5797 | 245 | 100 | 310.3152146 | 146.8614189 | -1.079279 | 5.26E-11 | 1.88E-10 |
| MSMEG_0990 | 1622 | 663 | 458.2922156 | 217.2080385 | -1.07719 | 4.58E-64 | 4.97E-63 |
| MSMEG_2106 | 203 | 83 | 70.67707394 | 33.50667633 | -1.076794 | 2.52E-09 | 8.14E-09 |
| MSMEG_1927 | 868 | 355 | 239.7193366 | 113.679572 | -1.076373 | 4.85E-35 | 3.90E-34 |
| MSMEG_4742 | 831 | 340 | 235.7036123 | 111.8188101 | -1.075811 | 1.37E-33 | 1.08E-32 |
| MSMEG_0593 | 342 | 140 | 116.3154308 | 55.20901488 | -1.075067 | 1.03E-14 | 5.34E-14 |
| MSMEG_3903 | 1487 | 609 | 650.2294947 | 308.7761332 | -1.074388 | 1.22E-58 | 1.26E-57 |
| MSMEG_1638 | 554 | 227 | 299.2511548 | 142.1748118 | -1.073691 | 7.38E-23 | 4.77E-22 |
| MSMEG_4323 | 18891 | 7750 | 1492.234162 | 709.8301913 | -1.071928 | 0 | 0 |
| MSMEG_4631 | 1070 | 439 | 383.4377744 | 182.4090462 | -1.071815 | 1.60E-42 | 1.41E-41 |
| MSMEG_2998 | 173 | 71 | 70.99995129 | 33.78633089 | -1.071378 | 4.34E-08 | 1.28E-07 |
| MSMEG_4755 | 1245 | 511 | 207.8651359 | 98.92451666 | -1.071248 | 3.71E-49 | 3.55E-48 |
| MSMEG_2975 | 621 | 255 | 201.8590103 | 96.10975157 | -1.070593 | 2.54E-25 | 1.74E-24 |
| MSMEG_2176 | 207 | 85 | 139.5111203 | 66.42447662 | -1.070593 | 2.09E-09 | 6.79E-09 |
| MSMEG_2428 | 168 | 69 | 43.76482055 | 20.84182264 | -1.07029 | 6.98E-08 | 2.02E-07 |
| MSMEG_4834 | 202 | 83 | 35.33190529 | 16.83311597 | -1.069669 | 3.35E-09 | 1.07E-08 |
| MSMEG_4998 | 348 | 143 | 62.35343248 | 29.70899045 | -1.069569 | 7.77E-15 | 4.06E-14 |
| MSMEG_6126 | 292 | 120 | 63.46453901 | 30.24128626 | -1.069431 | 1.12E-12 | 4.55E-12 |
| MSMEG_3507 | 1914 | 787 | 471.8355377 | 224.9542391 | -1.068653 | 2.47E-74 | 2.93E-73 |
| MSMEG_3376 | 1036 | 426 | 249.5312227 | 118.9721947 | -1.068596 | 4.93E-41 | 4.28E-40 |
| MSMEG_1061 | 1595 | 656 | 571.5731311 | 272.5747935 | -1.068286 | 3.28E-62 | 3.51E-61 |
| MSMEG_5741 | 367 | 151 | 127.1730778 | 60.67039182 | -1.067729 | 1.58E-15 | 8.41E-15 |
| MSMEG_0754 | 729 | 300 | 393.7799492 | 187.8962271 | -1.067454 | 2.58E-29 | 1.90E-28 |
| MSMEG_3208 | 294 | 121 | 104.8443638 | 50.03269115 | -1.067306 | 1.02E-12 | 4.17E-12 |
| MSMEG_0607 | 374 | 154 | 97.7755485 | 46.68207094 | -1.066605 | 9.04E-16 | 4.87E-15 |
| MSMEG_1568 | 1712 | 705 | 199.9484738 | 95.47159648 | -1.066485 | 1.82E-66 | 2.04E-65 |
| MSMEG_3594 | 2560 | 1055 | 458.6919171 | 219.1817127 | -1.065398 | 2.56E-98 | 3.52E-97 |
| MSMEG_5635 | 512 | 211 | 597.0275746 | 285.284134 | -1.065398 | 5.36E-21 | 3.30E-20 |
| MSMEG_5267 | 1849 | 762 | 342.1459338 | 163.4933821 | -1.06538 | 1.60E-71 | 1.86E-70 |
| MSMEG_0940 | 1485 | 612 | 305.5787965 | 146.0222108 | -1.065357 | 8.58E-58 | 8.80E-57 |
| MSMEG_1961 | 412 | 170 | 77.21045782 | 36.94014261 | -1.063607 | 3.77E-17 | 2.13E-16 |
| MSMEG_4024 | 344 | 142 | 56.66156459 | 27.11996964 | -1.063015 | 1.50E-14 | 7.69E-14 |
| MSMEG_5615 | 327 | 135 | 161.2228013 | 77.17616845 | -1.062828 | 6.70E-14 | 3.24E-13 |
| MSMEG_3906 | 264 | 109 | 68.53027427 | 32.8076993 | -1.062707 | 1.70E-11 | 6.27E-11 |
| MSMEG_1655 | 678 | 280 | 177.8838994 | 85.17962296 | -1.062356 | 3.54E-27 | 2.48E-26 |
| MSMEG_2556 | 818 | 338 | 80.12296622 | 38.38761675 | -1.061575 | 2.03E-32 | 1.57E-31 |
| MSMEG_1660 | 121 | 50 | 30.65154365 | 14.68614189 | -1.061504 | 5.66E-06 | 1.35E-05 |
| MSMEG_3980 | 329 | 136 | 112.4145216 | 53.88106382 | -1.060978 | 6.09E-14 | 2.97E-13 |
| MSMEG_2410 | 708 | 293 | 214.0385315 | 102.7062943 | -1.059346 | 3.46E-28 | 2.47E-27 |
| MSMEG_0102 | 1002 | 415 | 178.6633546 | 85.79986293 | -1.058197 | 3.83E-39 | 3.24E-38 |
| MSMEG_3631 | 6164 | 2553 | 1419.504997 | 681.7040044 | -1.058171 | 2.05E-231 | 4.13E-230 |
| MSMEG_3919 | 210 | 87 | 74.52704948 | 35.80013139 | -1.057799 | 2.29E-09 | 7.43E-09 |
| MSMEG_4525 | 403 | 167 | 75.71697716 | 36.38106658 | -1.057429 | 1.15E-16 | 6.39E-16 |
| MSMEG_5612 | 234 | 97 | 86.81917323 | 41.72941125 | -1.056949 | 2.87E-10 | 9.85E-10 |
| MSMEG_1528 | 545 | 226 | 177.9422029 | 85.55819906 | -1.056431 | 5.71E-22 | 3.61E-21 |
| MSMEG_2641 | 330 | 137 | 95.82049219 | 46.12493417 | -1.054787 | 7.33E-14 | 3.53E-13 |
| MSMEG_0917 | 867 | 360 | 145.0840118 | 69.85117144 | -1.054532 | 6.53E-34 | 5.16E-33 |
| MSMEG_2079 | 1901 | 790 | 419.3753133 | 202.0777842 | -1.053331 | 3.59E-72 | 4.19E-71 |
| MSMEG_4779 | 125 | 52 | 45.45939192 | 21.92742769 | -1.051842 | 4.66E-06 | 1.13E-05 |
| MSMEG_5850 | 995 | 414 | 353.1162583 | 170.3592459 | -1.051563 | 1.67E-38 | 1.40E-37 |
| MSMEG_1692 | 161 | 67 | 38.77850083 | 18.71158931 | -1.051325 | 1.98E-07 | 5.51E-07 |
| MSMEG_0539 | 557 | 232 | 181.8601964 | 87.82965567 | -1.05005 | 3.25E-22 | 2.06E-21 |
| MSMEG_3068 | 72 | 30 | 85.31114788 | 41.21594659 | -1.049532 | 0.0005477 | 0.0009803 |
| MSMEG_0099 | 120 | 50 | 54.41657581 | 26.29000709 | -1.049532 | 7.53E-06 | 1.78E-05 |
| MSMEG_2242 | 108 | 45 | 15.99584023 | 7.727989986 | -1.049532 | 2.18E-05 | 4.82E-05 |
| MSMEG_6422 | 2047 | 853 | 826.2499254 | 399.2209801 | -1.049391 | 4.10E-77 | 4.95E-76 |
| MSMEG_3703 | 1335 | 557 | 432.0364483 | 209.0090308 | -1.047588 | 8.97E-51 | 8.73E-50 |
| MSMEG_6643 | 642 | 268 | 99.71003437 | 48.26245022 | -1.046838 | 3.02E-25 | 2.06E-24 |
| MSMEG_6829 | 91 | 38 | 44.27202873 | 21.4359316 | -1.046364 | 0.000103 | 0.0002028 |
| MSMEG_4712 | 14635 | 6115 | 2897.902675 | 1403.971413 | -1.045496 | 0 | 0 |
| MSMEG_4900 | 3768 | 1576 | 2407.010764 | 1167.331181 | -1.044029 | 3.32E-139 | 5.40E-138 |
| MSMEG_2057 | 2055 | 860 | 189.893315 | 92.14399464 | -1.043227 | 1.10E-76 | 1.32E-75 |
| MSMEG_6191 | 1541 | 645 | 711.9844245 | 345.5399799 | -1.042993 | 6.20E-58 | 6.37E-57 |
| MSMEG_3229 | 2812 | 1177 | 779.5328494 | 378.3260989 | -1.04298 | 3.30E-104 | 4.74E-103 |
| MSMEG_4248 | 1969 | 825 | 597.7166157 | 290.3850783 | -1.041494 | 2.33E-73 | 2.74E-72 |
| MSMEG_0026 | 291 | 122 | 225.0268611 | 109.3885684 | -1.040635 | 3.73E-12 | 1.45E-11 |
| MSMEG_1414 | 43154 | 18123 | 10710.1197 | 5215.237523 | -1.03817 | 0 | 0 |
| MSMEG_4354 | 645 | 271 | 93.27408147 | 45.44031067 | -1.037504 | 5.16E-25 | 3.51E-24 |
| MSMEG_0058 | 157 | 66 | 65.53177979 | 31.94235861 | -1.036724 | 3.82E-07 | 1.03E-06 |
| MSMEG_5792 | 421 | 177 | 141.2221957 | 68.84380485 | -1.036568 | 7.75E-17 | 4.33E-16 |
| MSMEG_5910 | 88 | 37 | 48.97491823 | 23.87610643 | -1.036475 | 0.0001516 | 0.0002888 |
| MSMEG_0876 | 264 | 111 | 63.37930595 | 30.89849068 | -1.036475 | 4.29E-11 | 1.54E-10 |
| MSMEG_3458 | 88 | 37 | 23.17092906 | 11.2962224 | -1.036475 | 0.0001516 | 0.0002889 |
| MSMEG_5977 | 76 | 32 | 30.1791388 | 14.73377262 | -1.034425 | 0.0004466 | 0.0008067 |
| MSMEG_6113 | 622 | 262 | 94.99708671 | 46.39721666 | -1.033845 | 4.77E-24 | 3.17E-23 |
| MSMEG_5338 | 216 | 91 | 110.9641504 | 54.20521461 | -1.03359 | 2.72E-09 | 8.75E-09 |
| MSMEG_6047 | 178 | 75 | 40.60963717 | 19.83997429 | -1.033412 | 6.86E-08 | 1.99E-07 |
| MSMEG_3561 | 1329 | 560 | 215.0473557 | 105.0673763 | -1.03334 | 1.78E-49 | 1.71E-48 |
| MSMEG_4216 | 408 | 172 | 176.3097056 | 86.18173617 | -1.032658 | 2.85E-16 | 1.56E-15 |
| MSMEG_5451 | 83 | 35 | 30.64008703 | 14.98134072 | -1.032254 | 0.000247 | 0.0004599 |
| MSMEG_6520 | 460 | 194 | 175.0916766 | 85.62096815 | -1.032074 | 3.86E-18 | 2.25E-17 |
| MSMEG_2743 | 680 | 287 | 330.823951 | 161.897694 | -1.030981 | 4.95E-26 | 3.42E-25 |
| MSMEG_3072 | 2497 | 1054 | 416.8989914 | 204.043915 | -1.030818 | 5.06E-91 | 6.67E-90 |
| MSMEG_2118 | 360 | 152 | 100.9406712 | 49.41718584 | -1.030423 | 1.74E-14 | 8.93E-14 |
| MSMEG_5257 | 1068 | 451 | 305.2833424 | 149.4786379 | -1.03021 | 6.63E-40 | 5.68E-39 |
| MSMEG_1512 | 1299 | 549 | 287.432615 | 140.854256 | -1.029021 | 4.44E-48 | 4.21E-47 |
| MSMEG_3694 | 627 | 265 | 92.12182119 | 45.14520017 | -1.02897 | 4.70E-24 | 3.13E-23 |
| MSMEG_4911 | 537 | 227 | 90.68803824 | 44.45005612 | -1.028727 | 8.10E-21 | 4.97E-20 |
| MSMEG_6312 | 1298 | 549 | 229.7690742 | 112.6834048 | -1.02791 | 5.81E-48 | 5.50E-47 |
| MSMEG_0414 | 546 | 231 | 117.282041 | 57.53360498 | -1.027505 | 4.20E-21 | 2.60E-20 |
| MSMEG_1115 | 182 | 77 | 58.1310986 | 28.51665638 | -1.027505 | 5.63E-08 | 1.64E-07 |
| MSMEG_5172 | 104 | 44 | 44.41911188 | 21.7901361 | -1.027505 | 4.24E-05 | 8.94E-05 |
| MSMEG_4683 | 156 | 66 | 42.60271697 | 20.89908965 | -1.027505 | 5.05E-07 | 1.35E-06 |
| MSMEG_2064 | 508 | 215 | 280.5931405 | 137.696383 | -1.026989 | 1.00E-19 | 6.01E-19 |
| MSMEG_3138 | 1273 | 539 | 748.1408509 | 367.2945342 | -1.026372 | 5.83E-47 | 5.46E-46 |
| MSMEG_2592 | 1535 | 650 | 599.8124959 | 294.5040155 | -1.026224 | 2.97E-56 | 3.00E-55 |
| MSMEG_4186 | 1256 | 532 | 314.9103957 | 154.6606123 | -1.025836 | 2.56E-46 | 2.38E-45 |
| MSMEG_4568 | 1135 | 481 | 290.5219453 | 142.7574866 | -1.025081 | 5.68E-42 | 4.98E-41 |
| MSMEG_2476 | 99 | 42 | 50.50538442 | 24.8440567 | -1.023536 | 6.88E-05 | 0.0001393 |
| MSMEG_5258 | 278 | 118 | 163.3803272 | 80.40956407 | -1.022795 | 2.17E-11 | 7.93E-11 |
| MSMEG_1942 | 596 | 253 | 183.9646088 | 90.54808659 | -1.022672 | 9.93E-23 | 6.38E-22 |
| MSMEG_6096 | 400 | 170 | 108.0329079 | 53.23726435 | -1.020962 | 1.03E-15 | 5.51E-15 |
| MSMEG_5458 | 247 | 105 | 54.32696768 | 26.77802518 | -1.020619 | 3.03E-10 | 1.04E-09 |
| MSMEG_0679 | 3005 | 1278 | 392.103808 | 193.3562312 | -1.019974 | 2.57E-107 | 3.73E-106 |
| MSMEG_0594 | 1521 | 647 | 228.0332162 | 112.4718695 | -1.01968 | 3.40E-55 | 3.41E-54 |
| MSMEG_3816 | 4310 | 1834 | 429.610375 | 211.9666601 | -1.019191 | 6.91E-153 | 1.17E-151 |
| MSMEG_5018 | 263 | 112 | 51.52161398 | 25.44031406 | -1.018061 | 8.85E-11 | 3.13E-10 |
| MSMEG_6474 | 244 | 104 | 52.56545476 | 25.97853603 | -1.016795 | 4.40E-10 | 1.49E-09 |
| MSMEG_2571 | 129 | 55 | 95.72370381 | 47.32201275 | -1.016365 | 6.06E-06 | 1.44E-05 |
| MSMEG_6778 | 225 | 96 | 112.4424143 | 55.62750887 | -1.015316 | 2.19E-09 | 7.11E-09 |
| MSMEG_0837 | 288 | 123 | 139.1918729 | 68.92824752 | -1.013908 | 1.33E-11 | 4.94E-11 |
| MSMEG_5798 | 681 | 291 | 138.5813822 | 68.66279856 | -1.013133 | 2.23E-25 | 1.53E-24 |
| MSMEG_3752 | 365 | 156 | 388.6053294 | 192.5800171 | -1.012848 | 2.67E-14 | 1.35E-13 |
| MSMEG_1626 | 124 | 53 | 30.98413194 | 15.35551026 | -1.012773 | 9.78E-06 | 2.25E-05 |
| MSMEG_6201 | 4381 | 1874 | 407.9070661 | 202.3150994 | -1.011637 | 1.64E-153 | 2.78E-152 |
| MSMEG_6509 | 77 | 33 | 19.50552778 | 9.692853647 | -1.00889 | 0.0005349 | 0.0009593 |
| MSMEG_0846 | 518 | 222 | 71.52915689 | 35.54488026 | -1.00889 | 1.49E-19 | 8.94E-19 |
| MSMEG_2700 | 84 | 36 | 94.93599534 | 47.17640656 | -1.00889 | 0.0002956 | 0.0005462 |
| MSMEG_0406 | 2932 | 1257 | 351.9472065 | 174.9522648 | -1.008398 | 7.58E-103 | 1.08E-101 |
| MSMEG_6329 | 757 | 325 | 323.3198817 | 160.949869 | -1.006351 | 9.63E-28 | 6.81E-27 |
| MSMEG_3066 | 498 | 214 | 161.877274 | 80.65681112 | -1.005032 | 9.52E-19 | 5.62E-18 |
| MSMEG_3506 | 463 | 199 | 72.98944359 | 36.3749892 | -1.004741 | 1.58E-17 | 9.02E-17 |
| MSMEG_1355 | 221 | 95 | 81.17592697 | 40.4603209 | -1.004544 | 4.17E-09 | 1.32E-08 |
| MSMEG_4470 | 2107 | 906 | 227.6253369 | 113.4893212 | -1.004105 | 6.34E-74 | 7.49E-73 |
| MSMEG_0953 | 1083 | 466 | 249.4036196 | 124.4316749 | -1.003129 | 8.71E-39 | 7.33E-38 |
| MSMEG_3843 | 79 | 34 | 38.6901854 | 19.3073812 | -1.002815 | 0.0004818 | 0.0008685 |
| MSMEG_2924 | 367 | 158 | 84.78205184 | 42.32195103 | -1.002353 | 3.76E-14 | 1.88E-13 |
| MSMEG_1931 | 2290 | 986 | 722.0122065 | 360.459692 | -1.002185 | 6.12E-80 | 7.53E-79 |
| MSMEG_0829 | 123 | 53 | 34.62020082 | 17.29701156 | -1.001091 | 1.29E-05 | 2.93E-05 |
| MSMEG_6145 | 420 | 181 | 129.0970648 | 64.50841738 | -1.000897 | 6.00E-16 | 3.25E-15 |
